# Semi-Supervised Off-Policy Reinforcement Learning and Value Estimation for Dynamic Treatment Regimes

**Published:** 2023

**Authors:** Aaron Sonabend-W, Nilanjana Laha, Ashwin N. Ananthakrishnan, Tianxi Cai, Rajarshi Mukherjee

**Affiliations:** Department of Biostatistics, Harvard University, Boston, USA; Department of Statistics, Texas A& M, Texas, USA; Division of Gastroenterology, Massachusetts General Hospital, Boston, USA; Department of Biostatistics, Harvard University, Boston, USA; Department of Biostatistics, Harvard University, Boston, USA

**Keywords:** semi-supervised learning, *Q*-learning, reinforcement-learning, dynamical treatment regime, doubly robust value function, off-policy learning

## Abstract

Reinforcement learning (RL) has shown great promise in estimating dynamic treatment regimes which take into account patient heterogeneity. However, health-outcome information, used as the reward for RL methods, is often not well coded but rather embedded in clinical notes. Extracting precise outcome information is a resource-intensive task, so most of the available well-annotated cohorts are small. To address this issue, we propose a semi-supervised learning (SSL) approach that efficiently leverages a small-sized labeled data set with actual outcomes observed and a large unlabeled data set with outcome surrogates. In particular, we propose a semi-supervised, efficient approach to *Q*-learning and doubly robust off-policy value estimation. Generalizing SSL to dynamic treatment regimes brings interesting challenges: 1) Feature distribution for *Q*-learning is unknown as it includes previous outcomes. 2) The surrogate variables we leverage in the modified SSL framework are predictive of the outcome but not informative of the optimal policy or value function. We provide theoretical results for our *Q* function and value function estimators to understand the degree of efficiency gained from SSL. Our method is at least as efficient as the supervised approach, and robust to bias from mis-specification of the imputation models.

## 1. Introduction

Finding optimal treatment strategies incorporating patient heterogeneity is a cornerstone of personalized medicine. When treatment options change over time, optimal dynamic treatment regimes (DTR) can be learned using longitudinal patient data. With the increasing availability of large-scale longitudinal data such as electronic health records (EHR) data in recent years, reinforcement learning (RL) has shown promise in estimating such optimal DTR (Kosorok and Laber, 2019; Schulte et al., 2014; Sonabend-W et al., 2020b; Zhou et al., 2023; Chakraborty and Moodie, 2013). Existing RL methods include G-estimation (Robins, 2004), *Q*-learning (Watkins, 1989; Murphy, 2005), A-learning (Murphy, 2003) and directly maximizing the value function (Zhao et al., 2015). G-estimation and *A*-learning attempt to model only the component of the outcome regression relevant to the treatment contrast, while *Q*-learning posits complete models for the outcome regression. Although G-estimation and *A*-learning models can be more efficient and robust to mis-specification, *Q*-learning is widely adopted due to its ease of implementation, flexibility, and interpretability (Watkins, 1989; Chakraborty and Moodie, 2013; Schulte et al., 2014).

However, learning DTR with EHR data often faces an additional challenge of whether outcome information is readily available. Outcome information, such as the development of a clinical event or whether a patient is considered to have responded, is often not well coded but rather embedded in clinical notes. Proxy variables, such as diagnostic codes or mentions of relevant clinical terms in clinical notes via natural language processing (NLP), while predictive of the true outcome, are often not sufficiently accurate to be used directly in place of the outcome (Hong et al., 2019; Zhang et al., 2019; Sonabend W. et al., 2020; Cheng et al., 2020). On the other hand, extracting precise outcome information often requires manual chart review, which is resource intensive, particularly when the outcome needs to be annotated over time. This challenge indicates the need for a semi-supervised learning (SSL) approach that can efficiently leverage a small-sized labeled data ℒ with true outcome observed, and a large-sized unlabeled data 𝒰 for predictive modeling. It is worthwhile to note that the SSL setting differs from the standard missing data setting in that the probability of missing tends to 1 asymptotically, which violates the positivity assumption required by the classical missing data methods (Chakrabortty et al., 2018).

While SSL methods have been well developed for prediction, classification, and regression tasks (e.g. Chapelle et al., 2006; Zhu, 2008; Blitzer and Zhu, 2008; Zhixing and Shaohong, 2011; Qiao et al., 2018; Chakrabortty et al., 2018), there is a paucity of literature on SSL methods for estimating optimal treatment rules. Recently, Cheng et al. (2020) and Kallus and Mao (2020) proposed SSL methods for estimating an average causal treatment effect. Finn et al. (2016) proposed a semi-supervised RL method that achieves impressive empirical results and outperforms simple approaches such as direct imputation of the reward. However, there are no theoretical guarantees, and the approach lacks causal validity and interpretability within a domain context. Additionally, this method does not leverage available surrogates. In this work, we fill this gap by proposing a theoretically justified SSL approach to *Q*-learning using a large set of unlabeled data 𝒰, which contains sequential observations on features **O**, treatment assignment *A*, and surrogates **W** that are imperfect proxies of *Y*, as well as a small set of labeled data ℒ which contains true outcome *Y* at multiple stages along with **O**, *A* and **W**. We will also develop a robust and efficient SSL approach to estimating the value function of the derived optimal DTR, defined as the expected counterfactual outcome under the derived DTR.

To describe the main contributions of our proposed SSL approach to RL, we first note two crucial distinctions between the proposed framework and classical SSL methods. First, existing SSL literature often assumes that 𝒰 is large enough that the feature distribution is known (Wasserman and Lafferty, 2008). However, under the RL setting, the outcome of the stage t−1, denoted by $Y_{t-1}$, becomes a feature of stage t for predicting $Y_{t}$. As such, the feature distribution for predicting $Y_{t}$ can not be viewed as known in the *Q*-learning procedure. Our methods for estimating an optimal DTR and its associated value function carefully adapt to this sequentially missing data structure. We do not make a Markov decision process (MDP) assumption or have a partially observed MDP framework (Kaelbling et al., 1998), as biological mechanisms of the patient’s features or treatment may take more than one time step to manifest in the outcome. Second, we modify the SSL framework to handle the use of surrogate variables **W**, which are predictive of the outcome through the joint law ${\mathbb{P}}_{Y,O,A,W}$, but are not part of the conditional distribution of interest ${\mathbb{P}}_{Y|O,A}$. To address these issues, we propose a two-step fitting procedure for finding an optimal DTR and estimating its value function in the SSL setting. Our method consists of using the outcome surrogates (W) and features (O,A) for non-parametric estimation of the missing outcomes (Y). We subsequently use these imputations to estimate *Q* functions, learn the optimal treatment rule and estimate its associated value function. We provide theoretical results to understand when and to what degree efficiency can be gained from W and O,A.

We further show that our approach is robust to mis-specification of the imputation models. To account for potential mis-specification in the models for the *Q* function, we provide a double robust value function estimator for the derived DTR. If either the regression models for the *Q* functions or the propensity score functions are correctly specified, our value function estimators are consistent for the true value function.

We organize the rest of the paper as follows. In Section 2 we formalize the problem mathematically and provide some notation to be used in developing and analyzing the methods. In Section 3, we discuss traditional *Q*-learning and propose an SSL estimation procedure for the optimal DTR. Section 4 details an SSL doubly robust estimator of the value function for the derived DTR. In Section 5, we provide theoretical guarantees for our approach and discuss the implications of our assumptions and results. Section 6 is devoted to numerical experiments and real data analysis with an inflammatory bowel disease (IBD) data set. We end with a discussion of the methods and possible extensions in Section 7. The proposed method has been implemented in R, and the code can be found at github.com/asonabend/SSOPRL. A reference table with the main notation used can be found in Appendix A. An extension of the SSL algorithms to a general time horizon and simulations can be found in appendices B and C. Finally, all the technical proofs and supporting lemmas are collected in Appendices D and E.

## 2. Problem Setup

We consider a longitudinal observational study with outcomes, confounders and treatment indices potentially available over multiple stages. The proposed semi-supervised methods are valid for a general time horizon, we describe them in detail in Appendix B and show empirical results in Section 6. However, for ease of presentation, in the main text we will use two time points of (binary) treatment allocation as follows. For time point t∈{1, 2}, let $O_{t}\in {\mathbb{R}}^{d_{t}^{o}}$ denote the vector of covariates measured prior at stage *t* of dimension $d_{t}^{o}$; $A_{t}\in \{0, 1\}$ a treatment indicator variable; and $Y_{t+1}\in \mathbb{R}$ the outcome observed at stage t+1, for which higher values of $Y_{t+1}$ are considered beneficial. Additionally we observe surrogates $W_{t}\in {\mathbb{R}}^{d_{t}^{\omega }}$, a $d_{t}^{\omega }$-dimensional vector of post-treatment covariates potentially predictive of $Y_{t+1}$. In the labeled data where $Y={\left(Y_{2},Y_{3}\right)}^{⊤}$ is annotated, we observe a random sample of *n* independent and identically distributed (iid) random vectors, denoted by

$$ℒ={\left\{L_{i}={\left({\vec{U}}_{i}^{⊤},Y_{i}^{⊤}\right)}^{⊤}\right\}}_{i=1}^{n},\;\text{where}\;U_{ti}={\left(O_{ti}^{⊤},A_{ti},W_{ti}^{⊤}\right)}^{⊤}\;\text{and}\;{\vec{U}}_{i}={\left(U_{1i}^{⊤},U_{2i}^{⊤}\right)}^{⊤}.$$

We additionally observe an unlabeled set consisting of *N* iid random vectors,

$$𝒰={\left\{{\vec{U}}_{j}\right\}}_{j=1}^{N}$$

with N≫n. We denote the entire data as 𝕊=(ℒ∪𝒰). To operationalize our statistical arguments we denote the joint distribution of the observation vector $L_{i}$ in ℒ as ℙ. In order to connect to the unlabeled set, we assume that any observation vector ${\vec{U}}_{j}$ in 𝒰 has the distribution induced by ℙ.

We are interested in finding the optimal DTR and estimating its *value function* to be defined as expected counterfactual outcomes under the derived regime. To this end, let $Y_{t+1}^{\left(a\right)}$ be the potential outcome for a patient at time t+1 had the patient been assigned at time t to treatment a∈{0, 1}. A dynamic treatment regime is a set of functions $𝒟=\left(d_{1},d_{2}\right)$, where $d_{t}(\cdot )\in \{0, 1\}$, t=1, 2 map from the patient’s history up to time *t* to the treatment choice {0, 1}. We define the patient’s history as $H_{1}\equiv {\left[H_{10}^{⊤},H_{11}^{⊤}\right]}^{⊤}$ with $H_{1k}=\phi_{1k}\left(O_{1}\right)$, $H_{2}={\left[H_{20}^{⊤},H_{21}^{⊤}\right]}^{⊤}$ with $H_{2k}=\phi_{2k}\left(O_{1},A_{1},O_{2}\right)$, where $\left\{\phi_{tk}(\cdot ),t=1, 2,k=0, 1\right\}$ are pre-specified basis functions. We then define features derived from patient history for regression modeling as $X_{1}\equiv {\left[H_{10}^{⊤},A_{1}H_{11}^{⊤}\right]}^{⊤}$ and $X_{2}\equiv {\left[H_{20}^{⊤},A_{2}H_{21}^{⊤}\right]}^{⊤}$. For ease of presentation, we also use the check symbol (i.e., ${\overset{ˇ}{H}}_{2}$) to denote vectors that contain outcome $Y_{2}$ when applicable. Hence, we let ${\overset{ˇ}{H}}_{2}={\left(Y_{2},H_{2}^{⊤}\right)}^{⊤}$, ${\overset{ˇ}{X}}_{2}={\left(Y_{2},X_{2}^{⊤}\right)}^{⊤}$, for consistency we also write ${\overset{ˇ}{H}}_{1}=H_{1}$, ${\overset{ˇ}{X}}_{1}=X_{1}$, and finally we define $\Sigma_{t}=𝔼\left[{\overset{ˇ}{X}}_{t}{\overset{ˇ}{X}}_{t}^{⊤}\right]$. We collect this and the main notation used throughout the paper in Table 8, Appendix A.

Let ${𝔼}_{𝒟}$ be the expectation with respect to the measure that generated the data under regime 𝒟. Then these sets of rules 𝒟 have an associated value function which we can write as $V(𝒟)={𝔼}_{𝒟}\;\left[Y_{2}^{\left(d_{1}\right)}+Y_{3}^{\left(d_{2}\right)}\right]$. Thus, an optimal dynamic treatment regime is a rule $\bar{𝒟}=\left({\overset{‾}{d}}_{1},{\overset{‾}{d}}_{2}\right)$ such that $\overset{‾}{V}=V\;(\bar{𝒟})\geq V\;|(𝒟)$ for all 𝒟 in a suitable class of admissible decisions (Chakraborty and Moodie, 2013). To identify $\bar{𝒟}$ and $\overset{‾}{V}$ from the observed data we will require the following sets of standard assumptions (Robins, 1997; Schulte et al., 2014): (i) consistency – $Y_{t+1}=Y_{t+1}^{\left(0\right)}I\left(A_{t}=0\right)+Y_{t+1}^{\left(1\right)}I\left(A_{t}=1\right)$ for t=1,2, (ii) sequential ignorability, also known as no unmeasured confounding – for outcomes, intermediate covariates and surrogates: $Y_{t+1}^{\left(a\right)},O_{t}^{\left(a\right)},W_{t}^{\left(a\right)}⫫A_{t}|H_{t}$ for a∈{0, 1},t=1, 2 (iii) positivity – $\mathbb{P}\left(A_{t}|H_{t}\right)>\nu$, for $t=1, 2,A_{t}\in \{0, 1\}$, for some fixed ν>0.

We will develop SSL inference methods to derive optimal DTR $\bar{𝒟}$ as well the associated value function $\overset{‾}{V}$ by leveraging the richness of the unlabeled data and the predictive power of surrogate variables which allows us to gain crucial statistical efficiency. Our main contributions in this regard can be described as follows. First, we provide a systematic generalization of the *Q*-learning framework, with theoretical guarantees to the semi-supervised setting with improved efficiency. Second, we provide a doubly robust estimator of the value function in the semi-supervised setup. Third, our *Q*-learning procedure and value function estimator are flexible enough to allow for standard off-the-shelf machine learning tools and are shown to perform well in finite-sample numerical examples.

## 3. Semi-Supervised *Q*-Learning

In this section we propose a semi-supervised *Q*-learning approach to derive an optimal DTR. To this end, we first recall the basic mechanism of traditional linear parametric *Q* learning (Chakraborty and Moodie, 2013) and then detail our proposed method. We defer the theoretical guarantees to Section 5.

### 3.1 Traditional *Q*-Learning

*Q*-learning is a backward recursive algorithm that identifies optimal DTR by optimizing two stage *Q* functions. Under the consistency, sequential ignorability and positivity assumptions (i)-(iii), the optimal treatment is identifiable and the *Q* functions can be expressed as:

$$Q_{2}\left({\overset{ˇ}{H}}_{2},A_{2}\right)\equiv 𝔼\left[Y_{3}|{\overset{ˇ}{H}}_{2},A_{2}\right],\;\text{and}\;Q_{1}\left({\overset{ˇ}{H}}_{1},A_{1}\right)\equiv 𝔼\left[Y_{2}+\underset{a_{2}}{\max}Q_{2}\left({\overset{ˇ}{H}}_{2},a_{2}\right)|{\overset{ˇ}{H}}_{1},A_{1}\right]$$

(Sutton, 2018; Murphy, 2005). In order to perform inference one typically proceeds by positing models for the *Q* functions. In its simplest form one assumes a (working) linear model for some parameters $\theta_{t}={\left(\beta_{t}^{⊤},\gamma_{t}^{⊤}\right)}^{⊤},t=1, 2$, as follows:

(1) $$Q_{1}\left({\overset{ˇ}{H}}_{1},A_{1};\theta_{1}^{0}\right)={\overset{ˇ}{X}}_{1}^{⊤}\theta_{1}^{0}=H_{10}^{⊤}\beta_{1}^{0}+A_{1}\left(H_{11}^{⊤}\gamma_{1}^{0}\right),\;Q_{2}\left({\overset{ˇ}{H}}_{2},A_{2};\theta_{2}^{0}\right)={\overset{ˇ}{X}}_{2}^{⊤}\theta_{2}^{0}=Y_{2}\beta_{21}^{0}+H_{20}^{⊤}\beta_{22}^{0}+A_{2}\left(H_{21}^{⊤}\gamma_{2}^{0}\right).$$

Typical *Q*-learning consists of performing a least squares regression for the second stage to estimate ${\hat{\theta }}_{2}$ followed by defining the stage 1 pseudo-outcome for i=1,…,n as

$${\hat{Y}}_{2i}^{\text{*}}=Y_{2i}+\underset{a_{2}}{\max}\;Q_{2}\left({\overset{ˇ}{H}}_{2i},a_{2};{\hat{\theta }}_{2}\right)=Y_{2i}\left(1+{\hat{\beta }}_{21}\right)+H_{20i}^{⊤}{\hat{\beta }}_{22}+{\left[H_{21i}^{⊤}{\hat{\gamma }}_{2}\right]}_{+},$$

where $\left[x{]}_{+}=xI(x>0\right)$. One then proceeds to estimate ${\hat{\theta }}_{1}$ using least squares again, with ${\hat{Y}}_{2}^{*}$ as the outcome variable. Indeed, valid inference on $\bar{𝒟}$ using the method described above crucially depends on the validity of the model assumed. However as we shall see, even without validity of this model we will be able to provide valid inference on suitable analogues of the *Q* function working model parameters, and on the value function using a double robust type estimator. To that end it will be instructive to define the least square projections of $Y_{3}$ and $Y_{2}^{*}$ onto ${\overset{ˇ}{X}}_{2}$ and ${\overset{ˇ}{X}}_{1}$ respectively. The linear regression working models given by (1) have $\theta_{1}^{0},\theta_{2}^{0}$ as unknown regression parameters. To account for the potential mis-specification of the working models in (1), we define the target population parameters ${\bar{\theta }}_{1},{\bar{\theta }}_{2}$ as the population solutions to the expected normal equations

$$𝔼\left\{{\overset{ˇ}{X}}_{1}\left({\bar{Y}}_{2}^{\text{*}}-{\overset{ˇ}{X}}_{1}^{⊤}{\bar{\theta }}_{1}\right)\right\}=0,\;\text{and}\;𝔼\left\{{\overset{ˇ}{X}}_{2}\left(Y_{3}-{\overset{ˇ}{X}}_{2}^{⊤}{\bar{\theta }}_{2}\right)\right\}=0,$$

where ${\overset{‾}{Y}}_{2}^{*}=Y_{2}+\underset{a_{2}}{max}\;Q_{2}\left({\overset{ˇ}{H}}_{2},a_{2};{\bar{\theta }}_{2}\right)$. As these are linear in the parameters, uniqueness and existence for ${\bar{\theta }}_{1},{\bar{\theta }}_{2}$ are well defined. In fact, $Q_{1}\left({\overset{ˇ}{H}}_{1},A_{1};{\bar{\theta }}_{1}\right)={\overset{ˇ}{X}}_{1}^{⊤}{\bar{\theta }}_{1},Q_{2}\left({\overset{ˇ}{H}}_{2},A_{2};{\bar{\theta }}_{2}\right)={\overset{ˇ}{X}}_{2}^{⊤}{\bar{\theta }}_{2}$ are the $L_{2}$ projection of $𝔼\left(Y_{2}^{*}|{\overset{ˇ}{X}}_{1}\right)\in {ℒ}_{2}\left({\mathbb{P}}_{{\overset{ˇ}{X}}_{1}}\right)$, $𝔼\left(Y_{3}|{\overset{ˇ}{X}}_{2}\right)\in {ℒ}_{2}\left({\mathbb{P}}_{{\overset{ˇ}{X}}_{2}}\right)$ onto the subspace of all linear functions of ${\overset{ˇ}{X}}_{1},{\overset{ˇ}{X}}_{2}$ respectively. Therefore, *Q* functions in (1) are the best linear predictors of ${\overset{‾}{Y}}_{2}^{*}$ conditional on ${\overset{ˇ}{X}}_{1}$ and $Y_{3}$ conditional on ${\overset{ˇ}{X}}_{2}$.

Traditionally, one only has access to labeled data ℒ, and hence proceeds to estimate ($\theta_{1},\theta_{2}$) in (1) by solving the following sample version set of normal equations:

(2) $$\;{\mathbb{P}}_{n}\left[{\overset{ˇ}{X}}_{2}\left(Y_{3}-{\overset{ˇ}{X}}_{2}^{⊤}\theta_{2}\right)\right]\equiv {\mathbb{P}}_{n}\left[\begin{array}{c} Y_{2}\left\{Y_{3}-\left(Y_{2},X_{2}^{⊤}\right)\theta_{2}\right\}\\ X_{2}\left\{Y_{3}-\left(Y_{2},X_{2}^{⊤}\right)\theta_{2}\right\} \end{array}\right]=0,\;{\mathbb{P}}_{n}\left[X_{1}\left\{Y_{2}\left(1+\beta_{21}\right)+H_{20}^{⊤}\beta_{22}+{\left[H_{21}^{⊤}\gamma_{2}\right]}_{+}-X_{1}^{⊤}\theta_{1}\right\}\right]=0.$$

(Chakraborty and Moodie, 2013), where ${\mathbb{P}}_{n}$ denotes the empirical measure: i.e. for a measurable function $f\;:\;{\mathbb{R}}^{p}\mapsto \mathbb{R}$ and random sample ${\left\{L_{i}\right\}}_{i=1}^{n}$, ${\mathbb{P}}_{n}f=\frac{1}{n}\sum_{i=1}^{n}f\left(L_{i}\right)$. The asymptotic distribution for the *Q* function parameters in the fully-supervised setting has been well studied (see Laber et al., 2014). It is worth recalling that we use the check symbol: “ˇ” to denote the entire set of features available, including outcomes when available (for t=2), this symbol is used for both the patient history ${\overset{ˇ}{H}}_{t}$, and the linear features for the *Q*–functions ${\overset{ˇ}{X}}_{t}$. We also use *bar* (i.e., $\bar{\theta }$) over the variables to denote population parameters, and we use *hat* (i.e., $\hat{\theta }$) over the variables to denote estimated parameters. For more detail on notation see Table 8.

### 3.2 Semi-Supervised *Q*-Learning

We next detail our robust imputation-based semi-supervised *Q*-learning approach that leverages the unlabeled data 𝒰 to replace the unobserved $Y_{t}$ in (2) with their properly imputed values for subjects in 𝒰. Our SSL procedure includes three key steps: (i) imputation, (ii) refitting, and (iii) projection to the unlabeled data. In step (i), we develop flexible imputation models for the conditional mean functions $\left\{\mu_{t}(\cdot ),\mu_{2t}(\cdot ),t=2,3\right\}$, where $\mu_{t}(\vec{U})=𝔼\left(Y_{t}|\vec{U}\right)$ and $\mu_{2t}(\vec{U})=𝔼\left(Y_{2}Y_{t}|\vec{U}\right)$. The refitting in step (ii) will ensure the validity of the SSL estimators under potential mis-specifications of the imputation models.

#### Step I: Imputation

Our first imputation step involves weakly parametric or non-parametric prediction modeling to approximate the conditional mean functions $\left\{\mu_{t}(\cdot ),\mu_{2t}(\cdot ),t=2,3\right\}$. Commonly used models such as non-parametric kernel smoothing, basis function expansion or kernel machine regression can be used. We denote the corresponding estimated mean functions as $\left\{{\hat{m}}_{t}(\cdot ),{\hat{m}}_{2t}(\cdot ),t=2,3\right\}$ under the corresponding imputation models $\left\{m_{t}(\vec{U}),m_{2t}(\vec{U}),t=2, 3\right\}$. Theoretical properties of our proposed SSL estimators on specific choices of the imputation models are provided in Section 5. We also provide additional simulation results comparing different imputation models in Section 6.

#### Step II: Refitting

To overcome the potential bias in the fitting from the imputation model, especially under model mis-specification, we update the imputation model with an additional refitting step by expanding it to include linear effects of $\left\{X_{t},t=1, 2\right\}$ with cross-fitting to control overfitting bias. Specifically, to ensure the validity of the SSL algorithm from the refitted imputation model, we note that the final imputation models for $\left\{Y_{t},Y_{2}Y_{t},t=2, 3\right\}$, denoted by $\left\{{\overset{‾}{\mu }}_{t}(\vec{U}),{\overset{‾}{\mu }}_{2t},t=2, 3\right\}$, need to satisfy

$$\;𝔼\;\left[\vec{X}\left\{Y_{2}-{\bar{\mu }}_{2}(\vec{U})\right\}\right]=0,\;𝔼\;\left\{Y_{2}^{2}-{\bar{\mu }}_{22}(\vec{U})\right\}=0,\;𝔼\;\left[X_{2}\left\{Y_{3}-{\bar{\mu }}_{3}(\vec{U})\right\}\right]=0,\;𝔼\;\left\{Y_{2}Y_{3}-{\bar{\mu }}_{23}(\vec{U})\right\}=0,$$

where $\vec{X}={\left(1,X_{1}^{⊤},X_{2}^{⊤}\right)}^{⊤}$. We thus propose a refitting step that expands $\left\{m_{t}(\vec{U}),m_{2t}(\vec{U}),t=2, 3\right\}$ to additionally adjust for linear effects of $X_{1}$ and/or $X_{2}$ to ensure the subsequent projection step is unbiased. To this end, let $\left\{{ℐ}_{k},k=1,\ldots ,K\right\}$ denote K random equal sized partitions of the labeled index set {1,…,n}, and let $\left\{{\hat{m}}_{t}^{\left(-k\right)}(\vec{U}),{\hat{m}}_{2t}^{\left(-k\right)}(\vec{U}),t=2,3\right\}$ be the counterpart of $\left\{{\hat{m}}_{t}(\vec{U}),{\hat{m}}_{2t}(\vec{U}),t=2,3\right\}$ with labeled observations in $\{1,..,n\}\setminus {ℐ}_{k}$. We then obtain ${\hat{\eta }}_{2},{\hat{\eta }}_{22},{\hat{\eta }}_{3},{\hat{\eta }}_{23}$ respectively as the solutions to

(3) $$\;\overset{K}{\underset{k=1}{\Sigma }}\underset{i\in {ℐ}_{k}}{\Sigma }{\vec{X}}_{i}\left\{Y_{2i}-{\hat{m}}_{2}^{\left(-k\right)}\left({\vec{U}}_{i}\right)-\eta_{2}^{⊤}{\vec{X}}_{i}\right\}=0,\;\overset{K}{\underset{k=1}{\Sigma }}\underset{i\in {ℐ}_{k}}{\Sigma }\left\{Y_{2i}^{2}-{\hat{m}}_{22}^{\left(-k\right)}\left({\vec{U}}_{i}\right)-\eta_{22}\right\}=0,\;\overset{K}{\underset{k=1}{\Sigma }}\underset{i\in {ℐ}_{k}}{\Sigma }X_{2i}\left\{Y_{3i}-{\hat{m}}_{3}^{\left(-k\right)}\left({\vec{U}}_{i}\right)-\eta_{3}^{⊤}X_{2i}\right\}=0,\overset{K}{\underset{k=1}{\Sigma }}\underset{i\in {ℐ}_{k}}{\Sigma }\left\{Y_{2i}Y_{3i}-{\hat{m}}_{23}^{\left(-k\right)}\left({\vec{U}}_{i}\right)-\eta_{23}\right\}=0.$$

Finally, we impute $Y_{2},Y_{3},Y_{2}^{2}$ and $Y_{2}Y_{3}$ respectively as ${\hat{\mu }}_{2}(\vec{U})=K^{-1}\sum_{k=1}^{K}{\hat{m}}_{2}^{\left(-k\right)}(\vec{U})+{\hat{\eta }}_{2}^{⊤}\vec{X}$, ${\hat{\mu }}_{3}(\vec{U})=K^{-1}\sum_{k=1}^{K}{\hat{m}}_{3}^{\left(-k\right)}(\vec{U})+{\hat{\eta }}_{3}^{⊤}X_{2},{\hat{\mu }}_{22}(\vec{U})=K^{-1}\sum_{k=1}^{K}{\hat{m}}_{22}^{\left(-k\right)}(\vec{U})+{\hat{\eta }}_{22}$, and ${\hat{\mu }}_{23}(\vec{U})=K^{-1}\sum_{k=1}^{K}{\hat{m}}_{23}^{\left(-k\right)}(\vec{U})+{\hat{\eta }}_{23}$.

#### Step III: Projection

In the last step, we proceed to estimate $\hat{\theta }$ by replacing $\left\{Y_{t},Y_{2}Y_{t},t=2, 3\right\}$ in (2) with their the imputed values $\left\{{\hat{\mu }}_{t}(\vec{U}),{\hat{\mu }}_{2t}(\vec{U}),t=2,3\right\}$ and project to the unlabeled data. Specifically, we obtain the final SSL estimators for $\theta_{1}$ and $\theta_{2}$ via the following steps:

1. Stage 2 regression: we obtain the SSL estimator for $\theta_{2}$ as
$${\hat{\theta }}_{2}={\left({\hat{\beta }}_{2}^{⊤},{\hat{\gamma }}_{2}^{⊤}\right)}^{⊤}\text{: the solution to}\;{\mathbb{P}}_{N}\;\left[\begin{array}{c} {\hat{\mu }}_{23}(\vec{U})-\left[{\hat{\mu }}_{22}(\vec{U}),{\hat{\mu }}_{2}(\vec{U})X_{2}^{⊤}\right]\theta_{2}\\ X_{2}\left\{{\hat{\mu }}_{3}(\vec{U})-\left[{\hat{\mu }}_{2}(\vec{U}),X_{2}^{⊤}\right]\theta_{2}\right\} \end{array}\right]=0.$$
2. We compute the imputed pseudo-outcome:
$${\tilde{Y}}_{2}^{\text{*}}={\hat{\mu }}_{2}(\vec{U})+\underset{a\in \{0,1\}}{\max}Q_{2}\;\left(H_{2},{\hat{\mu }}_{2}(\vec{U}),a;{\hat{\theta }}_{2}\right).$$
3. Stage 1 regression: we estimate ${\hat{\theta }}_{1}={\left({\hat{\beta }}_{1}^{⊤},{\hat{\gamma }}_{1}^{⊤}\right)}^{⊤}$ as the solution to:
$${\mathbb{P}}_{N}\;\left\{X_{1}\left({\tilde{Y}}_{2}^{\text{*}}-X_{1}^{⊤}\theta_{1}\right)\right\}=0.$$

Based on the SSL estimator for the *Q*-learning model parameters, we can then obtain an estimate for the optimal treatment protocol as:

$$\hat{d_{t}}\equiv \hat{d_{t}}\left(H_{t}\right)\equiv d_{t}\left(H_{t};\hat{\theta_{t}}\right),\;\text{where}\;d_{t}\left(H_{t},\theta_{t}\right)=\underset{a\in \{0,1\}}{\arg\max}\;Q_{t}\left(H_{t},a;\theta_{t}\right)=I\left(H_{t1}^{⊤}\gamma_{t}>0\right),t=1,2.$$

Theorems 2 and 3 of Section 5 demonstrate the consistency and asymptotic normality of the SSL estimators $\left\{{\hat{\theta }}_{t},t=1, 2\right\}$ for their respective population parameters $\left\{{\bar{\theta }}_{t},t=\right.1, 2\}$, even in the case where (1) is mis-specified. As we explain next, this in turn yields desirable statistical results for evaluating the derived policy ${\overset{‾}{d}}_{t}\equiv {\overset{‾}{d}}_{t}\left(H_{t}\right)\equiv d_{t}\left(H_{t},{\bar{\theta }}_{t}\right)={argmax}_{a\in \{0, 1\}}Q_{t}\left({\overset{ˇ}{H}}_{t},a;{\bar{\theta }}_{t}\right)$ for t=1, 2.

## 4. Semi-Supervised Off-Policy Policy Evaluation

To evaluate the performance of the optimal policy $\bar{𝒟}=\left\{{\overset{‾}{d}}_{t}\left(H_{t}\right),t=1,2\right\}$, derived under the *Q*-learning framework, one may estimate the expected population outcome under the policy $\bar{𝒟}$:

$$\bar{V}\equiv 𝔼\left[𝔼\left\{Y_{2}+𝔼\left\{Y_{3}|{\overset{ˇ}{H}}_{2},\;A_{2}={\bar{d}}_{2}\left(H_{2}\right)\right\}|H_{1},A_{1}={\bar{d}}_{1}\left(H_{1}\right)\right\}\right].$$

If models in (1) are correctly specified, then under standard causal assumptions (i)-(iii) (consistency, sequential ignorability, and positivity), an asymptotically consistent supervised estimator for the value function can be obtained as

$${\hat{V}}_{Q}={\mathbb{P}}_{n}\left[Q_{1}^{o}\left({\overset{ˇ}{H}}_{1};{\hat{\theta }}_{1}\right)\right],$$

where $Q_{t}^{o}\left({\overset{ˇ}{H}}_{t};\theta_{t}\right)\equiv Q_{t}\left({\overset{ˇ}{H}}_{t},d_{t}\left(H_{t};\theta_{t}\right);\theta_{t}\right)$. However, ${\hat{V}}_{Q}$ is likely to be biased when the outcome models in (1) are mis-specified. This occurs frequently in practice since $Q_{1}\left({\overset{ˇ}{H}}_{1},A_{1}\right)$ is especially difficult to specify correctly.

To improve the robustness to model mis-specification, we augment ${\hat{V}}_{Q}$ via propensity score weighting. This gives us an SSL doubly robust (${SSL}_{DR}$) estimator for $\overset{‾}{V}$. To this end, we define propensity scores:

$$\pi_{t}\left({\overset{ˇ}{H}}_{t}\right)=\mathbb{P}\left\{A_{t}=1|{\overset{ˇ}{H}}_{t}\right\},\;t=1,2.$$

To estimate $\left\{\pi_{t}(\cdot ),t=1, 2\right\}$, we impose the following generalized linear models (GLMs):

(4) $$\pi_{t}\left({\overset{ˇ}{H}}_{t};\xi_{t}\right)=\sigma \left({\overset{ˇ}{H}}_{t}^{⊤}\xi_{t}\right),\;\text{with}\;\sigma (x)\equiv 1/\left(1+e^{-x}\right)\;\text{for}\;t=1,2.$$

We use the logistic model with potentially non-linear basis functions $\overset{ˇ}{H}$ for simplicity of presentation, but one may choose other GLMs or alternative basis expansions to incorporate non-linear effects in the propensity score models. One can estimate $\xi ={\left(\xi_{1}^{⊤},\xi_{2}^{⊤}\right)}^{⊤}$ based on the standard maximum likelihood estimators using labeled data, denoted by $\hat{\xi }={\left({\hat{\xi }}_{1}^{⊤},{\hat{\xi }}_{2}^{⊤}\right)}^{⊤}$. We denote the limit of $\hat{\xi }$ as $\bar{\xi }={\left({\bar{\xi }}_{1}^{⊤},{\bar{\xi }}_{2}^{⊤}\right)}^{⊤}$. Note that this is not necessarily equal to the true model parameter under correct specification of (4), but corresponds to the population solution of the fitted models.

Our framework is flexible to allow an SSL approach to estimate the propensity scores. As these are nuisance parameters needed for estimation of the value function, and SSL for GLMs has been widely explored (See Chakrabortty, 2016, Ch. 2), we proceed with the usual GLM estimation to keep the discussion focused. However, SSL for propensity scores can be beneficial in certain cases, as we show in Proposition 9.

### 4.1 SUP_DR_ Value Function Estimation

To derive a supervised doubly robust (SUP_DR_) estimator for $\overset{‾}{V}$ overcoming confounding in the observed data, we let $\Theta ={\left(\theta^{⊤},\xi^{⊤}\right)}^{⊤}$ and define the inverse probability weights (IPW) using the propensity scores as

$$\omega_{1}\left({\overset{ˇ}{H}}_{1},A_{1},\Theta \right)\equiv \frac{d_{1}\left(H_{1};\theta_{1}\right)A_{1}}{\pi_{1}\left({\overset{ˇ}{H}}_{1};\xi_{1}\right)}+\frac{\left\{1-d_{1}\left(H_{1};\theta_{1}\right)\right\}\left\{1-A_{1}\right\}}{1-\pi_{1}\left({\overset{ˇ}{H}}_{1};\xi_{1}\right)},\;\text{and}\;\omega_{2}\left({\overset{ˇ}{H}}_{2},A_{2},\Theta \right)\equiv \omega_{1}\left({\overset{ˇ}{H}}_{1},A_{1},\Theta \right)\;\left(\frac{d_{2}\left(H_{2};\theta_{2}\right)A_{2}}{\pi_{2}\left({\overset{ˇ}{H}}_{2};\xi_{2}\right)}+\frac{\left\{1-d_{2}\left(H_{2};\theta_{2}\right)\right\}\left\{1-A_{2}\right\}}{1-\pi_{2}\left({\overset{ˇ}{H}}_{2};\xi_{2}\right)}\right).$$

Then we augment $Q_{1}^{o}\left(H_{1};{\hat{\theta }}_{1}\right)$ based on the estimated propensity scores via

$${𝒱}_{{\text{SUP}}_{\text{DR}}}(L;\hat{\Theta })=Q_{1}^{o}\left(H_{1};{\hat{\theta }}_{1}\right)\;+\omega_{1}\left({\overset{ˇ}{H}}_{1},A_{1},\hat{\Theta }\right)\;\left[Y_{2}-\left\{Q_{1}^{o}\left(H_{1},{\hat{\theta }}_{1}\right)-Q_{2}^{o}\left({\overset{ˇ}{H}}_{2};{\hat{\theta }}_{2}\right)\right\}\right]\;\;+\omega_{2}\left({\overset{ˇ}{H}}_{2},A_{2},\hat{\Theta }\right)\;\left\{Y_{3}-Q_{2}^{o}\left({\overset{ˇ}{H}}_{2};{\hat{\theta }}_{2}\right)\right\}$$

and estimate $\overset{‾}{V}$ as

(5) $${\hat{V}}_{{\text{SUP}}_{\text{DR}}}={\mathbb{P}}_{n}\;\left\{{𝒱}_{{\text{SUP}}_{\text{DR}}}(L;\hat{\Theta })\right\}.$$

**Remark 1** *Importance-sampling value function estimators employ similar augmentation strategies. See, for example, the estimators shown in (*10*) in* Jiang and Li (2016a) *and (*3*) in* Thomas and Brunskill (2016b). *However, these consider a fixed policy, and we account for the fact that we estimate the DTR with the same data set. The construction of augmentation in* ${\hat{V}}_{{SUP}_{DR}}$ *also differs from the usual augmented IPW estimators (*Chakraborty and Moodie, 2013). *As we are interested in the value had the population been treated with policy* $\bar{𝒟}$ *and not a fixed sequence* ($A_{1},A_{2}$), *we augment the weights for a fixed treatment (i.e.,* $A_{t}=1$ *for* t=1, 2*) with the propensity score weights for the estimated regime* $I\left(A_{t}={\overset{‾}{d}}_{t}\right),\;t=1, 2$. *Finally, we note that this estimator can easily be extended to incorporate non-binary treatments.*

The supervised value function estimator ${\hat{V}}_{{\text{SUP}}_{\text{DR}}}$ is doubly robust in the sense that if either the outcome, or the propensity score models are correctly specified, then ${\hat{V}}_{{\text{SUP}}_{\text{DR}}}\overset{\mathbb{P}}{\to }\overset{‾}{V}$ in probability. Moreover, under certain reasonable assumptions, ${\hat{V}}_{{\text{SUP}}_{\text{DR}}}$ is asymptotically normal. Theoretical guarantees and proofs for this procedure are shown in Appendix F.1.

### 4.2 SSL_DR_ Value Function Estimation

Analogous to semi-supervised *Q*-learning, we propose a procedure for adapting the augmented value function estimator to leverage 𝒰, by imputing suitable functions of the unobserved outcome in (5). Note that since ${\overset{ˇ}{H}}_{2}$ involves $Y_{2}$, both $\omega_{2}\left({\overset{ˇ}{H}}_{2},A_{2};\Theta \right)$ and $Q_{2}^{o}\left({\overset{ˇ}{H}}_{2};\theta_{2}\right)=Y_{2}\beta_{21}+Q_{2-}^{o}\left(H_{2};\theta_{2}\right)$ are not available in the unlabeled set, where $Q_{2-}^{o}\left(H_{2};\theta_{2}\right)=H_{20}^{⊤}\beta_{22}+{\left[H_{21}^{⊤}\gamma_{2}\right]}_{+}$. By writing ${𝒱}_{{\text{SUP}}_{\text{DR}}}(L;\hat{\Theta })$ as

$${𝒱}_{{\text{SUP}}_{\text{DR}}}(L;\hat{\Theta })=Q_{1}^{o}\left(H_{1};{\hat{\theta }}_{1}\right)+\omega_{1}\left({\overset{ˇ}{H}}_{1},A_{1},\hat{\Theta }\right)\;\left\{\left(1+{\hat{\beta }}_{21}\right)Y_{2}-Q_{1}^{o}\left(H_{1},{\hat{\theta }}_{1}\right)+Q_{2-}^{o}\left(H_{2};{\hat{\theta }}_{2}\right)\right\}\;\;+\omega_{2}\left({\overset{ˇ}{H}}_{2},A_{2},\hat{\Theta }\right)\left\{Y_{3}-{\hat{\beta }}_{21}Y_{2}-Q_{2-}^{o}\left(H_{2};{\hat{\theta }}_{2}\right)\right\},$$

we note that to impute ${𝒱}_{{\text{SUP}}_{\text{DR}}}(L;\hat{\Theta })$ for subjects in 𝒰, we need to impute $Y_{2},\omega_{2}\left({\overset{ˇ}{H}}_{2},A_{2};\hat{\Theta }\right)$, and $Y_{t}\omega_{2}\left({\overset{ˇ}{H}}_{2},A_{2};\hat{\Theta }\right)$ for t=2, 3. We define the conditional mean functions

$$\mu_{2}^{v}(\vec{U})\equiv 𝔼\left[Y_{2}|\vec{U}\right],\;\mu_{\omega_{2}}^{v}(\vec{U})\equiv 𝔼\left[\omega_{2}\left({\overset{ˇ}{H}}_{2},A_{2};\bar{\Theta }\right)|\vec{U}\right],\;\mu_{t\omega_{2}}^{v}(\vec{U})\equiv 𝔼\left[Y_{t}\omega_{2}\left({\overset{ˇ}{H}}_{2},A_{2};\bar{\Theta }\right)|\vec{U}\right],$$

for t=2, 3, where $\bar{\Theta }={\left({\bar{\theta }}^{⊤},{\bar{\xi }}^{⊤}\right)}^{⊤}$. As in Section 3.2 we approximate these expectations using a flexible imputation model followed by a refitting step for bias correction under possible mis-specification of the imputation models.

#### Step I: Imputation

We fit flexible weakly parametric or non-parametric models to the labeled data to approximate the functions $\left\{\mu_{2}^{v}(\vec{U}),\mu_{\omega_{2}}^{v}(\vec{U}),\mu_{t\omega_{2}}^{v}(\vec{U}),\;t=2, 3\right\}$ with unknown parameter Θ, estimated via the SSL *Q*-learning as in Section 3.2 and the propensity score modeling as discussed above. Denote the respective imputation models as $\left\{m_{2}(\vec{U}),m_{\omega_{2}}(\vec{U}),m_{t\omega_{2}}(\vec{U}),t=2, 3\right\}$ and their fitted values as $\left\{{\hat{m}}_{2}(\vec{U}),{\hat{m}}_{\omega_{2}}(\vec{U}),{\hat{m}}_{t\omega_{2}}(\vec{U}),t=2, 3\right\}$.

#### Step II: Refitting

To correct for potential biases arising from finite sample estimation and model mis-specifications, we perform refitting to obtain final imputed models for $\left\{Y_{2},\omega_{2}\left({\overset{ˇ}{H}}_{2},A_{2};\bar{\Theta }\right)\right.,\left.Y_{t}\omega_{2}\left({\overset{ˇ}{H}}_{2},A_{2};\overset{‾}{\Theta }\right),t=2, 3\right\}$ as $\left\{{\overset{‾}{\mu }}_{2}^{v}(\vec{U})=m_{2}(\vec{U})+\eta_{2}^{v},{\overset{‾}{\mu }}_{\omega_{2}}^{v}(\vec{U})=m_{\omega_{2}}(\vec{U})+\eta_{\omega_{2}}^{v},{\overset{‾}{\mu }}_{t\omega_{2}}^{v}(\vec{U})=m_{t\omega_{2}}(\vec{U})+\eta_{t\omega_{2}}^{v},t=2, 3\right\}$. As for the estimation of θ for *Q*-learning case, these refitted models are not required to be correctly specified but need to satisfy the following constraints:

$$\;𝔼\left[\omega_{1}\left({\overset{ˇ}{H}}_{1},A_{1};\bar{\Theta }\right)\;\left\{Y_{2}-{\bar{\mu }}_{2}^{v}(\vec{U})\right\}\right]=0,\;\;𝔼\;\left[Q_{2-}^{o}\left(\vec{U};\theta_{2}\right)\;\left\{\omega_{2}\left({\overset{ˇ}{H}}_{2},A_{2};\bar{\Theta }\right)-{\bar{\mu }}_{\omega_{2}}^{v}(\vec{U})\right\}\right]=0,\;\;\;𝔼\;\left[\omega_{2}\left({\overset{ˇ}{H}}_{2},A_{2};\bar{\Theta }\right)Y_{t}-{\bar{\mu }}_{t\omega_{2}}^{v}(\vec{U})\right]=0,\;t=2,3.$$

To estimate $\eta_{2}^{v}\;\eta_{\omega_{2}}^{v}$, and $\eta_{t\omega_{2}}^{v}$ under these constraints, we again employ cross-fitting and obtain ${\hat{\eta }}_{2}^{v}{\hat{\eta }}_{\omega_{2}}^{v}$, and ${\hat{\eta }}_{t\omega_{2}}^{v}$ as the solution to the following estimating equations

(6) $$\;\overset{K}{\underset{k=1}{\Sigma }}\underset{i\in {ℐ}_{k}}{\Sigma }\omega_{1}\left({\overset{ˇ}{H}}_{1i},A_{1i};\hat{\Theta }\right)\;\left\{Y_{2}-{\hat{m}}_{2}^{\left(-k\right)}\left({\vec{U}}_{i}\right)-{\hat{\eta }}_{2}^{v}\right\}=0,\;\;\overset{K}{\underset{k=1}{\Sigma }}\underset{i\in {ℐ}_{k}}{\Sigma }Q_{2-}^{o}\left({\vec{U}}_{i};{\hat{\theta }}_{2}\right)\;\left\{\omega_{2}\left({\overset{ˇ}{H}}_{2i},A_{2i};\hat{\Theta }\right)-{\hat{m}}_{\omega_{2}}^{\left(-k\right)}\left({\vec{U}}_{i}\right)-{\hat{\eta }}_{\omega_{2}}^{v}\right\}=0,\;\;\;\overset{K}{\underset{k=1}{\Sigma }}\underset{i\in {ℐ}_{k}}{\Sigma }\;\left\{\omega_{2}\left({\overset{ˇ}{H}}_{2i},A_{2i};\hat{\Theta }\right)Y_{ti}-{\hat{m}}_{t\omega_{2}}^{\left(-k\right)}\left({\vec{U}}_{i}\right)-{\hat{\eta }}_{t\omega_{2}}^{v}\right\}=0,\;t=2,3.$$

The resulting imputation functions for $Y_{2},\omega_{2}\left({\overset{ˇ}{H}}_{2},A_{2};\bar{\Theta }\right)$ and $Y_{t}\omega_{2}\left({\overset{ˇ}{H}}_{2},A_{2};\bar{\Theta }\right)$ are respectively constructed as ${\hat{\mu }}_{2}^{v}(\vec{U})=K^{-1}\sum_{k=1}^{K}{\hat{m}}_{2}^{\left(-k\right)}(\vec{U})+{\hat{\eta }}_{2}^{v},{\hat{\mu }}_{\omega_{2}}^{v}(\vec{U})=K^{-1}\sum_{k=1}^{K}{\hat{m}}_{\omega_{2}}(\vec{U})+{\hat{\eta }}_{\omega_{2}}^{v}$, and ${\hat{\mu }}_{t\omega_{2}}^{v}(\vec{U})=K^{-1}\sum_{k=1}^{K}{\hat{m}}_{t\omega_{2}}^{\left(-k\right)}(\vec{U})+{\hat{\eta }}_{t\omega_{2}}^{v}$, for t=2, 3.

#### Step III: Projection, Semi-Supervised Augmented Value Function Estimator

Finally, we proceed to estimate the value of the policy $\overset{‾}{V}$, using the following semi-supervised augmented estimator:

(7) $${\hat{V}}_{{\text{SSL}}_{\text{DR}}}={\mathbb{P}}_{N}\left\{{𝒱}_{{\text{SSL}}_{\text{DR}}}(\vec{U};\hat{\Theta },\hat{\mu })\right\},$$

where ${\hat{𝒱}}_{{\text{SSL}}_{\text{DR}}}(\vec{U})$ is the semi-supervised augmented estimator for observation $\vec{U}$ defined as:

$${𝒱}_{{\text{SSL}}_{\text{DR}}}(\vec{U};\hat{\Theta },\hat{\mu })=Q_{1}^{o}\left({\overset{ˇ}{H}}_{1};{\hat{\theta }}_{1}\right)+\omega_{1}\left({\overset{ˇ}{H}}_{1},A_{1},\hat{\Theta }\right)\;\left[\left(1+{\hat{\beta }}_{21}\right){\hat{\mu }}_{2}^{v}(\vec{U})-Q_{1}^{o}\left({\overset{ˇ}{H}}_{1};{\hat{\theta }}_{1}\right)+Q_{2-}^{o}\left(H_{2};{\hat{\theta }}_{2}\right)\right]\;+{\hat{\mu }}_{3\omega_{2}}(\vec{U})-{\hat{\beta }}_{21}{\hat{\mu }}_{2\omega_{2}}(\vec{U})-Q_{2-}^{o}\left(H_{2};{\hat{\theta }}_{2}\right){\hat{\mu }}_{\omega_{2}}(\vec{U}).$$

The above SSL estimator uses both labeled and unlabeled data along with outcome surrogates to estimate the value function, which yields a gain in efficiency as we show in Proposition 9. As its supervised counterpart, ${\hat{V}}_{SSL_{DR}}$ is doubly robust in the sense that if either the *Q* functions or the propensity scores are correctly specified, the value function will converge in probability to the true value $\overset{‾}{V}$. Additionally, it does not assume that the estimated treatment regime was derived from a different sample. These properties are summarized in Theorem 7 and Proposition 8 of the following section.

## 5. Theoretical Results

In this section we discuss our assumptions and theoretical results for the semi-supervised *Q*-learning and value function estimators. Throughout, we define the norm $\|g(x){\|}_{L_{2}(\mathbb{P})}\equiv \sqrt{\int \;g(x{)}^{2}d\mathbb{P}(x)}$ for any real valued function g(⋅). Additionally, let $\left\{U_{n}\right\}$, and $\left\{V_{n}\right\}$ be two sequences of random variables. We will use $U_{n}=O_{\mathbb{P}}\left(V_{n}\right)$ to denote stochastic boundedness of the sequence $\left\{U_{n}/V_{n}\right\}$, that is, for any $\epsilon >0,\exists M_{\epsilon },n_{\epsilon }\in \mathbb{R}$ such that $\mathbb{P}\left(\left|U_{n}/V_{n}\right|>M_{\epsilon }\right)<\epsilon \;\forall n>n_{\epsilon }$. We use $U_{n}=o_{\mathbb{P}}\left(V_{n}\right)$ to denote that $U_{n}/V_{n}\overset{\mathbb{P}}{\to }0$.

### 5.1 Theoretical Results for SSL *Q*-Learning

**Assumption 1** *(a) Sample size for* 𝒰, *and* ℒ, *are such that* n/N⟶0 *as* N,n⟶∞, *(b)* ${\overset{ˇ}{H}}_{t}\in {ℋ}_{t},{\overset{ˇ}{X}}_{t}\in {𝒳}_{t}$ *have finite second moments and compact support in* ${ℋ}_{t}\subset {\mathbb{R}}^{q_{t}},{𝒳}_{t}\subset {\mathbb{R}}^{p_{t}}t=1, 2$ *respectively (c)* $\Sigma_{1},\Sigma_{2}$ *are nonsingular*.

**Assumption 2** *Functions* $m_{s},s\in \{2, 3,22, 23\}$ *are such that (i)* ${sup}_{\vec{U}}\left|m_{s}(\vec{U})\right|<\infty$, *and (ii) the estimated functions* ${\overset{ˆ}{m}}_{s}$ *satisfy (ii)* ${sup}_{\vec{U}}\left|{\hat{m}}_{s}(\vec{U})-m_{s}(\vec{U})\right|=o_{\mathbb{P}}(1)$.

**Assumption 3** *Suppose* $\Theta_{1},\Theta_{2}$ *are open bounded sets, and* $p_{1},p_{2}$ *fixed under* (1). *We define the following class of functions:*

$${𝒬}_{t}\equiv \left\{Q_{t}\;:\;{𝒳}_{1}\mapsto \mathbb{R}|\theta_{1}\in {\text{Θ}}_{1}\subset {\mathbb{R}}^{p_{t}}\right\},\;t=1,2.$$

*Further suppose for* t=1, 2, *the solutions for* $𝔼\left[S_{t}^{\theta }\left(\theta_{t}\right)\right]=0$, *i.e.*, ${\bar{\theta }}_{1}$ *and* ${\bar{\theta }}_{2}$ *satisfy*

$$S_{2}^{\theta }\left(\theta_{2}\right)=\frac{\partial }{\partial \theta_{2}^{⊤}}{\left\|Y_{3}-Q_{2}\left({\overset{ˇ}{X}}_{2};\theta_{2}\right)\right\|}_{2}^{2},\;S_{1}^{\theta }\left(\theta_{1}\right)=\frac{\partial }{\partial \theta_{1}^{⊤}}{\left\|Y_{2}^{\text{*}}-Q_{1}\left({\overset{ˇ}{X}}_{1};\theta_{1}\right)\right\|}_{2}^{2}.$$

*The target parameters satisfy* ${\bar{\theta }}_{t}\in \Theta_{t},t=1, 2$. *We write* ${\bar{\beta }}_{t},{\bar{\gamma }}_{t}$ *as the components of* ${\bar{\theta }}_{t}$, *according to* (2).

Assumption 1 (a) distinguishes our setting from the standard missing data context. Theoretical results for the missing completely at random (MCAR) setting generally assume that the missingness probability is bounded away from zero (Tsiatis, 2006), which enables the use of standard semiparametric theory. However, in our setting one can intuitively consider the probability of observing an outcome being $\frac{n}{n+N}$ which converges to 0.

Assumption 2 usually follows when imputation functions are bounded– which is natural to expect from the boundedness of the covariates. This is the case for many practical settings, including clinical history variables. We also require uniform convergence of the estimated functions to their limit. This allows for the normal equations targeting the imputation residuals in (6) and Appendix (B) (for the T>2 case) to be well defined. Moreover, several off-the-shelf flexible imputation models for estimation can satisfy these conditions. See for example, local polynomial estimators, basis expansion regression like natural cubic splines or wavelets (Tsybakov, 2009). In particular, it is worth noting that we do not require any specific rate of convergence. As a result, the required condition is typically much easier to verify for many off-the-shelf algorithms. It is likely that other classes of models such as random forests can satisfy Assumption 2. Recent work suggests that it is plausible to use the existing point-wise convergence results to show uniform convergence. (see Scornet et al., 2015; Biau et al., 2008). Using some $L_{2}$ -type guarantee might be possible with extra care in the analysis, but that is out of the scope of the present paper.

Assumption 3 is fairly standard in the literature and ensures well-defined population level solutions for *Q*-learning regressions $\bar{\theta }$ exist, and belong to that parameter space. In this regard, we differentiate between population solutions $\bar{\theta }$ and true model parameters $\theta^{0}$ shown in equation (1). If the working models are mis-specified, Theorems 2 and 3 still guarantee that $\hat{\theta }$ is consistent and asymptotically normal centered at the population solution $\bar{\theta }$. However, when equation (1) is correct, $\hat{\theta }$ is asymptotically normal and consistent for the true parameter $\theta^{0}$. Now we are ready to state the theoretical properties of the semi-supervised *Q*-learning procedure described in Section 3.2.

**Theorem 2 (Distribution of** ${\hat{\theta }}_{2}$) *Under Assumptions 1-3*, ${\hat{\theta }}_{2}$ *satisfies*

$$\sqrt{n}\left({\hat{\theta }}_{2}-{\bar{\theta }}_{2}\right)=\sum_{2}^{-1}\frac{1}{\sqrt{n}}\sum_{i=1}^{n}\psi_{2}\left(L_{i};{\bar{\theta }}_{2}\right)+o_{\mathbb{P}}(1)\overset{d}{\to }𝒩\left(0,V_{2\text{SSL}}\left({\bar{\theta }}_{2}\right)\right),$$

*where* $\Sigma_{2}=𝔼\left[{\overset{ˇ}{X}}_{2}{\overset{ˇ}{X}}_{2}^{⊤}\right]$ *is defined in* Section 2, *the influence function* $\psi_{2}$ *is given by*

$$\psi_{2}\left(L;{\bar{\theta }}_{2}\right)=\left[\begin{array}{c} \left\{Y_{2}Y_{3}-{\overset{‾}{\mu }}_{23}(\vec{U})\right\}-{\overset{‾}{\beta }}_{21}\left\{Y_{2}^{2}-{\overset{‾}{\mu }}_{22}(\vec{U})\right\}-Q_{2-}\left(H_{2},A_{2};{\bar{\theta }}_{2}\right)\left\{Y_{2}-{\overset{‾}{\mu }}_{2}(\vec{U})\right\}\\ X_{2}\left\{Y_{3}-{\overset{‾}{\mu }}_{3}(\vec{U})\right\}-{\overset{‾}{\beta }}_{21}X_{2}\left\{Y_{2}-{\overset{‾}{\mu }}_{2}(\vec{U})\right\} \end{array}\right],$$

*and* $V_{2\text{SSL}}\left({\bar{\theta }}_{2}\right)=\Sigma_{2}^{-1}𝔼\left[\psi_{2}\left(L;{\bar{\theta }}_{2}\right)\psi_{2}{\left(L;{\bar{\theta }}_{2}\right)}^{⊤}\right]{\left(\Sigma_{2}^{-1}\right)}^{⊤}$.

We hold off remarks until the end of the results for the *Q*-learning parameters. Since the first stage regression depends on the second stage regression through a non-smooth maximum function, we make the following standard assumption (Laber et al., 2014) in order to provide valid statistical inference.

**Assumption 4** *Non-zero estimable population treatment effects* ${\overset{‾}{\gamma }}_{t},\;t=1, 2$ *: i.e., the population solution to* (2), *is such that (a)* $H_{21}^{⊤}{\overset{‾}{\gamma }}_{2}\neq 0$ *for all* $H_{21}\neq 0$, *and (b)* ${\overset{‾}{\gamma }}_{1}$ *is such that* $H_{11}^{⊤}{\overset{‾}{\gamma }}_{1}\neq 0$ *for all* $H_{11}\neq 0$ *almost everywhere*.

Assumption 4 yields regular estimators for the stage one regression and the value function, which depend on non-smooth components of the form $[x{]}_{+}$. This property is needed to achieve asymptotic normality of the *Q*-learning parameters for the first stage regression. Note that the estimating equation for the stage one regression in Section 3.2 includes ${\left[H_{21}^{⊤}{\hat{\gamma }}_{2}\right]}_{+}$. Thus, for the asymptotic normality of ${\hat{\theta }}_{1}$, we require $\sqrt{n}{\mathbb{P}}_{n}\left({\left[H_{21}^{⊤}{\hat{\gamma }}_{2}\right]}_{+}-{\left[H_{21}^{⊤}{\overset{‾}{\gamma }}_{2}\right]}_{+}\right)$ to be asymptotically normal.

We also note that this requirement is automatically guaranteed as long as $H_{11}$ contains at least one continuous covariate, as this implies $\mathbb{P}\left(H_{21}^{⊤}{\overset{‾}{\gamma }}_{2}=0\right)=0$ (analogous for ${\overset{‾}{\gamma }}_{1}$). Several feature covariates in the DTR context are continuous, e.g., blood pressure, blood sugar level, blood cholesterol levels, body temperature, weight, etc. Violation of Assumption 4 will yield asymptotically biased and non-regular *Q*-learning estimates, which translate into poor coverage of the confidence intervals (see Laber et al. (2014) for a thorough discussion on this topic). This is why Assumption 4 is fairly standard in *Q*-learning, A-learning, etc. (Chakraborty and Moodie, 2013; Schulte et al., 2014; Laber et al., 2014; Robins, 2004; Tsiatis et al., 2019).

**Theorem 3 (Distribution of** ${\overset{ˆ}{\theta }}_{1}$) *Under Assumptions 1-3, and 4 (a)*, ${\hat{\theta }}_{1}$ *satisfies*

$$\sqrt{n}\left({\hat{\theta }}_{1}-{\bar{\theta }}_{1}\right)=\sum_{1}^{-1}\frac{1}{\sqrt{n}}\sum_{i=1}^{n}\psi_{1}\left(L_{i};{\bar{\theta }}_{1}\right)+o_{\mathbb{P}}(1)\overset{d}{\to }𝒩\left(0,V_{1\text{SSL}}\left({\bar{\theta }}_{1}\right)\right)$$

*where* $\Sigma_{1}^{-1}=𝔼\left[{\overset{ˇ}{X}}_{1}{\overset{ˇ}{X}}_{1}^{⊤}\right]$, *the influence function* $\psi_{1}$ *is given by*

$$\psi_{1}\left(L;{\bar{\theta }}_{1}\right)=X_{1}\left(1+{\bar{\beta }}_{21}\right)\left\{Y_{2}-{\bar{\mu }}_{2}(\vec{U})\right\}+𝔼\left[X_{1}\left(Y_{2},H_{20}^{⊤}\right)\right]\psi_{\beta_{2}}\left(L;{\bar{\theta }}_{2}\right)\;+𝔼\left[X_{1}H_{21}^{⊤}|H_{21}^{⊤}{\bar{\gamma }}_{2}>0\right]\mathbb{P}\left(H_{21}^{⊤}{\bar{\gamma }}_{2}>0\right)\psi_{\gamma_{2}}\left(L;{\bar{\theta }}_{2}\right),$$

$V_{\mathrm{1SSL}}\left({\bar{\theta }}_{1}\right)=\Sigma_{1}^{-1}𝔼\left[\psi_{1}\left(L;{\bar{\theta }}_{1}\right)\psi_{1}{\left(L;{\bar{\theta }}_{1}\right)}^{⊤}\right]{\left(\Sigma_{1}^{-1}\right)}^{⊤}$, *and* $\psi_{\beta_{2}},\psi_{\gamma_{2}}$ *are the elements corresponding to* ${\bar{\beta }}_{2},{\bar{\gamma }}_{2}$ *of the influence function* $\psi_{2}$ *defined in Theorem 2.*

**Remark 4** *1) Theorems 2 and 3 establish the* $\sqrt{n}$*-consistency and asymptotic normality of* ${\hat{\theta }}_{1},{\hat{\theta }}_{2}$ *for any* K≥2. *Beyond asymptotic normality at* $\sqrt{n}$ *scale, these theorems also provide an asymptotic linear expansion of the estimators with influence functions* $\psi_{1}$ *and* $\psi_{2}$ *respectively. For extension and discussion of the method for* T>2 *stages see* Appendix B.

*2)* $V_{\mathrm{1SSL}}(\bar{\theta }),V_{\mathrm{2SSL}}(\bar{\theta })$ *reflect an efficiency gain over the fully supervised approach due to sample* 𝒰 *and the surrogates contribution to prediction performance. This gain is formalized in Proposition 5 which quantifies how correlation between surrogates and outcome increases efficiency.*

*3) Let* $\psi ={\left[\psi_{1}^{⊤},\psi_{2}^{⊤}\right]}^{⊤}$, *we collect the vector of estimated Q-learning parameters* $\theta ={\left(\theta_{1}^{⊤},\theta_{2}^{⊤}\right)}^{⊤}$, *then under Assumptions 1-3, 4 (a), we have*

$$\sqrt{n}(\hat{\theta }-\bar{\theta })=\sum^{-1}\frac{1}{\sqrt{n}}\sum_{i=1}^{n}\psi \left(L_{i};\bar{\theta }\right)+o_{\mathbb{P}}(1)\overset{d}{\to }𝒩\left(0,V_{\text{SSL}}\;(\bar{\theta })\right)$$

*with* $V_{\text{SSL}}(\bar{\theta })=\Sigma^{-1}𝔼\left[\psi (L;\bar{\theta })\psi (L;\bar{\theta }{)}^{⊤}\right]{\left(\Sigma^{-1}\right)}^{⊤}$.

*4) Theorems 2 and 3 hold even when the Q functions are mis-specified, that is*, ${\hat{\theta }}_{1},{\hat{\theta }}_{2}$ *are consistent and asymptotically normal for* ${\bar{\theta }}_{1},{\bar{\theta }}_{2}$. *Furthermore, if model* (1) *is correctly specified then we can simply replace* $\bar{\theta }$ *with* $\theta^{0}$ *in the above result*.

*5) We estimate* $V_{\text{SSL}}(\bar{\theta })$ *via sample-splitting as*

$${\hat{V}}_{\text{SSL}}(\hat{\theta })={\hat{\Sigma }}^{-1}\hat{A}(\theta )\;{\left({\hat{\Sigma }}^{-1}\right)}^{⊤},\;\mathrm{where}$$

$$\hat{A}(\hat{\theta })=n^{-1}\sum_{k=1}^{K}\sum_{i\in {ℐ}_{k}}\psi^{\left(-k\right)}\;\left(L_{i};\hat{\theta }\right)\;\psi^{\left(-k\right)}\;{\left(L_{i};\hat{\theta }\right)}^{⊤},$$

$${\hat{\sum }}_{t}={\mathbb{P}}_{n}\left\{X_{t}X_{t}^{⊤}\right\},\;t=1,2.$$

Note that we can decompose ψ into the influence function for each set of parameters, for example, we have $\psi_{2}={\left(\psi_{\beta_{2}}^{⊤},\psi_{\gamma_{2}}^{⊤}\right)}^{⊤}$ where

$$\psi_{\gamma_{2}}\left(L;{\bar{\theta }}_{2}\right)=H_{21}A_{2}\;\left[\left\{Y_{3}-{\bar{\mu }}_{3}(\vec{U})\right\}-{\bar{\beta }}_{21}\left\{Y_{2}-{\bar{\mu }}_{2}(\vec{U})\right\}\right].$$

Thus, we can decompose the variance-covariance matrix into a component for each parameter, the variance-covariance for the treatment effect for stage 2 regression $\gamma_{2}$ is

$$𝔼\left[\psi_{\gamma_{2}}\left(L;{\bar{\theta }}_{2}\right)\psi_{\gamma_{2}}{\left(L;{\bar{\theta }}_{2}\right)}^{⊤}\right]=𝔼\;\left[H_{21}H_{21}^{⊤}A_{2}^{2}{\left\{Y_{3}-{\bar{\mu }}_{3}(\vec{U})-\beta_{21}\;\left(Y_{2}-{\bar{\mu }}_{2}(\vec{U})\right)\right\}}^{2}\right].$$

This gives us some insight into how the predictive power of $\vec{U}$, which contains surrogates $W_{1},W_{2}$, decreases uncertainty of parameter estimates, yielding smaller standard errors. This is the case in general for the influence functions of estimators for ${\bar{\theta }}_{1},{\bar{\theta }}_{2}$. We formalize this result with the following proposition. Let ${\hat{\theta }}_{\text{SUP}}$ be the estimator for the fully supervised *Q*-learning procedure (i.e. only using labeled data), with influence function and asymptotic variance denoted as $\psi_{\text{SUP}}$ and $V_{\text{SUP}}$ respectively (see Appendix D.1 for the derivation and exact form of $\psi_{\text{SUP}}$ and $V_{\mathrm{SUP}}$).

For the following proposition we need the imputation models ${\overset{‾}{\mu }}_{s},\;s\in \{2, 3,22, 23\}$ to satisfy additional constraints of the form $𝔼\;\left[X_{2}X_{2}^{⊤}\left\{Y_{2}Y_{3}-{\overset{‾}{\mu }}_{23}(\vec{U})\right\}\right]=0$. We list them in Assumption 7, Appendix D.1. One can construct estimators which satisfy such conditions by simply augmenting $\eta_{2},\eta_{22},\eta_{3},\eta_{23}$ in (3) with additional terms in the refitting step.

**Proposition 5** *Under Assumptions 1-3, 4 (a), and 7 then*

$$V_{\text{SSL}}(\bar{\theta })=V_{\text{SUP}}(\bar{\theta })-\Sigma^{-1}\mathrm{Var}\;\left[\psi_{\text{SUP}}(L;\bar{\theta })-\psi_{\text{SSL}}(L;\bar{\theta })\right]\;{\left(\Sigma^{-1}\right)}^{⊤}.$$

**Remark 6** *Proposition 5 illustrates how the estimates for the semi-supervised Q-learning parameters are at least as efficient, if not more so, than the supervised ones. Intuitively, the difference in efficiency is explained by how much information is gained by incorporating the surrogates* $W_{1},W_{2}$ *into the estimation procedure. If there is no new information in the surrogate variables, then residuals found in* $\psi_{\mathrm{SSL}}(L;\theta )$ *will be of similar magnitude to those in* $\psi_{\text{SUP}}(L;\theta )$, *and thus the difference in efficiency will be small:* $\mathrm{Var}\left[\psi_{\text{SUP}}(L;\bar{\theta })-\psi_{\text{SSL}}(L;\bar{\theta })\right]\approx 0$. *In this case both methods will yield equally efficient parameters. The gain in precision is especially relevant for the treatment interaction coefficients* $\gamma_{1},\gamma_{2}$ *used to learn the dynamic treatment rules. Finally, note that for Proposition 5, we do not need the correct specification of Q functions or imputation models*.

### 5.2 Theoretical Results for SSL Estimation of the Value Function

If model (1) is correct, one only needs to add Assumption 4 (b) for ${\mathbb{P}}_{N}\left\{Q_{1}^{o}\left(H_{1};{\hat{\theta }}_{1}\right)\right\}$ to be a consistent estimator of the value function $\overset{‾}{V}$ (Zhu et al., 2019). However, as we discussed earlier, (1) is likely mis-specified. This motivates the use of our doubly robust semi-supervised value function estimator. We also show our estimator is asymptotically normal and achieves efficiency equal to or exceeding that of the corresponding supervised estimator. To that end, define the following class of functions:

$${𝒲}_{t}\equiv \left\{\pi_{t}\;:\;{ℋ}_{t}\mapsto \mathbb{R}|\xi_{t}\in {\text{Ω}}_{t}\right\},\;t=1,2,$$

under propensity score models $\pi_{1},\pi_{2}$ in (4).

**Assumption 5** *Let the score population equations* $𝔼\;\left[S_{t}^{\xi }\left({\overset{ˇ}{H}}_{t};\xi_{t}\right)\right]=0,t=1, 2$ *have solutions* ${\bar{\xi }}_{1},{\bar{\xi }}_{2}$, *where*

$$S_{t}^{\xi }\left({\overset{ˇ}{H}}_{t};\xi_{t}\right)=\frac{\partial }{\partial \xi_{t}}\text{log}\;\left[\pi_{t}{\left({\overset{ˇ}{H}}_{t};\xi_{t}\right)}^{A_{t}}{\left\{1-\pi_{t}\left({\overset{ˇ}{H}}_{t};\xi_{t}\right)\right\}}^{\left(1-A_{t}\right)}\right],\;t=1,2,$$

- $\Omega_{1},\Omega_{2}$ *are open, bounded sets and the population solutions satisfy* ${\bar{\xi }}_{t}\in \Omega_{t},t=1, 2$,
- *for* ${\bar{\xi }}_{t},\;t=1, 2$, $\underset{{\overset{ˇ}{H}}_{t}\in {ℋ}_{1}}{inf}\pi_{1}\left({\overset{ˇ}{H}}_{t};{\bar{\xi }}_{t}\right)>0$,
- *Finite second moment:* $𝔼\;\left[S_{t}^{\xi }{\left({\overset{ˇ}{H}}_{t};\Theta_{t}\right)}^{2}\right]\leq \infty$, *and Fisher information matrix* $𝔼\;\left[\frac{\partial }{\partial \xi_{t}}S_{t}^{\xi }\left({\overset{ˇ}{H}}_{t};\Theta_{t}\right)\right]$ *exists and is non singular,*
- *Second-order partial derivatives of* $S_{t}^{\xi }\left({\overset{ˇ}{H}}_{t};\Theta_{t}\right)$ *with respect to* ξ *exist and for every* ${\overset{ˇ}{H}}_{t}$, *and satisfy* $\left|\partial^{2}S_{t}^{\xi }\left({\overset{ˇ}{H}}_{t};\Theta_{t}\right)/\partial \xi_{i}\partial \xi_{j}\right|\leq {\tilde{S}}_{t}\left({\overset{ˇ}{H}}_{t}\right)$ *for some integrable measurable function* ${\tilde{S}}_{t}$ *in a neighborhood of* $\bar{\xi }$.

**Assumption 6** *Functions* $m_{2},m_{\omega_{2}},m_{t\omega_{2}}\;t=2, 3$ *are such that (i)* $\underset{\vec{U}}{sup}\;\left|m_{s}(\vec{U})\right|<\infty$, *and (ii) the estimated functions* ${\overset{ˆ}{m}}_{s}$ *satisfy (ii)* $\underset{\vec{U}}{sup}\;\left|{\hat{m}}_{s}(\vec{U})-m_{s}(\vec{U})\right|=o_{\mathbb{P}}(1)$, $s\in \left\{2,\omega_{2},2\omega_{2},3\omega_{2}\right\}$.

Assumption 5 is standard for Z-estimators (see Vaart, 1998, Ch. 5.6). Finally, we use $\psi^{\xi }$ and $\psi^{\theta }$ to denote the influence function for $\hat{\xi }$, and $\hat{\theta }$ respectively. We are now ready to state our theoretical results for the value function estimator in equation (7). The proof, and the exact form of $\psi^{\xi }$ can be found in Appendix D.2.

**Theorem 7 (Asymptotic Normality for** ${\hat{V}}_{{SSL}_{DR}}$) *Under Assumptions 1-6*, ${\hat{V}}_{{SSL}_{DR}}$ *defined in* (7) *satisfies*

$$\sqrt{n}\left\{{\hat{V}}_{{\text{SSL}}_{\text{DR}}}-{𝔼}_{𝕊}\;\left[{𝒱}_{{\text{SSL}}_{\text{DR}}}(L;\bar{\Theta },\bar{\mu })\right]\right\}=\frac{1}{\sqrt{n}}\sum_{i=1}^{n}\psi_{{\text{SSL}}_{\text{DR}}}^{v}\left(L_{i};\bar{\Theta }\right)+o_{\mathbb{P}}(1),$$

where

$$\frac{1}{\sqrt{n}}\sum_{i=1}^{n}\psi_{{\text{SSL}}_{\text{DR}}}^{v}\left(L_{i};\bar{\Theta }\right)\overset{d}{\to }𝒩\;\left(0,\sigma_{{\text{SSL}}_{\text{DR}}}^{2}\right).$$

Here

$$\psi_{{\text{SSL}}_{\text{DR}}}^{v}(L;\bar{\Theta })=\nu_{{\text{SSL}}_{\text{DR}}}(L;\bar{\Theta })+{\psi^{\theta }{\left(L\right)}^{⊤}\frac{\partial }{\partial \theta }\int \;{𝒱}_{{\text{SUP}}_{\text{DR}}}(L;\Theta )d{\mathbb{P}}_{L}|}_{\Theta =\bar{\Theta }}\;+{\psi^{\xi }{\left(L\right)}^{⊤}\frac{\partial }{\partial \xi }\int \;{𝒱}_{{\text{SUP}}_{\text{DR}}}(L;\Theta )d{\mathbb{P}}_{L}|}_{\Theta =\bar{\Theta }},$$

$$\nu_{{\text{SSL}}_{\text{DR}}}(L;\bar{\Theta })=\omega_{1}\left({\overset{ˇ}{H}}_{1},A_{1};{\bar{\Theta }}_{1}\right)\left(1+{\bar{\beta }}_{21}\right)\;\left\{Y_{2}-{\bar{\mu }}_{2}^{v}(\vec{U})\right\}+\omega_{2}\left({\overset{ˇ}{H}}_{2},A_{2},{\bar{\Theta }}_{2}\right)Y_{3}-{\bar{\mu }}_{3\omega_{2}}(\vec{U})\;-{\bar{\beta }}_{21}\;\left\{\omega_{2}\left({\overset{ˇ}{H}}_{2},A_{2},{\bar{\Theta }}_{2}\right)Y_{2}-{\bar{\mu }}_{2\omega_{2}}(\vec{U})\right\}\;-Q_{2-}^{o}\left(H_{2};{\bar{\theta }}_{2}\right)\;\left\{\omega_{2}\left({\overset{ˇ}{H}}_{2},A_{2},{\bar{\Theta }}_{2}\right)-{\bar{\mu }}_{\omega_{2}}(\vec{U})\right\},$$

$\sigma_{{SSL}_{DR}}^{2}=𝔼\;\left[\psi_{{SSL}_{DR}}^{v}(L;\bar{\Theta }{)}^{2}\right]$, *and* ${𝒱}_{{SUP}_{DR}}(L;\Theta )$ *is as defined in* (5).

**Proposition 8 (Double robustness of** ${\hat{V}}_{{SSL}_{DR}}$ **as an estimator of** $\overset{‾}{V}$) *(a) If either* ${\left\|Q_{t}\left({\overset{ˇ}{H}}_{t},A_{t};{\hat{\theta }}_{t}\right)-Q_{t}\left({\overset{ˇ}{H}}_{t},A_{t}\right)\right\|}_{L_{2}(\mathbb{P})}\to 0$, *or* ${\left\|\pi_{t}\left({\overset{ˇ}{H}}_{t};{\hat{\xi }}_{t}\right)-\pi_{t}\left({\overset{ˇ}{H}}_{t}\right)\right\|}_{L_{2}(\mathbb{P})}\to 0$ *for* t=1, 2, *then under Assumptions 1-6*, ${\hat{V}}_{{SSL}_{DR}}$ *satisfies*

$${\hat{V}}_{{\text{SSL}}_{\text{DR}}}\overset{\mathbb{P}}{\to }\bar{V}.$$

*(b) If* ${\left\|Q_{t}\left({\overset{ˇ}{H}}_{t},A_{t};{\hat{\theta }}_{t}\right)-Q_{t}\left({\overset{ˇ}{H}}_{t},A_{t}\right)\right\|}_{L_{2}(\mathbb{P})}{\left\|\pi_{t}\left({\overset{ˇ}{H}}_{t};{\hat{\xi }}_{t}\right)-\pi_{t}\left({\overset{ˇ}{H}}_{t}\right)\right\|}_{L_{2}(\mathbb{P})}=o_{\mathbb{P}}\left(n^{-\frac{1}{2}}\right)$ *for* =1, 2, *then under Assumptions 1-6*, ${\hat{V}}_{{SSL}_{DR}}$ *satisfies*

$$\sqrt{n}\left({\hat{V}}_{{\text{SSL}}_{\text{DR}}}-\bar{V}\right)\overset{d}{\to }𝒩\left(0,\sigma_{{\text{SSL}}_{\text{DR}}}^{2}\right).$$

Note that our positivity assumptions guarantee overlap of the propensity scores. In particular, the positivity assumption (iii) in Section 2 guarantees that $\pi_{t}\left(H_{t}\right),\;t=1, 2$ are bounded away from zero almost everywhere; this translates into having distribution overlap between both treatments. As for the models, if they are correctly specified, then from assumption (iii), the logistic estimators $\hat{\pi }$ can not be too small. If, in the other case, the propensity score models are mis-specified, Assumption 5 (ii) guarantees that the target parameters of $\hat{\pi }$’s are bounded away from zero, which translates into the limits of $\hat{\pi }$ ’s being bounded as well.

Next we define the supervised influence function of estimator ${\hat{V}}_{{SUP}_{DR}}$ (see Theorem 19 and corresponding proof in Appendix F.1). Let $\psi_{\text{sup}}^{\theta }$, be the influence function of the supervised estimator ${\hat{\theta }}_{\text{SUP}}$ for model (1). The influence function, and variance of the Value Function ${\hat{V}}_{\text{DR}}$ defined in (5) are

$$\psi_{{\text{SUP}}_{\text{DR}}}^{v}(L;\bar{\Theta })={𝒱}_{{\text{SUP}}_{\text{DR}}}(L;\bar{\Theta })-{𝔼}_{𝕊}\;\left[{𝒱}_{{\text{SUP}}_{\text{DR}}}(L;\bar{\Theta })\right]\;+{\psi_{\text{SUP}}^{\theta }{\left(L\right)}^{⊤}\frac{\partial }{\partial \theta }\int \;{𝒱}_{{\text{SUP}}_{\text{DR}}}(L;\Theta )d{\mathbb{P}}_{L}|}_{\Theta =\bar{\Theta }}+{\psi^{\xi }{\left(L\right)}^{⊤}\frac{\partial }{\partial \xi }\int \;{𝒱}_{{\text{SUP}}_{\text{DR}}}(L;\Theta )d{\mathbb{P}}_{L}|}_{\Theta =\bar{\Theta }},\;\sigma_{{\text{SUP}}_{\text{DR}}}^{2}=𝔼\left[\psi_{{\text{SUP}}_{\text{DR}}}^{v}{\left(L;\bar{\Theta }\right)}^{2}\right].$$

The flexibility of our SSL value function estimator $V_{{SSL}_{DR}}$ allows the use of either supervised or SSL approach for estimation of propensity score nuisance parameters ξ. For SSL estimation, we can use an approach similar to Section 3.2, (see Chakrabortty et al., 2018, Ch. 2 for details). This allows us to quantify the efficiency gain of $V_{{SSL}_{DR}}$ vs. $V_{{SUP}_{DR}}$ by comparing the asymptotic variances. In light of this, we assume SSL is used for ξ when estimating $V_{{SSL}_{DR}}$.

Before stating the result we discuss an additional requirement for the imputation models. As for Proposition 5, models ${\overset{‾}{\mu }}_{2}^{v}(\vec{U}),{\overset{‾}{\mu }}_{\omega_{2}}^{v}(\vec{U}),{\overset{‾}{\mu }}_{t\omega_{2}}^{v}(\vec{U}),t=2,3$ need to satisfy a few additional constraints of the form

$$𝔼\;\left[\omega_{1}\left({\overset{ˇ}{H}}_{1},A_{1};{\bar{\Theta }}_{1}\right)Q_{2-}^{o}\left(H_{2};{\bar{\theta }}_{1}\right)\left\{Y_{2}-{\bar{\mu }}_{2}^{v}(\vec{U})\right\}\right]=0.$$

As there are several constraints, we list them in Appendix D.2, and condense them in Assumption 8, Appendix D.2. Again, one can construct estimators which satisfy such conditions by simply augmenting $\eta_{2}^{v},\eta_{\omega_{2}}^{v},\eta_{t\omega_{2}}^{v},\;t=2, 3$ in (6) with additional terms in the refitting step.

**Proposition 9** *Under Assumptions 1-6, and 8, asymptotic variances* $\sigma_{{SSL}_{DR}}^{2},\sigma_{{SUP}_{DR}}^{2}$ *satisfy*

$$\sigma_{{\text{SSL}}_{\text{DR}}}^{2}=\sigma_{{\text{SUP}}_{\text{DR}}}^{2}-\mathrm{Var}\;\left[\psi_{{\text{SUP}}_{\text{DR}}}^{v}(L;\bar{\Theta })-\psi_{{\text{SSL}}_{\text{DR}}}^{v}(L;\bar{\Theta })\right].$$

**Remark 10** *1) Proposition 8 illustrates how* ${\hat{V}}_{{SSL}_{DR}}$ *is asymptotically unbiased if either the Q functions or the propensity scores are correctly specified.*

*2) An immediate consequence of Proposition 9 is that the semi-supervised estimator is at least as efficient (or more) than its supervised counterpart, that is* $\mathrm{Var}\;\left[\psi_{{SSL}_{DR}}(L;\Theta )\right]\leq \mathrm{Var}\;\left[\psi_{{SUP}_{DR}}(L;\Theta )\right]$. *As with Proposition 5, the difference in efficiency is explained by the information gain from incorporating surrogates.*

*3) To estimate standard errors for* $V_{{SSL}_{DR}}(\vec{U};\bar{\Theta })$, *we will approximate the derivatives of the expectation terms* $\frac{\partial }{\partial \Theta }\int \;{𝒱}_{{SUP}_{DR}}(L;\bar{\Theta })d{\mathbb{P}}_{L}$ *using kernel smoothing to replace the indicator functions. In particular, let* ${𝕂}_{h}(x)=\frac{1}{h}\sigma (x/h),\;\sigma$ *defined as in* (4), *we approximate* $d_{t}\left(H_{t},\theta_{2}\right)=I\left(H_{t1}^{⊤}\gamma_{t}>0\right)$ *with* ${𝕂}_{h}\left(H_{t1}^{⊤}\gamma_{t}\right)\;t=1, 2$, *and define the smoothed propensity score weights as*

$${\tilde{\omega }}_{1}\left({\overset{ˇ}{H}}_{1},A_{1},\Theta \right)\equiv \frac{A_{1}{𝕂}_{h}\left(H_{11}^{⊤}\gamma_{1}\right)}{\pi_{1}\left({\overset{ˇ}{H}}_{1};\xi_{1}\right)}+\frac{\left\{1-A_{1}\right\}\;\left\{1-{𝕂}_{h}\left(H_{11}^{⊤}\gamma_{1}\right)\right\}}{1-\pi_{1}\left({\overset{ˇ}{H}}_{1};\xi_{1}\right)},\;\mathrm{and}\;{\tilde{\omega }}_{2}\left({\overset{ˇ}{H}}_{2},A_{2},\Theta \right)\equiv {\tilde{\omega }}_{1}\left({\overset{ˇ}{H}}_{1},A_{1},\Theta \right)\;\left[\frac{A_{2}{𝕂}_{h}\left(H_{21}^{⊤}\gamma_{2}\right)}{\pi_{2}\left({\overset{ˇ}{H}}_{2};\xi_{2}\right)}+\frac{\left\{1-A_{2}\right\}\;\left\{1-{𝕂}_{h}\left(H_{21}^{⊤}\gamma_{2}\right)\right\}}{1-\pi_{2}\left({\overset{ˇ}{H}}_{2};\xi_{2}\right)}\right].$$

*We simply replace the propensity score functions with these smooth versions in* $\psi_{{SSL}_{DR}}^{v}(L;\bar{\Theta })$, *detail is given in* Appendix D.2.1. *To estimate the variance we use a sample-split estimator:*

$${\hat{\sigma }}_{{\text{SSL}}_{\text{DR}}}^{2}=n^{-1}\sum_{k=1}^{K}\sum_{i\in {ℐ}_{k}}\psi_{{\text{SSL}}_{\text{DR}}}^{v(-k)}{\left({\vec{U}}_{i};\hat{\Theta }\right)}^{2}.$$

### 5.3 Related Literature

There is a significant amount of work that uses surrogate variables for estimating treatment effects. Applications range from economics and education to healthcare. Prentice (1989); Begg and Leung (2000) propose to use surrogate endpoints for randomized trials. These surrogates are proxies of the missing outcomes and usually require the outcomes to be independent of treatment when conditioned on the surrogate. While helpful in establishing a theoretical framework, this assumption is usually hard to validate in practice. For example, unmeasured confounding between a long-term outcome and the chosen surrogate would invalidate this assumption. Athey et al. (2019) provide a solution in this problem space by combining several short-term outcomes into a surrogate index, their work provides an introduction to using surrogates for treatment effect estimation and further development of the surrogate index. This surrogate index assimilates to our approach because it is a good proxy of the outcome. However, the short-term outcomes used in the index are not only proxies but outcomes relevant as direct effects of the intervention. In our case, the surrogates are only correlated with the outcome of interest through the joint probability law ${\mathbb{P}}_{Y,O,A,W}$ but are not relevant to the conditional law of interest ${\mathbb{P}}_{Y|O,A}$ used to find the optimal policy.

In line with our approach, Pepe (1992) proposes using labeled and unlabeled data to estimate regression parameters, a valuable framework for average treatment effect estimation. More recently, Kallus and Mao (2020) proposed SSL methods for estimating an average causal treatment effect and using semi-parametric efficiency to show efficiency bounds on the ATE. However, both methods focus on a single time step. A key difference is that we are interested in the conditional treatment effect, which is needed to learn an optimal policy function. Also, the approach of Kallus and Mao (2020) assumes a missing at random setting, whereas we have a missing completely at random setting by design, as we first sample health records randomly and then label them. Alternatively, Chapter 4 of Van der Laan and Rose (2018) gives an overview of DTR using targeted learning theory. Both frameworks differ from our approach as we require that the probability of missingness goes to one as both labeled and unlabeled samples increase in size.

Davidian et al. (2005) use a semi-parametric approach to propose a consistent estimator for the average treatment effect under missing follow-up data. Chakrabortty et al. (2022) provide a survey of semi-supervised causal inference for estimating average and quantile treatment effects. To the best of our knowledge, this is the first work to propose estimating the time-dependent conditional treatment effect for policy learning in the missing outcome space. As previously discussed, Finn et al. (2016) proposed a semi-supervised RL method that achieves good empirical results and outperforms simple approaches such as direct imputation of the reward. However, the method does not leverage surrogates; there are no theoretical guarantees, and the approach lacks causal validity.

As to doubly robust estimation of DTRs, Dudík et al. (2011); Thomas and Brunskill (2016a); Kallus and Uehara (2020b) consider doubly robust policy evaluation; see also Murphy et al. (2001). A key difference between these approaches and our work is that the policy they evaluate is a pre-existing fixed function.

Another line of research focuses on doubly robust policy learning. They optimize a doubly robust value function estimator over a rich class of functions to estimate the optimal policy. To the best of our knowledge, existing work in this direction either takes T=1 (Athey and Wager, 2021; Zhou et al., 2022a; Zhao et al., 2019; Bennett and Kallus, 2020), or does not provide theoretical guarantees (Zhang et al., 2013; Sonabend-W et al., 2020a). The associated optimization is generally complicated unless the search space for the policies is sufficiently small (Zhou et al., 2022b). Their estimate is doubly robust in the sense that if either the *Q* function or the propensity scores are correct, their value estimate is consistent for the optimal value function. See also Tsiatis et al. (2019) Ch. 2 for a discussion of doubly robust estimation of causal effects. Our work differs from these types of approaches because we use the *Q* functions to estimate the optimal policy and then estimate the value function of *such* policy using a doubly robust estimator. Therefore, our target policy is not the underlying optimal policy but the best one attainable by linear approximations of our *Q* functions. This target policy only matches the optimal treatment policy if the used models are correctly specified. This characteristic is expected because ours is a purely model-based approach not depending on complicated optimization, likely necessary for doubly robust estimation of the true value function.

## 6. Simulations and Application to Electronic Health Record Data:

We perform extensive simulations to evaluate the finite sample performance of our methods for T=2, 3,5, 7. Additionally we apply our methods to an EHR study of treatment response for patients with inflammatory bowel disease to identify the optimal treatment sequence for each patient. These data have treatment response outcomes available for a small subset of patients only.

### 6.1 Simulation Results

We compare our SSL *Q*-learning methods to fully supervised *Q*-learning using labeled data sets of different sizes and settings. We focus on the efficiency gains of our approach. First we discuss our simulation settings, then go on to show results for the *Q* function parameters under correct and incorrect working models for (1). We then show value function summary statistics under correct models, mis-specification for the *Q* models in (1), and the propensity score function $\pi_{2}$ in (4). Finally we show the correct and mis-specified results for a general T>2 time horizon setting (see Appendix B for the extension of the methods to a general time horizon).

Following a similar set-up as in Schulte et al. (2014), we first consider a simple scenario with a single confounder variable at each stage with $H_{10}=H_{11}={\left(1,O_{1}\right)}^{⊤},{\overset{ˇ}{H}}_{20}={\left(Y_{2},1,O_{1},A_{1},O_{1}A_{1},O_{2}\right)}^{⊤}$, and $H_{21}={\left(1,A_{1},O_{2}\right)}^{⊤}$. Specifically, recalling that $\sigma (x)\equiv 1/\left(1+e^{-1}\right)$, we sequentially generate

$$\begin{array}{lll} O_{1}∼\text{Bern}(0.5), & A_{1}∼\text{Bern}\left(\sigma \left\{H_{10}^{⊤}\xi_{1}^{0}\right\}\right),\; & Y_{2}∼𝒩\left({\overset{ˇ}{X}}_{1}^{⊤}\theta_{1}^{0},1\right),\\ O_{2}∼𝒩\left({\overset{ˇ}{H}}_{20}^{⊤}\delta^{0},2\right), & A_{2}∼\text{Bern}\left(\sigma \left\{H_{20}^{⊤}\xi_{2}^{0}+\xi_{26}^{0}O_{2}^{2}\right\}\right),\;\text{and} & Y_{3}∼𝒩(m_{3}\left\{{\overset{ˇ}{H}}_{20}\right\},2), \end{array}$$

where $m_{3}\left\{{\overset{ˇ}{H}}_{20}\right\}=H_{20}^{⊤}\beta_{2}^{0}+A_{2}\left(H_{21}^{⊤}\gamma_{2}^{0}\right)+\beta_{27}^{0}O_{2}^{2}Y_{2}sin\left\{{\left[O_{2}^{2}\left(Y_{2}+1\right)\right]}^{-1}\right\}$. Surrogates are generated as $W_{t}=\left\lfloor Y_{t+1}+Z_{t}\right\rfloor ,\;Z_{t}∼𝒩\left(0,\;\sigma_{z,t}^{2}\right),\;t=1, 2$ where ⌊x⌋ corresponds to the integer part of x∈ℝ. Throughout, we let $\xi_{1}^{0}=(0.3,-0.5{)}^{⊤},\;\beta_{1}^{0}=(1, 1{)}^{⊤},\gamma_{1}^{0}=(1,-2{)}^{⊤}\delta^{0}=(0, 0.5,-0.75, 0.25{)}^{⊤},\xi_{2}^{0}=(0, 0.5, 0.1,-1,-0.1{)}^{⊤}\;\beta_{2}^{0}=(.1, 3,0, 0.1,-0.5,-0.5{)}^{⊤},\gamma_{2}^{0}=(1, 0.25, 0.5{)}^{⊤}$.

We consider an additional case to mimic the structure of the EHR data set used for the real-data application. Outcomes $Y_{t}$ are binary, we use a higher number of covariates for the *Q* functions and multivariate count surrogates $W_{t}t=1, 2$. Data is simulated with $H_{10}={\left(1,O_{1},\ldots ,O_{6}\right)}^{⊤},\;H_{11}={\left(1,O_{2},\ldots ,O_{6}\right)}^{⊤},\;{\overset{ˇ}{H}}_{20}={\left(Y_{2},1,O_{1},\ldots ,O_{6},A_{1},Z_{21},Z_{22}\right)}^{⊤}$, and $H_{21}={\left(1,O_{1},\ldots ,O_{4},A_{1},Z_{21},Z_{22}\right)}^{⊤}$, generated according to

$$\begin{array}{lll} O_{1}∼𝒩\left(0,I_{6}\right), & A_{1}∼\text{Bern}\left(\sigma \left\{H_{10}^{⊤}\xi_{1}^{0}\right\}\right), & Y_{2}∼\text{Bern}\left(\sigma \left\{{\overset{ˇ}{X}}_{1}^{⊤}\theta_{1}^{0}\right\}\right),\\ O_{2}={\left[I\left\{Z_{1}>0\right\},I\left\{Z_{2}>0\right\}\right]}^{⊤} & A_{2}∼\text{Bern}\;\left({\tilde{m}}_{2}\left\{{\overset{ˇ}{H}}_{20}\right\}\right),\;\text{and} & Y_{3}∼\text{Bern}({\tilde{m}}_{3}\left\{{\overset{ˇ}{H}}_{20}\right\}), \end{array}$$

with ${\tilde{m}}_{2}=\sigma \;\left\{H_{20}^{⊤}\xi_{2}^{0}+{\tilde{\xi }}_{2}^{⊤}O_{2}\right\},\;{\tilde{m}}_{3}\left({\overset{ˇ}{H}}_{20}\right)=H_{20}^{⊤}\beta_{2}^{0}+A_{2}\left(H_{21}^{⊤}\gamma_{2}^{0}\right)+{\tilde{\beta }}_{2}^{⊤}O_{2}Y_{2}sin\left\{{\left\|O_{2}\right\|}_{2}^{2}/\left(Y_{2}+1)\}\right)\right.$ and $Z_{l}=O_{1l}\delta_{l}^{0}+\epsilon_{z},\;\epsilon_{z}∼𝒩(0, 1)\;l=1, 2$. The dimensions for the *Q* functions are 13 and 37 for the first and second stage respectively, which match with our IBD data set discussed in Section 6.2. The surrogates are generated according to $W_{t}=\left\lfloor Z_{t}\right\rfloor$, with $Z_{t}∼𝒩\left(\alpha^{⊤}\left(1,O_{t},A_{t},Y_{t}\right),I\right)$. Parameters are set to $\xi_{1}^{0}=(-0.1, 1,-1, 0.1{)}^{⊤},\;\beta_{1}^{0}=(0.5, 0.2,-1,-1, 0.1,-0.1, 0.1{)}^{⊤},\;\gamma_{1}^{0}=(1,-2,-2,-0.1, 0.1,-1.5{)}^{⊤}$, $\xi_{2}^{0}=(0, 0.5, 0.1,-1, 1,-0.1{)}^{⊤},\;\beta_{2}^{0}={\left(1,\beta_{1}^{0},0.25,-1,-0.5\right)}^{⊤}$, $\gamma_{2}^{0}=(1, 0.1,-0.1, 0.1,-0.1, 0.25,-1,-0.5{)}^{⊤}$, and $\alpha =(1,0,1{)}^{⊤}$.

Finally, we demonstrate how the method generalizes to T>2 stages. We extend our continuous simulation set-up into a data generation process which depends recursively on previous time steps at any given stage. The first stage has a single covariate: $H_{10}=H_{11}={\left(1,O_{1}\right)}^{⊤}$, then for t=2,…,T we have ${\overset{ˇ}{H}}_{t0}={\left(Y_{2},\ldots ,Y_{t},1,O_{t-1},A_{t-1},O_{t-1}A_{t-1},O_{t}\right)}^{⊤}$, and $H_{t1}={\left(1,A_{t-1},O_{t}\right)}^{⊤}$. We generate the data for the first stage as

$$O_{1}∼\text{Bern}(0.5),\;A_{1}∼\text{Bern}\left(\sigma \left\{H_{10}^{⊤}\xi_{1}^{0}\right\}\right),\;Y_{2}∼𝒩\left({\overset{ˇ}{X}}_{1}^{⊤}\theta_{1}^{0},1\right),$$

we then proceed sequentially for t=2,…,T+1 generating data according to the following models:

$$O_{t}∼𝒩\left({\left[1,O_{t-1},A_{t-1},O_{t-1}A_{t-1}\right]}^{⊤}\delta_{t}^{0},2\right),\;Y_{t+1}∼𝒩\left(m_{t}\left\{{\overset{ˇ}{H}}_{t0}\right\},2\right),\;\text{and}\;A_{t}∼\text{Bern}\left(\sigma \left\{{\left[1,O_{t-1},A_{t-1},O_{t-1}A_{t-1},O_{t},Y_{t-1}\right]}^{⊤}\xi_{t}^{0}+\xi_{t\text{*}}^{0}\text{sin}\;O_{t}^{2}\right\}\right).$$

where $m_{t}\left\{{\overset{ˇ}{H}}_{t0}\right\}=H_{t0}^{⊤}\beta_{t}^{0}+A_{t}\left(H_{t1}^{⊤}\gamma_{t}^{0}\right)+\beta_{t*}^{0}O_{t}^{2}Y_{t}sin\left\{{\left[O_{t}^{2}\left(Y_{t}+1\right)\right]}^{-1}\right\}$.

Throughout, we let $\xi_{1}^{0}=(0.3,-0.5{)}^{⊤},\;\beta_{1}^{0}=(0.1, 1{)}^{⊤},\;\gamma_{1}^{0}=(1,-0.2{)}^{⊤}$, $\delta_{t}^{0}=(0, 0.5,-0.75, 0.25, 1{)}^{⊤},\;\xi_{t}^{0}=(0, 0.5, 0.1,-1,-0.1, 1{)}^{⊤},\;\beta_{t}^{0}=(0.1, 0,0.1,-0.5,-0.5, 0.1{)}^{⊤}$, $\gamma_{t}^{0}=(1, 0.25, 0.5{)}^{⊤}$. Surrogates are generated as $W_{t}=\left\lfloor Y_{t+1}+Z_{t}\right\rfloor ,Z_{t}∼𝒩\left(0,\sigma_{z,t}^{2}\right)$, where ⌊x⌋ corresponds to the integer part of x∈ℝ.

For two-stage settings, we fit models $Q_{1}\left(H_{1},A_{1}\right)=H_{10}^{⊤}\beta_{1}^{0}+A_{1}\left(H_{11}^{⊤}\gamma_{1}^{0}\right),Q_{2}\left({\overset{ˇ}{H}}_{2},A_{2}\right)={\overset{ˇ}{H}}_{20}^{⊤}\beta_{2}^{0}+A_{2}\left(H_{21}^{⊤}\gamma_{2}^{0}\right)$ for the *Q* functions, $\pi_{1}\left(H_{1}\right)=\sigma \left(H_{10}^{⊤}\xi_{1}\right)$ and $\pi_{2}\left({\overset{ˇ}{H}}_{2}\right)=\sigma \;\left({\overset{ˇ}{H}}_{20}^{⊤}\xi_{2}\right)$ for the propensity scores. We fit analogous models for T>2-stage settings: $Q_{t}\left({\overset{ˇ}{H}}_{t},A_{t}\right)={\overset{ˇ}{H}}_{t0}^{⊤}\beta_{t}^{0}+A_{t}\left(H_{t1}^{⊤}\gamma_{t}^{0}\right)$, and $\pi_{t}\left({\overset{ˇ}{H}}_{t}\right)=\sigma \;\left({\overset{ˇ}{H}}_{t0}^{⊤}\xi_{t}\right)$ for t=1,…,T. To index mis-specification in the fitted *Q*-learning and the propensity score models, we use parameters $\xi_{26}^{0}$ and $\beta_{27}^{0},{\tilde{\xi }}_{2},{\tilde{\beta }}_{2}$, and $\xi_{t*}^{0},\beta_{t*}^{0}$ for T=2, and T>2 stage settings respectively. A value of 0 for these parameters corresponds to correct specification of their respective models. For mis-specification, we set $\xi_{26}^{0}=1,\;{\tilde{\xi }}_{2}=\frac{1}{\|(1,\ldots ,1){\|}_{2}}(1,\ldots ,1{)}^{⊤}$, and $\beta_{27}^{0}=1,{\tilde{\beta }}_{2}=\frac{1}{\|(1,\ldots ,1){\|}_{2}}(1,\ldots ,1{)}^{⊤}$ for the propensity score $\pi_{2}$ and $Q_{1},\;Q_{2}$ functions respectively. Similarly $\xi_{t*}^{0}=1$ and $\beta_{t*}^{0}=1$ for the general time horizon settings imply mis-specification of $\pi_{t}$, and $Q_{t}$ respectively for t=1…,T. Under mis-specification of the outcome model or propensity score model, the term omitted by the working models is highly non-linear, in which case the imputation model will be mis-specified as well. We show how our method does not need correct specification of the imputation model.

For the imputation models, we considered both random forest (RF) with 500 trees and basis expansion (BE) with piecewise-cubic splines with 2 equally spaced knots on the quantiles 33 and 67 (Hastie, 1992). We use 5-folds for our re-fitting bias correction step. For the two stage settings, we consider two choices of (n,N):(135, 1272) which are similar to the sizes of our EHR study and larger sizes of (500, 10000). For T>2 settings, we use (n,N)=(1000, 15000), and T=3, 5 stages, and (n,N)=(1500, 15000), and T=7 stages. We report statistics of the interaction-effect coefficients from the *Q* functions, in particular we show mean absolute bias $\frac{1}{3}\sum_{j=1}^{2}\left|\gamma_{tj}\right|$, and empirical standard error. For each configuration, we summarize results based on 1000 replications.

We start discussing results under correct specification of the *Q* functions. In Table 1, we present the results for the estimation of treatment interaction coefficients ${\overset{‾}{\gamma }}_{1},{\overset{‾}{\gamma }}_{2}$, under the correct model specification, continuous outcome setting with $\beta_{27}^{0}=\xi_{26}^{0}=0$. The complete tables for all $\bar{\theta }$ parameters for the continuous and EHR-like settings can be found in Appendix C. We report bias, empirical standard error (ESE), average standard error (ASE), 95% coverage probability (CovP) and relative efficiency (RE) defined as the ratio of the supervised over the SSL estimate ESE.

Overall, compared to the supervised approach, the proposed semi-supervised *Q*-learning approach has substantial gains in efficiency while maintaining comparable or even lower bias. This is likely due to the refitting step which helps take care of the finite sample bias, both from the missing outcome imputation and *Q* function parameter estimation. Imputation with BE yields slightly better estimates than when using RF, both in terms of efficiency and bias. Coverage probabilities are close to the nominal level thanks to the strong performance of our sample-split standard error estimator shown in Section 5.2.

**Figure 1:**
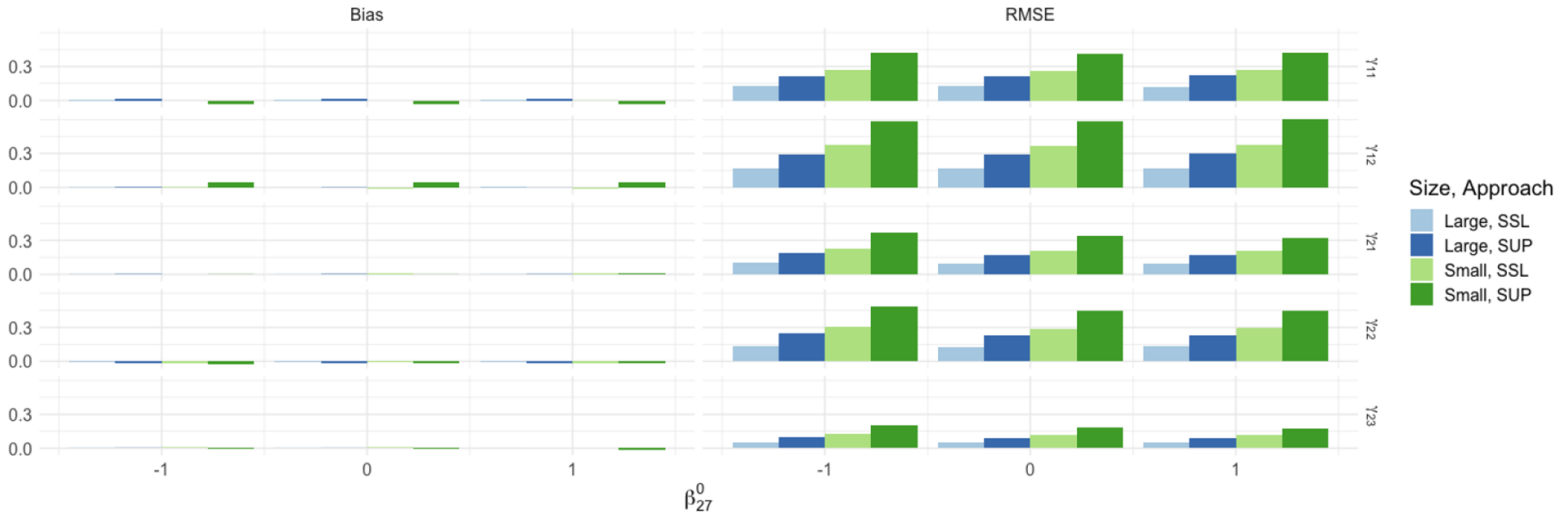
Monte Carlo estimates of bias and RMSE ratios for estimation of $\gamma_{11}$, $\gamma_{12}$, $\gamma_{21}$, $\gamma_{22}$, $\gamma_{23}$ under mis-specification of the *Q* functions through $\beta_{27}^{0}$. Results are shown for the large (N=10,000, n=500) and small (N=1,272, n=135) data samples for the continuous setting over 1,000 simulated data sets.

We next turn to *Q*-learning parameters under mis-specification of (1). Figure 1 shows the bias and root mean square error (RMSE) for the treatment interaction coefficients in the 2-stage *Q* functions. We focus on the continuous setting, where we set $\beta_{27}^{0}\in \{-1, 0,1\}$. Recall that $\beta_{27}^{0}\neq 0$ implies that both *Q* functions are mis-specified as the fitting of $Q_{1}$ depends on formulation of $Q_{2}$ as seen in (2). Semi-supervised *Q*-learning is more efficient for any degree of mis-specification for both small and large finite sample settings. As the theory predicts, there is no real difference in efficiency gain of SSL across mis-specification of the *Q* function models. This is because asymptotic distribution of ${\hat{\gamma }}_{\text{SSL}}$ shown in Theorems 2 & 3 are centered on the target parameters $\overset{‾}{\gamma }$. Thus, both SSL and SUP have negligible bias regardless of the true value of $\beta_{27}^{0}$.

Next we analyze performance of the doubly robust value function estimators for both continuous and EHR-like settings. Table 2 shows bias and RMSE across different sample sizes, and comparing SSL vs. SUP estimators. Results are shown for the correct specification of the *Q* functions and propensity scores, and when either is mis-specified. Bias across simulation settings is relatively similar between ${\hat{V}}_{{\text{SSL}}_{\text{DR}}}$ and ${\hat{V}}_{{\text{SUP}}_{\text{DR}}}$, and appears to be small relative to RMSE. The low magnitude of bias suggests both estimators are robust to model mis-specification. There is an exception on the EHR setting with small sample size, for which the bias is non-negligible. This is likely due to the fact that the *Q* function parameters to estimate are 13+37, and the propensity score functions have 12 parameters which add up to a large number relative to the labeled sample size: n=135. The SSL bias is lower in this case which could be due to the refitting step, which helped to reduce the finite sample bias. Efficiency gains of ${\hat{V}}_{{SSL}_{DR}}$ are consistent across model specification.

Finally we discuss results for the T>2 settings. Table 3 exhibits the interaction effect estimates $\gamma_{t}$ of the *Q* functions for t=1,…,T, with T=3, 5,7. Results show that SSL estimates remain more efficient than the supervised counterpart even as the time horizon increases. Although the SSL method requires imputation of $O\left(T^{2}\right)$ functions of the outcome, it still has low bias, and is much more efficient than the supervised counterpart as shown by the ≈2 relative efficiency for comparing SSL vs. supervised learning across settings. Similarly, Table 4, which displays the bias, standard error and the efficiency of the value function estimates, shows that for all time horizons the SSL outperforms its supervised counterpart in terms of efficiency. However as expected, both estimators loose efficiency as time horizon *T* increases. This is due to the fundamental information-theoretic difficult nature of the estimation problem for large *T*, in these contexts simplifying assumptions such as MDP or others are usually made (see Uehara et al., 2022). The high variance estimates for both approaches dominate the relative efficiency gain of SSL estimation as *T* grows. We also note that the relative efficiency is seemingly constant across correct and mis-specified models as our theoretical results state. We next illustrate our approach using an inflammatory bowl disease (IBD) data set.

### 6.2 Application to an EHR Study of Inflammatory Bowel Disease

Anti–tumor necrosis factor (anti-TNF) therapy has greatly changed the management and improved the outcomes of patients with IBD (Peyrin-Biroulet, 2010). However, it remains unclear whether a specific anti-TNF agent has any advantage in efficacy over other agents, especially at the individual level. There have been few randomized clinical trials performed to directly compare anti-TNF agents for treating IBD patients (Sands et al., 2019). Retrospective studies comparing infliximab and adalimumab for treating IBD have found limited and sometimes conflicting evidence of their relative effectiveness (Inokuchi et al., 2019; Lee et al., 2019; Osterman and Lichtenstein, 2017). There is even less evidence regarding optimal DTR for choosing these treatments over time (Ananthakrishnan et al., 2016). To explore this, we performed RL using data from a cohort of IBD patients previously identified via machine learning algorithms from the EHR systems of two tertiary referral academic centers in the Greater Boston metropolitan area (Ananthakrishnan et al., 2012). We focused on the subset of N=1,272 patients who initiated either Infliximab ($A_{1}=0$) or Adalimumab ($A_{1}=1$) and continued to be treated by either of these two therapies during the next 6 months. The observed treatment sequence distributions are shown in Table 5. The outcomes of interest are the binary indicator of treatment response at 6 months (t=2) and at 12 months (t=3), both of which were only available on a subset of n=135 patients whose outcomes were manually annotated via chart review.

To derive the DTR, we included gender, age, Charlson co-morbidity index (Charlson et al., 1987), prior exposure to anti-TNF agents, as well as mentions of clinical terms associated with IBD such as bleeding complications extracted from the clinical notes via natural language processing (NLP). These features and confounding variables are adjusted for in the *Q* functions at both time points. To improve the imputation of $Y_{t}$, we use 15 relevant NLP features such as mentions of rectal or bowel resection surgery as surrogates at t=1, 2. We transformed all count variables using x↦log(1+x) to decrease skewness in the distributions, and centered continuous features. We used RF with 500 trees to carry out the imputation step, and 5-fold cross-validation (CV) to estimate the value function. We only consider observations with estimated propensity scores within the [0.1,0.9] range to address the lack of overlap in the covariate distributions between treatment groups. We use this approximation to the optimal selection of observations proposed by Crump et al. (2009). Additionally, we use ridge regularization for our natural cubic splines model of the propensity scores.

The supervised and semi-supervised estimates are shown in Table 6 for the *Q*-learning models and in Table 7 for the value functions associated with the estimated DTR. Similar to those observed in the simulation studies, the semi-supervised *Q*-learning has more power to detect significant predictors of treatment response. Relative efficiency for almost all *Q* function estimates is near or over 2. The supervised *Q*-learning does not have the power to detect predictors such as prior use of anti-TNF agents, which are clearly relevant to treatment response (Ananthakrishnan et al., 2016). Semi-supervised *Q*-learning is able to detect that the efficacy of Adalimumab wears off as patients get older, meaning younger patients in the first stage experienced a higher rate of treatment response to Adalimumab, a finding that cannot be detected with supervised *Q*-learning. Additionally, supervised *Q*-learning does not pick up that there is a higher rate of response to Adalimumab among patients that are male or have experienced an abscess. This translates into a far from optimal treatment rule as seen in the cross-validated value function estimates. Table 7 reflects that using our semi-supervised approach to find the regime and to estimate the value function of such treatment policy yields a more efficient estimate, as the semi-supervised value function estimate ${\hat{V}}_{{\text{SUP}}_{\mathrm{DR}}}$ yielded a smaller standard error than that of the supervised estimate ${\hat{V}}_{{\mathrm{SUP}}_{\mathrm{DR}}}$. However, the standard errors are large relative to the point estimates. On the upside, they both yield estimates very close in numerical value which is reassuring: both should be unbiased as predicted by theory and simulations.

## 7. Discussion

We have proposed an efficient and robust strategy for estimating optimal DTRs and their value function in a setting where patient outcomes are scarce. In particular, we developed a two step estimation procedure amenable to non-parametric imputation of the missing outcomes. This helped us establish $\sqrt{n}$-consistency and asymptotic normality for both the *Q* function parameters $\hat{\theta }$ and the doubly robust value function estimator ${\hat{V}}_{{SSL}_{DR}}$. We additionally provided theoretical results which illustrate if and when the outcome-surrogates W contribute towards efficiency gain in estimation of ${\hat{\theta }}_{\text{SSL}}$ and ${\hat{V}}_{{\mathrm{SSL}}_{\mathrm{DR}}}$. These results let us conclude that our procedure is always preferable to using the labeled data only: since estimation is robust to mis-specification of the imputation models, our approach is safe to use and will be at least as efficient as the supervised methods.

Regarding our theoretical results, we believe that no specific aspects of the proofs explicitly require the number stages to be T=2. Indeed, we hypothesize that we can generalize the theory to any fixed T>2 using induction, as we have already proven the results for the first couple of stages. We chose to leave this generalization for future work, as we believe no new or particularly interesting theoretical methodology is required for this extension, beyond the already cumbersome notation and book-keeping in this relatively simpler T=2 setting.

Both the semi-supervised *Q* and value function estimation hold validity as long as the time horizon *T* remains finite. However, it is crucial to acknowledge that practical implementation may introduce instability to our estimators, particularly in cases involving large values of *T*. Instability in the presence of a large *T* scenario is a commonly observed phenomenon even for supervised approaches in RL problems. This issue has gained much attention in the context of policy evaluation, where it was shown that (supervised) doubly robust estimators can exhibit instability due to their reliance on products of *T* inverse propensity weights (cf. Jiang and Li, 2016a; Thomas and Brunskill, 2016a; Kallus and Uehara, 2020b). Such terms tend to exhibit significant variability as *T* increases, primarily because they often involve nested products of these weights (Levine et al., 2020).

In the offline RL literature, alternative policy evaluation methods have been proposed to improve stability. For instance, methods such as the weighted doubly robust estimator, MAGIC (Thomas and Brunskill, 2016a), and IH (Liu et al., 2018), have shown promise. Detailed information can be found in Voloshin et al. (2019). However, theoretical properties of these estimators are not as well-understood. Therefore, utilizing these ideas for our problem is not feasible with currently available tools.

As the time horizon *T* increases in our semi-supervised method, the number of terms requiring imputation for the *Q* and value function estimations is naturally higher. While this leads to increased variance, our approach offers a key advantage: the complexity of the conditional means to be imputed remains constant with respect to *T*. Therefore, efficiency is still gained as the primary source of variability arises from higher-order propensity scores, similar to previously mentioned supervised methods.

In particular, Appendix B delves into the generalization of our SSL *Q*-learning algorithm and value function estimation. We provide a thorough illustration of the functions requiring imputation. The analysis reveals that these functions exclusively consist of linear or quadratic terms involving missing outcomes, analogous to the T=2 case. Existing imputation techniques readily extend to T>2 scenarios, but the number of terms requiring imputation grows quadratically with time horizon $\left(O\left(T^{2}\right)\right)$. The normal equations for a three-stage setting is presented to concretely illustrate the advantages and challenges of our semi-supervised *Q*-learning approach. Additionally, the doubly robust SSL value function algorithm is extended for the general T>2 case. As expected, the value function also requires $O\left(T^{2}\right)$ terms to be imputed, limited to linear or quadratic terms of missing outcomes and propensity scores, again highlighting the manageability of complexity within our framework.

Finally, we are interested in extending this framework to handle missing at random (MAR) sampling mechanisms in the future. In the EHR setting, it is feasible to sample a subset of the data completely at random in order to annotate the records. Hence, we argue that our missingness assumption, which requires that the labeled sample has the same distribution as the unlabeled sample, is satisfied by design as we choose a random sample from the unlabeled data and then label it. However, the MAR context allows us to leverage different data sources for ℒ and 𝒰. For example, we could use an annotated EHR data cohort and a large unlabeled registry data repository for our inference, ultimately making the policies and value estimation more efficient and robust. We believe this line of work has the potential to leverage massive observational cohorts, which will help to improve personalized clinical care for a wide range of diseases.

## Figures and Tables

**Table 1:** Bias, empirical standard error (ESE) of the supervised and the SSL estimators with either random forest or basis expansion imputation strategies for ${\overset{‾}{\gamma }}_{1}$, ${\overset{‾}{\gamma }}_{2}$ when (a) n=135 and N=1272 and (b) n=500 and N=10,000. For the SSL estimators, we also obtain the average of the estimated standard errors (ASE) as well as the empirical coverage probabilities (CovP) of the 95% confidence intervals.

(a) n=135 and N=1272												
	Supervised		Semi-Supervised									
			Random Forests					Basis Expansion				
Parameter	Bias	ESE	Bias	ESE	ASE	CovP	RE	Bias	ESE	ASE	CovP	RE
		
$\gamma_{11}=1.4$	−0.03	0.41	0.00	0.26	0.24	0.93	1.57	0.00	0.24	0.23	0.93	1.68
$\gamma_{12}=-2.6$	0.04	0.58	−0.01	0.36	0.34	0.94	1.61	−0.02	0.35	0.31	0.90	1.69
$\gamma_{21}=0.8$	0.00	0.34	0.01	0.21	0.20	0.93	1.61	0.00	0.20	0.19	0.94	1.71
$\gamma_{22}=0.2$	−0.02	0.45	−0.01	0.28	0.28	0.95	1.60	−0.01	0.27	0.26	0.94	1.70
$\gamma_{23}=0.5$	0	0.18	0.01	0.11	0.11	0.94	1.59	0.00	0.11	0.11	0.94	1.68

(b) n=500 and N=10,000												
Parameter	Supervised		Semi-Supervised									
			Random Forests					Basis Expansion				
	Bias	ESE	Bias	ESE	ASE	CovP	RE	Bias	ESE	ASE	CovP	RE
		
$\gamma_{11}=1.4$	0.01	0.22	0.01	0.12	0.11	0.92	1.76	0.01	0.12	0.11	0.92	1.80
$\gamma_{12}=-2.6$	0	0.29	0	0.17	0.16	0.93	1.73	−0.01	0.16	0.15	0.93	1.80
$\gamma_{21}=0.8$	0.00	0.17	0.00	0.10	0.09	0.93	1.80	0.00	0.09	0.09	0.93	1.86
$\gamma_{22}=0.2$	−0.01	0.23	0	0.13	0.12	0.93	1.81	0	0.13	0.12	0.94	1.83
$\gamma_{23}=0.5$	0.00	0.09	0.00	0.05	0.05	0.94	1.78	0.00	0.05	0.05	0.95	1.81

**Table 2:** Bias, empirical standard error (ESE) of the supervised estimator ${\hat{V}}_{{SSL}_{DR}}$ and bias, ESE, average standard error (ASE) and coverage probability (CovP) for ${\hat{V}}_{{SSL}_{DR}}$ with either random forest or basis expansion imputation strategies when (a) n=135 and N=1272 and (b) n=500 and N=10,000. We show performance and relative efficiency across both simulation settings for estimation under correct models, and mis-specification of *Q* function or propensity score function.

(a) n=135 and N=1272														
Setting	Model	$\overset{‾}{V}$	Supervised		Semi-Supervised									
			Bias	ESE	Random Forests					Basis Expansion				
					Bias	ESE	ASE	CovP	RE	Bias	ESE	ASE	CovP	RE
			
Continuous	Correct	6.08	0.02	0.27	0.04	0.21	0.24	0.97	1.27	0.02	0.23	0.25	0.97	1.18
	Missp. *Q*	6.34	0.01	0.24	0.03	0.19	0.22	0.97	1.27	0.00	0.20	0.22	0.97	1.20
	Missp. π	6.08	0.01	0.28	0.02	0.22	0.24	0.97	1.24	0.01	0.25	0.25	0.97	1.12
EHR	Correct	1.38	0.09	0.15	0.05	0.12	0.12	0.94	1.24	0.04	0.13	0.12	0.95	1.12
	Missp. *Q*	1.43	0.09	0.14	0.04	0.12	0.12	0.96	1.12	0.03	0.14	0.12	0.95	1.02
	Missp. π	1.38	0.09	0.15	0.05	0.14	0.13	0.96	1.13	0.04	0.14	0.13	0.96	1.05
(b) n=500 and N=10,000														
Setting	Model	$\overset{‾}{V}$	Supervised		Semi-Supervised									
			Bias	ESE	Random Forests					Basis Expansion				
					Bias	ESE	ASE	CovP	RE	Bias	ESE	ASE	CovP	RE
			
Continuous	Correct	6.08	0.02	0.15	0.03	0.11	0.12	0.96	1.32	0.02	0.13	0.13	0.95	1.16
	Missp. *Q*	6.34	0.01	0.13	0.03	0.10	0.10	0.96	1.31	0.01	0.11	0.11	0.96	1.16
	Missp. π	6.08	0.01	0.14	0.03	0.11	0.12	0.96	1.28	0.02	0.12	0.12	0.95	1.16
EHR	Correct	1.38	0.02	0.07	0.01	0.04	0.06	0.99	1.55	0.00	0.06	0.06	0.98	1.23
	Missp. *Q*	1.43	0.01	0.07	0.00	0.04	0.05	0.99	1.66	0.00	0.05	0.06	0.98	1.35
	Missp. π	1.38	0.02	0.08	0.01	0.06	0.07	0.99	1.22	0.00	0.07	0.07	0.97	1.03

**Table 3:** Mean absolute bias, and empirical standard error (ESE) of the supervised and the SSL *Q* function interaction effect estimates $\gamma_{t}$, for T=3, 5,7 stages. Random forest imputation is used for SSL estimation.

	T=3					T=5					T=7				
	Sup.		SSL			Sup.		SSL			Sup.		SSL		
	Bias	ESE	Bias	ESE	RE	Bias	ESE	Bias	ESE	RE	Bias	ESE	Bias	ESE	RE
$\gamma_{1}$	0.02	0.18	0.09	0.09	2.14	0.00	0.18	0.13	0.08	2.32	0.01	0.19	0.14	0.08	2.32
$\gamma_{2}$	0.03	0.13	0.03	0.07	2.0	0.04	0.12	0.06	0.06	2.07	0.03	0.13	0.07	0.06	2.21
$\gamma_{3}$	0.01	0.11	0.1	0.06	2.08	0.01	0.12	0.06	0.06	2.09	0.05	0.12	0.06	0.06	2.09
$\gamma_{4}$						0.03	0.11	0.08	0.05	2.01	0.08	0.12	0.09	0.06	2.12
$\gamma_{5}$						0.01	0.1	0.07	0.04	1.87	0.04	0.11	0.08	0.06	1.95
$\gamma_{6}$											0.03	0.08	0.1	0.05	1.89
$\gamma_{7}$											0.02	0.03	0.08	0.04	1.97

**Table 4:** Bias, and empirical standard error (ESE) of the value function estimators ${\hat{V}}_{{SUP}_{DR}}$, ${\hat{V}}_{{SSL}_{DR}}$ for T=3,5,7 stages. Random forests imputation is used for semi-supervised estimation. We show performance and relative efficiency for estimation under correct models, and mis-specification of *Q* function or propensity score functions.

	T=3					T=5					T=7				
	Sup.		SSL			Sup.		SSL			Sup.		SSL		
Model	Bias	ESE	Bias	ESE	RE	Bias	ESE	Bias	ESE	RE	Bias	ESE	Bias	ESE	RE
															
Correct	0.0	0.11	0.01	0.08	1.47	0.0	0.25	0.0	0.19	1.34	0.05	0.68	0.05	0.61	1.12
Missp. *Q*	0.0	0.11	0.01	0.07	1.52	0.02	0.32	0.01	0.25	1.29	0.02	0.61	0.02	0.54	1.09
Missp. π	0.01	0.11	0.01	0.07	1.49	0.03	0.33	0.02	0.24	1.38	0.05	0.8	0.03	0.66	1.22

**Table 5:** Distribution of treatment trajectories for an observed sample of size 1407.

		$A_{1}$	
	
		0	1
$A_{2}$	0	912	327
	1	27	183

**Table 6:** Results for the Inflammatory Bowel Disease data set, for first and second stage regressions. Fully supervised *Q*-learning is shown on the left and semi-supervised is shown on the right. Last columns in the panels show relative efficiency (RE) defined as the ratio of standard errors of the semi-supervised vs. supervised method, RE greater than one favors semi-supervised. Statistically significant coefficients at the 0.05 level are in bold.

	
Stage 1 Regression								Stage 2 Regression							
Parameter	Supervised			Semi-Supervised				Supervised				Semi-Supervised			
	Estimate	SE	P-val	Estimate	SE	P-val	RE	Parameter	Estimate	SE	P-val	Estimate	SE	P-val	RE
					
Intercept	**0.424**	**0.082**	**0.00**	**0.518**	**0.028**	**0.00**	2.937	*Y*_1_	**0.37**	**0.11**	**0.00**	**0.55**	**0.05**	**0.00**	2.08
Female	−0.237	0.167	0.16	**−0.184**	**0.067**	**0.007**	2.514	Intercept	0.08	0.06	0.17	0.04	0.02	0.14	2.40
Age	0.155	0.088	0.081	**0.18**	**0.034**	**0.00**	2.588	Female	−0.01	0.10	0.92	−0.00	0.05	0.98	2.21
Charlson Score	0.006	0.072	0.929	−0.047	0.026	0.075	2.776	Age	0.05	0.06	0.35	**0.07**	**0.02**	**0.00**	2.33
Prior anti-TNF	−0.038	0.06	0.524	**−0.085**	**0.019**	**0.00**	3.177	Charlson Score	0.04	0.04	0.33	**0.06**	**0.02**	**0.01**	2.06
Perianal	**0.138**	**0.06**	**0.022**	**0.179**	**0.022**	**0.00**	2.688	Prior anti-TNF	−0.05	0.05	0.29	**−0.09**	**0.02**	**0.00**	2.39
Bleeding	0.049	0.08	0.54	0.058	0.03	0.055	2.675	Perianal	−0.01	0.04	0.80	**−0.03**	**0.02**	**0.06**	2.31
A1	0.163	0.488	0.739	0.148	0.206	0.473	2.374	Bleeding	−0.04	0.05	0.49	−0.03	0.03	0.29	2.14
Female×*A*_1_	0.168	0.696	0.81	−0.042	0.287	0.886	2.424	A1	0.11	0.25	0.67	0.03	0.10	0.74	2.60
Age×*A*_1_	−0.177	0.264	0.503	**−0.278**	**0.109**	**0.013**	2.418	Abscess_2_	0.06	0.04	0.16	**0.05**	**0.01**	**0.00**	2.68
Charlson Score×*A*_1_	0.136	0.391	0.728	0.195	0.178	0.276	2.194	Fistula_2_	0.02	0.05	0.67	0.01	0.02	0.62	2.33
Perianal×*A*_1_	−0.113	0.226	0.618	−0.019	0.08	0.808	2.838	Female×*A*_1_	0.13	0.38	0.74	0.17	0.16	0.30	2.37
Bleeding×*A*_1_	0.262	0.364	0.474	0.127	0.161	0.431	2.267	Age×*A*_1_	−0.02	0.12	0.88	−0.09	0.06	0.17	1.94
								Charlson Score×*A*_1_	−0.02	0.16	0.89	0.04	0.07	0.55	2.19
								Perianal×*A*_1_	−0.14	0.09	0.15	**−0.17**	**0.04**	**0.00**	2.34
								Bleeding×*A*_1_	0.13	0.20	0.51	0.03	0.09	0.76	2.17
								A2	0.07	0.17	0.69	**0.22**	**0.07**	**0.00**	2.55
								Female×*A*_2_	−0.39	0.28	0.16	**−0.51**	**0.11**	**0.00**	2.53
								Age×*A*_2_	0.09	0.10	0.40	**0.15**	**0.04**	**0.00**	2.27
								Charlson Score×*A*_2_	0.01	0.07	0.84	−0.03	0.03	0.42	2.08
								Perianal×*A*_2_	**0.20**	**0.09**	**0.04**	**0.23**	**0.04**	**0.00**	2.23
								Bleeding×*A*_2_	0.03	0.08	0.77	0.02	0.04	0.49	2.34
								Abscess_2_ × *A*_2_	−0.13	0.07	0.06	**−0.09**	**0.03**	**0.00**	2.31
								Fistula_2_ × *A*_2_	−0.04	0.06	0.56	−0.03	0.03	0.36	2.17

**Table 7:** Value function estimates for the Inflammatory Bowel Disease data set. The first row shows the estimate for treatment rule learned using U and its respective value function, the second row shows the same for a rule estimated using L and its estimated value.

	Estimate	SE
${\hat{V}}_{{SUP}_{DR}}$	0.851	0.486
${\hat{V}}_{{SSL}_{DR}}$	0.871	0.397

**Figure C.1:**
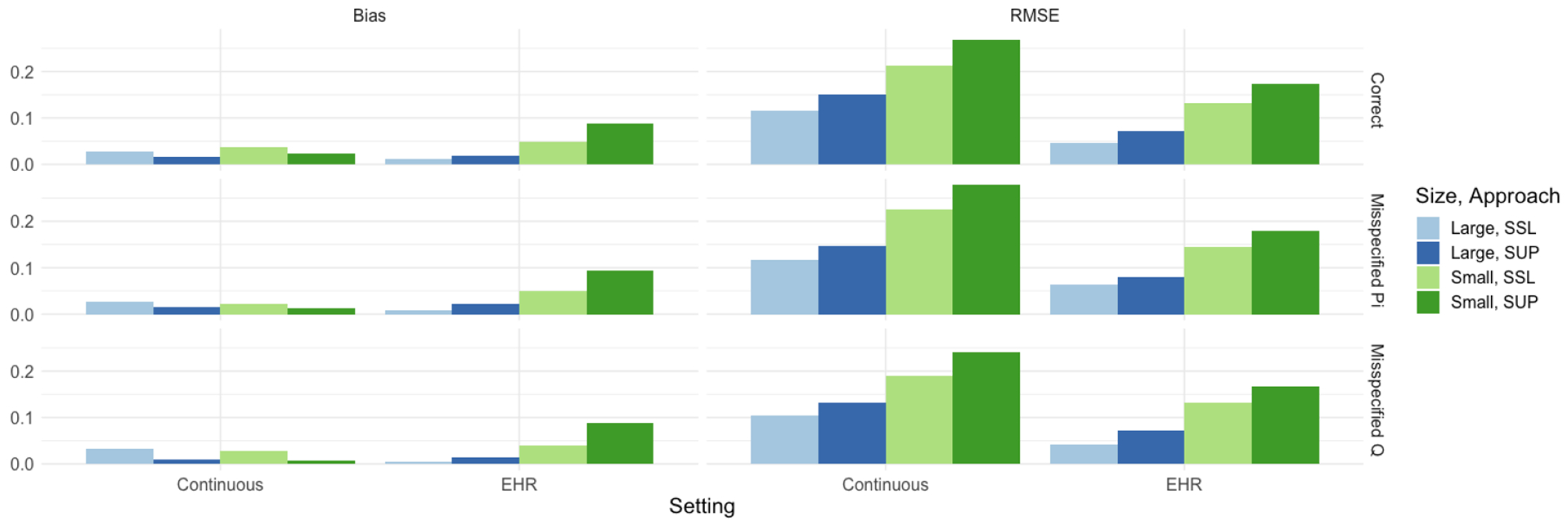
Monte Carlo estimates for doubly-robust value function estimation: ${\hat{V}}_{{SSL}_{DR}}$, ${\hat{V}}_{{\text{SUP}}_{\text{DR}}}$ under continuous, and EHR settings. Columns show bias and RMSE respectively, rows show different mis-specification scenarios. Results are shown for the large (N=10,000,n=500) and small data samples (N=1,272, n=135) for the continuous setting over 1,000 simulated data sets.

## Appendix A. Notation

We first summarize the main notation used throughout the paper in Table 8. Note that we use a few conventions: 1) The check symbol: “ˇ” denotes the entire set of features available, including outcomes when available (for t=2), this symbol is used for both the patient history ${\overset{ˇ}{H}}_{t}$, and the linear features for the *Q*-functions ${\overset{ˇ}{X}}_{t}$. 2) We use the bar symbol: “¯” to denote population parameters, for example $\overset{‾}{V}$ is the population value function under the optimal treatment policy ${\overset{‾}{d}}_{t}$. 3) As usual, the hat symbol “^” is used to denote estimated parameters.

**Table 8:** Main notation.

Notation	Definition
$O_{t}\in {\mathbb{R}}^{d_{t}^{o}}$	Vector of covariates measured prior to t
$A_{t}\in \{0, 1\}$	A treatment indicator variable at t
$Y_{t+1}\in \mathbb{R}$	The outcome observed at t+1
$W_{t}\in {\mathbb{R}}^{d_{t}^{\omega }}$	A $d_{t}^{\omega }$-dimensional vector of post-treatment covariates
$U_{t}={\left(O_{t}^{⊤},A_{t},W_{t}^{⊤}\right)}^{⊤}$	All observed variables for labeled & unlabeled data at t
$\vec{U}={\left(U_{1}^{⊤},U_{2}^{⊤}\right)}^{⊤}$	All observed variables for t=1, 2
$\phi_{tk}(\cdot )$	Pre-specified basis functions for $Q_{t}$ function regressions
$H_{1k}=\phi_{1k}\left(O_{1}\right)$	Baseline (k=0), or interaction (k=1) features at t=1
$H_{2k}=\phi_{2k}\left(O_{1},A_{1},O_{2}\right)$	Baseline (k=0), or interaction (k=1) features at t=2
$H_{t}\equiv {\left[H_{t0}^{⊤},H_{t1}^{⊤}\right]}^{⊤}$	Patient’s concatenated history features at t
${\overset{ˇ}{H}}_{1}=H_{1}$	Patient’s history features at t=1
${\overset{ˇ}{H}}_{2}={\left(Y_{2},H_{2}^{⊤}\right)}^{⊤}$	Patient’s history including previous outcome at t=2
$X_{t}={\left[H_{t0}^{⊤},A_{1}H_{t1}^{⊤}\right]}^{⊤}$	Linear regression features available for all data
${\overset{ˇ}{X}}_{1}=X_{1}$	Linear regression features for *Q* function at t=1
${\overset{ˇ}{X}}_{2}={\left(Y_{2},X_{2}^{⊤}\right)}^{⊤}$	Linear regression features for *Q* function at t=2
$\theta_{t}={\left(\beta_{t}^{⊤},\gamma_{t}^{⊤}\right)}^{⊤}$	*Q* function working model parameters
$\Sigma_{t}=𝔼\left[{\overset{ˇ}{X}}_{t}{\overset{ˇ}{X}}_{t}^{⊤}\right]$	Second moment for all features at stages t=1, 2
${\hat{Y}}_{2}^{*}=Y_{2}+\underset{a_{2}}{max}\;Q_{2}\left({\overset{ˇ}{H}}_{2},a_{2};{\hat{\theta }}_{2}\right)$	Labeled-data stage 1 estimated pseudo-outcome
${\overset{‾}{Y}}_{2}^{*}=Y_{2}+\underset{a_{2}}{max}\;Q_{2}\left({\overset{ˇ}{H}}_{2},a_{2};{\bar{\theta }}_{2}\right)$	Stage 1 population pseudo-outcome
$\mu_{2t}(\vec{U})=𝔼\left(Y_{2}Y_{t}|\vec{U}\right)$	Example of a conditional mean function
${\hat{m}}_{2t}(\vec{U})$	Weakly or non-parametric estimator for $\mu_{2t}(\vec{U})$
${\hat{\eta }}_{2t}$	Refitting-step linear parameter, ensures (3) is satisfied
${\hat{\mu }}_{2t}(\vec{U})=\frac{1}{K}\sum_{k}{\hat{m}}_{2t}^{\left(-k\right)}(\vec{U})+{\hat{\eta }}_{2t}$	Augmented cross-fitted model for $\mu_{2t}(\vec{U})$
${\hat{d}}_{t},{\hat{d}}_{t}\left(H_{t}\right),d_{t}\left(H_{t};{\hat{\theta }}_{t}\right)$	Estimated optimal treatment given history $H_{t}$
${\overset{‾}{d}}_{t}\equiv {\overset{‾}{d}}_{t}\left(H_{t}\right)\equiv d_{t}\left(H_{t},{\bar{\theta }}_{t}\right)$	Population-parameter optimal treatment given $H_{t}$
$\pi_{t}\left({\overset{ˇ}{H}}_{t}\right)=P\left\{A_{t}=1|{\overset{ˇ}{H}}_{t}\right\}$	Treatment propensity scores at stages t=1, 2
$\omega_{t}\left({\overset{˘}{H}}_{t},A_{t},\Theta \right)$	Inverse probability weights at stages t=1, 2
$\overset{‾}{V}$	Expected population outcome under optimal policy
${\hat{V}}_{{\mathrm{SUP}}_{\mathrm{DR}}}$	Supervised doubly robust estimator for $\overset{‾}{V}$
${\hat{V}}_{{\mathrm{SSL}}_{\mathrm{DR}}}$	Semi-Supervised doubly robust estimator for $\overset{‾}{V}$

## Appendix B. SSL *Q*-Learning and Off-Policy Value Estimation for T>2 Stages

In this section we explore generalizing our methods to an arbitrary finite time horizon *T* and discuss some advantages and challenges of our method in this context. We start by showing the generalized SSL *Q* learning algorithm, and the functions that need imputation by writing the missing outcomes explicitly. We demonstrate that the functions of the outcome that need to be imputed consist of linear outcomes or products of at most two missing outcomes. Next, we extend the doubly robust SSL value function algorithm for T>2. The value function, as expected, also has more terms to impute. However, these are all products of at most two missing outcomes or an outcome and a single propensity score function. We show that the terms in the individual products do not increase with *T*. Naturally, the number of conditional means needed to be imputed does increase. We end by highlighting the benefits and challenges of both algorithms applied to a time horizon larger than two stages.

### SSL Q-Learning

Extending our *Q* function notation for a general time horizon fixed at *T* time steps we define

$$Q_{t}\left({\overset{ˇ}{H}}_{t},A_{t}\right)\equiv 𝔼\left[Y_{t+1}+\underset{a}{\max}\;Q_{t+1}\left({\overset{ˇ}{H}}_{t+1},a\right)|{\overset{ˇ}{H}}_{t},A_{t}\right]\;\text{for}\;t=1,\ldots ,T,$$

and $Q_{T+1}\left({\overset{ˇ}{H}}_{T+1},A_{T+1}\right)\equiv 0$ for all ${\overset{ˇ}{H}}_{T+1},A_{T+1}$.

The corresponding working linear models for the *Q* functions with parameters $\theta_{t}={\left(\beta_{t}^{⊤},\gamma_{t}^{⊤}\right)}^{⊤},t=1,\ldots ,T$ are:

$$\begin{array}{l} Q_{1}\left({\overset{ˇ}{H}}_{1},A_{1};\theta_{1}^{0}\right)={\overset{ˇ}{X}}_{1}^{⊤}\theta_{1}^{0}=H_{10}^{⊤}\beta_{1}^{0}+A_{1}\left(H_{11}^{⊤}\gamma_{1}^{0}\right),\\ Q_{2}\left({\overset{ˇ}{H}}_{2},A_{2};\theta_{2}^{0}\right)={\overset{ˇ}{X}}_{2}^{⊤}\theta_{2}^{0}=Y_{2}\beta_{21}^{0}+H_{20}^{⊤}\beta_{22}^{0}+A_{2}\left(H_{21}^{⊤}\gamma_{2}^{0}\right),\\ \;\;\vdots \\ Q_{t}\left({\overset{ˇ}{H}}_{t},A_{t};\theta_{t}^{0}\right)={\overset{ˇ}{X}}_{t}^{⊤}\theta_{t}^{0}=\sum_{\ell =1}^{t-1}Y_{\ell +1}\beta_{t\ell }^{0}+H_{t0}^{⊤}\beta_{tt}^{0}+A_{t}\left(H_{t1}^{⊤}\gamma_{t}^{0}\right). \end{array}$$

The pseudo-outcomes for stages 1 and t=2,…,T under the linear *Q* function are

$${\bar{Y}}_{ti}^{\text{*}}=Y_{ti}+\sum_{\ell =1}^{t-1}Y_{\ell +1,i}\beta_{t\ell }^{0}+H_{t0}^{⊤}\beta_{tt}^{0}+{\left[H_{t1}^{⊤}\gamma_{t}^{0}\right]}_{+},t=2,\ldots ,T$$

Defining the outcome vector as ${\vec{Y}}_{t:2}\equiv {\left(Y_{t},Y_{t-1},\ldots ,Y_{2}\right)}^{⊤}$, the normal equations for *T*, t=2,…,T−1 and t=1 are $𝔼\left[{\overset{ˇ}{X}}_{T}\left(Y_{T+1}-{\overset{ˇ}{X}}_{T}^{⊤}\theta_{T}\right)\right]=0,𝔼\left[{\overset{ˇ}{X}}_{t}\left({\overset{‾}{Y}}_{t+1}^{*}-{\overset{ˇ}{X}}_{t}^{⊤}\theta_{t}\right)\right]=0$, and $𝔼\left[{\overset{ˇ}{X}}_{1}\left({\overset{‾}{Y}}_{2}^{*}-{\overset{ˇ}{X}}_{1}^{⊤}\theta_{1}\right)\right]=0$, which can be respectively written out as :

$$𝔼\;\left[\begin{array}{c} Y_{T}\left\{Y_{T+1}-\left({\vec{Y}}_{T:2}^{⊤},X_{T}^{⊤}\right)\theta_{T}\right\}\\ Y_{T-1}\left\{Y_{T+1}-\left({\vec{Y}}_{T:2}^{⊤},X_{T}^{⊤}\right)\theta_{T}\right\}\\ \vdots \\ Y_{2}\left\{Y_{T+1}-\left({\vec{Y}}_{T:2}^{⊤},X_{T}^{⊤}\right)\theta_{T}\right\}\\ X_{T}\left\{Y_{T+1}-\left({\vec{Y}}_{T:2}^{⊤},X_{T}^{⊤}\right)\theta_{T}\right\} \end{array}\right]=0,\;𝔼\;\left[\begin{array}{c} Y_{t}\left\{Y_{t+1}+\overset{t-1}{\underset{\ell =1}{\Sigma }}Y_{\ell +1}\beta_{t+1,\ell }^{0}+H_{t+1,0}^{⊤}\beta_{t+1,t+1}^{0}+{\left[H_{t+1,1}^{⊤}\gamma_{t+1}^{0}\right]}_{+}-\left({\vec{Y}}_{t:2}^{⊤},X_{t}^{⊤}\right)\theta_{t}\right\}\\ Y_{t-1}\left\{Y_{t+1}+\overset{t-1}{\underset{\ell =1}{\Sigma }}Y_{\ell +1}\beta_{t+1,\ell }^{0}+H_{t+1,0}^{⊤}\beta_{t+1,t+1}^{0}+{\left[H_{t+1,1}^{⊤}\gamma_{t+1}^{0}\right]}_{+}-\left({\vec{Y}}_{t:2}^{⊤},X_{t}^{⊤}\right)\theta_{t}\right\}\\ \vdots \\ Y_{2}\left\{Y_{t+1}+\overset{t-1}{\underset{\ell =1}{\Sigma }}Y_{\ell +1}\beta_{t+1,\ell }^{0}+H_{t+1,0}^{⊤}\beta_{t+1,t+1}^{0}+{\left[H_{t+1,1}^{⊤}\gamma_{t+1}^{0}\right]}_{+}-\left({\vec{Y}}_{t:2}^{⊤},X_{t}^{⊤}\right)\theta_{t}\right\}\\ X_{t}\left\{Y_{t+1}+\overset{t-1}{\underset{\ell =1}{\Sigma }}Y_{\ell +1}\beta_{t+1,\ell }^{0}+H_{t+1,0}^{⊤}\beta_{t+1,t+1}^{0}+{\left[H_{t+1,1}^{⊤}\gamma_{t+1}^{0}\right]}_{+}-\left({\vec{Y}}_{t:2}^{⊤},X_{t}^{⊤}\right)\theta_{t}\right\} \end{array}\right]=0,\;𝔼\left[X_{1}\left\{Y_{2}\left(1+\beta_{21}\right)+H_{20}^{⊤}\beta_{22}+{\left[H_{21}^{⊤}\gamma_{2}\right]}_{+}-X_{1}^{⊤}\theta_{1}\right\}\right]=0.$$

With the above we can generalize our robust imputation-based semi-supervised *Q*-learning with the same three steps: (i) imputation, (ii) refitting, and (iii) projection to the unlabeled data for *T* stages as follows.

#### Step I: Imputation

We use the usual weakly parametric or non-parametric imputation models for the conditional mean functions $\left\{\mu_{t}(\cdot ),\mu_{t^{\prime }t}(\cdot ),t^{\prime },t=2,\ldots ,T+1\right\}$, where $\mu_{t}(\vec{U})=𝔼\left(Y_{t}|\vec{U}\right)$ and $\mu_{t^{\prime }t}(\vec{U})=𝔼\left(Y_{t^{\prime }}Y_{t}|\vec{U}\right)$ consist. Notice the products consist of at most two missing outcomes. As in the case when T=2, we denote the corresponding estimated mean functions as $\left\{{\hat{m}}_{t}(\cdot ),{\hat{m}}_{t^{\prime }t}(\cdot ),t^{\prime },t=2,\ldots ,T+1\right\}$ under the corresponding imputation models $\left\{m_{t}(\vec{U}),m_{t^{\prime }t}(\vec{U}),t^{\prime },t=2,\ldots ,T+1\right\}$.

#### Step II: Refitting

The refitting ensures the validity of the SSL estimators under potential mis-specifications of the imputation models and helps to control for overfitting bias. We update the imputation model by expanding it to include linear effects of $\left\{X_{t},t=1,\ldots ,T\right\}$ with cross-fitting.

In this case, the final imputation models for $\left\{Y_{t},Y_{t^{\prime }}Y_{t},t=2,\ldots ,T+1\right\}$, denoted by $\left\{{\overset{‾}{\mu }}_{t}(\vec{U}),{\overset{‾}{\mu }}_{t^{\prime }t}\right\}$, need to satisfy

$$𝔼\;\left[{\vec{X}}_{t^{\prime }}\left\{Y_{t}-{\bar{\mu }}_{t}(\vec{U})\right\}\right]=0,\;t^{\prime }=1,\ldots ,T,t=2,\ldots ,t^{\prime }+1,\;𝔼\left\{Y_{t^{\prime }}Y_{t}-{\bar{\mu }}_{t^{\prime }t}(\vec{U})\right\}=0,\;t^{\prime }=2,\ldots ,T,t=2,\ldots ,T+1,$$

where feature vector $X_{t}^{\prime }$ has one intercept entry of 1, for $t^{\prime }=1,\ldots ,T$.

Next we expand $\left\{m_{t}(\vec{U}),m_{t^{\prime }t}(\vec{U})\right\}$. Using the same notation as in the main paper for the *K* random equal sized partitions of the labeled index set {1,…,n}, and using $\left\{{\hat{m}}_{t}^{\left(-k\right)}(\vec{U}),{\hat{m}}_{t^{\prime }t}^{\left(-k\right)}(\vec{U})\right\}$ for the counterpart of $\left\{{\hat{m}}_{t}(\vec{U}),{\hat{m}}_{t^{\prime }t}(\vec{U})\right\}$ with labeled observations in $\{1,..,n\}\setminus {ℐ}_{k}$ we obtain ${\hat{\eta }}_{t}$, and ${\hat{\eta }}_{t^{\prime }t}$ respectively as the solutions to

$$\sum_{k=1}^{K}\sum_{i\in {ℐ}_{k}}{\vec{X}}_{t^{\prime }i}\left\{Y_{ti}-{\hat{m}}_{t}^{\left(-k\right)}\left({\vec{U}}_{i}\right)-\eta_{t}^{⊤}{\vec{X}}_{i}\right\}=0,t^{\prime }=1,\ldots ,T,t=2,\ldots ,t^{\prime }+1,\;\sum_{k=1}^{K}\sum_{i\in {ℐ}_{k}}\left\{Y_{t^{\prime }i}Y_{ti}-{\hat{m}}_{t^{\prime }t}^{\left(-k\right)}\left({\vec{U}}_{i}\right)-\eta_{t^{\prime }t}\right\}=0,\;t^{\prime }=2,\ldots ,T,t=2,\ldots ,T+1.$$

Finally, we impute $Y_{t}$, and $Y_{t^{\prime }}Y_{t}$ respectively as ${\hat{\mu }}_{t}(\vec{U})=K^{-1}\sum_{k=1}^{K}{\hat{m}}_{t}^{\left(-k\right)}(\vec{U})+{\hat{\eta }}_{t}^{⊤}\vec{X}$, and ${\hat{\mu }}_{t^{\prime }t}(\vec{U})=K^{-1}\sum_{k=1}^{K}{\hat{m}}_{t^{\prime }t}^{\left(-k\right)}(\vec{U})+{\hat{\eta }}_{t^{\prime }t}$.

#### Step III: Projection

In the last step, we proceed to estimate $\hat{\theta }$ by replacing $\left\{Y_{t},Y_{t^{\prime }}Y_{t}\right\}$ in the *Q* function normal equations with their the imputed values $\left\{{\hat{\mu }}_{t}(\vec{U}),{\hat{\mu }}_{t^{\prime }t}(\vec{U})\right\}$ and project to the unlabeled data. For this we define vectors ${\vec{\overset{ˆ}{\mu }}}_{t:2}(\vec{U})={\left[{\hat{\mu }}_{t}(\vec{U}),{\hat{\mu }}_{t-1}(\vec{U}),\ldots ,{\hat{\mu }}_{2}(\vec{U})\right]}^{⊤}$, and ${\vec{\hat{\mu }}}_{t^{\prime },t:2}(\vec{U})={\left[{\hat{\mu }}_{t^{\prime }t}(\vec{U}),{\hat{\mu }}_{t^{\prime },t-1}(\vec{U}),\ldots ,{\hat{\mu }}_{t^{\prime }2}(\vec{U})\right]}^{⊤}$.

We obtain the final SSL estimators for $\theta_{t},\;t=T,\ldots ,1$ with the following regressions (We omit the dependency of the imputed models on $\vec{U}$ for notation brevity).

1. We get stage *T* regression parameters $\theta_{T}$ from:
  $${\mathbb{P}}_{N}\;\left[\begin{array}{c} {\hat{\mu }}_{T,T+1}-\left[{\vec{\hat{\mu }}}_{T,T:2}^{⊤},{\hat{\mu }}_{T}X_{T}^{⊤}\right]{\hat{\theta }}_{T}\\ {\hat{\mu }}_{T-1,T+1}-\left[{\vec{\hat{\mu }}}_{T-1,T:2}^{⊤},{\hat{\mu }}_{T-1}X_{T}^{⊤}\right]{\hat{\theta }}_{T}\\ \vdots \\ {\hat{\mu }}_{2,T+1}-\left[{\vec{\hat{\mu }}}_{2,T:2}^{⊤},{\hat{\mu }}_{2}X_{T}^{⊤}\right]{\hat{\theta }}_{T}\\ X_{T}\left\{{\hat{\mu }}_{T+1}-\left[{\vec{\hat{\mu }}}_{T:2}^{⊤},X_{T}^{⊤}\right]{\hat{\theta }}_{T}\right\} \end{array}\right]=0$$
2. For finding stage t=T−1,…,2 regression parameters, $\theta_{t}$, we use:
  $${\mathbb{P}}_{N}\;\left[\begin{array}{c} {\hat{\mu }}_{t,t+1}+\sum_{\ell =1}^{t-1}{\hat{\mu }}_{t,\ell +1}{\hat{\beta }}_{t+1,\ell }+{\hat{\mu }}_{t}\left\{H_{t+1,0}^{⊤}{\hat{\beta }}_{t+1,t+1}+{\left[H_{t+1,1}^{⊤}{\hat{\gamma }}_{t+1}\right]}_{+}\right\}\\ {\hat{\mu }}_{t-1,t+1}+\sum_{\ell =1}^{t-1}{\hat{\mu }}_{t-1,\ell +1}{\hat{\beta }}_{t+1,\ell }+{\hat{\mu }}_{t-1}\left\{H_{t+1,0}^{⊤}{\hat{\beta }}_{t+1,t+1}+{\left[H_{t+1,1}^{⊤}{\hat{\gamma }}_{t+1}\right]}_{+}\right\}\\ \vdots \\ {\hat{\mu }}_{2,t+1}+\sum_{\ell =1}^{t-1}{\hat{\mu }}_{2,\ell +1}{\hat{\beta }}_{t+1,\ell }+{\hat{\mu }}_{2}\left\{H_{t+1,0}^{⊤}{\hat{\beta }}_{t+1,t+1}+{\left[H_{t+1,1}^{⊤}{\hat{\gamma }}_{t+1}\right]}_{+}\right\}\\ X_{t}\left\{{\hat{\mu }}_{t+1}+\sum_{\ell =1}^{t-1}{\hat{\mu }}_{\ell +1}{\hat{\beta }}_{t+1,\ell }+H_{t+1,0}^{⊤}{\hat{\beta }}_{t+1,t+1}+{\left[H_{t+1,1}^{⊤}{\hat{\gamma }}_{t+1}\right]}_{+}\right\} \end{array}\right]\;-{\mathbb{P}}_{N}\;\left[\begin{array}{c} \left[{\vec{\hat{\mu }}}_{t,t:2}^{⊤},{\hat{\mu }}_{t}X_{t}^{⊤}\right]{\hat{\theta }}_{t}\}\\ \left[{\vec{\hat{\mu }}}_{t-1,t:2}^{⊤},{\hat{\mu }}_{t-1}X_{t}^{⊤}\right]{\hat{\theta }}_{t}\}\\ \vdots \\ \left[{\vec{\hat{\mu }}}_{2,t:2}^{⊤},{\hat{\mu }}_{2}X_{t}^{⊤}\right]{\hat{\theta }}_{t}\}\\ X_{t}\left[{\vec{\hat{\mu }}}_{t:2}^{⊤},X_{t}^{⊤}\right]{\hat{\theta }}_{t} \end{array}\right]=0.$$
3. Finally the first stage parameters $\theta_{1}$ are found using
  $${\mathbb{P}}_{N}\;\left[X_{1}\left\{{\hat{\mu }}_{2}\left(1+{\hat{\beta }}_{21}\right)+H_{20}^{⊤}{\hat{\beta }}_{22}+{\left[H_{21}^{⊤}{\hat{\gamma }}_{2}\right]}_{+}-X_{1}^{⊤}\theta_{1}\right\}\right]=0.$$

Next we go over a simpler T=3 scenario to highlight how the conditional mean models needed to be imputed are only functions of at most 2 missing outcomes. The three stage SSL *Q*-learning functions can be defined recursively as:

$$Q_{3}\left({\overset{ˇ}{H}}_{3},A_{3}\right)\equiv 𝔼\left[Y_{4}|{\overset{ˇ}{H}}_{3},A_{3}\right]\;Q_{2}\left({\overset{ˇ}{H}}_{2},A_{2}\right)\equiv 𝔼\left[Y_{3}+\underset{a_{3}}{\max}\;Q_{3}\left({\overset{ˇ}{H}}_{3},a_{3}\right)|{\overset{ˇ}{H}}_{2},A_{2}\right]\;Q_{1}\left({\overset{ˇ}{H}}_{1},A_{1}\right)\equiv 𝔼\left[Y_{2}+\underset{a_{2}}{\max}\;Q_{2}\left({\overset{ˇ}{H}}_{2},a_{2}\right)|{\overset{ˇ}{H}}_{1},A_{1}\right]$$

The corresponding working linear models for the *Q* functions with parameters $\theta_{t}={\left(\beta_{t}^{⊤},\gamma_{t}^{⊤}\right)}^{⊤}$, t=1, 2,3 are:

$$Q_{1}\left({\overset{ˇ}{H}}_{1},A_{1};\theta_{1}^{0}\right)={\overset{ˇ}{X}}_{1}^{⊤}\theta_{1}^{0}=H_{10}^{⊤}\beta_{1}^{0}+A_{1}\left(H_{11}^{⊤}\gamma_{1}^{0}\right),\;Q_{2}\left({\overset{ˇ}{H}}_{2},A_{2};\theta_{2}^{0}\right)={\overset{ˇ}{X}}_{2}^{⊤}\theta_{2}^{0}=Y_{2}\beta_{21}^{0}+H_{20}^{⊤}\beta_{22}^{0}+A_{2}\left(H_{21}^{⊤}\gamma_{2}^{0}\right),\;Q_{3}\left({\overset{ˇ}{H}}_{3},A_{3};\theta_{3}^{0}\right)={\overset{ˇ}{X}}_{3}^{⊤}\theta_{3}^{0}=Y_{3}\beta_{31}^{0}+Y_{2}\beta_{32}^{0}+H_{30}^{⊤}\beta_{33}^{0}+A_{3}\left(H_{31}^{⊤}\gamma_{3}^{0}\right).$$

The pseudo-outcomes for stages 1 and 2 under the linear *Q* function are

$${\bar{Y}}_{2i}^{\text{*}}=Y_{2i}\left(1+\beta_{21}\right)+H_{20i}^{⊤}\beta_{22}+{\left[H_{21i}^{⊤}\gamma_{2}\right]}_{+},\;{\bar{Y}}_{3i}^{\text{*}}=Y_{3i}\left(1+\beta_{31}\right)+Y_{2i}\beta_{32}+H_{30i}^{⊤}\beta_{33}+{\left[H_{31i}^{⊤}\gamma_{3}\right]}_{+}.$$

We expand the normal equations so that the outcomes $Y_{t},\;t=2, 3,4$ and their products are explicit:

$$𝔼\;\left[{\overset{ˇ}{X}}_{3}\left(Y_{4}-{\overset{ˇ}{X}}_{3}^{⊤}\theta_{3}\right)\right]=𝔼\;\left[\begin{array}{c} Y_{3}\left\{Y_{4}-\left(Y_{3},Y_{2},X_{3}^{⊤}\right)\theta_{3}\right\}\\ Y_{2}\left\{Y_{4}-\left(Y_{3},Y_{2},X_{3}^{⊤}\right)\theta_{3}\right\}\\ X_{3}\left\{Y_{4}-\left(Y_{3},Y_{2},X_{3}^{⊤}\right)\theta_{3}\right\} \end{array}\right]=0,\;\;𝔼\left[{\overset{ˇ}{X}}_{2}\left({\bar{Y}}_{3}^{\text{*}}-{\overset{ˇ}{X}}_{2}^{⊤}\theta_{2}\right)\right]=𝔼\;\left[\begin{array}{c} Y_{2}\left\{Y_{3}\left(1+\beta_{31}\right)+Y_{2}\beta_{32}+H_{30}^{⊤}\beta_{33}+{\left[H_{31}^{⊤}\gamma_{3}\right]}_{+}-\left(Y_{2},X_{2}^{⊤}\right)\theta_{2}\right\}\\ X_{2}\left\{Y_{3}\left(1+\beta_{31}\right)+Y_{2}\beta_{32}+H_{30}^{⊤}\beta_{33}+{\left[H_{31}^{⊤}\gamma_{3}\right]}_{+}-\left(Y_{2},X_{2}^{⊤}\right)\theta_{2}\right\} \end{array}\right]=0,\;𝔼\left[{\overset{ˇ}{X}}_{1}\left({\bar{Y}}_{2}^{\text{*}}-{\overset{ˇ}{X}}_{1}^{⊤}\theta_{1}\right)\right]=𝔼\left[X_{1}\left\{Y_{2}\left(1+\beta_{21}\right)+H_{20}^{⊤}\beta_{22}+{\left[H_{21}^{⊤}\gamma_{2}\right]}_{+}-X_{1}^{⊤}\theta_{1}\right\}\right]=0.$$

From the three-stage normal equations above, it is clear that all the imputations needed will be similar to the two-stage case: linear, pairwise products and squares of the missing outcomes. Next, we go over the generalization of our off-policy value function algorithm.

### SSL Value Function Estimation

We further extend the notation of our inverse probability weights by using $\omega_{0}\left({\overset{ˇ}{H}}_{0},A_{0};\Theta \right)\equiv 1$ for all ${\overset{ˇ}{H}}_{0},A_{0}$ and defining for t=1,…,T:

$$\omega_{t}\left({\overset{ˇ}{H}}_{t},A_{t},\Theta \right)\equiv \omega_{t-1}\left({\overset{ˇ}{H}}_{t-1},A_{t-1},\Theta \right)\left(\frac{d_{t}\left(H_{t};\theta_{t}\right)A_{t}}{\pi_{t}\left({\overset{ˇ}{H}}_{t};\xi_{t}\right)}+\frac{\left\{1-d_{t}\left(H_{t};\theta_{t}\right)\right\}\left\{1-A_{t}\right\}}{1-\pi_{t}\left({\overset{ˇ}{H}}_{t};\xi_{t}\right)}\right).$$

The corresponding doubly-robust supervised value function estimator is ${\hat{V}}_{{SUP}_{DR}}={\mathbb{P}}_{n}\left\{{𝒱}_{{SUP}_{DR}}(L;\hat{\Theta })\right\}$, where

$${𝒱}_{{\text{SUP}}_{\text{DR}}}(L;\hat{\Theta })=Q_{1}^{o}\left(H_{1};{\hat{\theta }}_{1}\right)\;+\sum_{t=1}^{T}\omega_{t}\left({\overset{ˇ}{H}}_{t},A_{t},\hat{\Theta }\right)\;\left[Y_{t+1}-\left\{Q_{t}^{o}\left({\overset{ˇ}{H}}_{t},{\hat{\theta }}_{t}\right)-Q_{t+1}^{o}\left(H_{t+1},{\hat{\theta }}_{t+1}\right)\right\}\right].$$

As with two time steps, we define $Q_{t-}^{o}\left(H_{t};\theta_{t}\right)=H_{t0}^{⊤}\beta_{tt}+{\left[H_{t,1}^{⊤}\gamma_{t}\right]}_{+}$ for t=1,…,T. Next, to simplify our next expression we let ${\hat{\beta }}_{T+1,\ell }\equiv 0$ for ℓ=1,…,T. Then we can re-write ${𝒱}_{{\text{SUP}}_{\text{DR}}}(L;\hat{\Theta })$ as:

$${𝒱}_{{\text{SUP}}_{\text{DR}}}(L;\hat{\Theta })=Q_{1}^{o}\left(H_{1};{\hat{\theta }}_{1}\right)+\;+\sum_{t=1}^{T}\omega_{t}\left({\overset{ˇ}{H}}_{t},A_{t},\hat{\Theta }\right)\;\left[\left(1+{\hat{\beta }}_{t+1,t}\right)Y_{t+1}+\sum_{\ell =1}^{t-1}Y_{\ell +1}\left({\hat{\beta }}_{t+1,\ell }-{\hat{\beta }}_{t,\ell }\right)\right]\;+\sum_{t=1}^{T}\omega_{t}\left({\overset{ˇ}{H}}_{t},A_{t},\hat{\Theta }\right)\;\left[Q_{t+1-}^{o}\left(H_{t+1};{\hat{\theta }}_{t+1}\right)-Q_{t-}^{o}\left(H_{t};{\hat{\theta }}_{t}\right)\right].$$

Note that as with the case when t=2 there are only three types of functions of the outcome to impute, hence we define similar conditional mean functions:

$$\mu_{2}^{v}(\vec{U})\equiv 𝔼\left[Y_{2}|\vec{U}\right],\;\mu_{\omega_{t}}^{v}(\vec{U})\equiv 𝔼\left[\omega_{t}\left({\overset{ˇ}{H}}_{t},A_{t};\bar{\Theta }\right)|\vec{U}\right],\;\mu_{t^{\prime }\omega_{t}}^{v}(\vec{U})\equiv 𝔼\left[Y_{t^{\prime }}\omega_{t}\left({\overset{ˇ}{H}}_{t},A_{t};\bar{\Theta }\right)|\vec{U}\right].$$

Next we give a summary of the algorithm for the semi-supervised doubly robust value function estimation:

#### Step I: Imputation

As in the two-stage setting, we fit flexible, weakly parametric, or non-parametric models to the labeled data to approximate the conditional mean functions defined above. We also denote the respective imputation models as $\left\{m_{2}(\vec{U}),m_{\omega_{t}}(\vec{U}),m_{t^{\prime }\omega_{t}}(\vec{U})\right\}$ and their fitted values as $\left\{{\overset{ˆ}{m}}_{2}(\vec{U}),{\hat{m}}_{\omega_{t}}(\vec{U}),{\hat{m}}_{t^{\prime }\omega_{t}}(\vec{U})\right\}$.

#### Step II: Refitting

Analogous to the two time step case our mis-specification and finite sample bias-corrected imputation models are $\left\{{\overset{‾}{\mu }}_{2}^{v}(\vec{U})=m_{2}(\vec{U})+\eta_{2}^{v},{\overset{‾}{\mu }}_{\omega_{t}}^{v}(\vec{U})=m_{\omega_{t}}(\vec{U})+\eta_{\omega_{t}}^{v},{\overset{‾}{\mu }}_{t^{\prime }\omega_{t}}^{v}(\vec{U})=m_{t^{\prime }\omega_{t}}(\vec{U})+\eta_{t^{\prime }\omega_{t}}^{v}\right\}$. The conditional mean functions terms are of the same 3 forms as the T=2 case, so we write similar constraints:

$$\;𝔼\;\left[\omega_{1}\left({\overset{ˇ}{H}}_{1},A_{1};\bar{\Theta }\right)\;\left\{Y_{2}-{\bar{\mu }}_{2}^{v}(\vec{U})\right\}\right]=0,\;\;𝔼\;\left[\left\{Q_{t+1-}^{o}\left(H_{t+1};{\bar{\theta }}_{t+1}\right)-Q_{t-}^{o}\left(H_{t};{\bar{\theta }}_{t}\right)\right\}\;\left\{\omega_{t}\left({\overset{ˇ}{H}}_{t},A_{t};\bar{\Theta }\right)-{\bar{\mu }}_{\omega_{t}}^{v}(\vec{U})\right\}\right]=0,t=2,\ldots ,T.\;\;\;𝔼\;\left[\omega_{t}\left({\overset{ˇ}{H}}_{t},A_{t};\bar{\Theta }\right)Y_{t^{\prime }}-{\bar{\mu }}_{t^{\prime }\omega_{t}}^{v}(\vec{U})\right]=0,t,t^{\prime }=2,\ldots ,T+1.$$

To estimate $\eta_{2}^{v}\;\eta_{\omega_{t}}^{v}$, and $\eta_{t^{\prime }\omega_{t}}^{v}$ under these constraints, we again employ cross-fitting using the following estimating equations

$$\;\sum_{k=1}^{K}\sum_{i\in {ℐ}_{k}}\omega_{1}\left({\overset{ˇ}{H}}_{1i},A_{1i};\hat{\Theta }\right)\;\left\{Y_{2}-{\hat{m}}_{2}^{\left(-k\right)}\left({\vec{U}}_{i}\right)-{\hat{\eta }}_{2}^{v}\right\}=0,\;\;\sum_{k=1}^{K}\sum_{i\in {ℐ}_{k}}\left\{Q_{t+1-}^{o}\left(H_{t+1};{\hat{\theta }}_{t+1}\right)-Q_{t-}^{o}\left(H_{t};{\hat{\theta }}_{t}\right)\right\}\;\left\{\omega_{t}\left({\overset{ˇ}{H}}_{ti},A_{ti};\hat{\Theta }\right)-{\hat{m}}_{\omega_{t}}^{\left(-k\right)}\left({\vec{U}}_{i}\right)-{\hat{\eta }}_{\omega_{t}}^{v}\right\}=0,\;\;\sum_{k=1}^{K}\sum_{i\in {ℐ}_{k}}\left\{\omega_{t}\left({\overset{ˇ}{H}}_{ti},A_{ti};\hat{\Theta }\right)Y_{t^{\prime }i}-{\hat{m}}_{t^{\prime }\omega_{t}}^{\left(-k\right)}\left({\vec{U}}_{i}\right)-{\hat{\eta }}_{t^{\prime }\omega_{t}}^{v}\right\}=0,$$

from the above we obtain ${\hat{\eta }}_{2}^{v}\;{\hat{\eta }}_{\omega_{t}}^{v}$, and ${\hat{\eta }}_{t^{\prime }\omega_{t}}^{v}$ and use these to construct our imputation functions:

$${\hat{\mu }}_{2}^{v}(\vec{U})=K^{-1}\sum_{k=1}^{K}{\hat{m}}_{2}^{\left(-k\right)}(\vec{U})+{\hat{\eta }}_{2}^{v},\;{\hat{\mu }}_{\omega_{t}}^{v}(\vec{U})=K^{-1}\sum_{k=1}^{K}{\hat{m}}_{\omega_{t}}(\vec{U})+{\hat{\eta }}_{\omega_{t}}^{v},\;\text{for}\;t=2,\ldots ,T,$$

and

$${\hat{\mu }}_{t^{\prime }\omega_{t}}^{v}(\vec{U})=K^{-1}\sum_{k=1}^{K}{\hat{m}}_{t^{\prime }\omega_{t}}^{\left(-k\right)}(\vec{U})+{\hat{\eta }}_{t^{\prime }\omega_{t}}^{v},\;\text{for}\;t,t^{\prime }=2,\ldots ,T+1.$$

#### Step III: Semi-supervised augmented value function estimator.

Finally, our semi-supervised augmented estimator of the value of the policy $\overset{‾}{V}$ is

$${\hat{V}}_{{\text{SSL}}_{\text{DR}}}={\mathbb{P}}_{N}\left\{{𝒱}_{{\text{SSL}}_{\text{DR}}}(\vec{U};\hat{\Theta },\hat{\mu })\right\},$$

where ${\hat{𝒱}}_{{\text{SSL}}_{\text{DR}}}(\vec{U})$ is defined as:

$${𝒱}_{{\text{SSL}}_{\text{DR}}}(\vec{U};\hat{\Theta },\hat{\mu })=Q_{1}^{o}\left(H_{1};{\hat{\theta }}_{1}\right)+\omega_{1}\left(H_{1},A_{1},\hat{\Theta }\right)\;\left[\left(1+{\hat{\beta }}_{21}\right){\hat{\mu }}_{2}^{v}(\vec{U})+Q_{2-}^{o}\left(H_{2};{\hat{\theta }}_{2}\right)-Q_{1}^{o}\left(H_{1};{\hat{\theta }}_{1}\right)\right]\;+\overset{T}{\underset{t=2}{\Sigma }}\left(1+{\hat{\beta }}_{t+1,t}\right){\hat{\mu }}_{t+1,\omega_{t}}^{v}(\vec{U})+\overset{T}{\underset{t=2}{\Sigma }}\overset{t-1}{\underset{\ell =1}{\Sigma }}{\hat{\mu }}_{\ell +1,\omega_{t}}^{v}(\vec{U})\left({\hat{\beta }}_{t+1,\ell }-{\hat{\beta }}_{t,\ell }\right)\;+\overset{T}{\underset{t=2}{\Sigma }}{\hat{\mu }}_{\omega_{t}}^{v}(\vec{U})\;\left[Q_{t+1-}^{o}\left(H_{t+1};{\hat{\theta }}_{t+1}\right)-Q_{t-}^{o}\left(H_{t};{\hat{\theta }}_{t}\right)\right].$$

Next, we discuss whether the proposed method would work well for an arbitrary finite time horizon and where it might break down. Theoretically, the semi-supervised *Q* function and value function estimation will work as long as *T* is finite. However, as it is well known, we may face instability in our estimates during implementation. In the context of supervised doubly robust estimators for policy evaluation methods from (Jiang and Li, 2016b; Thomas and Brunskill, 2016a; Kallus and Uehara, 2020a) can be evaluated at the estimated optimal DTR to obtain the optimal value function. However, for large *T*, even these supervised estimators can be unstable as they depend on the products of the *T* inverse propensity weights. These become highly variable as *T* increases because they usually have nested products of such weights (Levine et al., 2020).

Regarding our theoretical results, as mentioned in the conclusion, there is nothing in the proofs explicitly requiring the timeline to be limited to two stages. We hypothesize that we can generalize the results to T>2 using induction, as we have already proven the results for the first couple of stages. We chose to leave the theoretical results of this generalization for future work, as we believe no new or particularly interesting theoretical methodology is required for this extension, and the notation and results are already cumbersome in this more simple setting.

In the context of our semi-supervised method, as *T* grows, our Q- and value function estimators naturally require more terms to impute. We expect that imputing a higher number of terms will increase the variance. However, an advantage of our approach is that the conditional means to be imputed are, at most, products of two missing functions: outcomes and propensity scores, so their complexity does not increase with *T*. Most of the variability would come from the higher-order propensity scores, as is the case with the supervised methods previously mentioned.

## Appendix C. Simulation Results for Alternative Settings

In this section we provide additional results for data generating scenarios described in Section 6. Tables C.1 and C.1 contain results for estimation of *Q* function parameters for the EHR simulation setting for small and large sample sizes respectively. Table C.3 contains the complete parameter results for the continuous data generating setting for both small and large samples.

**Table C.1:** Bias, empirical standard error (ESE) of the supervised and the SSL estimators with either random forest imputation or basis expansion imputation strategies for $\bar{\theta }$ when (a) n=135 and N=1272 under the EHR simulation setting. For the SSL estimators, we also obtain the average of the estimated standard errors (ASE) as well as the empirical coverage probabilities (CovP) of the 95% confidence intervals.

(a) n=135 and N=1272												
	Supervised		Semi-Supervised									
			Random Forests					Basis Expansion				
Parameter	Bias	ESE	Bias	ESE	ASE	CovP	RE	Bias	ESE	ASE	CovP	RE
		
$\beta_{11}=1.2$	0.05	0.09	0.03	0.06	0.05	0.88	1.65	0.03	0.06	0.05	0.89	1.60
$\beta_{12}=0$	0.00	0.06	0.00	0.04	0.04	0.90	1.57	0.00	0.04	0.04	0.91	1.62
$\beta_{13}=-0.4$	0	0.07	−0.01	0.05	0.04	0.92	1.53	0	0.05	0.05	0.93	1.56
$\beta_{14}=-0.3$	0.00	0.07	−0.01	0.04	0.04	0.93	1.67	0	0.04	0.04	0.93	1.64
$\beta_{15}=0$	0.00	0.08	0.00	0.04	0.04	0.93	1.69	0.00	0.04	0.04	0.92	1.69
$\beta_{16}=0$	0	0.07	0.00	0.04	0.04	0.93	1.67	0.00	0.04	0.04	0.93	1.74
$\beta_{17}=0$	0.00	0.08	0.00	0.05	0.04	0.92	1.62	0.00	0.05	0.04	0.92	1.62
$\gamma_{11}=0.1$	−0.01	0.14	0.00	0.09	0.08	0.91	1.55	0	0.09	0.07	0.89	1.55
$\gamma_{12}=0$	−0.01	0.09	−0.01	0.06	0.05	0.92	1.53	−0.01	0.06	0.06	0.93	1.51
$\gamma_{13}=0$	0	0.08	0	0.05	0.05	0.93	1.58	0	0.05	0.05	0.94	1.58
$\gamma_{14}=0$	0	0.08	0.00	0.05	0.05	0.93	1.58	0	0.05	0.05	0.93	1.58
$\gamma_{15}=0$	0.00	0.09	0.00	0.05	0.05	0.92	1.59	0	0.05	0.05	0.95	1.65
$\gamma_{16}=-0.1$	0	0.09	0	0.06	0.05	0.92	1.52	0	0.06	0.05	0.93	1.49
$\beta_{21}=0.1$	0.00	0.10	−0.01	0.15	0.13	0.91	0.71	0	0.14	0.13	0.93	0.75
$\beta_{22}=0.6$	0	0.13	0.01	0.11	0.10	0.91	1.16	0	0.11	0.11	0.94	1.18
$\beta_{23}=0$	0.00	0.06	0.00	0.04	0.04	0.93	1.44	0.00	0.04	0.04	0.93	1.47
$\beta_{24}=-0.2$	0.00	0.06	0	0.05	0.04	0.89	1.16	0	0.05	0.05	0.93	1.20
$\beta_{25}=-0.2$	0.00	0.05	0	0.05	0.04	0.90	1.13	0	0.04	0.04	0.92	1.18
$\beta_{26}=0$	0.00	0.04	0.00	0.02	0.02	0.94	1.50	0.00	0.02	0.02	0.94	1.50
$\beta_{27}=0$	0.00	0.04	0.00	0.03	0.02	0.94	1.52	0.00	0.02	0.02	0.94	1.58
$\beta_{28}=0$	0.00	0.05	0.00	0.04	0.03	0.92	1.49	0.00	0.04	0.03	0.92	1.49
$\beta_{29}=0$	0	0.12	0.00	0.08	0.07	0.91	1.49	0.00	0.08	0.08	0.93	1.52
$\beta_{210}=-0.2$	0.00	0.11	0	0.07	0.07	0.94	1.54	0.00	0.07	0.07	0.94	1.57
$\beta_{211}=-0.1$	0.01	0.11	0.00	0.07	0.07	0.94	1.54	0.00	0.07	0.07	0.93	1.56
$\gamma_{21}=0.1$	0.01	0.16	0.01	0.11	0.10	0.92	1.47	0.01	0.11	0.10	0.94	1.51
$\gamma_{22}=0$	0.00	0.08	0.00	0.06	0.05	0.94	1.47	0.00	0.06	0.06	0.93	1.50
$\gamma_{23}=0$	0	0.08	0.00	0.06	0.05	0.94	1.45	0.00	0.05	0.05	0.94	1.48
$\gamma_{24}=0$	0	0.07	0.00	0.05	0.05	0.93	1.43	0.00	0.05	0.05	0.94	1.46
$\gamma_{25}=0$	0	0.07	0	0.05	0.05	0.94	1.48	0	0.05	0.05	0.94	1.48
$\gamma_{26}=0$	0	0.18	0	0.12	0.11	0.92	1.45	0	0.12	0.11	0.94	1.52
$\gamma_{27}=-0.2$	−0.01	0.16	−0.01	0.11	0.10	0.93	1.47	−0.01	0.11	0.10	0.94	1.48
$\gamma_{28}=-0.1$	−0.01	0.15	−0.01	0.10	0.10	0.94	1.54	−0.01	0.10	0.10	0.94	1.57

**Table C.2:** Bias, empirical standard error (ESE) of the supervised and the SSL estimators with either random forest imputation or basis expansion imputation strategies for $\bar{\theta }$ when (b) n=500 and N=10,000 under the EHR simulation setting. For the SSL estimators, we also obtain the average of the estimated standard errors (ASE) as well as the empirical coverage probabilities (CovP) of the 95% confidence intervals.

(b) n=500 and N=10,000												
	Supervised		Semi-Supervised									
			Random Forests					Basis Expansion				
Parameter	Bias	ESE	Bias	ESE	ASE	CovP	RE	Bias	ESE	ASE	CovP	RE
		
$\beta_{11}=1.2$	0.01	0.05	0.00	0.02	0.02	0.91	2.09	0.00	0.02	0.02	0.92	2.00
$\beta_{12}=0$	0.00	0.03	0.00	0.01	0.01	0.91	2.07	0.00	0.01	0.01	0.92	2.07
$\beta_{13}=-0.4$	0.00	0.04	0	0.02	0.02	0.92	2.05	0	0.02	0.02	0.92	2.05
$\beta_{14}=-0.3$	0	0.04	0	0.02	0.01	0.92	2.06	0	0.02	0.02	0.92	2.06
$\beta_{15}=0$	0.00	0.04	0	0.02	0.02	0.94	2.18	0	0.02	0.02	0.94	2.06
$\beta_{16}=0$	0	0.04	0.00	0.02	0.02	0.94	2.18	0.00	0.02	0.02	0.94	2.18
$\beta_{17}=0$	0.00	0.04	0.00	0.02	0.02	0.93	2.06	0.00	0.02	0.02	0.94	2.06
$\gamma_{11}=0.1$	0	0.07	0	0.03	0.03	0.91	2.00	0	0.03	0.03	0.91	2.00
$\gamma_{12}=0$	−0.01	0.05	0	0.02	0.02	0.90	2.00	0	0.02	0.02	0.89	2.00
$\gamma_{13}=0$	0.00	0.04	0.00	0.02	0.02	0.92	2.00	0.00	0.02	0.02	0.91	1.90
$\gamma_{14}=0$	0	0.04	0.00	0.02	0.02	0.94	2.00	0.00	0.02	0.02	0.94	1.90
$\gamma_{15}=0$	0.00	0.04	0.00	0.02	0.02	0.94	2.16	0.00	0.02	0.02	0.94	2.05
$\gamma_{16}=-0.1$	0	0.04	0	0.02	0.02	0.93	2.05	0	0.02	0.02	0.92	1.95
$\beta_{21}=0.1$	0.00	0.05	0.00	0.04	0.04	0.95	1.16	0.00	0.04	0.05	0.96	1.13
$\beta_{22}=0.6$	0	0.07	0	0.04	0.04	0.95	1.74	0	0.04	0.04	0.96	1.69
$\beta_{23}=0$	0.00	0.03	0.00	0.01	0.01	0.94	1.87	0.00	0.01	0.01	0.94	1.87
$\beta_{24}=-0.2$	0.00	0.03	0.00	0.02	0.02	0.94	1.71	0.00	0.02	0.02	0.95	1.71
$\beta_{25}=-0.2$	0.00	0.02	0	0.01	0.01	0.94	1.60	0	0.01	0.01	0.95	1.60
$\beta_{26}=0$	0.00	0.02	0.00	0.01	0.01	0.92	1.90	0.00	0.01	0.01	0.93	1.90
$\beta_{27}=0$	0.00	0.02	0.00	0.01	0.01	0.94	1.89	0.00	0.01	0.01	0.94	1.89
$\beta_{28}=0$	0.00	0.03	0.00	0.01	0.01	0.94	1.92	0.00	0.01	0.01	0.94	1.92
$\beta_{29}=0$	0.00	0.06	0.00	0.03	0.03	0.92	1.94	0.00	0.03	0.03	0.93	1.88
$\beta_{210}=-0.2$	0	0.05	0	0.03	0.03	0.94	2.00	0.00	0.03	0.03	0.94	2.00
$\beta_{211}=-0.1$	0.00	0.06	0.00	0.03	0.03	0.94	2.00	0.00	0.03	0.03	0.94	2.00
$\gamma_{21}=0.1$	0	0.08	0.00	0.04	0.04	0.94	1.98	0.00	0.04	0.04	0.94	1.98
$\gamma_{22}=0$	0.00	0.04	0.00	0.02	0.02	0.93	1.95	0.00	0.02	0.02	0.93	1.86
$\gamma_{23}=0$	0	0.04	0	0.02	0.02	0.94	1.81	0	0.02	0.02	0.93	1.90
$\gamma_{24}=0$	0	0.03	0.00	0.02	0.02	0.94	1.83	0.00	0.02	0.02	0.95	1.83
$\gamma_{25}=0$	0	0.04	0	0.02	0.02	0.94	1.84	0	0.02	0.02	0.94	1.84
$\gamma_{26}=0$	−0.01	0.09	0	0.04	0.04	0.93	2.00	0	0.04	0.04	0.93	2.00
$\gamma_{27}=-0.2$	0.01	0.08	0.00	0.04	0.04	0.94	1.98	0.00	0.04	0.04	0.94	1.98
$\gamma_{28}=-0.1$	0.00	0.08	0.00	0.04	0.04	0.94	1.95	0.00	0.04	0.04	0.94	1.95

## Appendix D. Proof of Main Results

### D.1 Semi-Supervised Q-Learning Asymptotics

In this section we first show the proofs for the theoretical results on the generalized semi-supervised Q-learning shown in Section 5.

**Table C.3:** Bias, empirical standard error (ESE) of the supervised and the SSL estimators with either random forest imputation or basis expansion imputation strategies for $\bar{\theta }$ when (a) n=135 and N=1272 and (b) n=500 and N=10,000 under the continuous outcome simulation setting. For the SSL estimators, we also obtain the average of the estimated standard errors (ASE) as well as the empirical coverage probabilities (CovP) of the 95% confidence intervals.

(a) n=135 and N=1272												
Parameter	Supervised		Semi-Supervised									
	Bias	ESE	Random Forests					Basis Expansion				
			Bias	ESE	ASE	CovP	RE	Bias	ESE	ASE	CovP	RE
		
*β*_11_=4.9	0.04	0.34	0.01	0.22	0.18	0.91	1.58	0.01	0.20	0.17	0.90	1.70
*β*_12_=1.1	−0.03	0.42	0.00	0.26	0.24	0.94	1.61	0.01	0.25	0.23	0.92	1.68
*γ*_11_=1.4	−0.03	0.41	0.00	0.26	0.24	0.93	1.57	0.00	0.24	0.23	0.93	1.68
*γ*_12_=−2.6	0.04	0.58	−0.01	0.36	0.34	0.94	1.61	−0.02	0.35	0.31	0.90	1.69
*β*_21_=0.1	0.00	0.10	0.00	0.13	0.12	0.94	0.82	0.00	0.16	0.17	0.94	0.64
*β*_22_=3	0.00	0.33	0.00	0.24	0.23	0.93	1.39	0	0.26	0.25	0.93	1.30
*β*_23_=0	−0.01	0.34	−0.01	0.24	0.22	0.93	1.43	−0.01	0.24	0.24	0.94	1.39
*β*_24_=0.1	0	0.43	0	0.29	0.28	0.94	1.49	0	0.30	0.29	0.94	1.46
*β*_25_=−0.5	0.01	0.15	0	0.09	0.09	0.93	1.62	0.00	0.09	0.09	0.93	1.71
*β*_26_=−0.4	0.03	0.48	0.01	0.37	0.35	0.93	1.29	0.01	0.41	0.40	0.94	1.16
*γ*_21_=0.8	0.00	0.34	0.01	0.21	0.20	0.93	1.61	0.00	0.20	0.19	0.94	1.71
*γ*_22_=0.2	−0.02	0.45	−0.01	0.28	0.28	0.95	1.60	−0.01	0.27	0.26	0.94	1.70
*γ*_23_=0.5	0	0.18	0.01	0.11	0.11	0.94	1.59	0.00	0.11	0.11	0.94	1.68

(b) n=500 and N=10,000												
	Supervised		Semi-Supervised									
			Random Forests					Basis Expansion				
Parameter	Bias	ESE	Bias	ESE	ASE	CovP	RE	Bias	ESE	ASE	CovP	RE
		
*β*_11_=4.9	0.00	0.17	0	0.10	0.09	0.91	1.72	0	0.10	0.08	0.92	1.79
*β*_12_=1.1	0	0.22	0.00	0.12	0.11	0.93	1.80	0.00	0.12	0.11	0.93	1.86
*γ*_11_=1.4	0.01	0.22	0.01	0.12	0.11	0.92	1.76	0.01	0.12	0.11	0.92	1.80
*γ*_12_=−2.6	0	0.29	0	0.17	0.16	0.93	1.73	−0.01	0.16	0.15	0.93	1.80
*β*_21_=0.1	−0.01	0.05	0	0.05	0.05	0.94	1.06	0	0.07	0.08	0.95	0.74
*β*_22_=3	0.00	0.17	0.00	0.11	0.10	0.93	1.60	0.00	0.12	0.11	0.94	1.45
*β*_23_=0	0.00	0.17	0.00	0.10	0.10	0.95	1.66	0.00	0.11	0.11	0.95	1.54
*β*_24_=0.1	0.02	0.23	0.01	0.13	0.12	0.94	1.77	0.01	0.14	0.13	0.94	1.68
*β*_25_=−0.5	0.00	0.07	0.00	0.04	0.04	0.93	1.74	0.00	0.04	0.04	0.94	1.78
*β*_26_=−0.4	−0.01	0.25	−0.01	0.17	0.15	0.93	1.51	−0.01	0.19	0.18	0.94	1.31
*γ*_21_=0.8	0.00	0.17	0.00	0.10	0.09	0.93	1.80	0.00	0.09	0.09	0.93	1.86
*γ*_22_=0.2	−0.01	0.23	0	0.13	0.12	0.93	1.81	0	0.13	0.12	0.94	1.83
*γ*_23_=0.5	0.00	0.09	0.00	0.05	0.05	0.94	1.78	0.00	0.05	0.05	0.95	1.81

#### D.1.1 Proofs of Theoretical Results for *Q*-Learning in Section 5

We first define $\theta_{2-}\equiv {\left(\beta_{22}^{⊤},\gamma_{2}^{⊤}\right)}^{⊤}$, and ${\overset{ˆ}{\Delta }}_{s}^{\left(-k\right)}(\vec{U})\equiv {\hat{m}}_{s}^{\left(-k\right)}(\vec{U})-m_{s}(\vec{U}),s\in \{2,3,22,23\}$, and note that from Assumptions 1, 2 & 3 it follows that:

(8)
$$\sum_{k=1}^{K}\underset{\vec{U}}{\sup}\left|{\hat{\text{Δ}}}_{2t}^{\left(-k\right)}(\vec{U})\right|=o_{\mathbb{P}}(1)\;\text{for}\;t=2,3,\;\sum_{k=1}^{K}\underset{\vec{X},\vec{U}}{\sup}\left\|\vec{X}{\hat{\text{Δ}}}_{2}^{\left(-k\right)}(\vec{U})\right\|=o_{\mathbb{P}}(1),\;\sum_{k=1}^{K}\underset{,X_{2},\vec{U}}{\sup}\left\|X_{2}{\hat{\text{Δ}}}_{3}^{\left(-k\right)}(\vec{U})\right\|=o_{\mathbb{P}}(1),$$

Next we remind that, to ensure the validity of the SSL algorithm from the refitted imputation model, the final imputation models for $\left\{Y_{t},Y_{2t},t=2,3\right\}$, denoted by $\left\{{\overset{‾}{\mu }}_{t}(\vec{U}),{\overset{‾}{\mu }}_{2t},t=2,3\right\}$, need to satisfy the constraints shown in Section 3.2:

(9)
$$\;𝔼\left[\vec{X}\left\{Y_{2}-{\bar{\mu }}_{2}(\vec{U})\right\}\right]=0,\;𝔼\left\{Y_{2}^{2}-{\bar{\mu }}_{22}(\vec{U})\right\}=0,\;𝔼\left[X_{2}\left\{Y_{3}-{\bar{\mu }}_{3}(\vec{U})\right\}\right]=0\;,𝔼\left\{Y_{2}Y_{3}-{\bar{\mu }}_{23}(\vec{U})\right\}=0.$$

where $\vec{X}={\left(1,X_{1}^{⊤},X_{2}^{⊤}\right)}^{⊤}$.

**Proof** [Proof of Theorem 2]

Recall the estimating equation for stage 2 regression in Section 3.2 is

$${\mathbb{P}}_{N}\left[\begin{array}{c} {\hat{\mu }}_{23}(\vec{U})-{\hat{\beta }}_{21}{\hat{\mu }}_{22}(\vec{U})-{\hat{\mu }}_{2}(\vec{U})X_{2}^{⊤}{\hat{\theta }}_{2-}\\ X_{2}\left\{{\hat{\mu }}_{3}(\vec{U})-{\hat{\beta }}_{21}{\hat{\mu }}_{2}(\vec{U})-X_{2}^{⊤}{\hat{\theta }}_{2-}\right\} \end{array}\right]=0.$$

Centering the above at ${\bar{\theta }}_{2}$ we get

(10)
$${\mathbb{P}}_{N}\left[\begin{array}{c} {\hat{\mu }}_{22}(\vec{U}),{\hat{\mu }}_{2}(\vec{U})X_{2}^{⊤}\\ X_{2}{\hat{\mu }}_{2}(\vec{U}),X_{2}X_{2}^{⊤} \end{array}\right]\left({\hat{\theta }}_{2}-{\bar{\theta }}_{2}\right)={\mathbb{P}}_{N}\left[\begin{array}{c} {\hat{\mu }}_{23}(\vec{U})-{\bar{\beta }}_{21}{\hat{\mu }}_{22}(\vec{U})-{\hat{\mu }}_{2}(\vec{U})X_{2}^{⊤}{\bar{\theta }}_{2-}\\ X_{2}\left\{{\hat{\mu }}_{3}(\vec{U})-{\bar{\beta }}_{21}{\hat{\mu }}_{2}(\vec{U})-X_{2}^{⊤}{\bar{\theta }}_{2-}\right\} \end{array}\right].$$

Define

$${ℛ}_{𝒰}={\mathbb{P}}_{N}\left[\begin{array}{c} {\bar{\mu }}_{23}(\vec{U})-{\bar{\beta }}_{21}{\bar{\mu }}_{22}(\vec{U})-{\bar{\mu }}_{2}(\vec{U})X_{2}^{⊤}{\bar{\theta }}_{2-}\\ X_{2}\left\{{\bar{\mu }}_{3}(\vec{U})-{\bar{\beta }}_{21}{\bar{\mu }}_{2}(\vec{U})-X_{2}^{⊤}{\bar{\theta }}_{2-}\right\} \end{array}\right],$$

$${\hat{ℛ}}_{𝕊}^{\left(K\right)}={\mathbb{P}}_{N}\left[\begin{array}{c} \left\{{\hat{\mu }}_{23}(\vec{U})-{\bar{\mu }}_{23}(\vec{U})\right\}-{\bar{\beta }}_{21}\left\{{\hat{\mu }}_{22}(\vec{U})-{\bar{\mu }}_{22}(\vec{U})\right\}-\left\{{\hat{\mu }}_{2}(\vec{U})-{\bar{\mu }}_{2}(\vec{U})\right\}X_{2}^{⊤}{\bar{\theta }}_{2-}\\ X_{2}\left\{{\hat{\mu }}_{3}(\vec{U})-{\bar{\mu }}_{3}(\vec{U})\right\}-{\bar{\beta }}_{21}X_{2}\left\{{\hat{\mu }}_{2}(\vec{U})-{\bar{\mu }}_{2}(\vec{U})\right\} \end{array}\right],$$

$${\text{Γ}}_{𝒰}={\mathbb{P}}_{N}\left[\begin{array}{cc} {\bar{\mu }}_{22}(\vec{U}) & {\bar{\mu }}_{2}(\vec{U})X_{2}^{⊤}\\ {\bar{\mu }}_{2}(\vec{U})X_{2} & X_{2}X_{2}^{⊤} \end{array}\right],$$

$${\hat{\text{Γ}}}_{𝕊}^{\left(K\right)}={\mathbb{P}}_{N}\left[\begin{array}{cc} {\hat{\mu }}_{22}(\vec{U})-{\bar{\mu }}_{22}(\vec{U}) & \left\{{\hat{\mu }}_{2}(\vec{U})-{\bar{\mu }}_{2}(\vec{U})\right\}X_{2}^{⊤}\\ \left\{{\hat{\mu }}_{2}(\vec{U})-{\bar{\mu }}_{2}(\vec{U})\right\}X_{2} & 0 \end{array}\right],$$

with these we can re-write equation (10) as $\left(\Gamma_{𝒰}+{\overset{ˆ}{\Gamma }}_{𝕊}^{\left(K\right)}\right)\left({\hat{\theta }}_{2}-{\bar{\theta }}_{2}\right)={ℛ}_{𝒰}+{\hat{ℛ}}_{𝕊}^{\left(K\right)}$. We next deal with each term.

(I) We first consider ${\hat{ℛ}}_{𝕊}^{\left(K\right)}$, let

$${\hat{𝒮}}_{𝕊}^{\eta }={\mathbb{P}}_{N}\left[\begin{array}{c} \left({\hat{\eta }}_{23}-\eta_{23}\right)-{\hat{\beta }}_{21}\left({\hat{\eta }}_{22}-\eta_{22}\right)-{\left({\hat{\eta }}_{2}-\eta_{2}\right)}^{⊤}X_{2}X_{2}^{⊤}{\bar{\theta }}_{2-}\\ X_{2}X_{2}^{⊤}\left\{\left({\hat{\eta }}_{3}-\eta_{3}\right)-{\hat{\beta }}_{21}\left({\hat{\eta }}_{2}-\eta_{2}\right)\right\} \end{array}\right]$$

$${\hat{𝒮}}_{𝕊}^{\left(K\right)}=\frac{1}{K}\sum_{k=1}^{K}{\mathbb{P}}_{N}\left[\begin{array}{c} {\hat{\text{Δ}}}_{23}^{\left(-k\right)}(\vec{U})-{\hat{\beta }}_{21}{\hat{\text{Δ}}}_{22}^{\left(-k\right)}(\vec{U})-{\hat{\text{Δ}}}_{2}^{\left(-k\right)}(\vec{U})X_{2}^{⊤}{\bar{\theta }}_{2-}\\ X_{2}\left\{{\hat{\text{Δ}}}_{3}^{\left(-k\right)}(\vec{U})-{\hat{\beta }}_{21}{\hat{\text{Δ}}}_{2}^{\left(-k\right)}(\vec{U})\right\} \end{array}\right]$$

$${\bar{𝒮}}_{k}={𝔼}_{ℒ}\left[\begin{array}{c} {\hat{\text{Δ}}}_{23}^{\left(-k\right)}(\vec{U})-{\hat{\beta }}_{21}{\hat{\text{Δ}}}_{22}^{\left(-k\right)}(\vec{U})-{\hat{\text{Δ}}}_{2}^{\left(-k\right)}(\vec{U})X_{2}^{⊤}{\bar{\theta }}_{2-}\\ X_{2}\left\{{\hat{\text{Δ}}}_{3}^{\left(-k\right)}(\vec{U})-{\hat{\beta }}_{21}{\hat{\text{Δ}}}_{2}^{\left(-k\right)}(\vec{U})\right\} \end{array}\right]\;\text{for}\;k\in \{1,\ldots ,K\}.$$

From (3) it follows that ${\hat{ℛ}}_{𝕊}^{\left(K\right)}={\hat{𝒮}}_{𝕊}^{\eta }+{\hat{𝒮}}_{𝕊}^{\left(K\right)}$. Next using (8), Assumption 2, and Lemma 15 it follows that ${\hat{𝒮}}_{𝕊}^{\left(K\right)}=\frac{1}{K}\sum_{k}{\bar{𝒮}}_{k}+O_{\mathbb{P}}\left(N^{-\frac{1}{2}}\right)$, which lets us write ${\hat{ℛ}}_{𝕊}^{\left(K\right)}={\hat{𝒮}}_{𝕊}^{\eta }+\frac{1}{K}\sum_{k}{\bar{𝒮}}_{k}+O_{\mathbb{P}}\left(N^{-\frac{1}{2}}\right)$.

Now consider ${\hat{𝒮}}_{𝕊}^{\eta }$, note that by the central limit theorem (CLT) ${\mathbb{P}}_{n}X_{2}X_{2}=𝔼X_{2}X_{2}+O_{\mathbb{P}}\left(n^{-\frac{1}{2}}\right)$. Thus using this, Slutsky’s theorem and Assumption 1

$${\left({\mathbb{P}}_{n}X_{2}X_{2}\right)}^{-1}\left({\mathbb{P}}_{N}X_{2}X_{2}\right)=I+O_{\mathbb{P}}\left(n^{-\frac{1}{2}}\right),$$

then using (9), (3) and Assumption 2 we can write

$$\;{\mathbb{P}}_{N}\left\{{\left({\hat{\eta }}_{2}-\eta_{2}\right)}^{⊤}X_{2}X_{2}^{⊤}{\bar{\theta }}_{2-}\right\}\;=[{\left({\mathbb{P}}_{n}X_{2}X_{2}^{⊤}\right)}^{-1}\frac{1}{n}\sum_{k=1}^{K}\sum_{i\in {ℐ}_{k}}X_{2i}{\left\{Y_{2i}-{\bar{\mu }}_{2}\left({\vec{U}}_{i}\right)+m_{2}\left({\vec{U}}_{i}\right)-m_{2}^{\left(-k\right)}\left({\vec{U}}_{i}\right)\right\}}^{⊤}{\mathbb{P}}_{N}\left(X_{2}X_{2}^{⊤}\right)\theta_{2-}\;=\left[{\mathbb{P}}_{n}X_{2}^{⊤}\left\{Y_{2}-{\bar{\mu }}_{2}(\vec{U})\right\}+\frac{1}{n}\sum_{k=1}^{K}\sum_{i\in {ℐ}_{k}}X_{2i}^{⊤}{\hat{\text{Δ}}}_{2}^{\left(-k\right)}\left({\vec{U}}_{i}\right)\right]{\left({\mathbb{P}}_{n}X_{2}X_{2}^{⊤}\right)}^{-1}{\mathbb{P}}_{N}\left(X_{2}X_{2}^{⊤}\right)\theta_{2-}\;={\mathbb{P}}_{n}X_{2}\theta_{2-}^{⊤}\left\{Y_{2}-{\bar{\mu }}_{2}(\vec{U})\right\}+\frac{1}{n}\sum_{k=1}^{K}\sum_{i\in {ℐ}_{k}}X_{2i}^{⊤}\theta_{2-}{\hat{\text{Δ}}}_{2}^{\left(-k\right)}\left({\vec{U}}_{i}\right)\;+O_{\mathbb{P}}\left(n^{-\frac{1}{2}}\right)\left[{\mathbb{P}}_{n}X_{2}\theta_{2-}^{⊤}\left\{Y_{2}-{\bar{\mu }}_{2}(\vec{U})\right\}+\frac{1}{n}\sum_{k=1}^{K}\sum_{i\in {ℐ}_{k}}X_{2i}^{⊤}\theta_{2-}{\hat{\text{Δ}}}_{2}^{\left(-k\right)}\left({\vec{U}}_{i}\right)\right]\;={\mathbb{P}}_{n}X_{2}\theta_{2-}^{⊤}\left\{Y_{2}-{\bar{\mu }}_{2}(\vec{U})\right\}+\frac{1}{n}\sum_{k=1}^{K}\sum_{i\in {ℐ}_{k}}X_{2i}^{⊤}\theta_{2-}{\hat{\text{Δ}}}_{2}^{\left(-k\right)}\left({\vec{U}}_{i}\right)+O_{\mathbb{P}}\left(n^{-1}\right)+O_{\mathbb{P}}\left(n^{-\frac{1}{2}}\right)o_{\mathbb{P}}(1).$$

Analogous derivations for all terms in ${\hat{𝒮}}_{𝕊}^{\eta }$ gives us

$${\hat{𝒮}}_{𝕊}^{\eta }={𝕋}_{ℒ}-{𝕋}_{ℒ}^{\left(K\right)}+O_{\mathbb{P}}\left(n^{-1}\right)+O_{\mathbb{P}}\left(n^{-\frac{1}{2}}\right)o_{\mathbb{P}}(1),$$

where

$${𝕋}_{ℒ}={\mathbb{P}}_{n}\left[\begin{array}{c} \left\{Y_{2}Y_{3}-{\bar{\mu }}_{23}(\vec{U})\right\}-{\bar{\beta }}_{21}\left\{Y_{2}^{2}-{\bar{\mu }}_{22}(\vec{U})\right\}-\left\{Y_{2}-{\bar{\mu }}_{2}(\vec{U})\right\}X_{2}^{⊤}{\bar{\theta }}_{2-}\\ X_{2}\left\{Y_{3}-{\bar{\mu }}_{3}(\vec{U})\right\}-{\bar{\beta }}_{21}X_{2}\left\{Y_{2}-{\bar{\mu }}_{2}(\vec{U})\right\} \end{array}\right],$$

$${𝕋}_{ℒ}^{\left(K\right)}=\frac{1}{n}\sum_{k=1}^{K}\sum_{i\in {ℐ}_{k}}\left[\begin{array}{c} {\hat{\text{Δ}}}_{23}^{\left(-k\right)}\left({\vec{U}}_{i}\right)-{\bar{\beta }}_{21}{\hat{\text{Δ}}}_{22}^{\left(-k\right)}\left({\vec{U}}_{i}\right)-{\hat{\text{Δ}}}_{2}^{\left(-k\right)}\left({\vec{U}}_{i}\right)X_{2i}^{⊤}{\bar{\theta }}_{2-}\\ X_{2i}\left\{{\hat{\text{Δ}}}_{3}^{\left(-k\right)}\left({\vec{U}}_{i}\right)-{\bar{\beta }}_{21}{\hat{\text{Δ}}}_{2}^{\left(-k\right)}\left({\vec{U}}_{i}\right)\right\} \end{array}\right].$$

From the above it follows that ${\hat{ℛ}}_{𝕊}^{\left(K\right)}={𝕋}_{ℒ}-{𝕋}_{ℒ}^{\left(K\right)}+\frac{1}{K}\sum_{k}{\bar{𝒮}}_{k}+O_{\mathbb{P}}\left(n^{-1}\right)+O_{\mathbb{P}}\left(n^{-\frac{1}{2}}\right)o_{\mathbb{P}}(1)$. Next by Assumption 2 and using Lemma 16 with ${\overset{ˆ}{C}}_{n,N}=1$, and setting functions ${\overset{ˆ}{l}}_{n}(\cdot )$, ${\overset{ˆ}{\pi }}_{n}(\cdot )$ to be the constant 1, and $f\left(X_{2}\right)=X_{2}$ to be the identity function, we have $\sqrt{n}\left({𝕋}_{ℒ}^{\left(K\right)}-\frac{1}{K}\sum_{k}{\bar{𝒮}}_{k}\right)=O_{\mathbb{P}}\left(c_{n_{K}^{-}}\right)$. Therefore ${\hat{ℛ}}_{𝕊}^{\left(K\right)}={𝕋}_{ℒ}+O_{\mathbb{P}}\left(n^{-\frac{1}{2}}c_{n_{K}^{-}}\right)$.

(II) Now we consider ${ℛ}_{𝒰}$, from the CLT, assuming working model (1), as constraints (9) are satisfied it follows that

$${ℛ}_{𝒰}=𝔼\left[\begin{array}{c} {\bar{\mu }}_{23}(\vec{U})-{\bar{\beta }}_{21}{\bar{\mu }}_{22}(\vec{U})-{\bar{\mu }}_{2}(\vec{U})X_{2}^{⊤}{\bar{\theta }}_{2-}\\ X_{2}\left\{{\bar{\mu }}_{3}(\vec{U})-{\bar{\beta }}_{21}{\bar{\mu }}_{2}(\vec{U})-X_{2}^{⊤}{\bar{\theta }}_{2-}\right\} \end{array}\right]+O_{\mathbb{P}}\left(N^{-\frac{1}{2}}\right)=1O_{\mathbb{P}}\left(N^{-\frac{1}{2}}\right).$$

(III) Next we focus on ${\overset{ˆ}{\Gamma }}_{𝕊}^{\left(K\right)}$, we use a similar expansion to (I) and define

$${\hat{ℱ}}_{𝕊}^{\eta }=\left[\begin{array}{cc} {\hat{\eta }}_{22}-\eta_{22} & \left({\hat{\eta }}_{2}-\eta_{2}\right)X_{2}^{⊤}\\ \left({\hat{\eta }}_{2}-\eta_{2}\right)X_{2} & 0 \end{array}\right],$$

$${\hat{ℱ}}_{𝕊}^{\left(K\right)}=\frac{1}{K}\sum_{k=1}^{K}{\mathbb{P}}_{N}\left[\begin{array}{cc} {\hat{\text{Δ}}}_{22}^{\left(-k\right)}(\vec{U}) & {\hat{\text{Δ}}}_{2}^{\left(-k\right)}(\vec{U})X_{2}^{⊤}\\ {\hat{\text{Δ}}}_{2}^{\left(-k\right)}(\vec{U})X_{2} & 0 \end{array}\right],$$

$${\bar{ℱ}}_{k}={𝔼}_{ℒ}\left[\begin{array}{cc} {\hat{\text{Δ}}}_{22}^{\left(-k\right)}(\vec{U}) & {\hat{\text{Δ}}}_{2}^{\left(-k\right)}(\vec{U})X_{2}^{⊤}\\ {\hat{\text{Δ}}}_{2}^{\left(-k\right)}(\vec{U})X_{2} & 0 \end{array}\right]\;\forall k\in \{1,\ldots ,K\},$$

We argue as in (I), that from (3) it follows that ${\overset{ˆ}{\Gamma }}_{𝕊}^{\left(K\right)}={\hat{ℱ}}_{𝕊}^{\eta }+{\hat{ℱ}}_{𝕊}^{\left(K\right)}$. Using (8), Assumptions 2 and Lemma 15 ${\hat{ℱ}}_{𝕊}^{\left(K\right)}-\frac{1}{K}\sum_{k}{\bar{ℱ}}_{k}=O_{\mathbb{P}}\left(N^{-\frac{1}{2}}\right)$, therefore ${\overset{ˆ}{\Gamma }}_{𝕊}^{\left(K\right)}={\hat{ℱ}}_{𝕊}^{\eta }+\frac{1}{K}\sum_{k}{\bar{ℱ}}_{k}+O_{\mathbb{P}}\left(N^{-\frac{1}{2}}\right)$. Next we follow the same decomposition for ${\hat{ℱ}}_{𝕊}^{\eta }$ as we did in (I) for ${\hat{𝒮}}_{𝕊}^{\eta }$, it follows that

$${\hat{\text{Γ}}}_{𝕊}^{\left(K\right)}={\mathbb{P}}_{n}\left[\begin{array}{cc} Y_{2}^{2}-{\bar{\mu }}_{22}(\vec{U}) & \left\{Y_{2}-{\bar{\mu }}_{2}(\vec{U})\right\}X_{2}^{⊤}\\ \left\{Y_{2}-{\bar{\mu }}_{2}(\vec{U})\right\}X_{2} & 0 \end{array}\right]\;\;-\frac{1}{n}\sum_{k=1}^{K}\sum_{i\in {ℐ}_{k}}\left[\begin{array}{cc} {\hat{\text{Δ}}}_{22}^{\left(-k\right)}(\vec{U}) & {\hat{\text{Δ}}}_{2}^{\left(-k\right)}(\vec{U})X_{2}^{⊤}\\ {\hat{\text{Δ}}}_{2}^{\left(-k\right)}(\vec{U})X_{2} & 0 \end{array}\right]+\frac{1}{K}\sum_{k}{\bar{ℱ}}_{k}+O_{\mathbb{P}}\left(n^{-1}\right)+O_{\mathbb{P}}\left(n^{-\frac{1}{2}}\right)o_{\mathbb{P}}(1).$$

The first term in the right hand side is $O_{\mathbb{P}}\left(n^{-\frac{1}{2}}\right)$ by he CLT, the next two terms together are $O_{\mathbb{P}}\left(n^{-\frac{1}{2}}c_{n_{K}^{-}}\right)$ by Lemma 16, thus ${\overset{ˆ}{\Gamma }}_{𝕊}^{\left(K\right)}=O_{\mathbb{P}}\left(n^{-\frac{1}{2}}c_{n_{K}^{-}}\right)$.

(IV) Finally we consider $\Gamma_{𝒰}$. By central limit theorem and (9) it follows that

$${\text{Γ}}_{𝒰}=𝔼\left[\begin{array}{cc} {\bar{\mu }}_{22}(\vec{U}) & {\bar{\mu }}_{2}(\vec{U})X_{2}^{⊤}\\ {\bar{\mu }}_{2}(\vec{U})X_{2} & X_{2}X_{2}^{⊤} \end{array}\right]+O_{\mathbb{P}}\left(N^{-\frac{1}{2}}\right)=𝔼\left[{\overset{ˇ}{X}}_{2}{\overset{ˇ}{X}}_{2}^{⊤}\right]+O_{\mathbb{P}}\left(N^{-\frac{1}{2}}\right).$$

From (I)-(IV) we can write (10) as $({\hat{\theta }}_{2}-{\bar{\theta }}_{2})=𝔼{\left[{\overset{ˇ}{X}}_{2}{\overset{ˇ}{X}}_{2}^{⊤}\right]}^{-1}{𝕋}_{ℒ}+O_{\mathbb{P}}\left(n^{-\frac{1}{2}}c_{n_{K}^{-}}\right)$, it follows that

$$\sqrt{n}\left({\hat{\theta }}_{2}-{\bar{\theta }}_{2}\right)=𝔼{\left[{\overset{ˇ}{X}}_{2}{\overset{ˇ}{X}}_{2}^{⊤}\right]}^{-1}\;\times \frac{1}{\sqrt{n}}\sum_{i=1}^{n}\left[\begin{array}{c} \left\{Y_{2i}Y_{3i}-{\bar{\mu }}_{23}\left({\vec{U}}_{i}\right)\right\}-{\bar{\beta }}_{21}\left\{Y_{2i}^{2}-{\bar{\mu }}_{22}\left({\vec{U}}_{i}\right)\right\}-{\overset{ˇ}{X}}_{2i}^{⊤}{\bar{\theta }}_{2-}\left\{Y_{2i}-{\bar{\mu }}_{2}\left({\vec{U}}_{i}\right)\right\}\\ X_{2i}\left\{Y_{3i}-{\bar{\mu }}_{3}\left({\vec{U}}_{i}\right)\right\}-{\bar{\beta }}_{21}X_{2i}\left\{Y_{2i}-{\bar{\mu }}_{2}\left({\vec{U}}_{i}\right)\right\} \end{array}\right]\;+o_{\mathbb{P}}(1).$$

■

**Proof** [Proof of Theorem 3] The solution to stage 1 estimating equation $\theta_{1}$ in Section 3.2 satisfies

$${\mathbb{P}}_{N}\left[X_{1}\left\{{\hat{\mu }}_{2}(\vec{U})+{\hat{\beta }}_{21}{\hat{\mu }}_{2}(\vec{U})+H_{20}^{⊤}{\hat{\beta }}_{22}+{\left[H_{21}^{⊤}{\hat{\gamma }}_{2}\right]}_{+}-X_{1}^{⊤}{\hat{\theta }}_{1}\right\}\right]=0.$$

We center the above at ${\bar{\theta }}_{1}$ and get

(11)
$${\mathbb{P}}_{N}\left[X_{1}X_{1}^{⊤}\right]\left({\hat{\theta }}_{1}-{\bar{\theta }}_{1}\right)={\mathbb{P}}_{N}\left[X_{1}\left\{{\bar{\mu }}_{2}(\vec{U})+{\hat{\beta }}_{21}{\bar{\mu }}_{2}(\vec{U})+H_{20}^{⊤}{\hat{\beta }}_{22}+{\left[H_{21}^{⊤}{\hat{\gamma }}_{2}\right]}_{+}-X_{1}^{⊤}{\bar{\theta }}_{1}\right\}\right].$$

Next, with the following definitions

$${\hat{\text{Σ}}}_{𝒰}={\mathbb{P}}_{N}\left[X_{1}X_{1}^{⊤}\right],\;{\hat{\text{Σ}}}_{ℒ}={\mathbb{P}}_{n}\left[X_{1}X_{1}^{⊤}\right],$$

$${ℛ}^{\left(1\right)}={\mathbb{P}}_{N}\left[X_{1}\left\{{\bar{\mu }}_{2}(\vec{U})+{\hat{\beta }}_{21}{\bar{\mu }}_{2}(\vec{U})+H_{20}^{⊤}{\hat{\beta }}_{22}+{\left[H_{21}^{⊤}{\hat{\gamma }}_{2}\right]}_{+}-X_{1}^{⊤}{\hat{\theta }}_{1}\right\}\right],$$

$${\hat{ℛ}}_{𝕊}^{\left(1K\right)}={\mathbb{P}}_{N}\left[X_{1}\left\{{\hat{\mu }}_{2}(\vec{U})-{\bar{\mu }}_{2}(\vec{U})\right\}\right],$$

we can write (11) as ${\overset{ˆ}{\Sigma }}_{𝒰}({\hat{\theta }}_{1}-{\bar{\theta }}_{1})={ℛ}^{\left(1\right)}+\left(1+{\overset{ˆ}{\beta }}_{21}\right){\hat{ℛ}}_{𝕊}^{\left(1K\right)}$. We now analyze both terms ${ℛ}^{\left(1\right)}$, and $\left(1+{\overset{ˆ}{\beta }}_{21}\right){\hat{ℛ}}_{𝕊}^{\left(1K\right)}$.

I) First we consider $\left(1+{\overset{ˆ}{\beta }}_{21}\right){\hat{ℛ}}_{𝕊}^{\left(1K\right)}$, define

$${\hat{𝒮}}_{𝕊}^{\left(1\eta \right)}={\hat{\text{Σ}}}_{𝒰}\left({\hat{\eta }}_{2}-\eta_{2}\right),$$

$${\hat{𝒮}}_{𝕊}^{\left(1𝕂\right)}=\frac{1}{K}\sum_{k=1}^{K}{\mathbb{P}}_{N}\left[X_{1}{\hat{\text{Δ}}}_{2}^{\left(-k\right)}(\vec{U})\right],$$

$${\bar{𝒮}}_{k}^{\left(1\right)}=𝔼\left[X_{1}{\hat{\text{Δ}}}_{2}^{\left(-k\right)}(\vec{U})\right],$$

from (3) it follows that ${\hat{ℛ}}_{𝕊}^{\left(1K\right)}={\hat{𝒮}}_{𝕊}^{\left(1\eta \right)}+{\hat{𝒮}}_{𝕊}^{\left(1𝕂\right)}$, next from Assumptions 1, 2, we get $\sum_{k=1}^{K}\underset{X_{1},\vec{U}}{sup}\|X_{1}{\overset{ˆ}{\Delta }}_{2}^{\left(-k\right)}(\vec{U})\|=o_{\mathbb{P}}(1)$, thus by Lemma 15 ${\hat{𝒮}}_{𝕊}^{\left(1𝕂\right)}={\bar{𝒮}}_{k}^{\left(1\right)}+\left(N^{-\frac{1}{2}}\right)$. Using (3) again, and recalling ${\overset{‾}{\mu }}_{2}(\vec{U})=m_{2}(\vec{U})+X_{1}^{⊤}\eta_{2}$ we have

$${\hat{𝒮}}_{𝕊}^{\left(1\eta \right)}={\hat{\text{Σ}}}_{𝒰}{\hat{\text{Σ}}}_{ℒ}^{-1}\frac{1}{n}\sum_{k=1}^{K}\sum_{i\in {ℐ}_{k}}X_{1i}\left\{Y_{2i}-{\bar{\mu }}_{2}\left({\vec{U}}_{i}\right)-{\hat{m}}_{2}^{\left(-k\right)}\left({\vec{U}}_{i}\right)+m_{2}\left({\vec{U}}_{i}\right)\right\}\;\;=\frac{1}{n}\sum_{i=1}^{n}X_{1i}\left\{Y_{2i}-{\bar{\mu }}_{2}\left({\vec{U}}_{i}\right)\right\}-\frac{1}{n}\sum_{k=1}^{K}\sum_{i\in {ℐ}_{k}}X_{1i}{\hat{\text{Δ}}}_{2}^{\left(-k\right)}\left({\vec{U}}_{i}\right)+O_{\mathbb{P}}\left(n^{-\frac{1}{2}}\right),$$

where the last line follows by the CLT and Assumptions 1 and 2 as

$${\hat{\text{Σ}}}_{𝒰}{\hat{\text{Σ}}}_{ℒ}^{-1}=I+O_{\mathbb{P}}\left(n^{-\frac{1}{2}}\right)$$

Now using Lemma 15 and Assumptions 1, 2 again, it follows that

$${\hat{𝒮}}_{𝕊}^{\left(1𝕂\right)}={\bar{𝒮}}_{k}^{\left(1\right)}+O_{\mathbb{P}}\left(N^{-\frac{1}{2}}\right),$$

combining the above we can write

$${\hat{ℛ}}_{𝕊}^{\left(1K\right)}={\mathbb{P}}_{n}X_{1}\left\{Y_{2}-{\bar{\mu }}_{2}(\vec{U})\right\}-\frac{1}{n}\sum_{k=1}^{K}\left\{\sum_{i\in {ℐ}_{k}}X_{1i}{\hat{\text{Δ}}}_{2}^{\left(-k\right)}\left({\vec{U}}_{i}\right)-𝔼\left[X_{1}{\hat{\text{Δ}}}_{2}^{\left(-k\right)}(\vec{U})\right]\right\}+O_{\mathbb{P}}\left(n^{-\frac{1}{2}}\right).$$

Next by Assumption 2 and Lemma 16 we have

$$\frac{1}{\sqrt{n}}\sum_{k=1}^{K}\left\{\sum_{i\in {ℐ}_{k}}X_{1i}{\hat{\text{Δ}}}_{2}^{\left(-k\right)}\left({\vec{U}}_{i}\right)-𝔼\left[X_{1}{\hat{\text{Δ}}}_{2}^{\left(-k\right)}(\vec{U})\right]\right\}=O_{\mathbb{P}}\left(c_{n_{K}^{-}}\right),$$

therefore ${\hat{ℛ}}_{𝕊}^{\left(1K\right)}={\mathbb{P}}_{n}X_{1}\left\{Y_{2}-{\overset{‾}{\mu }}_{2}(\vec{U})\right\}+O_{\mathbb{P}}\left(n^{-\frac{1}{2}}c_{n_{K}^{-}}\right)$. Finally using Theorem 2 we have ${\overset{ˆ}{\beta }}_{21}-{\overset{‾}{\beta }}_{21}=O_{\mathbb{P}}\left(n^{-\frac{1}{2}}\right)$, and by CLT ${\mathbb{P}}_{n}X_{1}\left\{Y_{2}-{\overset{‾}{\mu }}_{2}(\vec{U})\right\}=O_{\mathbb{P}}\left(n^{-\frac{1}{2}}\right)$, thus we can write

$$\left(1+{\hat{\beta }}_{21}\right){\hat{ℛ}}_{𝕊}^{\left(1K\right)}=\left(1+{\hat{\beta }}_{21}\right){\mathbb{P}}_{n}X_{1}\left\{Y_{2}-{\bar{\mu }}_{2}(\vec{U})\right\}+O_{\mathbb{P}}\left(n^{-\frac{1}{2}}c_{n_{K}^{-}}\right).$$

II) Next we consider ${ℛ}^{\left(1\right)}$ by writing

$${ℛ}^{\left(1\right)}={\mathbb{P}}_{N}\left[X_{1}\left\{{\bar{\mu }}_{2}(\vec{U})+{\bar{\beta }}_{21}{\bar{\mu }}_{2}(\vec{U})+H_{20}^{⊤}{\bar{\beta }}_{22}+{\left[H_{21}^{⊤}{\bar{\gamma }}_{2}\right]}_{+}-X_{1}^{⊤}{\bar{\theta }}_{1}\right\}\right]\;\;+{\mathbb{P}}_{N}\left[X_{1}\left\{{\bar{\mu }}_{2}(\vec{U})\left({\hat{\beta }}_{21}-{\bar{\beta }}_{21}\right)+H_{20}^{⊤}\left({\hat{\beta }}_{22}-{\bar{\beta }}_{22}\right)+{\left[H_{21}^{⊤}{\hat{\gamma }}_{2}\right]}_{+}-{\left[H_{21}^{⊤}{\bar{\gamma }}_{2}\right]}_{+}\right\}\right],$$

note that under (9) using model (1) the first term in the right hand side is mean zero, therefore from Assumption 1 and CLT

$${\mathbb{P}}_{N}\left\{X_{1}\left({\bar{\mu }}_{2}(\vec{U})+{\bar{\beta }}_{21}{\bar{\mu }}_{2}(\vec{U})+H_{20}^{⊤}{\bar{\beta }}_{22}+{\left[H_{21}^{⊤}{\bar{\gamma }}_{2}\right]}_{+}-X_{1}^{⊤}{\bar{\theta }}_{1}\right)\right\}=O_{\mathbb{P}}\left(N^{-\frac{1}{2}}\right).$$

Hence, we have

$$\sqrt{n}{ℛ}^{\left(1\right)}=\sqrt{n}{\mathbb{P}}_{N}\left[X_{1}\left\{{\bar{\mu }}_{2}(\vec{U})\left({\hat{\beta }}_{21}-{\bar{\beta }}_{21}\right)+H_{20}^{⊤}\left({\hat{\beta }}_{22}-{\bar{\beta }}_{22}\right)+{\left[H_{21}^{⊤}{\hat{\gamma }}_{2}\right]}_{+}-{\left[H_{21}^{⊤}{\bar{\gamma }}_{2}\right]}_{+}\right\}\right]\;\;+O_{\mathbb{P}}\left(\sqrt{\frac{n}{N}}\right)\;\;={\mathbb{P}}_{N}\left[X_{1}\left({\bar{\mu }}_{2}(\vec{U}),H_{20}^{⊤}\right)\right]\sqrt{n}\left({\hat{\beta }}_{2}-{\bar{\beta }}_{2}\right)+\sqrt{n}{\mathbb{P}}_{N}\left[X_{1}\left({\left[H_{21}^{⊤}{\hat{\gamma }}_{2}\right]}_{+}-{\left[H_{21}^{⊤}{\bar{\gamma }}_{2}\right]}_{+}\right)\right]\;\;+O_{\mathbb{P}}\left(\sqrt{\frac{n}{N}}\right)\;\;=𝔼\left[X_{1}\left({\bar{\mu }}_{2}(\vec{U}),H_{20}^{⊤}\right)\right]n^{-\frac{1}{2}}\sum_{i=1}^{n}\psi_{2i\beta }+\sqrt{n}{\mathbb{P}}_{N}\left[X_{1}\left({\left[H_{21}^{⊤}{\hat{\gamma }}_{2}\right]}_{+}-{\left[H_{21}^{⊤}{\bar{\gamma }}_{2}\right]}_{+}\right)\right]\;\;+O_{\mathbb{P}}\left(\sqrt{\frac{n}{N}}\right),$$

where the last inequality follows from the CLT, where $\psi_{2i\beta }$ is the element corresponding to ${\hat{\beta }}_{2}$ of the influence function $\psi_{2i}$ defined in Theorem 2.

Next by Theorem 2 we know that

$$\sqrt{n}\left({\hat{\gamma }}_{2}-{\bar{\gamma }}_{2}\right)=O_{\mathbb{P}}(1),$$

using Lemma 17 (a) we have

$$\mathbb{P}\left[\sqrt{n}{\mathbb{P}}_{N}\left\{X_{1}\left({\left[H_{21}^{⊤}{\hat{\gamma }}_{2}\right]}_{+}-{\left[H_{21}^{⊤}{\bar{\gamma }}_{2}\right]}_{+}\right)\right\}={\mathbb{P}}_{N}\left\{X_{1}H_{21}^{⊤}I\left(H_{21}^{⊤}{\bar{\gamma }}_{2}>0\right)\right\}\sqrt{n}\left({\hat{\gamma }}_{2}-{\bar{\gamma }}_{2}\right)\right]\to 1.$$

Therefore, letting $\psi_{2i\gamma }$ be the element corresponding to ${\hat{\gamma }}_{2}$ of the influence function $\psi_{2i}$ defined in Theorem 2,

$$\sqrt{n}{\mathbb{P}}_{N}\left\{X_{1}\left({\left[H_{21}^{⊤}{\hat{\gamma }}_{2}\right]}_{+}-{\left[H_{21}^{⊤}{\bar{\gamma }}_{2}\right]}_{+}\right)\right\}\;={\mathbb{P}}_{N}\left\{X_{1}H_{21}^{⊤}I\left(H_{21}^{⊤}{\bar{\gamma }}_{2}>0\right)I\left({\hat{\gamma }}_{2}\in 𝒜\right)\right\}\sqrt{n}\left({\hat{\gamma }}_{2}-{\bar{\gamma }}_{2}\right)\;+\sqrt{n}{\mathbb{P}}_{N}\left\{X_{1}\left({\left[H_{21}^{⊤}{\bar{\gamma }}_{2}\right]}_{+}-{\left[H_{21}^{⊤}{\bar{\gamma }}_{2}\right]}_{+}\right)\right\}I_{\left\{{\hat{\gamma }}_{2}\notin 𝒜\right\}}\;=𝔼\left[X_{1}H_{21}^{⊤}|H_{21}^{⊤}{\hat{\gamma }}_{2}>0,{\hat{\gamma }}_{2}\in 𝒜\right]\mathbb{P}\left(H_{21}^{⊤}{\bar{\gamma }}_{2}>0\right)\mathbb{P}\left({\hat{\gamma }}_{2}\in 𝒜\right)\frac{1}{\sqrt{n}}\sum_{i=1}^{n}\psi_{2_{\gamma 2i}}+O_{\mathbb{P}}\left(c_{n_{K}^{-}}\right)+o_{\mathbb{P}}\left(1\right)\;=𝔼\left[X_{1}H_{21}^{⊤}|H_{21}^{⊤}{\bar{\gamma }}_{2}>0\right]\mathbb{P}\left(H_{21}^{⊤}{\bar{\gamma }}_{2}>0\right)\frac{1}{\sqrt{n}}\sum_{i=1}^{n}\psi_{2_{\gamma 2i}}+o_{\mathbb{P}}(1),$$

combining all terms

$$\sqrt{n}{ℛ}^{\left(1\right)}=𝔼\left[X_{1}\left({\bar{\mu }}_{2}(\vec{U}),H_{20}^{⊤}\right)\right]n^{-\frac{1}{2}}\sum_{i=1}^{n}\psi_{2i(\beta )}\;\;+𝔼\left[X_{1}H_{21}^{⊤}|H_{21}^{⊤}{\bar{\gamma }}_{2}>0\right]\mathbb{P}\left(H_{21}^{⊤}{\bar{\gamma }}_{2}>0\right)\frac{1}{\sqrt{n}}\sum_{i=1}^{n}\psi_{2i(\gamma )}\;\;+O_{\mathbb{P}}\left(c_{n_{K}^{-}}\right).$$

Finally, from I), II), and since ${\overset{ˆ}{\Sigma }}_{𝒰}^{-1}=𝔼{\left[X_{1}X_{1}^{⊤}\right]}^{-1}+o_{\mathbb{P}}(1)$ by the LLN, we have

$$\sqrt{n}\left({\hat{\theta }}_{1}-{\bar{\theta }}_{1}\right)=𝔼{\left[X_{1}X_{1}^{⊤}\right]}^{-1}\sum_{𝒰}^{-1}{ℛ}^{\left(1\right)}+𝔼{\left[X_{1}X_{1}^{⊤}\right]}^{-1}\left(1+{\hat{\beta }}_{21}\right){\hat{ℛ}}_{𝕊}^{\left(1K\right)}+o_{\mathbb{P}}(1)\;\;=𝔼{\left[X_{1}X_{1}^{⊤}\right]}^{-1}\left(1+{\bar{\beta }}_{21}\right)\frac{1}{\sqrt{n}}\sum_{i=1}^{n}\left\{Y_{2i}-{\bar{\mu }}_{2}\left({\vec{U}}_{i}\right)\right\}\;\;+𝔼{\left[X_{1}X_{1}^{⊤}\right]}^{-1}𝔼\left[X_{1}\left({\bar{\mu }}_{2}(\vec{U}),H_{20}^{⊤}\right)\right]\frac{1}{\sqrt{n}}\sum_{i=1}^{n}\psi_{2i(\beta )}\;\;+𝔼{\left[X_{1}X_{1}^{⊤}\right]}^{-1}𝔼\left[X_{1}H_{21}^{⊤}|H_{21}^{⊤}{\bar{\gamma }}_{2}>0\right]\mathbb{P}\left(H_{21}^{⊤}{\bar{\gamma }}_{2}>0\right)\frac{1}{\sqrt{n}}\sum_{i=1}^{n}\psi_{2i\gamma }\;\;+o_{\mathbb{P}}(1),$$

using (9) we have $𝔼\left[X_{1}\left({\overset{‾}{\mu }}_{2}(\vec{U}),H_{20}^{⊤}\right)\right]=𝔼\left[X_{1}\left(Y_{2},H_{20}^{⊤}\right)\right]$ which yields our required results ■

Next we discuss some results and assumptions needed for Proposition 5. First we show the asymptotic results for the supervised estimation of the Q function parameters. Recall ${\hat{\theta }}_{\text{1SUP}}$, ${\hat{\theta }}_{\text{2SUP}}$ are the estimators for the Q function parameters, when using the labeled data ℒ only. From Laber et al. (2014) we have that the following results for ${\hat{\theta }}_{\text{2SUP}}$:

$$\sqrt{n}\left({\hat{\theta }}_{\mathrm{2SUP}}-{\bar{\theta }}_{2}\right)=\sum_{2}^{-1}\frac{1}{\sqrt{n}}\sum_{i=1}^{n}\psi_{\mathrm{2SUP}}\left(L;{\bar{\theta }}_{2}\right)\to 𝒩\left(0,V_{\mathrm{2SUP}}\left[{\bar{\theta }}_{2}\right]\right),$$

with

$$\psi_{\mathrm{2SUP}}\;\left(L;{\bar{\theta }}_{2}\right)={\overset{ˇ}{X}}_{2}\left\{Y_{3i}-{\overset{ˇ}{X}}_{2i}^{⊤}{\bar{\theta }}_{2}\right\},$$

$$V_{\mathrm{2SUP}}\;\left[{\bar{\theta }}_{2}\right]=\sum_{2}^{-1}𝔼\left[\psi_{\mathrm{2SUP}}\;\left(L;{\bar{\theta }}_{2}\right)\psi_{\mathrm{2SUP}}{\left(L;{\bar{\theta }}_{2}\right)}^{⊤}\right]{\left(\sum_{2}^{-1}\;\right)}^{⊤},$$

and for ${\hat{\theta }}_{\mathrm{1SUP}}$:

$$\sqrt{n}\left({\hat{\theta }}_{\mathrm{1SUP}}-{\bar{\theta }}_{1}\right)=\sum_{1}^{-1}\frac{1}{\sqrt{n}}\sum_{i=1}^{n}\psi_{\mathrm{1SUP}}\left(L;{\bar{\theta }}_{1}\right)\to 𝒩\left(0,V_{1\text{sup}}\left[{\bar{\theta }}_{1}\right]\right),$$

with

$$\psi_{\mathrm{1SUP}}\left(L_{i};{\bar{\theta }}_{2}\right)=X_{i1}\left\{Y_{2i}+Y_{2i}{\bar{\beta }}_{21}+H_{20i}^{⊤}{\bar{\beta }}_{22}+{\left[H_{21i}^{⊤}{\bar{\gamma }}_{2}\right]}_{+}-X_{1i}^{⊤}{\bar{\theta }}_{1}\right\}\;\;+𝔼\left[X_{1}\left(Y_{2},H_{20}^{⊤}\right)\right]\psi_{\mathrm{2SUP}\;,(\beta )}\left(L_{i}\right)\;\;+𝔼\left[X_{1}H_{21}^{⊤}|H_{21}^{⊤}{\bar{\gamma }}_{2}>0\right]\mathbb{P}\left(H_{21}^{⊤}{\bar{\gamma }}_{2}>0\right)\psi_{\mathrm{2SUP},\;(\gamma )}\left(L_{i}\right),\;\;V_{\mathrm{1SUP}}\;\left[{\bar{\theta }}_{1}\right]=\sum_{1}^{-1}𝔼\left[\psi_{\mathrm{1SUP}}\;\left(L;{\bar{\theta }}_{1}\right)\psi_{\mathrm{1SUP}}\;{\left(L;{\bar{\theta }}_{1}\right)}^{⊤}\right]{\left(\sum_{1}^{-1}\right)}^{⊤}.$$

Next we discuss the assumption required for Proposition 5. We need the imputation models ${\overset{‾}{\mu }}_{s}(\vec{U}),s\in \{2,3,22,23\}$ to satisfy several additional constraints. For example, for the stage two Q function parameters, recall $\theta_{2-}={\left(\beta_{22}^{⊤},\gamma_{2}^{⊤}\right)}^{⊤}$, the imputation models should satisfy:

$$𝔼\left[{\vec{X}}^{⊤}{\bar{\mu }}_{j}(\vec{U})\left\{g_{s}(Y)-{\bar{\mu }}_{s}(\vec{U})\right\}\right]=0\;𝔼\left[{\vec{X}}^{⊤}{\bar{\mu }}_{2}(\vec{U})X_{2}^{⊤}{\bar{\theta }}_{2-}\left\{g_{s}(Y)-{\bar{\mu }}_{s}(\vec{U})\right\}\right]=0,$$

for s,j∈{2,3,22,23}, and

$$𝔼\left[\vec{X}{\vec{X}}^{⊤}{\bar{\mu }}_{j}(\vec{U})\left\{g_{s}(Y)-{\bar{\mu }}_{s}(\vec{U})\right\}\right]=0,\;𝔼\left[\vec{X}{\vec{X}}^{⊤}X_{2}^{⊤}{\bar{\theta }}_{2-}\left\{g_{s}(Y)-{\bar{\mu }}_{s}(\vec{U})\right\}\right]=0,$$

for s,j∈{2,3}, where $X={\left(1,X_{1}^{⊤},X_{2}^{⊤}\right)}^{⊤},g_{2}(Y)=Y_{2},g_{3}(Y)=Y_{3},g_{22}(Y)=Y_{2}^{2},g_{23}(Y)=Y_{2}Y_{3}$.

To summarize all the assumptions needed, we define the following functions:

(12)
$${ℰ}^{\theta }(\vec{U})\equiv {\left\{{ℰ}_{1}{\left(\vec{U}\right)}^{⊤},{ℰ}_{2}{\left(\vec{U}\right)}^{⊤}\right\}}^{⊤},\;{ℰ}_{2}(\vec{U})\equiv \left[\begin{array}{c} {\bar{\mu }}_{23}(\vec{U})-\left[{\bar{\mu }}_{22}(\vec{U}),{\bar{\mu }}_{2}(\vec{U})X_{2}^{⊤}\right]{\bar{\theta }}_{2}\\ X_{2}\left\{{\bar{\mu }}_{3}(\vec{U})-\left[{\bar{\mu }}_{2}(\vec{U}),X_{2}^{⊤}\right]{\bar{\theta }}_{2}\right\} \end{array}\right],\;{ℰ}_{1}(\vec{U})\equiv X_{1}\left\{{\bar{\mu }}_{2}(\vec{U})\left(1+{\bar{\beta }}_{21}\right)+Q_{2-}^{o}\left(H_{2};{\bar{\theta }}_{2}\right)-X_{1}^{⊤}{\bar{\theta }}_{1}\right\}\;\;+𝔼\left[X_{1}\left(Y_{2},H_{20}^{⊤}\right)\right]{ℰ}_{2\beta }(\vec{U})\;\;+𝔼\left[X_{1}H_{21}^{⊤}|H_{21}^{⊤}{\bar{\gamma }}_{2}>0\right]\mathbb{P}\left(H_{21}^{⊤}{\bar{\gamma }}_{2}>0\right){ℰ}_{2\gamma }(\vec{U}),$$

where ${ℰ}_{2\beta }(\vec{U}),{ℰ}_{2\gamma }(\vec{U})$ are the elements corresponding to ${\bar{\beta }}_{2},{\bar{\gamma }}_{2}$ of ${ℰ}_{2}(\vec{U})$. Now we can succinctly summarize the constraints, by having ${\overset{‾}{\mu }}_{s}(\vec{U}),s\in \{2,3,22,23\}$ satisfy

$$𝔼\left[\left\{\psi_{\mathrm{2SUP}}\left(L;{\bar{\theta }}_{2}\right)-{ℰ}_{2}(\vec{U})\right\}{ℰ}_{2}{\left(\vec{U}\right)}^{⊤}\right]=0.$$

This is condensed in the following assumption.

**Assumption 7** *Let* ${ℰ}^{\theta }(\vec{U})$ *be as defined in* (12), *and*

$$\psi_{\text{SUP}}(L;\bar{\theta })={\left[\psi_{\mathrm{1SUP}}{\left(L;{\bar{\theta }}_{1}\right)}^{⊤},\psi_{\mathrm{2SUP}}{\left(L;{\bar{\theta }}_{2}\right)}^{⊤}\right]}^{⊤},$$

*the imputation models* ${\overset{‾}{\mu }}_{s}(\vec{U}),s\in \{2,3,22,23\}$ *satisfy*

$$𝔼\left[\left\{\psi_{\text{SUP}}(L;\bar{\theta })-{ℰ}^{\theta }(\vec{U})\right\}{ℰ}^{\theta }{\left(\vec{U}\right)}^{⊤}\right]=0.$$

**Proof** [Proof of Proposition 5]

We first show the result is true for $V_{2SSL}\left[{\bar{\theta }}_{2}\right]$. To simplify algebra, we denote the influence function from Theorem 2 as $\psi_{2\text{SSL}}\left(L;{\bar{\theta }}_{2}\right)$. Using the influence function of ${\hat{\theta }}_{\mathrm{2SUP}}$ and Theorem 2 we have the following relationship:

$$\psi_{2\text{SSL}}\left(L;{\bar{\theta }}_{2}\right)=\psi_{\mathrm{2SUP}}\left(L;{\bar{\theta }}_{2}\right)-{ℰ}_{2}(\vec{U}).$$

Therefore

$$V_{2\text{SSL}}\left({\bar{\theta }}_{2}\right)=\sum_{2}^{-1}𝔼\left[\psi_{2\text{SSL}}\left(L;{\bar{\theta }}_{2}\right)\psi_{2\text{SSL}}{\left(L;{\bar{\theta }}_{2}\right)}^{⊤}\right]{\left(\sum_{2}^{-1}\right)}^{⊤}\;\;=\sum_{2}^{-1}𝔼\left[\left\{\psi_{\mathrm{2SUP}}\left(L;{\bar{\theta }}_{2}\right)-{ℰ}_{2}(\vec{U})\right\}\;{\left\{\psi_{\mathrm{2SUP}}\left(L;{\bar{\theta }}_{2}\right)-{ℰ}_{2}(\vec{U})\right\}}^{⊤}\right]{\left(\sum_{2}^{-1}\right)}^{⊤}\;\;=\sum_{2}^{-1}𝔼\left[\psi_{\mathrm{2SUP}}\left(L;{\bar{\theta }}_{2}\right)\psi_{\mathrm{2SUP}}{\left(L;{\bar{\theta }}_{2}\right)}^{⊤}\right]{\left(\sum_{2}^{-1}\right)}^{⊤}\;\;+\sum_{2}^{-1}𝔼\left[{ℰ}_{2}(\vec{U}){ℰ}_{2}{\left(\vec{U}\right)}^{⊤}\right]{\left(\sum_{2}^{-1}\right)}^{⊤}\;\;-2\sum_{2}^{-1}𝔼\left[\psi_{\mathrm{2SUP}}\left(L;{\bar{\theta }}_{2}\right){ℰ}_{2}{\left(\vec{U}\right)}^{⊤}\right]{\left(\sum_{2}^{-1}\right)}^{⊤}$$

Now, since our imputation models satisfy Assumption 7, it follows that

$$𝔼\left[\left\{\psi_{\text{2SUP}}\left(L;{\bar{\theta }}_{2}\right)-{ℰ}_{2}(\vec{U})\right\}{ℰ}_{2}{\left(\vec{U}\right)}^{⊤}\right]=0.$$

Therefore we have

$$V_{2\text{SSL}}\left({\bar{\theta }}_{2}\right)=V_{\mathrm{2SUP}}\left({\bar{\theta }}_{2}\right)-\sum_{2}^{-1}\text{Var}\left[{ℰ}_{2}(\vec{U})\right]{\left(\sum_{2}^{-1}\right)}^{⊤}.$$

To show the result is true for $V_{1\text{SSL}}\left[{\bar{\theta }}_{1}\right]$, We denote by ${ℰ}_{2\beta }(\vec{U})$ and ${ℰ}_{2\gamma }(\vec{U})$ the vectors corresponding to ${\bar{\beta }}_{2},{\bar{\gamma }}_{2}$ in ${ℰ}_{2}(\vec{U})$ respectively, and further recall the definition of ${ℰ}_{1}(\vec{U})$:

$${ℰ}_{1}(\vec{U})=X_{1}\left\{{\bar{\mu }}_{2}(\vec{U})+{\bar{\mu }}_{2}(\vec{U}){\bar{\beta }}_{21}+H_{20}^{⊤}{\bar{\beta }}_{22}+{\left[H_{21}^{⊤}{\bar{\gamma }}_{2}\right]}_{+}-X_{1}^{⊤}{\bar{\theta }}_{1}\right\}\;\;+𝔼\left[X_{1}\left(Y_{2},H_{20}^{⊤}\right)\right]{ℰ}_{2\beta }(\vec{U})\;\;+𝔼\left[X_{1}H_{21}^{⊤}|H_{21}^{⊤}{\bar{\gamma }}_{2}>0\right]\mathbb{P}\left(H_{21}^{⊤}{\bar{\gamma }}_{2}>0\right){ℰ}_{2\gamma }(\vec{U}).$$

From the form of the influence function of ${\hat{\theta }}_{\mathrm{1SUP}}$, and Theorems 2 & 3 we have that:

$$\psi_{1\text{SSL}}\left(L;{\bar{\theta }}_{1}\right)=\psi_{\mathrm{1SUP}}\left(L;{\bar{\theta }}_{1}\right)-{ℰ}_{1}(\vec{U}).$$

Analogous steps for the proof of ${\bar{\theta }}_{2}$ can then be used to show

$$V_{1\text{SSL}}\left({\bar{\theta }}_{1}\right)=V_{\mathrm{1SUP}}\left({\bar{\theta }}_{1}\right)-\sum_{1}^{-1}\text{Var}\left[{ℰ}_{1}(\vec{U})\right]{\left(\sum_{1}^{-1}\right)}^{⊤}.$$

The required result is obtained by stacking the influence functions for $\theta_{1},\theta_{2}$ for the supervised and semi-supervised versions, noting that

$$\psi_{\text{SSL}}(L;\bar{\theta })=\psi_{\text{SUP}}(L;\bar{\theta })-{ℰ}^{\theta }(\vec{U}).$$

and repeating the steps above. ■

### D.2 Value Function Results

In this section we prove the main results for our SSL value function estimator. Before the proofs we go over some useful definitions, notation and lemmas. First recall that, in order to correct for potential biases arising from finite sample estimation and model mis-specifications, the final imputed models for $\left\{Y_{2},\omega_{2}({\overset{ˇ}{H}}_{2},A_{2};\bar{\Theta }),Y_{t}\omega_{2}({\overset{ˇ}{H}}_{2},A_{2};\bar{\Theta }),t=2,3\right\}$ satisfy the following constraints:

(13)
$$\;𝔼\left[\omega_{1}\left({\overset{ˇ}{H}}_{1},A_{1};\bar{\Theta }\right)\left\{Y_{2}-{\bar{\mu }}_{2}^{v}(\vec{U})\right\}\right]=0,\;𝔼\left[Q_{2-}^{o}\left(\vec{U};\theta_{2}\right)\left\{\omega_{2}\left({\overset{ˇ}{H}}_{2},A_{2};\bar{\Theta }\right)-{\bar{\mu }}_{\omega_{2}}^{v}(\vec{U})\right\}\right]=0,\;\;𝔼\left[\omega_{2}\left({\overset{ˇ}{H}}_{2},A_{2};\bar{\Theta }\right)Y_{t}-{\bar{\mu }}_{t\omega_{2}}^{v}(\vec{U})\right]=0,t=2,3.$$

Next, define the set

$$𝒮(\delta )=\left\{(\theta ,\xi )|{\left\|\hat{\theta }-\theta \right\|}_{2}^{2}<\delta ,{\left\|\hat{\xi }-\xi \right\|}_{2}^{2}<\delta ,\theta_{t}\in {\text{Θ}}_{t},\xi_{t}\in {\text{Ω}}_{t},t=1,2,\pi_{1}\left(H_{1};\xi_{1}\right)>0,\pi_{2}\left({\overset{ˇ}{H}}_{2};\xi_{2}\right)>0,\forall H\in ℋ\right\}.$$

We will be using the influence functions for our model parameters Θ. In this regard let $\psi^{\theta }={\left(\psi_{1}^{⊤},\psi_{2}^{⊤}\right)}^{⊤}$. By Theorems 2 & 3 $\sqrt{n}(\hat{\theta }-\bar{\theta })=n^{-1/2}\sum_{i=1}^{n}\psi^{\theta }\left({\vec{U}}_{i}\right)+o_{\mathbb{P}}(1)$. Next, from Assumption 5, it can be shown that $\hat{\xi }$ has the following expansion: $\sqrt{n}(\hat{\xi }-\bar{\xi })=n^{-1/2}\sum_{i=1}^{n}\psi^{\xi }\left(L_{i};\bar{\xi }\right)+o_{\mathbb{P}}(1)$, where

$$\psi_{t}^{\xi }(L;\bar{\xi })=𝔼{\left\{{\overset{ˇ}{H}}_{t}^{⊤}{\overset{ˇ}{H}}_{t}\sigma \left({\overset{ˇ}{H}}_{t}^{⊤}{\bar{\xi }}_{t}\right)\left[1-\sigma \left({\overset{ˇ}{H}}_{t}^{⊤}{\bar{\xi }}_{t}\right)\right]\right\}}^{-1}{\overset{ˇ}{H}}_{t}\left\{A_{t}-\sigma \left({\overset{ˇ}{H}}_{t}^{⊤}{\bar{\xi }}_{t}\right)\right\},\;t=1,2,$$

$\psi^{\xi }(L;\bar{\xi })=\left[\psi_{1}^{\xi }(L;\bar{\xi }),\psi_{2}^{\xi }(L;\bar{\xi })\right]$ and $𝔼[\psi^{\xi }]=0,𝔼[{\left(\psi^{\xi }\right)}^{⊤}\psi^{\xi }]<\infty$.

We now introduce a set of definitions used in this section to make the proofs easier to read. Recall from (7) we have ${\hat{V}}_{{SSL}_{DR}}={\mathbb{P}}_{N}\{{𝒱}_{{SSL}_{DR}}(\vec{U};\hat{\Theta },\hat{\mu })\}$, where ${𝒱}_{{SS}_{DR}}(\vec{U};\hat{\Theta },\hat{\mu })$ is the semi-supervised augmented estimator for observation $\vec{U}$, we re-write ${𝒱}_{{SSL}_{DR}}(\vec{U};\hat{\Theta },\hat{\mu })$ as ${𝒱}_{\hat{\Theta },\hat{\mu }}(\vec{U})$ recall its definition, and define the following functions:

(14)
$${𝒱}_{\hat{\Theta },\hat{\mu }}(\vec{U})\equiv Q_{1}^{o}\left({\overset{ˇ}{H}}_{1};{\hat{\theta }}_{1}\right)+\omega_{1}\left({\overset{ˇ}{H}}_{1},A_{1},\hat{\Theta }\right)\left[\left(1+{\hat{\beta }}_{21}\right){\hat{\mu }}_{2}^{v}(\vec{U})-Q_{1}^{o}\left({\overset{ˇ}{H}}_{1};{\hat{\theta }}_{1}\right)+Q_{2-}^{o}\left\{H_{2};{\hat{\theta }}_{2}\right\}\right]\;\;+{\hat{\mu }}_{3\omega_{2}}^{v}(\vec{U})-{\hat{\beta }}_{21}{\hat{\mu }}_{2\omega_{2}}^{v}(\vec{U})-Q_{2-}^{o}\left(H_{2};{\hat{\theta }}_{2}\right){\hat{\mu }}_{\omega_{2}}^{v}(\vec{U}),\;{𝒱}_{\bar{\Theta },\hat{\mu }}(\vec{U})\equiv Q_{1}^{o}\left({\overset{ˇ}{H}}_{1};{\bar{\theta }}_{1}\right)+\omega_{1}\left({\overset{ˇ}{H}}_{1},A_{1},\bar{\Theta }\right)\left[\left(1+{\bar{\beta }}_{21}\right){\hat{\mu }}_{2}^{v}(\vec{U})-Q_{1}^{o}\left({\overset{ˇ}{H}}_{1};{\bar{\theta }}_{1}\right)+Q_{2-}^{o}\left\{H_{2};{\bar{\theta }}_{2}\right\}\right]\;\;+{\hat{\mu }}_{3\omega_{2}}^{v}(\vec{U})-{\bar{\beta }}_{21}{\hat{\mu }}_{2\omega_{2}}^{v}(\vec{U})-Q_{2-}^{o}\left(H_{2};{\bar{\theta }}_{2}\right){\hat{\mu }}_{\omega_{2}}^{v}(\vec{U}).$$

We next replace the estimated imputation functions with their limits ${\overset{‾}{\mu }}_{2}^{v},{\overset{‾}{\mu }}_{2\omega_{2}}^{v},{\overset{‾}{\mu }}_{3\omega_{2}}^{v}$ and ${\overset{‾}{\mu }}_{\omega_{2}}^{v}$, and define:

(15)
$${𝒱}_{\hat{\Theta },\bar{\mu }}(\vec{U})\equiv Q_{1}^{o}\left({\overset{ˇ}{H}}_{1};{\hat{\theta }}_{1}\right)+\omega_{1}\left({\overset{ˇ}{H}}_{1},A_{1},\hat{\Theta }\right)\left[\left(1+{\hat{\beta }}_{21}\right){\bar{\mu }}_{2}^{v}(\vec{U})-Q_{1}^{o}\left({\overset{ˇ}{H}}_{1};{\hat{\theta }}_{1}\right)+Q_{2-}^{o}\left(H_{2};{\hat{\theta }}_{2}\right)\right]\;\;+{\bar{\mu }}_{3\omega_{2}}^{v}(\vec{U})-{\hat{\beta }}_{21}{\bar{\mu }}_{2\omega_{2}}^{v}(\vec{U})-Q_{2-}^{o}\left(H_{2};{\hat{\theta }}_{2}\right){\bar{\mu }}_{\omega_{2}}^{v}(\vec{U}),\;{𝒱}_{\bar{\Theta },\bar{\mu }}(\vec{U})\equiv Q_{1}^{o}\left({\overset{ˇ}{H}}_{1};{\bar{\theta }}_{1}\right)+\omega_{1}\left({\overset{ˇ}{H}}_{1},A_{1},\bar{\Theta }\right)\left[\left(1+{\bar{\beta }}_{21}\right){\bar{\mu }}_{2}^{v}(\vec{U})-Q_{1}^{o}\left({\overset{ˇ}{H}}_{1};{\bar{\theta }}_{1}\right)+Q_{2-}^{o}\left(H_{2};{\bar{\theta }}_{2}\right)\right]\;\;+{\bar{\mu }}_{3\omega_{2}}^{v}(\vec{U})-{\bar{\beta }}_{21}{\bar{\mu }}_{2\omega_{2}}^{v}(\vec{U})-Q_{2-}^{o}\left(H_{2};{\bar{\theta }}_{2}\right){\bar{\mu }}_{\omega_{2}}^{v}(\vec{U}).$$

Finally we define the following functions which are weighted sums of the imputation function errors:

(16)
$${ℰ}_{\hat{\Theta }}(\vec{U})\equiv \omega_{1}\left({\overset{ˇ}{H}}_{1},A_{1};\hat{\Theta }\right)\left(1+{\hat{\beta }}_{21}\right)\left\{{\hat{\mu }}_{2}^{v}(\vec{U})-{\bar{\mu }}_{2}^{v}(\vec{U})\right\}+{\hat{\mu }}_{3\omega_{2}}^{v}(\vec{U})-{\bar{\mu }}_{3\omega_{2}}^{v}(\vec{U})\;\;-{\hat{\beta }}_{21}\left\{{\hat{\mu }}_{2\omega_{2}}^{v}(\vec{U})-{\bar{\mu }}_{2\omega_{2}}^{v}(\vec{U})\right\}-Q_{2-}^{o}\left(H_{2};{\hat{\theta }}_{2}\right)\left\{{\hat{\mu }}_{\omega_{2}}^{v}(\vec{U})-{\bar{\mu }}_{\omega_{2}}^{v}(\vec{U})\right\},\;{ℰ}_{\bar{\Theta }}(\vec{U})\equiv \omega_{1}\left({\overset{ˇ}{H}}_{1},A_{1};\bar{\Theta }\right)\left(1+{\bar{\beta }}_{21}\right)\left\{{\hat{\mu }}_{2}^{v}(\vec{U})-{\bar{\mu }}_{2}^{v}(\vec{U})\right\}+{\hat{\mu }}_{3\omega_{2}}^{v}(\vec{U})-{\bar{\mu }}_{3\omega_{2}}^{v}(\vec{U})\;\;-{\bar{\beta }}_{21}\left\{{\hat{\mu }}_{2\omega_{2}}^{v}(\vec{U})-{\bar{\mu }}_{2\omega_{2}}^{v}(\vec{U})\right\}-Q_{2-}^{o}\left(H_{2};{\bar{\theta }}_{2}\right)\left\{{\hat{\mu }}_{\omega_{2}}^{v}(\vec{U})-{\bar{\mu }}_{\omega_{2}}^{v}(\vec{U})\right\}.$$

These definitions will come in handy in the following proofs as we can use them to write ${𝒱}_{\hat{\Theta },\hat{\mu }}(\vec{U})={𝒱}_{\hat{\Theta },\overset{‾}{\mu }}(\vec{U})+{ℰ}_{\hat{\Theta }}(\vec{U}),{𝒱}_{\bar{\Theta },\hat{\mu }}(\vec{U})={𝒱}_{\bar{\Theta },\overset{‾}{\mu }}(\vec{U})+{ℰ}_{\bar{\Theta }}(\vec{U})$. Finally, recalling that ${\mathbb{P}}_{\vec{U}}$ is the underlying distribution of the data, we define function $g_{1}:\Theta \mapsto \mathbb{R}$ as

$$g_{1}(\Theta )=\int {𝒱}_{\Theta ,\bar{\mu }}(\vec{U})d{\mathbb{P}}_{\vec{U}}.$$

With the above definitions we proceed by stating three lemmas that will be used to prove Theorem 7. We defer the proofs of these lemmas for after proving the main Theorem in this section.

**Lemma 11** *Under Assumptions 1-6, we have*

$$I)\;\sqrt{n}\left\{{\mathbb{P}}_{N}\left[{𝒱}_{\bar{\Theta },\bar{\mu }}\right]-g_{1}(\bar{\Theta })\right\}=o_{\mathbb{P}}(1),$$

$$II)\;\sqrt{n}\left\{g_{1}(\hat{\Theta })-g_{1}(\bar{\Theta })\right\}=\frac{1}{\sqrt{n}}\sum_{i=1}^{n}\left\{{\left(\frac{\partial }{\partial \theta }g_{1}(\bar{\Theta })\right)}^{⊤}\psi^{\theta }\left({\vec{U}}_{i}\right)+{\left(\frac{\partial }{\partial \xi }g_{1}(\bar{\Theta })\right)}^{⊤}\psi^{\xi }\left({\vec{U}}_{i}\right)\right\}\;\;+o_{\mathbb{P}}(1).$$

**Lemma 12** *Under Assumptions 1-6, the following holds:*

$$\sqrt{n}\left\{\left({\mathbb{P}}_{N}\left[{𝒱}_{\hat{\Theta },\bar{\mu }}\right]-g_{1}(\hat{\Theta })\right)-\left({\mathbb{P}}_{N}\left[{𝒱}_{\bar{\Theta },\bar{\mu }}\right]-g_{1}(\bar{\Theta })\right)\right\}=o_{\mathbb{P}}(1).$$

**Lemma 13** *Under Assumptions 1-6, the following assertions hold:*

$$I)\;\sqrt{n}{\mathbb{P}}_{N}\left\{{ℰ}_{\hat{\text{Θ}}}-{ℰ}_{\bar{\text{Θ}}}\right\}=o_{\mathbb{P}}(1),$$

$$II)\;\sqrt{n}{\mathbb{P}}_{N}\left[{ℰ}_{\bar{\Theta }}\right]={𝔾}_{n}\left\{\nu_{{\text{SSL}}_{\text{DR}}}(L;\bar{\Theta })\right\}\;\;+{\frac{1}{\sqrt{n}}\sum_{i=1}^{n}\psi^{\theta }{\left(L_{i}\right)}^{⊤}\frac{\partial }{\partial \theta }\int \nu_{{\text{SSL}}_{\text{DR}}}\left(L_{i};\Theta \right)d{\mathbb{P}}_{L}|}_{\Theta =\bar{\Theta }}\;\;+{\frac{1}{\sqrt{n}}\sum_{i=1}^{n}\psi^{\xi }{\left(L_{i}\right)}^{⊤}\frac{\partial }{\partial \xi }\int \nu_{{\text{SSL}}_{\text{DR}}}\left(L_{i};\Theta \right)d{\mathbb{P}}_{L}|}_{\Theta =\bar{\Theta }}\;\;+o_{\mathbb{P}}(1).$$

**Proof** [Proof of Theorem 7. We start by expanding the expression in (7) and using definitions (14), (15), (16):

$$\sqrt{n}\left\{{\mathbb{P}}_{N}\left[{𝒱}_{\hat{\Theta },\hat{\mu }}\right]-{𝔼}_{𝕊}\left[{𝒱}_{\bar{\Theta },\bar{\mu }}\right]\right\}\;=\sqrt{n}\left\{\underset{\left(I\right)}{\underset{︸}{{\mathbb{P}}_{N}\left[{𝒱}_{\bar{\Theta },\bar{\mu }}\right]+{\mathbb{P}}_{N}\left[{ℰ}_{\bar{\Theta }}\right]}}-\underset{\left(II\right)}{\underset{︸}{g_{1}(\bar{\Theta })-{𝔼}_{𝕊}\left[{ℰ}_{\bar{\Theta }}\right]}}\right\}\;+\sqrt{n}\left\{({\mathbb{P}}_{N}\left[{𝒱}_{\hat{\Theta },\bar{\mu }}\right]+{\mathbb{P}}_{N}\left[{ℰ}_{\hat{\Theta }}\right]-\underset{\left(III\right)}{\underset{︸}{g_{1}(\hat{\Theta }))-{𝔼}_{𝕊}\left[{ℰ}_{\hat{\Theta }}\right]}}\right\}\;-\left\{\underset{\left(I\right)}{\underset{︸}{{\mathbb{P}}_{N}\left[{𝒱}_{\bar{\Theta },\bar{\mu }}\right]+{\mathbb{P}}_{N}\left[{ℰ}_{\bar{\Theta }}\right]}}-\underset{\left(II\right)}{\underset{︸}{g_{1}(\bar{\Theta })-{𝔼}_{𝕊}\left[{ℰ}_{\bar{\Theta }}\right]}}\right\}\;+\sqrt{n}\left\{\underset{\left(III\right)}{\underset{︸}{g_{1}(\hat{\Theta })+{𝔼}_{𝕊}\left[{ℰ}_{\hat{\Theta }}\right]}}-{𝔼}_{𝕊}\left[{𝒱}_{\bar{\Theta },\bar{\mu }}\right]\right\}\;=\sqrt{n}\left\{{\mathbb{P}}_{N}\left[{𝒱}_{\bar{\Theta },\bar{\mu }}\right]-g_{1}(\bar{\Theta })\right\}\;+\sqrt{n}\left\{g_{1}(\hat{\Theta })-g_{1}(\bar{\Theta })\right\}\;+\sqrt{n}\left\{\left({\mathbb{P}}_{N}\left[{𝒱}_{\hat{\Theta },\bar{\mu }}\right]-g_{1}(\hat{\Theta })\right)-\left({\mathbb{P}}_{N}\left[{𝒱}_{\bar{\Theta },\bar{\mu }}\right]-g_{1}(\bar{\Theta })\right)\right\}\;+\sqrt{n}{\mathbb{P}}_{N}\left[{ℰ}_{\hat{\Theta }}-{ℰ}_{\bar{\Theta }}\right]\;+\sqrt{n}{\mathbb{P}}_{N}\left[{ℰ}_{\bar{\Theta }}\right]\;=\frac{1}{\sqrt{n}}\sum_{i=1}^{n}\psi_{{\text{SSL}}_{\mathrm{DR}}}^{v}\left(L_{i};\bar{\Theta }\right)+o_{\mathbb{P}}(1).$$

which follows from Lemmas 11, 12 & 13 with the influence function $\psi_{{\text{SSL}}_{\text{DR}}}^{v}$ defined as

$$\psi_{{\text{SSL}}_{\text{DR}}}^{v}(L;\bar{\Theta })=\nu_{{\text{SSL}}_{\text{DR}}}(L;\bar{\Theta })+{\psi^{\theta }{\left(L\right)}^{⊤}\frac{\partial }{\partial \theta }\int \left\{{𝒱}_{\Theta ,\bar{\mu }}(L)+\nu_{{\text{SSL}}_{\text{DR}}}(L;\Theta )\right\}\;d{\mathbb{P}}_{L}|}_{\Theta =\bar{\Theta }}\;\;+{\psi^{\xi }{\left(L\right)}^{⊤}\frac{\partial }{\partial \xi }\int \left\{{𝒱}_{\Theta ,\bar{\mu }}(\vec{U})+\nu_{{\text{SSL}}_{\text{DR}}}(L;\Theta )\right\}\;d{\mathbb{P}}_{L}|}_{\Theta =\bar{\Theta }},$$

$$\nu_{{\text{SSL}}_{\text{DR}}}(L;\bar{\Theta })=\omega_{1}\left({\overset{ˇ}{H}}_{1},A_{1};{\bar{\Theta }}_{1}\right)\left(1+{\bar{\beta }}_{21}\right)\left\{Y_{2}-{\bar{\mu }}_{2}^{v}(\vec{U})\right\}+\omega_{2}\left({\overset{ˇ}{H}}_{2},A_{2},{\bar{\Theta }}_{2}\right)Y_{3}-{\bar{\mu }}_{3\omega_{2}}(\vec{U})\;\;-{\bar{\beta }}_{21}\left\{\omega_{2}\left({\overset{ˇ}{H}}_{2},A_{2},{\bar{\Theta }}_{2}\right)Y_{2}-{\bar{\mu }}_{2\omega_{2}}(\vec{U})\right\}-Q_{2-}^{o}\left(H_{2};{\bar{\theta }}_{2}\right)\left\{\omega_{2}\left({\overset{ˇ}{H}}_{2},A_{2},{\bar{\Theta }}_{2}\right)-{\bar{\mu }}_{\omega_{2}}(\vec{U})\right\}$$

Next note that

$$\int {\left({𝒱}_{\Theta ,\bar{\mu }}(\vec{U})+\nu_{{\text{SSL}}_{\text{DR}}}(L;\Theta )\right)d{\mathbb{P}}_{L}|}_{\Theta =\bar{\Theta }}=\int {𝒱}_{{\text{SUP}}_{\text{DR}}}(L;\Theta ){d{\mathbb{P}}_{L}|}_{\Theta =\bar{\Theta }},$$

where ${𝒱}_{{SUP}_{DR}}(L;\Theta )$ is defined in (5). Finally, all random variables in the expression of $\psi_{{SS}_{L_{DR}}}^{v}(L;\bar{\Theta })$ are bounded by Assumptions 1 and 5 we have $𝔼\left[\psi_{{SSL}_{DR}}^{v}(L;\bar{\Theta }{)}^{2}\right]<\infty$, the central limit theorem yields that

$$\sqrt{n}\left\{{\mathbb{P}}_{N}\left[{𝒱}_{\hat{\Theta },\hat{\mu }}\right]-g_{1}(\bar{\Theta })\right\}=\frac{1}{\sqrt{n}}\sum_{i=1}^{n}\psi_{{\text{SSL}}_{\text{DR}}}^{v}\left(L_{i};\bar{\Theta }\right)+o_{\mathbb{P}}(1)\overset{d}{\to }N\left(0,\sigma_{{\text{SSL}}_{\text{DR}}}^{2}\right).$$

■

**Proof** [Proof of Lemma 11 I) We start with $\sqrt{n}\left\{{\mathbb{P}}_{N}\left[{𝒱}_{\bar{\Theta },\overset{‾}{\mu }}\right]-g_{1}(\bar{\Theta })\right\}$. Note that ${𝒱}_{\bar{\Theta },\overset{‾}{\mu }}(\vec{U})$ is a deterministic function of random variable $\vec{U}$ as parameters and imputation functions are fixed. We have that $𝔼\left[{𝒱}_{\overset{‾}{\Theta },\overset{‾}{\mu }}(\vec{U}{)}^{2}\right]<\infty$ holds by Assumption 1 & 5. Thus the central limit theorem yields ${𝔾}_{N}\left\{{𝒱}_{\bar{\Theta },\overset{‾}{\mu }}\right\}\overset{d}{\to }𝒩\left(0,\mathrm{Var}\left[{𝒱}_{\bar{\Theta },\overset{‾}{\mu }}\right]\right)$, therefore

$$\sqrt{n}\left\{{\mathbb{P}}_{N}\left[{𝒱}_{\bar{\Theta },\bar{\mu }}\right]-g_{1}(\bar{\Theta })\right\}=\sqrt{\frac{n}{N}}{𝔾}_{N}\left\{{𝒱}_{\bar{\Theta },\bar{\mu }}\right\}=O_{\mathbb{P}}\left(\frac{\sqrt{n}}{N}\right)=o_{\mathbb{P}}(1).$$

II) We next consider $\sqrt{n}\left\{g_{1}(\hat{\Theta })-g_{1}(\bar{\Theta })\right\}$. Using a Taylor series expansion

$$g_{1}(\hat{\Theta })=g_{1}(\bar{\Theta })+{\left(\hat{\theta }-\bar{\theta }\right)}^{⊤}\frac{\partial }{\partial \theta }g_{1}(\bar{\Theta })+{\left(\hat{\xi }-\bar{\xi }\right)}^{⊤}\frac{\partial }{\partial \xi }g_{1}(\bar{\Theta })+O_{\mathbb{P}}\left(n^{-1}\right),$$

as both $\|\hat{\theta }-\bar{\theta }{\|}_{2}^{2}=O_{\mathbb{P}}\left(n^{-1}\right)$ and $\|\hat{\xi }-\bar{\xi }{\|}_{2}^{2}=O_{\mathbb{P}}\left(n^{-1}\right)$ by Theorems 2, 3 and Assumption 5, therefore

$$\sqrt{n}\left\{g_{1}(\hat{\Theta })-g_{1}(\bar{\Theta })\right\}=\sqrt{n}{\left(\hat{\theta }-\bar{\theta }\right)}^{⊤}\frac{\partial }{\partial \theta }g_{1}(\bar{\Theta })+\sqrt{n}{\left(\hat{\xi }-\bar{\xi }\right)}^{⊤}\frac{\partial }{\partial \xi }g_{1}(\bar{\Theta })+o_{\mathbb{P}}(1).$$

We can write

$$\sqrt{n}\left\{g_{1}(\hat{\Theta })-g_{1}(\bar{\Theta })\right\}=\frac{\partial }{\partial \theta }g_{1}(\bar{\Theta })\frac{1}{\sqrt{n}}\sum_{i=1}^{n}\psi^{\theta }\left({\vec{U}}_{i}\right)+\frac{\partial }{\partial \xi }g_{1}(\bar{\Theta })\frac{1}{\sqrt{n}}\sum_{i=1}^{n}\psi^{\xi }\left({\vec{U}}_{i}\right)+o_{\mathbb{P}}(1).$$

■

**Proof** [Proof of Lemma 12]

We consider $\sqrt{n}\left\{\left({\mathbb{P}}_{N}\left[{𝒱}_{\hat{\Theta },\overset{‾}{\mu }}\right]-g_{1}(\hat{\Theta })\right)-\left({\mathbb{P}}_{N}\left[{𝒱}_{\bar{\Theta },\overset{‾}{\mu }}\right]-g_{1}(\bar{\Theta })\right)\right\}$, recall that $d_{t}({\overset{ˇ}{H}}_{t},\theta_{t})=I(H_{t1}^{⊤}\gamma_{t}>0)t=1,2$, thus the inverse probability weight functions are defined as

$$\omega_{1}\left({\overset{ˇ}{H}}_{1},A_{1},\Theta \right)\equiv \frac{I\left(H_{11}^{⊤}\gamma_{1}>0\right)A_{1}}{\pi_{1}\left({\overset{ˇ}{H}}_{1};\xi_{1}\right)}+\frac{\left\{1-I\left(H_{11}^{⊤}\gamma_{1}>0\right)\right\}\left\{1-A_{1}\right\}}{1-\pi_{1}\left({\overset{ˇ}{H}}_{1};\xi_{1}\right)},\;\text{and}$$

$$\omega_{2}\left({\overset{ˇ}{H}}_{2},A_{2},\Theta \right)\equiv \omega_{1}\left({\overset{ˇ}{H}}_{1},A_{1},\Theta \right)\left(\frac{I\left(H_{21}^{⊤}\gamma_{2}>0\right)A_{2}}{\pi_{2}\left({\overset{ˇ}{H}}_{2};\xi_{2}\right)}+\frac{\left\{1-I\left(H_{21}^{⊤}\gamma_{2}>0\right)\right\}\left\{1-A_{2}\right\}}{1-\pi_{2}\left({\overset{ˇ}{H}}_{2};\xi_{2}\right)}\right).$$

Define the class

$$\ell_{t}=\left\{I\left(H_{t}^{⊤}\gamma_{t}\geq 0\right):{ℋ}_{t1},\gamma \in {\mathbb{R}}^{q_{t}}\right\},\;t=1,2$$

and the collection of half spaces ${𝒞}_{\ell }\equiv \left\{H_{t}\in {\mathbb{R}}^{q_{t}}:H_{t}^{⊤}\gamma_{t}\geq 0,\gamma \in {\mathbb{R}}^{q_{t}},t\in \{1,2\}\right\}$. By Dudley (1979) ${𝒞}_{\ell }$ is a VC class of VC dimension $q_{t}+1$. Next by van der Vart and Wellner (1996) we have that as ${𝒞}_{\ell }$ is a VC-class $\ell_{t}$ is a class of the same index. Finally, by Theorem 2.6.7 we have that $\ell_{t}$ is a ℙ-Donsker class. Next define the following function

$$f_{\Theta }(\vec{U})=Q_{1}^{o}\left({\overset{ˇ}{H}}_{1};\theta_{1}\right)+\omega_{1}\left({\overset{ˇ}{H}}_{1},A_{1},\Theta \right)\left[\left(1+\beta_{21}\right){\bar{\mu }}_{2}^{v}(\vec{U})-Q_{1}^{o}\left({\overset{ˇ}{H}}_{1};\theta_{1}\right)+Q_{2-}^{o}\left(H_{2};\theta_{2}\right)\right]+{\bar{\mu }}_{3\omega_{2}}^{v}(\vec{U})-\beta_{21}{\bar{\mu }}_{2\omega_{2}}^{v}(\vec{U})-Q_{2-}^{o}\left(H_{2};\theta_{2}\right){\bar{\mu }}_{\omega_{2}}^{v}(\vec{U}).$$

We define the associated class of functions ${𝒞}_{1}=\{f_{\Theta }(\vec{U})|\vec{U},\Theta \in 𝒮(\delta )\}$.

i) By Assumptions 3, 5 and Theorem 19.5 in Vart (1998), $\ell_{t}$, ${𝒲}_{t}$, ${𝒬}_{t}$, t=1,2 are ℙ-Donsker classes. Thus it follows that ${𝒞}_{1}$ is a Donsker class.

ii) We estimate $\xi_{1}$, $\xi_{2}$ for (4) with their maximum likelihood estimators, ${\hat{\xi }}_{1}$, ${\hat{\xi }}_{2}$, solving ${\mathbb{P}}_{n}\left[S_{t}\left(\xi_{t}\right)\right]=0$, t=1,2. By Assumption (5) and Theorem 5.9 in Vart (1998) ${\hat{\xi }}_{t}\overset{p}{\to }{\bar{\xi }}_{t}$, t=1,2. Next, by Theorems 2, 3, under Assumptions 1, 2, ${\hat{\theta }}_{t}\overset{p}{\to }{\bar{\theta }}_{t}$, t=1,2. Thus $\mathbb{P}(\hat{\Theta }\in 𝒮(\delta ))\to 1$, ∀δ.

iii) We next show $\int {\left({𝒱}_{\hat{\Theta },\overset{‾}{\mu }}-{𝒱}_{\bar{\Theta },\overset{‾}{\mu }}\right)}^{2}d{\mathbb{P}}_{\vec{U}}⟶0$. By Assumptions 5 (ii), 6, and bounded covariates and there exists a constant c∈ℝ such that we can write

$$\int {\left({𝒱}_{\hat{\Theta },\bar{\mu }}-{𝒱}_{\bar{\Theta },\bar{\mu }}\right)}^{2}d{\mathbb{P}}_{\vec{U}}\;\leq \int {\left(Q_{1}^{o}\left(H_{1};{\hat{\theta }}_{1}\right)-Q_{1}^{o}\left(H_{1};{\bar{\theta }}_{1}\right)\right)}^{2}d{\mathbb{P}}_{\vec{U}}\;+c\int {\left(\frac{1}{1-\pi_{1}\left(H_{1};{\hat{\xi }}_{1}\right)}-\frac{1}{1-\pi_{1}\left(H_{1};{\bar{\xi }}_{1}\right)}\right)}^{2}d{\mathbb{P}}_{\vec{U}}\;+c\int {\left(\frac{1}{\pi_{1}\left(H_{1};{\hat{\xi }}_{1}\right)}-\frac{1}{\pi_{1}\left(H_{1};{\bar{\xi }}_{1}\right)}\right)}^{2}\;+c\int {\left\{Q_{2-}^{o}\left({\overset{ˇ}{H}}_{2};{\hat{\theta }}_{2}\right)-Q_{2-}^{o}\left(H_{2};{\bar{\theta }}_{2}\right)\right\}}^{2}d{\mathbb{P}}_{\vec{U}}\;+c\int {\left\{I\left(H_{11}^{⊤}{\hat{\gamma }}_{1}>0\right)-I\left(H_{11}^{⊤}{\bar{\gamma }}_{1}>0\right)\right\}}^{2}d{\mathbb{P}}_{\vec{U}}\;+{\left({\hat{\beta }}_{21}-{\bar{\beta }}_{21}\right)}^{2}\;+c\int \;{\left(H_{20}^{⊤}{\bar{\beta }}_{22}+{\left[H_{20}^{⊤}{\bar{\gamma }}_{2}\right]}_{+}-H_{20}^{⊤}{\hat{\beta }}_{22}-{\left[H_{20}^{⊤}{\hat{\gamma }}_{2}\right]}_{+}\right)}^{2}d{\mathbb{P}}_{\vec{U}}$$

where we use $(a-b{)}^{2}$, $(a+b{)}^{2}\leq 2a^{2}+2b^{2}\forall a$, b∈ℝ, ${\hat{d}}_{1}$, $A_{1}\leq 1$ for all H∈ℋ, and boundedness of ${\hat{\theta }}_{t}$, t=1,2 by Assumptions 1-3. Next note that all terms outside integrals are bounded by Assumptions 1-3. Finally we consider terms within the integrals with the following example

$$\int {\left(Q_{2-}^{o}\left(H_{2};{\hat{\theta }}_{2}\right)-Q_{2-}^{o}\left(H_{2};\bar{\theta }\right)\right)}^{2}d{\mathbb{P}}_{\vec{U}}\;=\int {\left(H_{20}^{⊤}{\hat{\beta }}_{22}+{\left[H_{21}^{⊤}{\hat{\gamma }}_{2}\right]}_{+}-H_{20}^{⊤}{\bar{\beta }}_{22}-{\left[H_{21}^{⊤}{\bar{\gamma }}_{2}\right]}_{+}\right)}^{2}d{\mathbb{P}}_{\vec{U}}\;=4{\left\|{\hat{\beta }}_{22}-{\bar{\beta }}_{22}\right\|}_{2}^{2}\int H_{20}^{⊤}H_{20}d{\mathbb{P}}_{\vec{U}}\;+4{\left\|{\hat{\gamma }}_{2}-{\bar{\gamma }}_{2}\right\|}_{2}^{2}\int H_{21}^{⊤}H_{21}d{\mathbb{P}}_{\vec{U}}=O_{\mathbb{P}}\left(n^{-1}\right),$$

which follows from Theorem 2 and Lemma 17 (a). All similar terms can be handled accordingly. We get the convergence in probability to $0:\int {\left({𝒱}_{\hat{\Theta },\overset{‾}{\mu }}-{𝒱}_{\bar{\Theta },\overset{‾}{\mu }}\right)}^{2}d{\mathbb{P}}_{\vec{U}}\to 0$ as all other terms within expectation are $O_{\mathbb{P}}\left(n^{-1}\right)$ by the dominating convergence theorem, boundedness conditions as stated in Assumptions 2, 5, and the consistency of $\hat{\xi }$ and $\hat{\theta }$ as $\mathbb{P}(\hat{\Theta }\in 𝒮(\delta ))\to 1$, ∀δ>0.

Finally, we have i) $\mathbb{P}(\hat{\Theta }\in 𝒮(\delta ))\to 1$, ii) ${𝒞}_{1}$ is a Donsker class, and iii) $\int {\left({𝒱}_{\hat{\Theta },\overset{‾}{\mu }}-{𝒱}_{\bar{\Theta },\overset{‾}{\mu }}\right)}^{2}d{\mathbb{P}}_{\vec{U}}⟶0$, then by Theorem 2.1 in Van Der Vaart and Wellner (2007),

$$\sqrt{\frac{n}{N}}\sqrt{n}\left\{\left({\mathbb{P}}_{N}\left[{𝒱}_{\hat{\Theta },\bar{\mu }}\right]-g_{1}(\hat{\Theta })\right)-\left({\mathbb{P}}_{N}\left[{𝒱}_{\bar{\Theta },\bar{\mu }}\right]-g_{1}(\bar{\Theta })\right)\right\}=\sqrt{\frac{n}{N}}o_{\mathbb{P}}(1).$$

■

**Proof** [Proof of Lemma 13]I) First note that from the empirical normal equations (6), we have that the solution ${\hat{\eta }}_{2}^{v}$ satisfies ${\hat{\eta }}_{2}^{v}-\eta_{2}^{v}=O_{\mathbb{P}}\left(n^{-\frac{1}{2}}\right)$. Therefore

$$\underset{\vec{U}}{\sup}\left|{\hat{\mu }}_{2}^{v}(\vec{U})-\mu_{2}^{v}(\vec{U})\right|=\underset{\vec{U}}{\sup}\left|\frac{1}{K}{\hat{m}}_{2}^{\left(-k\right)}(\vec{U})+{\hat{\eta }}_{2}^{v}-m_{2}(\vec{U})+\eta_{2}^{v}\right|\;\;\leq \frac{1}{K}\underset{\vec{U}}{\sup}\left|{\hat{m}}_{2}^{\left(-k\right)}(\vec{U})+m_{2}(\vec{U})\right|+\left|{\hat{\eta }}_{2}^{v}-\eta_{2}^{v}\right|\;\;=o_{\mathbb{P}}(1)+O_{\mathbb{P}}\left(n^{-\frac{1}{2}}\right)=o_{\mathbb{P}}(1),$$

where we additionally use Assumption 6 for the difference of estimated and true imputation models ${\overset{ˆ}{m}}_{2}$, $m_{2}$. Similarly $\underset{\vec{U}}{sup}\;\left|{\hat{\mu }}_{t\omega_{2}}^{v}(\vec{U})-{\overset{‾}{\mu }}_{t\omega_{2}}^{v}(\vec{U})\right|=o_{\mathbb{P}}(1)$, $\underset{\vec{U}}{sup}\;\left|{\hat{\mu }}_{\omega_{2}}^{v}(\vec{U})-{\overset{‾}{\mu }}_{\omega_{2}}^{v}(\vec{U})\right|=o_{\mathbb{P}}(1)$, t=2,3. Next, using the triangle and Jensen’s inequalities, we have

$${\mathbb{P}}_{N}\left[{ℰ}_{\hat{\Theta }}-{ℰ}_{\bar{\Theta }}\right]\;\leq {\mathbb{P}}_{N}\left|\omega_{1}\left({\overset{ˇ}{H}}_{1},A_{1};{\hat{\Theta }}_{1}\right)\left(1+{\hat{\beta }}_{21}\right)-\omega_{1}\left({\overset{ˇ}{H}}_{1},A_{1};{\bar{\Theta }}_{1}\right)\left(1+{\bar{\beta }}_{21}\right)\right|\underset{\vec{U}}{\sup}\left|{\hat{\mu }}_{2}^{v}(\vec{U})-{\bar{\mu }}_{2}^{v}(\vec{U})\right|\;+\left|{\hat{\beta }}_{21}-{\bar{\beta }}_{21}\right|\underset{\vec{U}}{\sup}\left|{\hat{\mu }}_{2\omega_{2}}(\vec{U})-\mu_{2\omega_{2}}(\vec{U})\right|\;+{\mathbb{P}}_{N}\left|Q_{2-}^{o}\left(H_{2};{\hat{\theta }}_{2}\right)-Q_{2-}^{o}\left(H_{2};{\bar{\theta }}_{2}\right)\right|\underset{\vec{U}}{\sup}\left|{\hat{\mu }}_{\omega_{2}}(\vec{U})-{\bar{\mu }}_{\omega_{2}}(\vec{U})\right|\;\leq {\mathbb{P}}_{N}\left|\omega_{1}\left({\overset{ˇ}{H}}_{1},A_{1};{\hat{\Theta }}_{1}\right)-\omega_{1}\left({\overset{ˇ}{H}}_{1},A_{1};{\bar{\Theta }}_{1}\right)\right|o_{\mathbb{P}}(1)\;+{\mathbb{P}}_{N}\left|\omega_{1}\left({\overset{ˇ}{H}}_{1},A_{1};{\hat{\Theta }}_{1}\right){\hat{\beta }}_{21}-\omega_{1}\left({\overset{ˇ}{H}}_{1},A_{1};{\bar{\Theta }}_{1}\right){\bar{\beta }}_{21}\right|o_{\mathbb{P}}(1)\;+\left|{\hat{\beta }}_{21}-{\bar{\beta }}_{21}\right|o_{\mathbb{P}}(1)+{\mathbb{P}}_{N}\left|{\left({\hat{\beta }}_{22}-{\hat{\beta }}_{22}\right)}^{⊤}H_{20}+{\left[{\hat{\gamma }}_{2}^{⊤}H_{21}\right]}_{+}-\left[{\bar{\gamma }}_{2}^{⊤}H_{21}\right]+\right|o_{\mathbb{P}}(1).$$

By Theorem 2 we have ${\hat{\theta }}_{2}-{\bar{\theta }}_{2}=O_{\mathbb{P}}\left(n^{-\frac{1}{2}}\right)$, also from Lemma 17 (a) it follows that ${\mathbb{P}}_{N}\left({\left[H_{21}^{⊤}{\hat{\gamma }}_{2}\right]}_{+}-{\left[H_{21}^{⊤}{\overset{‾}{\gamma }}_{2}\right]}_{+}\right)=O_{\mathbb{P}}\left(n^{-\frac{1}{2}}\right)$, hence as covariates are bounded we have

$$\left|{\hat{\beta }}_{21}-{\bar{\beta }}_{21}\right|o_{\mathbb{P}}(1)+{\mathbb{P}}_{N}\left|{\left({\hat{\beta }}_{22}-{\hat{\beta }}_{22}\right)}^{⊤}H_{20}+{\left[{\hat{\gamma }}_{2}^{⊤}H_{21}\right]}_{+}-{\left[{\bar{\gamma }}_{2}^{⊤}H_{21}\right]}_{+}\right|\;\leq \left\{o_{\mathbb{P}}(1)+\underset{H_{20}}{\sup}{\left\|H_{20}\right\|}_{2}{\left\|{\hat{\beta }}_{22}-{\bar{\beta }}_{22}\right\|}_{2}+\underset{H_{21}}{\sup}{\left\|H_{21}\right\|}_{2}\right\}O_{\mathbb{P}}\left(n^{-\frac{1}{2}}\right)=O_{\mathbb{P}}\left(n^{-\frac{1}{2}}\right).$$

Next, we can write

$$\omega_{1}\left(H_{1},A_{1};{\hat{\Theta }}_{1}\right)=I\left\{A_{1}=d_{1}\left(H_{1};{\hat{\xi }}_{1}\right)\right\}\left\{\frac{A_{1}}{\pi_{1}\left(H_{1};{\hat{\xi }}_{1}\right)}+\frac{1-A_{1}}{1-\pi_{1}\left(H_{1};{\hat{\xi }}_{1}\right)}\right\}.$$

By Lemma 17 (b) it follows that

$${\mathbb{P}}_{N}\left[I\left\{A_{1}=d_{1}\left(H_{1};{\hat{\xi }}_{1}\right)\right\}-I\left\{A_{1}=d_{1}\left(H_{1};{\bar{\xi }}_{1}\right)\right\}\right]=O_{\mathbb{P}}\left(n^{-\frac{1}{2}}\right),$$

$${\mathbb{P}}_{N}\left[\frac{A_{1}}{\pi_{1}\left(H_{1};{\hat{\xi }}_{1}\right)}-\frac{A_{1}}{\pi_{1}\left(H_{1};{\bar{\xi }}_{1}\right)}\right]=O_{\mathbb{P}}\left(n^{-\frac{1}{2}}\right),$$

$${\mathbb{P}}_{N}\left[\frac{1-A_{1}}{1-\pi_{1}\left(H_{1};{\hat{\xi }}_{1}\right)}-\frac{1-A_{1}}{1-\pi_{1}\left(H_{1};{\bar{\xi }}_{1}\right)}\right]=O_{\mathbb{P}}\left(n^{-\frac{1}{2}}\right).$$

Using the above and Lemma 14 we get

$${\hat{\beta }}_{21}{\mathbb{P}}_{N}\left\{\omega_{1}\left({\overset{ˇ}{H}}_{1},A_{1};{\hat{\Theta }}_{1}\right)\right\}-{\bar{\beta }}_{21}{\mathbb{P}}_{N}\left\{\omega_{1}\left({\overset{ˇ}{H}}_{1},A_{1};{\bar{\Theta }}_{1}\right)\right\}=O_{\mathbb{P}}\left(n^{-\frac{1}{2}}\right),$$

$${\mathbb{P}}_{N}\left\{\omega_{1}\left({\overset{ˇ}{H}}_{1},A_{1};{\hat{\Theta }}_{1}\right)\right\}-{\mathbb{P}}_{N}\left\{\omega_{1}\left({\overset{ˇ}{H}}_{1},A_{1};{\bar{\Theta }}_{1}\right)\right\}=O_{\mathbb{P}}\left(n^{-\frac{1}{2}}\right).$$

From the above we get

$${\mathbb{P}}_{N}\left\{{ℰ}_{\hat{\text{Θ}}}-{ℰ}_{\bar{\text{Θ}}}\right\}=O_{\mathbb{P}}\left(n^{-\frac{1}{2}}\right)o_{\mathbb{P}}(1).$$

II) To show the relevant result, we first recall the definition of $\nu_{{\text{SSL}}_{\text{DR}}}$ from Theorem 7 and show that

(17)
$$\frac{1}{\sqrt{n}}\sum_{i=1}^{n}\nu_{{\text{SSL}}_{\text{DR}}}\left(L_{i};\hat{\Theta }\right)=\frac{1}{\sqrt{n}}\sum_{i=1}^{n}\nu_{{\text{SSL}}_{\text{DR}}}\left(L_{i};\bar{\Theta }\right)\;\;+\frac{1}{\sqrt{n}}\sum_{i=1}^{n}{\left(\frac{\partial }{\partial \theta }𝔼\left[\nu_{{\text{SSL}}_{\text{DR}}}\left(L_{i};\bar{\Theta }\right)\right]\right)}^{⊤}\psi^{\theta }\left(L_{i}\right)\;\;+\frac{1}{\sqrt{n}}\sum_{i=1}^{n}{\left(\frac{\partial }{\partial \xi }𝔼\left[\nu_{{\text{SSL}}_{\text{DR}}}\left(L_{i};\bar{\Theta }\right)\right]\right)}^{⊤}\psi^{\xi }\left(L_{i}\right)+o_{\mathbb{P}}(1).$$

We start expanding $\frac{1}{\sqrt{n}}\sum_{i=1}^{n}\;\nu_{{SSL}_{DR}}(L_{i};\hat{\Theta })$ as

$$\frac{1}{\sqrt{n}}\sum_{i=1}^{n}\nu_{{\text{SSL}}_{\text{DR}}}\left(L_{i};\hat{\Theta }\right)\;={𝔾}_{n}\left\{\nu_{{\text{SSL}}_{\text{DR}}}(L;\bar{\Theta })\right\}+{𝔾}_{n}\left\{\nu_{{\text{SSL}}_{\text{DR}}}(L;\hat{\Theta })-\nu_{{\text{SSL}}_{\text{DR}}}(L;\bar{\Theta })\right\}+\sqrt{n}\int \nu_{{\text{SSL}}_{\text{DR}}}(L;\hat{\Theta })d{\mathbb{P}}_{L},$$

we next consider the limit of each term above.

1) Using a Taylor series expansion on $\int \nu_{{\text{SSL}}_{\text{DR}}}(L;\hat{\Theta })d{\mathbb{P}}_{L}$ we get

$$\int \nu_{{\text{SSL}}_{\text{DR}}}(L;\hat{\Theta })d{\mathbb{P}}_{L}=\int \nu_{{\text{SSL}}_{\text{DR}}}(L;\bar{\Theta })d{\mathbb{P}}_{L}+{{\left(\hat{\Theta }-\bar{\Theta }\right)}^{⊤}\frac{\partial }{\partial \Theta }\int \nu_{{\text{SSL}}_{\text{DR}}}(L;\Theta )d{\mathbb{P}}_{L}|}_{\Theta =\bar{\Theta }}+O_{\mathbb{P}}\left(n^{-1}\right),$$

where the remaining terms are of order $O\left\{(\hat{\Theta }-\bar{\Theta }{)}^{2}\right\}$ which by Theorems 2 & 3 are $O_{\mathbb{P}}\left(n^{-1}\right)$. Next note that from (13) it follows that $\int \nu_{{\text{SSL}}_{\text{DR}}}(L;\bar{\Theta })d{\mathbb{P}}_{L}=0$, and thus letting $g_{2}(\Theta )=\int \;\nu_{{\text{SSL}}_{\text{DR}}}(L;\Theta )d{\mathbb{P}}_{L}$ we have

$$\sqrt{n}g_{2}(\hat{\Theta })={\sqrt{n}{\left(\hat{\theta }-\bar{\theta }\right)}^{⊤}\frac{\partial }{\partial \Theta }g_{2}(\Theta )|}_{\Theta =\bar{\Theta }}+{\sqrt{n}{\left(\hat{\xi }-\bar{\xi }\right)}^{⊤}\frac{\partial }{\partial \xi }g_{2}(\Theta )|}_{\Theta =\bar{\Theta }}+o_{\mathbb{P}}(1).$$

We can write

$$\sqrt{n}g_{2}(\hat{\Theta })={\frac{1}{\sqrt{n}}\sum_{i=1}^{n}\psi^{\theta }{\left(L_{i}\right)}^{⊤}\frac{\partial }{\partial \theta }g_{2}(\Theta )|}_{\Theta =\bar{\Theta }}+{\frac{1}{\sqrt{n}}\sum_{i=1}^{n}\psi^{\xi }{\left(L_{i}\right)}^{⊤}\frac{\partial }{\partial \xi }g_{2}(\Theta )|}_{\Theta =\bar{\Theta }}+o_{\mathbb{P}}(1).$$

2) We next show

$${𝔾}_{n}\left\{\nu_{{\text{SSL}}_{\text{DR}}}(L;\hat{\Theta })-\nu_{{\text{SSL}}_{\text{DR}}}(L;\bar{\Theta })\right\}=o_{\mathbb{P}}(1),$$

define the class

$$\ell_{t}=\left\{I\left(H^{⊤}\gamma_{t}\geq 0\right):{ℋ}_{t1},\gamma \in {\mathbb{R}}^{q_{t}}\right\},\;t=1,2$$

and the collection of half spaces ${𝒞}_{\ell }\equiv \left\{H_{t}\in {\mathbb{R}}^{q_{t}}:H^{⊤}\gamma_{t}\geq 0,\gamma \in {\mathbb{R}}^{q_{t}},t\in \{1,2\}\right\}$, by Dudley (1979) ${𝒞}_{\ell }$ is a VC class of VC dimension $q_{t}+1$, next by van der Vaart and Wellner (1996) we have that as ${𝒞}_{\ell }$ is a VC-class $\ell_{t}$ is a class of the same index. Finally, by Theorem 2.6.7 we have that $\ell_{t}$ is a Donsker class.

$$f_{\Theta }\left(L_{i}\right)=\omega_{1}\left({\overset{ˇ}{H}}_{1i},A_{1i};\Theta_{1}\right)\left(1+\beta_{21}\right)\left\{Y_{2i}-{\bar{\mu }}_{2}^{v}\left({\vec{U}}_{i}\right)\right\}+\omega_{2}\left({\overset{ˇ}{H}}_{2i},A_{2i};\Theta_{2}\right)Y_{3i}-{\bar{\mu }}_{3\omega_{2}}\left({\vec{U}}_{i}\right)\;\;-\beta_{21}\left\{\omega_{2}\left({\overset{ˇ}{H}}_{2i},A_{2i};\Theta_{2}\right)Y_{2i}-{\bar{\mu }}_{2\omega_{2}}\left({\vec{U}}_{i}\right)\right\}-Q_{2-}^{o}\left(H_{2i};\theta_{2}\right)\left\{\omega_{2}\left({\overset{ˇ}{H}}_{2i},A_{2i};\Theta_{2}\right)-{\bar{\mu }}_{\omega_{2}}\left({\vec{U}}_{i}\right)\right\},$$

we define the class of functions ${𝒞}_{2}=\left\{f_{\Theta }(L)|\Theta \in 𝒮(\delta )\right\}$.

i) By Assumptions 3, 5 and Theorem 19.5 in Vaart (1998), ${𝒲}_{t}$, ${𝒬}_{t}$, t=1,2 are a ℙ-Donsker class. Additionally, the terms in the $\omega_{t}\left(H_{t},A_{t};\Theta_{t}\right)$ functions of the form $H_{t1}^{⊤}\gamma_{t}I\left(H_{t1}^{⊤}\gamma_{t}>0\right)$ constitute a ℙ-Donsker class, as $H_{t1}^{⊤}\gamma_{t}$ is linear in $\gamma_{t}$ and $I\left(H_{t1}^{⊤}\gamma_{t}>0\right)$ is ℙ-Donsker. Thus it follows that ${𝒞}_{2}$ is a ℙ-Donsker class.

ii) We estimate $\xi_{1}$, $\xi_{2}$ for (4) with their maximum likelihood estimators, ${\hat{\xi }}_{1}$, ${\hat{\xi }}_{2}$, solving ${\mathbb{P}}_{n}\left[S_{t}\left(\xi_{t}\right)\right]=0$, t=1,2, by Assumption 5 and Theorem 5.9 in Vaart (1998) ${\hat{\xi }}_{t}\overset{p}{\to }{\bar{\xi }}_{t}$, t=1,2. Next, by Theorems 2, 3, under Assumptions 1, 2, ${\hat{\theta }}_{t}\overset{p}{\to }{\bar{\theta }}_{t}$, t=1,2. Thus $\mathbb{P}(\hat{\Theta }\in 𝒮(\delta ))\overset{p}{\to }1$, ∀δ. Therefore, we have $\nu_{{SSL}_{DR}}(L;\hat{\Theta })\in {𝒞}_{2}$ with high probability.

iii) We then show $\int {\left\{\nu_{{\text{SSL}}_{DR}}(L;\overset{ˆ}{\Theta })-\nu_{{\text{SSL}}_{DR}}(L;\bar{\Theta })\right\}}^{2}d{\mathbb{P}}_{L}⟶0$. Using simple algebra for a large enough constant *c* we have

$$\int \;{\left\{\nu_{{\text{SSL}}_{\text{DR}}}(L;\hat{\Theta })-\nu_{{\text{SSL}}_{\text{DR}}}(L;\bar{\Theta })\right\}}^{2}d{\mathbb{P}}_{L}\;\leq c\;\underset{Y_{2},\vec{U}}{\sup}{\left\{Y_{2}-{\bar{\mu }}_{2}^{v}(\vec{U})\right\}}^{2}\;\times \underset{{\overset{ˇ}{H}}_{1},A_{1}}{\sup}{\left\{\left(1+{\hat{\beta }}_{21}\right)\omega_{1}\left({\overset{ˇ}{H}}_{1},A_{1};{\hat{\Theta }}_{1}\right)-\left(1+{\bar{\beta }}_{21}\right)\omega_{1}\left({\overset{ˇ}{H}}_{1},A_{1};{\bar{\Theta }}_{1}\right)\right\}}^{2}\;+c\;\underset{Y_{3}}{\sup}Y_{3}^{2}\underset{{\overset{ˇ}{H}}_{2},A_{2}}{\sup}{\left\{\omega_{2}\left({\overset{ˇ}{H}}_{2},A_{2};{\hat{\Theta }}_{2}\right)-\omega_{2}\left({\overset{ˇ}{H}}_{2},A_{2};{\bar{\Theta }}_{2}\right)\right\}}^{2}\;+c\;\underset{Y_{2}}{\sup}Y_{2}^{2}\underset{{\overset{ˇ}{H}}_{2},A_{2}}{\sup}{\left\{{\hat{\beta }}_{21}\omega_{2}\left({\overset{ˇ}{H}}_{2},A_{2};{\hat{\Theta }}_{2}\right)-{\bar{\beta }}_{21}\omega_{2}\left({\overset{ˇ}{H}}_{2},A_{2};{\bar{\Theta }}_{2}\right)\right\}}^{2}\;+c\;\underset{\vec{U}}{\sup}{\bar{\mu }}_{2\omega_{3}}{\left(\overset{\to }{U}\right)}^{2}{\left({\hat{\beta }}_{21}-{\bar{\beta }}_{21}\right)}^{2}\;+c\;\underset{{\overset{ˇ}{H}}_{2},A_{2}}{\sup}{\left\{Q_{2-}^{o}\left(H_{2};{\hat{\theta }}_{2}\right)\omega_{2}\left({\overset{ˇ}{H}}_{2},A_{2};{\hat{\Theta }}_{2}\right)-Q_{2-}^{o}\left(H_{2};{\bar{\theta }}_{2}\right)\omega_{2}\left({\overset{ˇ}{H}}_{2},A_{2};{\bar{\Theta }}_{2}\right)\right\}}^{2}\;+c\;\underset{\vec{U}}{\sup}{\bar{\mu }}_{2\omega_{2}}{\left(\overset{\to }{U}\right)}^{2}\underset{H_{2}}{\sup}{\left\{Q_{2-}^{o}\left(H_{2};{\hat{\theta }}_{2}\right)-Q_{2-}^{o}\left(H_{2};{\bar{\theta }}_{2}\right)\right\}}^{2}\;\;\overset{p}{\to }0$$

where we use $(a-b{)}^{2}$, $(a+b{)}^{2}\leq 2a^{2}+2b^{2}\forall a$, b∈ℝ, boundedness of $\bar{\Theta }$ and covariates by Assumptions 1, 2 to bound all supremum quantities.

By Theorems 2 and 3 we have ${\hat{\theta }}_{2}-{\bar{\theta }}_{2}=O_{\mathbb{P}}\left(n^{-\frac{1}{2}}\right)$, ${\hat{\theta }}_{1}-{\bar{\theta }}_{1}=O_{\mathbb{P}}\left(n^{-\frac{1}{2}}\right)$, also from Lemma 17 (a) it follows that

$$\underset{H_{2}}{\sup}{\left\{Q_{2-}^{o}\left(H_{2};{\hat{\theta }}_{2}\right)-Q_{2-}^{o}\left(H_{2};{\bar{\theta }}_{2}\right)\right\}}^{2}\;\leq 2\underset{H_{20}}{\sup}{\left\|H_{20}\right\|}_{2}^{2}{\left\|{\hat{\beta }}_{22}-{\bar{\beta }}_{22}\right\|}_{2}^{2}+2\underset{H_{21}}{\sup}{\left\|H_{21}\right\|}_{2}^{2}{\left\|{\hat{\gamma }}_{22}-{\bar{\gamma }}_{22}\right\|}_{2}\;=O_{\mathbb{P}}\left(n^{-1}\right).$$

Next, we can write

$$\omega_{1}\left(H_{1},A_{1};{\hat{\Theta }}_{1}\right)=I\left\{A_{1}=d_{1}\left(H_{1};{\hat{\xi }}_{1}\right)\right\}\left\{\frac{A_{1}}{\pi_{1}\left(H_{1};{\hat{\xi }}_{1}\right)}+\frac{1-A_{1}}{1-\pi_{1}\left(H_{1};{\hat{\xi }}_{1}\right)}\right\}$$

$$\omega_{2}\left({\overset{ˇ}{H}}_{2},A_{2};{\hat{\Theta }}_{1}\right)=\omega_{1}\left(H_{1},A_{1};{\hat{\Theta }}_{1}\right)I\left\{A_{2}=d_{2}\left(H_{2};{\hat{\xi }}_{2}\right)\right\}\left\{\frac{A_{2}}{\pi_{2}\left({\overset{ˇ}{H}}_{2};{\hat{\xi }}_{2}\right)}+\frac{1-A_{2}}{2-\pi_{2}\left({\overset{ˇ}{H}}_{2};{\hat{\xi }}_{2}\right)}\right\}.$$

By Lemma 17 (b) it follows that

$$\underset{H_{1},a_{1}}{\sup}\left|I\left({\hat{d}}_{1}=A_{1}\right)-I\left({\bar{d}}_{1}=A_{1}\right)\right|=o_{\mathbb{P}}(1),$$

$$\underset{H_{2},a_{2}}{\sup}\left|I\left({\hat{d}}_{1}=A_{1}\right)I\left(A_{2}={\hat{d}}_{2}\right)-I\left({\bar{d}}_{1}=A_{1}\right)I\left({\bar{d}}_{2}=A_{2}\right)\right|=o_{\mathbb{P}}(1),$$

$$\underset{H_{1}}{\sup}\left|\frac{1}{\pi_{1}\left(H_{1};{\hat{\xi }}_{1}\right)}-\frac{1}{\pi_{1}\left(H_{1};{\bar{\xi }}_{1}\right)}\right|=O_{\mathbb{P}}\left(n^{-\frac{1}{2}}\right).$$

Using the above and Lemma 14 we get

$$\underset{{\overset{ˇ}{H}}_{1},A_{1}}{\sup}{\left\{\left(1+{\hat{\beta }}_{21}\right)\omega_{1}\left({\overset{ˇ}{H}}_{1},A_{1};{\hat{\Theta }}_{1}\right)-\left(1+{\bar{\beta }}_{21}\right)\omega_{1}\left({\overset{ˇ}{H}}_{1},A_{1};{\bar{\Theta }}_{1}\right)\right\}}^{2}=o_{\mathbb{P}}(1),$$

$$\underset{{\overset{ˇ}{H}}_{2},A_{2}}{\sup}{\left\{\left(1+{\hat{\beta }}_{21}\right)\omega_{2}\left({\overset{ˇ}{H}}_{2},A_{2};{\hat{\Theta }}_{2}\right)-\left(1+{\bar{\beta }}_{21}\right)\omega_{2}\left({\overset{ˇ}{H}}_{2},A_{2};{\bar{\Theta }}_{2}\right)\right\}}^{2}=o_{\mathbb{P}}(1),$$

$$\underset{{\overset{ˇ}{H}}_{2},A_{2}}{\sup}{\left\{Q_{2-}^{o}\left(H_{2};{\hat{\theta }}_{2}\right)\omega_{2}\left({\overset{ˇ}{H}}_{2},A_{2};{\hat{\Theta }}_{2}\right)-Q_{2-}^{o}\left(H_{2};{\bar{\theta }}_{2}\right)\omega_{2}\left({\overset{ˇ}{H}}_{2},A_{2};{\bar{\Theta }}_{2}\right)\right\}}^{2}=o_{\mathbb{P}}(1),$$

$$\underset{{\overset{ˇ}{H}}_{2},A_{2}}{\sup}{\left\{{\hat{\beta }}_{21}\omega_{2}\left({\overset{ˇ}{H}}_{2},A_{2};{\hat{\Theta }}_{2}\right)-{\bar{\beta }}_{21}\omega_{2}\left({\overset{ˇ}{H}}_{2},A_{2};{\bar{\Theta }}_{2}\right)\right\}}^{2}=o_{\mathbb{P}}(1).$$

which gives us $\int {\left\{\nu_{{SSL}_{DR}}(L;\hat{\Theta })-\nu_{{SSL}_{DR}}(L;\bar{\Theta })\right\}}^{2}d{\mathbb{P}}_{L}\overset{p}{\to }0$.

Therefore we have i) $\mathbb{P}(\hat{\Theta }\in 𝒮(\delta ))\to 1$, ∀δ, ii) ${𝒞}_{2}$ is a ℙ-Donsker class, and iii) $\int {\left(\nu_{{\text{SSL}}_{\text{DR}}}(L;\hat{\Theta })-\nu_{{\text{SSL}}_{\text{DR}}}(L;\bar{\Theta })\right)}^{2}d{\mathbb{P}}_{L}\to 0$. By Theorem 2.1 in Van Der Vaart and Wellner (2007)

$$\frac{1}{\sqrt{n}}\sum_{i=1}^{n}\left\{\left(\nu_{{\text{SSL}}_{\text{DR}}}\left(L_{i};\hat{\Theta }\right)-{𝔼}_{𝕊}\left[\nu_{{\text{SSL}}_{\text{DR}}}(L;\hat{\Theta })\right]\right)-\left(\nu_{{\text{SSL}}_{\text{DR}}}\left(L_{i};\bar{\Theta }\right)-{𝔼}_{𝕊}\left[\nu_{{\text{SSL}}_{\text{DR}}}(L;\bar{\Theta })\right]\right)\right\}=o_{\mathbb{P}}(1).$$

by 1), 2) and noting that $\nu_{{SSL}_{DR}}(L_{i};\bar{\Theta })$ has mean zero we obtain the result in (17).

We next re-write $\sqrt{n}{\mathbb{P}}_{N}\left[{ℰ}_{\bar{\Theta }}\right]$ by expressing the estimated imputation functions in ${ℰ}_{\bar{\Theta }}$ in terms of the labeled sample ℒ. Letting

$${\hat{C}}_{n,N}^{\left(1\right)}=\frac{\left(1+{\bar{\beta }}_{21}\right){\mathbb{P}}_{N}\left\{\omega_{1}\left({\overset{ˇ}{H}}_{1},A_{1};\bar{\Theta }\right)\right\}}{\left(1+{\hat{\beta }}_{21}\right){\mathbb{P}}_{n}\left\{\omega_{1}\left({\overset{ˇ}{H}}_{1},A_{1};\hat{\Theta }\right)\right\}},{\hat{C}}_{n,N}^{\left(2\right)}=\frac{{\mathbb{P}}_{N}\left\{Q_{2-}^{o}\left(H_{2};{\bar{\theta }}_{2}\right)\right\}}{{\mathbb{P}}_{n}\left\{Q_{2-}^{o}\left(H_{2};{\hat{\theta }}_{2}\right)\right\}},$$

we can write:

$$\frac{1}{N}\sum_{j=1}^{N}\omega_{1}\left({\overset{ˇ}{H}}_{1j},A_{1j},\bar{\Theta }\right)\left(1+{\bar{\beta }}_{21}\right)\left\{{\hat{\mu }}_{2}^{v}\left({\vec{U}}_{j}\right)-{\bar{\mu }}_{2}^{v}\left({\vec{U}}_{j}\right)\right\}\;=\frac{1}{N}\sum_{j=1}^{N}\omega_{1}\left({\overset{ˇ}{H}}_{1j},A_{1j},\bar{\Theta }\right)\left(1+{\bar{\beta }}_{21}\right)\left\{\frac{1}{K}\sum_{k=1}^{K}{\hat{m}}_{2}^{\left(-k\right)}\left({\vec{U}}_{j}\right)+{\hat{\eta }}_{2}^{v}-m_{2}\left({\vec{U}}_{j}\right)-\eta_{2}^{v}\right\}\;=\left(1+{\bar{\beta }}_{21}\right)\frac{1}{KN}\sum_{j=1}^{N}\sum_{k=1}^{K}\omega_{1}\left({\overset{ˇ}{H}}_{1j},A_{1j},\bar{\Theta }\right){\hat{\text{Δ}}}_{2}^{\left(-k\right)}\left({\vec{U}}_{j}\right)+\left({\hat{\eta }}_{2}^{v}-\eta_{2}^{v}\right)\frac{1}{N}\sum_{j=1}^{N}\omega_{1}\left({\overset{ˇ}{H}}_{1j},A_{1j},\bar{\Theta }\right)\left(1+{\bar{\beta }}_{21}\right),$$

where the first step follows from constrains shown in (6) and we simply regroup terms in the second step.

Next note that we can use Lemma 15 to replace

$${\mathbb{P}}_{N}\left[\left(1+{\bar{\beta }}_{21}\right)\omega_{1}\left({\overset{ˇ}{H}}_{1},A_{1},\bar{\Theta }\right){\hat{\text{Δ}}}_{2}^{\left(-k\right)}\left({\vec{U}}_{j}\right)\right]$$

by

$${𝔼}_{ℒ}\left[\left(1+{\bar{\beta }}_{21}\right)\omega_{1}\left({\overset{ˇ}{H}}_{1},A_{1},\bar{\Theta }\right){\hat{\text{Δ}}}_{2}^{\left(-k\right)}\left({\vec{U}}_{j}\right)\right]+O_{\mathbb{P}}\left(N^{-\frac{1}{2}}\right),$$

using ${𝔼}_{ℒ}[\cdot ]$ to denote expectation with respect to ℒ. Additionally, using (6) and the definition of ${\overset{‾}{\mu }}_{2}^{v}(\vec{U})$ for the second term we get:

$$\frac{1}{N}\sum_{j=1}^{N}\omega_{1}\left({\overset{ˇ}{H}}_{1j},A_{1j};\bar{\Theta }\right)\left(1+{\bar{\beta }}_{21}\right)\;\left\{{\hat{\mu }}_{2}^{v}\left({\vec{U}}_{j}\right)-{\bar{\mu }}_{2}^{v}\left({\vec{U}}_{j}\right)\right\}\;={𝔼}_{ℒ}\left[\frac{1}{K}\sum_{k=1}^{K}\left(1+{\bar{\beta }}_{21}\right)\omega_{1}\left({\overset{ˇ}{H}}_{1},A_{1},\bar{\Theta }\right){\hat{\text{Δ}}}_{2}^{\left(-k\right)}(\vec{U})\right]+O_{\mathbb{P}}\left(N^{-\frac{1}{2}}\right)\;-{\hat{C}}_{n,N}^{\left(1\right)}\frac{1}{n}\sum_{k=1}^{K}\sum_{i\in {ℐ}_{k}}\left(1+{\hat{\beta }}_{21}\right)\omega_{1}\left({\overset{ˇ}{H}}_{1i},A_{1i};\hat{\Theta }\right){\hat{\text{Δ}}}_{2}^{\left(-k\right)}\left({\vec{U}}_{i}\right)\;+{\hat{C}}_{n,N}^{\left(1\right)}\left(1+{\hat{\beta }}_{21}\right)\frac{1}{n}\sum_{i=1}^{n}\omega_{1}\left({\overset{ˇ}{H}}_{1i},A_{1i};\hat{\Theta }\right)\;\left\{Y_{2i}-{\bar{\mu }}_{2}^{v}\left({\vec{U}}_{i}\right)\right\}\;=\left\{1+O_{\mathbb{P}}\left(n^{-\frac{1}{2}}\right)\right\}\;\left(1+{\hat{\beta }}_{21}\right)\frac{1}{n}\sum_{i=1}^{n}\omega_{1}\left({\overset{ˇ}{H}}_{1i},A_{1i};\hat{\Theta }\right)\;\left\{Y_{2i}-{\bar{\mu }}_{2}^{v}\left({\vec{U}}_{i}\right)\right\}+O_{\mathbb{P}}\left(n^{-\frac{1}{2}}c_{n_{K}^{-}}\right),$$

where the last step follows from Assumption 6 and Lemma 16 choosing *f* to be the constant function 1, setting ${\overset{ˆ}{\Delta }}_{k}(\vec{U})={\overset{ˆ}{\Delta }}_{2}^{\left(-k\right)}(\vec{U})$, $\overset{ˆ}{l}({\overset{ˇ}{H}}_{1})=A_{1}I\left(H_{11}^{⊤}{\hat{\gamma }}_{1}>0\right)$, and $\overset{ˆ}{\pi }({\overset{ˇ}{H}}_{1})=\pi_{1}({\overset{ˇ}{H}}_{1};{\hat{\xi }}_{1})$ and with ${\overset{ˆ}{C}}_{n,N}={\overset{ˆ}{C}}_{n,N}^{\left(1\right)}$-which satisfies ${\overset{ˆ}{C}}_{n,N}^{\left(1\right)}=1+O_{\mathbb{P}}(n^{-\frac{1}{2}})$ by Lemma 17 (c).

Using similar arguments we have

$$\frac{1}{N}\sum_{j=1}^{N}Q_{2-}^{o}\left(H_{2j};{\bar{\theta }}_{2}\right)\left\{{\hat{\mu }}_{\omega_{2}}\left({\vec{U}}_{j}\right)-{\bar{\mu }}_{\omega_{2}}\left({\vec{U}}_{j}\right)\right\}\;=\frac{1}{N}\sum_{j=1}^{N}Q_{2-}^{o}\left(H_{2j};{\bar{\theta }}_{2}\right)\left\{\frac{1}{K}\sum_{k=1}^{K}{\hat{m}}_{\omega_{2}}^{\left(-k\right)}\left({\vec{U}}_{j}\right)+{\hat{\eta }}_{\omega_{2}}^{v}-m_{\omega_{2}}\left({\vec{U}}_{j}\right)-\eta_{\omega_{2}}^{v}\right\}\;=\frac{1}{KN}\sum_{j=1}^{N}\sum_{k=1}^{K}Q_{2-}^{o}\left(H_{2j};{\bar{\theta }}_{2}\right){\hat{\text{Δ}}}_{\omega_{2}k}\left({\vec{U}}_{j}\right)+\left({\hat{\eta }}_{\omega_{2}}^{v}-\eta_{\omega_{2}}^{v}\right)\frac{1}{N}\sum_{j=1}^{N}Q_{2-}^{o}\left(H_{2j};{\bar{\theta }}_{2}\right)\;={𝔼}_{ℒ}\left[\frac{1}{K}\sum_{k=1}^{K}Q_{2-}^{o}\left(H_{2j};{\bar{\theta }}_{2}\right){\hat{\text{Δ}}}_{\omega_{2}k}(\vec{U})\right]-{\hat{C}}_{n,N}^{\left(2\right)}\frac{1}{n}\sum_{k=1}^{K}\sum_{i\in {ℐ}_{k}}Q_{2-}^{o}\left(H_{2i};{\bar{\theta }}_{2}\right){\hat{\text{Δ}}}_{\omega_{2}k}\left({\vec{U}}_{i}\right)\;+{\hat{C}}_{n,N}^{\left(2\right)}\frac{1}{n}\sum_{i=1}^{n}Q_{2-}^{o}\left(H_{2i};{\hat{\theta }}_{2}\right)\left\{\omega_{2}\left({\overset{ˇ}{H}}_{2i},A_{2i};\hat{\Theta }\right)-{\bar{\mu }}_{\omega_{2}}\left({\vec{U}}_{i}\right)\right\}+O_{\mathbb{P}}\left(N^{-\frac{1}{2}}\right)\;=\left\{1+O_{\mathbb{P}}\left(n^{-\frac{1}{2}}\right)\right\}\frac{1}{n}\sum_{i=1}^{n}Q_{2-}^{o}\left(H_{2i};{\hat{\theta }}_{2}\right)\left\{\omega_{2}\left({\overset{ˇ}{H}}_{2i},A_{2i};\hat{\Theta }\right)-{\bar{\mu }}_{\omega_{2}}\left({\vec{U}}_{i}\right)\right\}+O_{\mathbb{P}}\left(n^{-\frac{1}{2}}c_{n_{K}^{-}}\right),$$

and for t=2,3

$$\frac{1}{N}\sum_{j=1}^{N}\left\{{\hat{\mu }}_{t\omega_{2}}^{v}\left({\vec{U}}_{j}\right)-{\bar{\mu }}_{t\omega_{2}}^{v}\left({\vec{U}}_{j}\right)\right\}=\frac{1}{KN}\sum_{j=1}^{N}\left\{\sum_{k=1}^{K}{\hat{m}}_{t\omega_{2}}^{\left(-k\right)}\left({\vec{U}}_{j}\right)+{\hat{\eta }}_{t\omega_{2}}^{v}-m_{t\omega_{2}}\left({\vec{U}}_{j}\right)-\eta_{t\omega_{2}}^{v}\right\}\;\;=\frac{1}{KN}\sum_{j=1}^{N}\sum_{k=1}^{K}{\hat{\text{Δ}}}_{3\omega_{2}k}\left({\vec{U}}_{j}\right)+\left({\hat{\eta }}_{t\omega_{2}}^{v}-\eta_{t\omega_{2}}^{v}\right)\;\;={𝔼}_{ℒ}\left[\frac{1}{K}\sum_{k=1}^{K}{\hat{\text{Δ}}}_{3\omega_{2}k}(\vec{U})\right]-\frac{1}{n}\sum_{k=1}^{K}\sum_{i\in {ℐ}_{k}}{\hat{\text{Δ}}}_{3\omega_{2}k}\left({\vec{U}}_{i}\right)\;\;+\frac{1}{n}\sum_{i=1}^{n}\omega_{2}\left({\overset{ˇ}{H}}_{2i},A_{2i};\hat{\Theta }\right)Y_{ti}-{\bar{\mu }}_{t\omega_{2}}^{v}\left({\vec{U}}_{i}\right)+O_{\mathbb{P}}\left(N^{-\frac{1}{2}}\right)\;\;=\frac{1}{n}\sum_{i=1}^{n}\omega_{2}\left({\overset{ˇ}{H}}_{2i},A_{2i};\hat{\Theta }\right)Y_{ti}-{\bar{\mu }}_{t\omega_{2}}^{v}\left({\vec{U}}_{i}\right)+O_{\mathbb{P}}\left(n^{-\frac{1}{2}}c_{n_{K}^{-}}\right),$$

finally by Theorem 2, ${\overset{ˆ}{\beta }}_{21}-{\overset{‾}{\beta }}_{21}=O_{\mathbb{P}}\left(n^{-\frac{1}{2}}\right)$.

Therefore, recalling the definition of $\nu_{{\mathrm{SSL}}_{\mathrm{DR}}}$ from Theorem 7, using the derivations above, we can write

(18)
$$\frac{\sqrt{n}}{N}\sum_{j=1}^{N}{ℰ}_{\bar{\Theta }}\left({\vec{U}}_{j}\right)=\frac{1}{\sqrt{n}}\sum_{i=1}^{n}\nu_{{\text{SSL}}_{\text{DR}}}\left({\vec{U}}_{i};\hat{\Theta }\right)\;\;+O_{\mathbb{P}}\left(n^{-\frac{1}{2}}\right)\frac{1}{\sqrt{n}}\sum_{i=1}^{n}\omega_{1}\left({\overset{ˇ}{H}}_{1},A_{1};{\hat{\Theta }}_{1}\right)\left(1+{\hat{\beta }}_{21}\right)\left\{Y_{2}-{\bar{\mu }}_{2}^{v}(\vec{U})\right\}\;\;-O_{\mathbb{P}}\left(n^{-\frac{1}{2}}\right)\frac{1}{\sqrt{n}}\sum_{i=1}^{n}{\hat{\beta }}_{21}\left\{\omega_{2}\left({\overset{ˇ}{H}}_{2},A_{2},{\hat{\Theta }}_{2}\right)Y_{2}-{\bar{\mu }}_{2\omega_{2}}(\vec{U})\right\}\;\;-O_{\mathbb{P}}\left(n^{-\frac{1}{2}}\right)\frac{1}{\sqrt{n}}\sum_{i=1}^{n}Q_{2-}^{o}\left(H_{2};{\hat{\theta }}_{2}\right)\left\{\omega_{2}\left({\overset{ˇ}{H}}_{2},A_{2},{\bar{\Theta }}_{2}\right)-{\bar{\mu }}_{\omega_{2}}(\vec{U})\right\}\;\;+O_{\mathbb{P}}\left(c_{n_{K}^{-}}\right).$$

Using result (17) we know $\frac{1}{\sqrt{n}}\sum_{i=1}^{n}\nu_{{SSL}_{DR}}({\vec{U}}_{i};\hat{\Theta })=O_{\mathbb{P}}(1)$, therefore the second, third and fourth terms in (18) are $o_{\mathbb{P}}(1)$. Using (17) again for the first term in (18) we get our required result:

$$\sqrt{n}{\mathbb{P}}_{N}\left[{ℰ}_{\bar{\Theta }}\right]=\frac{1}{\sqrt{n}}\sum_{i=1}^{n}\nu_{{\text{SSL}}_{\text{DR}}}\left(L_{i};\bar{\Theta }\right)\;\;+\frac{1}{\sqrt{n}}\sum_{i=1}^{n}{\left(\frac{\partial }{\partial \theta }𝔼\left[\nu_{{\text{SSL}}_{\text{DR}}}\left(L_{i};\bar{\Theta }\right)\right]\right)}^{⊤}\psi^{\theta }\left(L_{i}\right)\;\;+\frac{1}{\sqrt{n}}\sum_{i=1}^{n}{\left(\frac{\partial }{\partial \xi }𝔼\left[\nu_{{\text{SSL}}_{\text{DR}}}\left(L_{i};\bar{\Theta }\right)\right]\right)}^{⊤}\psi^{\xi }\left(L_{i}\right)+o_{\mathbb{P}}(1).$$

■

Proof [Proof of Proposition 8]

Recall the definition of ${𝒱}_{{\text{SUP}}_{\text{DR}}}(L;\bar{\Theta })$ in (5), using (13) we have $𝔼\left[{𝒱}_{{\text{SSL}}_{\text{DR}}}(\vec{U};\bar{\Theta },\overset{‾}{\mu })\right]=𝔼\left[{𝒱}_{{\text{SUP}}_{\text{DR}}}(L;\bar{\Theta })\right]$, therefore

$$\text{Bias}\left\{\bar{V},{𝒱}_{{\text{SUP}}_{\text{DR}}}(L;\bar{\Theta })\right\}=\text{Bias}\left\{\bar{V},{𝒱}_{{\text{SSL}}_{\text{DR}}}(\vec{U};\bar{\Theta },\bar{\mu })\right\}.$$

Therefore, by Lemma 18 we have

$$\text{Bias}\left\{\bar{V},{𝒱}_{{\text{SSL}}_{\text{DR}}}(\vec{U};\bar{\Theta },\bar{\mu })\right\}\;\leq \sqrt{\underset{{\overset{˘}{H}}_{1}}{\sup}\left|{\left\{1-\pi_{1}\left({\overset{ˇ}{H}}_{1};{\bar{\xi }}_{1}\right)\right\}}^{-1}\right|}\sqrt{{\left\|\pi_{1}\left({\overset{ˇ}{H}}_{1};{\bar{\xi }}_{1}\right)-\pi_{1}\left({\overset{ˇ}{H}}_{1}\right)\right\|}_{L_{2}(\mathbb{P})}}\sqrt{{\left\|Q_{1}^{o}\left({\overset{ˇ}{H}}_{1};{\bar{\theta }}_{1}\right)-Q_{1}^{o}\left({\overset{ˇ}{H}}_{1}\right)\right\|}_{L_{2}(\mathbb{P})}}\;+\sqrt{\underset{{\overset{ˇ}{H}}_{2}}{\sup}\left|\left\{\frac{A_{1}}{\pi_{1}\left({\overset{ˇ}{H}}_{1};{\bar{\xi }}_{1}\right)}+\frac{1-A_{1}}{1-\pi_{1}\left({\overset{ˇ}{H}}_{1};{\bar{\xi }}_{1}\right)}\right\}\;{\left\{1-\pi_{1}\left({\overset{ˇ}{H}}_{1};{\bar{\xi }}_{1}\right)\right\}}^{-1}{\left\{1-\pi_{2}\left({\overset{ˇ}{H}}_{2};{\bar{\xi }}_{2}\right)\right\}}^{-1}\right|}\;\times \sqrt{{\left\|\pi_{2}\left({\overset{ˇ}{H}}_{2};{\bar{\xi }}_{2}\right)-\pi_{2}\left({\overset{ˇ}{H}}_{2}\right)\right\|}_{L_{2}(\mathbb{P})}}\sqrt{{\left\|Q_{2}^{o}\left({\overset{ˇ}{H}}_{2};{\bar{\theta }}_{2}\right)-Q_{2}^{o}\left({\overset{ˇ}{H}}_{2}\right)\right\|}_{L_{2}(\mathbb{P})}}.$$

Next using Theorem 7

(19)
$$\sqrt{n}\left\{{\hat{V}}_{{\text{SSL}}_{\text{DR}}}-\bar{V}\right\}+\sqrt{n}\text{Bias}\left\{\bar{V},{𝒱}_{{\text{SSL}}_{\text{DR}}}(\vec{U};\bar{\Theta },\bar{\mu })\right\}\overset{d}{\to }N\left(0,\sigma_{{\text{SSL}}_{\text{DR}}}^{2}\right),$$

if either (1) or (4) are correct then $Bias\{\overset{‾}{V},{𝒱}_{{SSL}_{DR}}(\vec{U};\bar{\Theta },\overset{‾}{\mu })\}=o_{\mathbb{P}}(1)$, multiplying (19) by $n^{-\frac{1}{2}}$ we have

$${\hat{V}}_{{\text{SSL}}_{\text{DR}}}-\bar{V}\overset{\mathbb{P}}{\to }0,$$

which is the required result for Proposition 8 (a).

Next, if $\sqrt{{\left\|\pi_{t}({\overset{ˇ}{H}}_{t};{\hat{\xi }}_{t})-\pi_{t}({\overset{ˇ}{H}}_{t})\right\|}_{L_{2}(\mathbb{P})}}\sqrt{{\left\|Q_{t}^{o}({\overset{ˇ}{H}}_{t};{\hat{\theta }}_{t})-Q_{t}^{o}({\overset{ˇ}{H}}_{t})\right\|}_{L_{2}(\mathbb{P})}}=O_{\mathbb{P}}\left(n^{-1}\right)$ for t=1,2 then $\text{Bias}\{\overset{‾}{V},{𝒱}_{{SSL}_{DR}}(\vec{U};\bar{\Theta },\overset{‾}{\mu })\}=O_{\mathbb{P}}\left(n^{-1}\right)$ and from (19) we get

$$\sqrt{n}\left\{{\hat{V}}_{{\text{SSL}}_{\text{DR}}}-\bar{V}\right\}\overset{d}{\to }N\left(0,\sigma_{{\text{SSL}}_{\text{DR}}}^{2}\right),$$

which is the required result for Proposition 8 (b). ■

Before proving Proposition 9, we introduce a useful definition and state the necessary assumption to prove the result. Let $\psi_{\text{SUP}}^{\xi }(L;\bar{\xi })$ and $\psi_{\text{SSL}}^{\xi }(L;\bar{\xi })$ be the supervised and SSL influence functions respectively for ξ, then we define

$${ℰ}^{v}(\vec{U})={𝒱}_{{\text{SSL}}_{\text{DR}}}(\vec{U};\bar{\Theta },\bar{\mu })-{𝔼}_{𝕊}\left[{𝒱}_{{\text{SUP}}_{\text{DR}}}(L;\bar{\Theta })\right]+{{ℰ}^{\theta }{\left(\vec{U}\right)}^{⊤}\frac{\partial }{\partial \theta }\int {𝒱}_{{\text{SUP}}_{\text{DR}}}(L;\bar{\Theta })d{\mathbb{P}}_{L}|}_{\Theta =\bar{\Theta }}+{{ℰ}^{\xi }{\left(\vec{U}\right)}^{⊤}\frac{\partial }{\partial \xi }\int {𝒱}_{{\text{SUP}}_{\text{DR}}}(L;\bar{\Theta })d{\mathbb{P}}_{L}|}_{\Theta =\bar{\Theta }},$$

$${ℰ}^{\xi }(\vec{U})=\psi_{\text{SUP}}^{\xi }(L;\bar{\xi })-\psi_{\text{SSL}}^{\xi }(L;\bar{\xi }).$$

We need to ensure that the imputation models ${\overset{‾}{\mu }}_{2}^{v}(\vec{U})$, ${\overset{‾}{\mu }}_{\omega_{2}}^{v}(\vec{U})$, ${\overset{‾}{\mu }}_{t\omega_{2}}^{v}(\vec{U})$, t=2,3 used in the SSL value function estimator $V_{{\text{SSL}}_{\text{DR}}}$ are unbiased when multiplied by several functions. For example, we need additional constraints of the type:

$$𝔼\left[\omega_{1}\left({\overset{ˇ}{H}}_{1},A_{1};{\bar{\Theta }}_{1}\right)Q_{2-}^{o}\left(H_{2};{\bar{\theta }}_{1}\right)\left\{Y_{2}-{\bar{\mu }}_{2}(\vec{U})\right\}\right]=0,$$

$$𝔼\left[\omega_{1}{\left({\overset{ˇ}{H}}_{1},A_{1};{\bar{\Theta }}_{1}\right)}^{2}\left\{Y_{2}-{\bar{\mu }}_{2}(\vec{U})\right\}\right]=0,$$

$$𝔼\left[\omega_{1}{\left({\overset{ˇ}{H}}_{1},A_{1};{\bar{\Theta }}_{1}\right)}^{2}\left\{Y_{2}-{\bar{\mu }}_{2}(\vec{U})\right\}\right]=0,$$

so the imputation models are unbiased in expectation when multiplied by every term and cross-product of terms in $\psi_{{\text{SUP}}_{\text{DR}}}^{v}(L;\bar{\Theta }),{ℰ}^{v}(\vec{U})$. These constraints can be summarized in the following Assumption.

**Assumption 8** *Imputation models* ${\overset{‾}{\mu }}_{2}^{v}(\vec{U})$, ${\overset{‾}{\mu }}_{\omega_{2}}^{v}(\vec{U})$, ${\overset{‾}{\mu }}_{t\omega_{2}}^{v}(\vec{U})$, t=2,3 *satisfy*

$$𝔼\left[\left\{{ℰ}^{v}(\vec{U})-\psi_{{\text{SUP}}_{\text{DR}}}^{v}(L;\bar{\Theta })\right\}{ℰ}^{v}(\vec{U})\right]=0.$$

**Proof** [Proof of Proposition 9] From Theorem 19 in Appendix F.1 we have that the influence function for the fully-supervised value function estimator (5) is:

$$\psi_{{\text{SUP}}_{\text{DR}}}^{v}(L;\bar{\Theta })={𝒱}_{{\text{SUP}}_{\text{DR}}}(L;\bar{\Theta })-{𝔼}_{𝕊}\left[{𝒱}_{{\text{SUP}}_{\text{DR}}}(L;\bar{\Theta })\right]+{\psi_{\text{SUP}}^{\theta }{\left(L\right)}^{⊤}\frac{\partial }{\partial \theta }\int {𝒱}_{{\text{SUP}}_{\text{DR}}}(L;\bar{\Theta })d{\mathbb{P}}_{L}|}_{\Theta =\bar{\Theta }}+{\psi_{\text{SUP}}^{\xi }{\left(L\right)}^{⊤}\frac{\partial }{\partial \xi }\int {𝒱}_{{\text{SUP}}_{\text{DR}}}(L;\bar{\Theta })d{\mathbb{P}}_{L}|}_{\Theta =\bar{\Theta }}.$$

Next, as we estimate ξ with a semi-supervised approach such that $\psi_{\text{SSL}}^{\xi }(L;\bar{\xi })=\psi_{\text{SUP}}^{\xi }(L;\bar{\xi })-{ℰ}^{\xi }(\vec{U})$, simple algebra can be used to show that

$$\psi_{{\text{SSL}}_{\text{DR}}}^{v}(L;\bar{\Theta })=\psi_{{\text{SUP}}_{\text{DR}}}^{v}(L;\bar{\Theta })-{ℰ}^{v}(\vec{U}).$$

Using the above we can write

$$\sigma_{{\text{SSL}}_{\text{DR}}}^{2}=𝔼\left[\psi_{{\text{SSL}}_{\text{DR}}}^{v}{\left(L;\bar{\Theta }\right)}^{2}\right]=𝔼\left[{\left\{\psi_{{\text{SUP}}_{\text{DR}}}^{v}(L;\bar{\Theta })-{ℰ}^{v}(\vec{U})\right\}}^{2}\right]\;\;=𝔼\left[\psi_{{\text{SUP}}_{\text{DR}}}^{v}{\left(L;\bar{\Theta }\right)}^{2}\right]+𝔼\left[{ℰ}^{v}{\left(\vec{U}\right)}^{2}\right]\;\;-2𝔼\left[\psi_{{\text{SUP}}_{\text{DR}}}^{v}(L;\bar{\Theta }){ℰ}^{v}(\vec{U})\right].$$

By Assumption 8, we have $𝔼\left[\left\{{ℰ}^{v}(\vec{U})-\psi_{{\mathrm{SUP}}_{\mathrm{DR}}}^{v}(L;\bar{\Theta })\}{ℰ}^{v}(\vec{U}\right)\right]=0$, hence

$$\sigma_{{\text{SSL}}_{\text{DR}}}^{2}=\sigma_{{\text{SUP}}_{\text{DR}}}^{2}-\text{Var}\left[{ℰ}^{v}(\vec{U})\right].$$

■

#### D.2.1 Variance Estimation for ${\hat{V}}_{{SUP}_{DR}}$

As discussed in Remark 10, to estimate standard errors for $V_{{SSL}_{DR}}(\vec{U};\bar{\Theta })$, we will approximate the derivatives of the expectation terms $\frac{\partial }{\partial \Theta }\int {𝒱}_{{\text{SUP}}_{\text{DR}}}(L;\bar{\Theta })d{\mathbb{P}}_{L}$ using kernel smoothing to replace the indicator functions. In particular, let ${𝕂}_{h}(x)=\frac{1}{h}\sigma (x/h)$, with σ defined as in (4), we approximate $d_{t}\left(H_{t},\theta_{t}\right)=I\left(H_{t1}^{⊤}\gamma_{t}>0\right)$ with ${𝕂}_{h}\left(H_{t1}^{⊤}\gamma_{t}\right)t=1,2$, and define the smoothed propensity score weights as

$${\tilde{\omega }}_{1}\left({\overset{ˇ}{H}}_{1},A_{1},\Theta \right)\equiv \frac{A_{1}{𝕂}_{h}\left(H_{11}^{⊤}\gamma_{1}\right)}{\pi_{1}\left({\overset{ˇ}{H}}_{1};\xi_{1}\right)}+\frac{\left\{1-A_{1}\right\}\left\{1-{𝕂}_{h}\left(H_{11}^{⊤}\gamma_{1}\right)\right\}}{1-\pi_{1}\left({\overset{ˇ}{H}}_{1};\xi_{1}\right)},\;\text{and}$$

$${\tilde{\omega }}_{2}\left({\overset{ˇ}{H}}_{2},A_{2},\Theta \right)\equiv {\tilde{\omega }}_{1}\left({\overset{ˇ}{H}}_{1},A_{1},\Theta \right)\left[\frac{A_{2}{𝕂}_{h}\left(H_{21}^{⊤}\gamma_{2}\right)}{\pi_{2}\left({\overset{ˇ}{H}}_{2};\xi_{2}\right)}+\frac{\left\{1-A_{2}\right\}\left\{1-{𝕂}_{h}\left(H_{21}^{⊤}\gamma_{2}\right)\right\}}{1-\pi_{2}\left({\overset{ˇ}{H}}_{2};\xi_{2}\right)}\right].$$

For simplicity we’ll set h=1, the derivatives are as follows:

$$\frac{\partial }{\partial \theta }{𝒱}_{{\text{SUP}}_{\text{DR}}}(L;\Theta )=\frac{\partial }{\partial \theta }Q_{1}^{o}\left(H_{1};\theta_{1}\right)+\left\{\frac{\partial }{\partial \theta }{\tilde{\omega }}_{1}\left({\overset{ˇ}{H}}_{1},A_{1},\Theta \right)\right\}\left[Y_{2}-\left\{Q_{1}^{o}\left(H_{1},\theta_{1}\right)-Q_{2}^{o}\left({\overset{ˇ}{H}}_{2};\theta_{2}\right)\right\}\right]\;\;+{\tilde{\omega }}_{1}\left({\overset{ˇ}{H}}_{1},A_{1},\Theta \right)\left[-\frac{\partial }{\partial \theta }Q_{1}^{o}\left(H_{1},\theta_{1}\right)+\frac{\partial }{\partial \theta }Q_{2}^{o}\left({\overset{ˇ}{H}}_{2};\theta_{2}\right)\right]\;\;+\left\{\frac{\partial }{\partial \theta }{\tilde{\omega }}_{2}\left({\overset{ˇ}{H}}_{2},A_{2},\Theta \right)\right\}\left[Y_{3}-Q_{2}^{o}\left({\overset{ˇ}{H}}_{2};\theta_{2}\right)\right]\;\;-{\tilde{\omega }}_{2}\left({\overset{ˇ}{H}}_{2},A_{2},\Theta \right)\frac{\partial }{\partial \theta }Q_{2}^{o}\left({\overset{ˇ}{H}}_{2};\theta_{2}\right),$$

where

$$\frac{\partial }{\partial \theta }Q_{1}^{o}\left(H_{1};\theta_{1}\right)={\left[H_{10}^{⊤},H_{11}^{⊤}I\left(H_{11}^{⊤}\gamma_{1}>0\right),0^{⊤}\right]}^{⊤},\;\frac{\partial }{\partial \theta }Q_{2}^{o}\left({\overset{ˇ}{H}}_{2};\theta_{2}\right)={\left[0^{⊤},H_{20}^{⊤},H_{21}^{⊤}I\left(H_{21}^{⊤}\gamma_{2}>0\right)\right]}^{⊤},\;\frac{\partial }{\partial \theta }{\tilde{\omega }}_{1}\left({\overset{ˇ}{H}}_{1},A_{1},\Theta \right)=\left\{\frac{A_{1}}{\pi_{1}\left({\overset{ˇ}{H}}_{1};\xi_{1}\right)}-\frac{1-A_{1}}{1-\pi_{1}\left({\overset{ˇ}{H}}_{1};\xi_{1}\right)}\right\}\;\times {\left[0^{⊤},H_{11}^{⊤}{𝕂}_{h}\left(H_{11}^{⊤}\gamma_{1}\right)\left\{1-{𝕂}_{h}\left(H_{11}^{⊤}\gamma_{1}\right)\right\},0^{⊤}\right]}^{⊤}\;\frac{\partial }{\partial \theta }{\tilde{\omega }}_{2}\left({\overset{ˇ}{H}}_{2},A_{2},\Theta \right)=\frac{\partial }{\partial \theta }{\tilde{\omega }}_{1}\left({\overset{ˇ}{H}}_{1},A_{1},\Theta \right)\left\{\frac{A_{2}d_{2}\left(H_{2};\theta_{2}\right)}{\pi_{2}\left({\overset{ˇ}{H}}_{2};\xi_{2}\right)}+\frac{\left\{1-A_{2}\right\}\left\{1-d_{2}\left(H_{2};\theta_{2}\right)\right\}}{1-\pi_{2}\left({\overset{ˇ}{H}}_{2};\xi_{2}\right)}\right\}\;+{\tilde{\omega }}_{1}\left({\overset{ˇ}{H}}_{1},A_{1},\Theta \right){\left[0^{⊤},H_{21}^{⊤}{𝕂}_{h}\left(H_{21}^{⊤}\gamma_{2}\right)\left(1-{𝕂}_{h}\left(H_{21}^{⊤}\gamma_{2}\right)\right)\left\{\frac{A_{2}}{\pi_{2}\left({\overset{ˇ}{H}}_{2};\xi_{2}\right)}-\frac{1-A_{2}}{1-\pi_{2}\left({\overset{ˇ}{H}}_{2};\xi_{2}\right)}\right\}\right]}^{⊤}.$$

Next we have

$$\frac{\partial }{\partial \xi }{𝒱}_{{\text{SUP}}_{\text{DR}}}(L;\Theta )=\left\{\frac{\partial }{\partial \xi }{\tilde{\omega }}_{1}\left({\overset{ˇ}{H}}_{1},A_{1},\Theta \right)\right\}\left[Y_{2}-\left\{Q_{1}^{o}\left(H_{1},\theta_{1}\right)-Q_{2}^{o}\left({\overset{ˇ}{H}}_{2};\theta_{2}\right)\right\}\right]\;+\left\{\frac{\partial }{\partial \xi }{\tilde{\omega }}_{2}\left({\overset{ˇ}{H}}_{2},A_{2},\Theta \right)\right\}\;\left[Y_{3}-Q_{2}^{o}\left({\overset{ˇ}{H}}_{2};\theta_{2}\right)\right],$$

where

$$\frac{\partial }{\partial \xi }{\tilde{\omega }}_{1}\left({\overset{ˇ}{H}}_{1},A_{1},\Theta \right)={\left[\varpi_{1}{\left({\overset{ˇ}{H}}_{1};\xi_{1}\right)}^{⊤},0^{⊤}\right]}^{⊤},\;\frac{\partial }{\partial \xi }{\tilde{\omega }}_{2}\left({\overset{ˇ}{H}}_{2},A_{2},\Theta \right)={\left[{\tilde{\omega }}_{1}\left({\overset{ˇ}{H}}_{1},A_{1},\Theta \right)\varpi_{2}{\left({\overset{ˇ}{H}}_{2};\xi_{2}\right)}^{⊤},0^{⊤}\right]}^{⊤},\;\varpi_{t}\left({\overset{ˇ}{H}}_{t};\xi_{t}\right)\equiv {\overset{ˇ}{H}}_{t1}\left\{1-d_{t}\left(H_{t},\theta_{t}\right)\right\}\left\{1-A_{t}\right\}\frac{\pi_{t}\left({\overset{ˇ}{H}}_{t};\xi_{t}\right)}{1-\pi_{t}\left({\overset{ˇ}{H}}_{t};\xi_{t}\right)}\;-{\overset{ˇ}{H}}_{t1}d_{t}\left({\overset{ˇ}{H}}_{t},\theta_{t}\right)A_{t}\frac{1-\pi_{t}\left({\overset{ˇ}{H}}_{t};\xi_{t}\right)}{\pi_{t}\left({\overset{ˇ}{H}}_{t};\xi_{t}\right)}.$$

## Appendix E. Technical Lemmas

We start with a simple Lemma that will save us some algebra:

**Lemma 14** *For a fixed* ℓ, *let* $X\in {\mathbb{R}}^{\ell }$ *be a random bounded vector and functions* $g_{1}(X),g_{2}(X)$ *be measurable functions of* X. *Let* ${𝕊}_{n}=\{X{\}}_{i=1}^{n}$ *be an i.i.d. sample, and* ${\overset{ˆ}{g}}_{1}(\cdot ),{\overset{ˆ}{g}}_{2}(\cdot )$ *be the estimators for functions* $g_{1},g_{2}\in \mathbb{R}$ *respectively with* $\sup_{X}\left|g_{1}(X)\right|,\sup_{X}\left|g_{2}(X)\right|$, $\sup_{X}\left|{\overset{ˆ}{g}}_{1}(X)\right|,\sup_{X}\left|{\overset{ˆ}{g}}_{2}(X)\right|<\kappa$ *for fixed* κ∈ℝ. *If* ${\mathbb{P}}_{n}\left\{{\overset{ˆ}{g}}_{k}-g_{k}\right\}=O_{\mathbb{P}}\left(n^{-\frac{1}{2}}\right)$, *for* k=1,2, *then* ${\mathbb{P}}_{n}\left\{{\overset{ˆ}{g}}_{1}{\overset{ˆ}{g}}_{2}-g_{1}g_{2}\right\}=O_{\mathbb{P}}\left(n^{-\frac{1}{2}}\right)$.

**Proof** [Proof of Lemma 14] By definition, ${\mathbb{P}}_{n}\left\{{\overset{ˆ}{g}}_{1}{\overset{ˆ}{g}}_{2}-g_{1}g_{2}\right\}=O_{\mathbb{P}}\left(n^{-\frac{1}{2}}\right)$ if and only if for a given any $\epsilon >0,\exists M_{\epsilon }>0$ such that $\mathbb{P}\left(\left|{\mathbb{P}}_{n}\left\{{\overset{ˆ}{g}}_{1}{\overset{ˆ}{g}}_{2}-g_{1}g_{2}\right\}\right|>M_{\epsilon }n^{-\frac{1}{2}}\right)\leq \epsilon \forall n$. Let $M_{\epsilon }>0$,

$$\mathbb{P}\left(\left|{\mathbb{P}}_{n}\left\{g_{1}g_{2}-g_{1}g_{2}\right\}\right|>M_{\epsilon }n^{-\frac{1}{2}}\right)\;=\mathbb{P}\left(\left|{\mathbb{P}}_{n}\left\{{\hat{g}}_{1}{\hat{g}}_{2}-{\hat{g}}_{1}g_{2}+{\hat{g}}_{1}g_{2}-g_{1}g_{2}\right\}\right|>M_{\epsilon }n^{-\frac{1}{2}}\right)\;\;\leq \mathbb{P}\left(\left|{\mathbb{P}}_{n}\left\{{\hat{g}}_{1}\left({\hat{g}}_{2}-g_{2}\right)\right\}\right|+\left|{\mathbb{P}}_{n}\left\{g_{2}\left({\hat{g}}_{1}-g_{1}\right)\right\}\right|>M_{\epsilon }n^{-\frac{1}{2}}\right)\;\;\leq \mathbb{P}\left(\underset{X}{\sup}\;\left|{\hat{g}}_{1}(X)\right|\left|{\mathbb{P}}_{n}\left\{{\hat{g}}_{2}-g_{2}\right\}\right|+\underset{X}{\sup}\;\left|g_{2}(X)\right|\left|{\mathbb{P}}_{n}\left\{{\hat{g}}_{1}-g_{1}\right\}\right|>M_{\epsilon }n^{-\frac{1}{2}}\right)$$

which follows from bounded functions, the union bound, now since ${\mathbb{P}}_{n}\left\{{\overset{ˆ}{g}}_{k}(X)-g_{k}(X)\right\}=O_{\mathbb{P}}\left(n^{-\frac{1}{2}}\right),k=1,2$, there exists $M_{\epsilon }>0$ such that

$$\mathbb{P}\left(\left|{\mathbb{P}}_{n}\left\{{\hat{g}}_{2}-g_{2}\right\}\right|>M_{\epsilon }n^{-\frac{1}{2}}\frac{1}{\kappa }\right)+\mathbb{P}\left(\left|{\mathbb{P}}_{n}\left\{{\hat{g}}_{1}-g_{1}\right\}\right|>M_{\epsilon }n^{-\frac{1}{2}}\frac{1}{\kappa }\right)\leq \frac{\epsilon }{2}+\frac{\epsilon }{2}=\epsilon .$$

■

**Lemma 15** *(Lemma (A.1) (a) in* Chakrabortty et al. (2018)*)*

*Let* $X\in {\mathbb{R}}^{\ell }$ *be any random vector and* $g(X)\in {\mathbb{R}}^{\ell }$ *be any measurable function of* X, *with* ℓ *and* d *fixed. Let* ${𝕊}_{n}=\{X{\}}_{i=1}^{n},{𝕊}_{N}=\{X{\}}_{j=1}^{N}$ *be two random samples of* n *and* N *i.i.d observations of* X *respectively, such that* ${𝕊}_{n}⫫{𝕊}_{N}$. *Let* ${\overset{ˆ}{g}}_{n}(\cdot )$ *be any estimator of* g(⋅) *estimated with* ${𝕊}_{n}$ *such that the random sequence:* ${\overset{ˆ}{T}}_{n}=\sup_{x\in 𝒳}\left\|{\overset{ˆ}{g}}_{n}(\cdot )\right\|=O_{\mathbb{P}}(1)$, *where* $X\in 𝒳\subseteq {\mathbb{R}}^{\ell }$. *Further define the following random sequences:* ${\overset{ˆ}{G}}_{n,N}\equiv \frac{1}{N}\sum_{j=1}^{N}{\overset{ˆ}{g}}_{n}\left(X_{j}\right)$, *and* ${\bar{G}}_{n}\equiv {𝔼}_{{𝕊}_{N}}\left[{\overset{ˆ}{G}}_{n,N}\right]={𝔼}_{X}\left[{\overset{ˆ}{g}}_{n}(X)\right]$, *where* ${𝔼}_{X}$ *is the expectation with respect to* $X\in {𝕊}_{N}$. *We assume all expectations involved are finite almost surely (a.s.)* ${𝕊}_{n}\forall n$. *Then* $G_{n,N}-{\overset{‾}{G}}_{n}=O_{\mathbb{P}}\left(N^{-\frac{1}{2}}\right)$.

**Proof** [Proof of lemma 15]

The following proof follows similar arguments to Chakrabortty et al. (2018). Let ${𝒢}_{n,N}$, ${\bar{𝒢}}_{n}$ be the $j^{\mathrm{th}}$ element of ${\overset{ˆ}{G}}_{n,N}$ and ${\bar{G}}_{n}$ respectively, with j∈{1,…,ℓ}. We show that ${𝒢}_{n,N}-{\bar{𝒢}}_{n}=O_{\mathbb{P}}\left(N^{-\frac{1}{2}}\right)$, which implies Lemma 15 for any ℓ dimensional ${\overset{ˆ}{G}}_{n,N},{\bar{G}}_{n}$. Denote by ${\mathbb{P}}_{{𝕊}_{n}},{\mathbb{P}}_{{𝕊}_{n},{𝕊}_{N}}$ denote the joint probability distributions of samples ${𝕊}_{n}$ and ${𝕊}_{n},{𝕊}_{N}$ respectively. Further let ${𝔼}_{{𝕊}_{n}}[\cdot ]$ denote the expectation with respect to ${𝕊}_{n}$. Since ${𝕊}_{n}⫫{𝕊}_{N}$ using Hoeffding’s inequality

$${\mathbb{P}}_{{𝕊}_{N}}\left(\left|{\hat{𝒢}}_{n,N}-{\hat{𝒢}}_{n}\right|>N^{-\frac{1}{2}}t|\;{𝕊}_{n}\right)\leq 2\text{exp}\left(-\frac{2N^{2}t^{2}}{4N^{2}{\hat{T}}_{n}^{2}}\right)\;\text{a.s.}\;{\mathbb{P}}_{{𝕊}_{n}}.$$

Also, as ${𝕊}_{n}⫫{𝕊}_{N}$ we have

$${\mathbb{P}}_{{𝕊}_{n},{𝕊}_{N}}\left[\left|{\hat{𝒢}}_{n,N}-{\hat{𝒢}}_{n}\right|>N^{-\frac{1}{2}}t\right]={𝔼}_{{𝕊}_{n}}\left[{\mathbb{P}}_{{𝕊}_{N}}\left\{\left|{\hat{𝒢}}_{n,N}-{\hat{𝒢}}_{n}\right|>N^{-\frac{1}{2}}t|{𝕊}_{n}\right\}\right].$$

Next, we have that ${\overset{ˆ}{T}}_{n}=\sup_{x\in 𝒳}\left\|{\overset{ˆ}{g}}_{n}(\cdot )\right\|=O_{\mathbb{P}}(1)$ and is non-negative, thus ∀ϵ>0∃δ(ϵ)>0 such that ${\mathbb{P}}_{{𝕊}_{n}}\left({\overset{ˆ}{T}}_{n}>\delta (\epsilon )\right)<\epsilon /4$, using the above we have that ∀n,N:

$${\mathbb{P}}_{{𝕊}_{n},{𝕊}_{N}}\left(\left|{\hat{𝒢}}_{n,N}-{\hat{𝒢}}_{n}\right|>N^{-\frac{1}{2}}t\right)\leq {𝔼}_{{𝕊}_{n}}\;\left[2\;\text{exp}\left(-\frac{2N^{2}t^{2}}{4N^{2}{\hat{T}}_{n}^{2}}\right)\right]\;={𝔼}_{{𝕊}_{n}}\;\left[2\;\text{exp}\left(-\frac{t^{2}}{2{\hat{T}}_{n}^{2}}\right)\right]={𝔼}_{{𝕊}_{n}}\;\left[2\;\text{exp}\left(-\frac{t^{2}}{2{\hat{T}}_{n}^{2}}\right)\;\left(I\left\{{\hat{T}}_{n}>\delta (\epsilon )\right\}+I\left\{{\hat{T}}_{n}\leq \delta (\epsilon )\right\}\right)\right]\;\leq 2{\mathbb{P}}_{{𝕊}_{n}}\;\left({\hat{T}}_{n}<\delta (\epsilon )\right)+2\;\text{exp}\left(-\frac{t^{2}}{2\delta^{2}(\epsilon )}\right){\mathbb{P}}_{{𝕊}_{n}}\left({\hat{T}}_{n}>\delta (\epsilon )\right)\leq 2\;\text{exp}\left(-\frac{t^{2}}{2\delta^{2}(\epsilon )}\right)+\frac{\epsilon }{2}\leq \frac{2\epsilon }{2}=\epsilon ,$$

where the last step follows from choosing t large enough such that $exp\left(-\frac{t^{2}}{2\delta^{2}(\epsilon )}\right)\leq \epsilon /4$. ■

For Assumption 9 and Lemma 16 we first define some notation and set up the problem. Let $X=\left(X_{1},X_{2}\right)\in {\mathbb{R}}^{\ell_{1}+\ell_{2}}$ be any random vector and $g\left(X_{1}\right)\in \mathbb{R}$ be any measurable function of $X_{1}\in {\mathbb{R}}^{\ell_{1}}$ with $\ell_{1},\ell_{2}$ fixed. Suppose we’re interested in estimating $m\left(X_{2}\right)=𝔼\left[g\left(X_{1}\right)|X_{2}\right]$. Let ${𝕊}_{n}=\{X{\}}_{i=1}^{n}$ be a random sample of n i.i.d. observations of X, and ${𝕊}_{k=1}^{K}$ denote a random partition of ${𝕊}_{n}$ into *K* disjoint subsets of size $n_{K}=\frac{n}{K}$ with index sets ${\left\{{ℐ}_{k}\right\}}_{k=1}^{K}$. We will use cross-validation to estimate $\hat{m}\left(X_{2}\right)$, that is, we use subset ${ℐ}_{k}$ to train estimator ${\hat{m}}_{k}$ and we estimate $m\left(X_{2}\right)$ with: $\hat{m}\left(X_{2}\right)=K^{-1}\sum_{k=1}^{K}\sum_{i\in {ℐ}_{k}}{\hat{m}}_{k}\left(X_{2}\right),K\geq 2$. Denote by ${\overset{ˆ}{C}}_{n,N}\in \mathbb{R}$ an estimator which depends on both samples ${𝕊}_{n},{𝕊}_{N}$. Additionally, let function ${\overset{ˆ}{\pi }}_{n}(\cdot ):{\mathbb{R}}^{\ell_{2}}\to (0,1)$ be a random function with limit $\pi (\cdot ),{\overset{ˆ}{l}}_{n}\left(X_{2}\right):{\mathbb{R}}^{\ell_{2}}\to \{0,1\}$, be a random function with limit $l\left(X_{2}\right)$, and finally function $f:{\mathbb{R}}^{\ell_{2}}\to {\mathbb{R}}^{d},d\leq \ell_{2}$ be any deterministic function of $X_{2}$.

**Assumption 9** *Let* $𝒳\subset {\mathbb{R}}^{p}$ *for an arbitrary* p∈ℕ *i) function* w:𝒳↦ℝ *and estimator* ${\overset{ˆ}{\pi }}_{n}$ *are such that* $\sup_{X_{2}}\left|{\overset{ˆ}{\pi }}_{n}{\left(X_{2}\right)}^{-1}-\pi {\left(X_{2}\right)}^{-1}\right|=O_{\mathbb{P}}\left(n^{-\frac{1}{2}}\right)$, *ii) function* l:𝒳↦{0,1} *and estimator* ${\overset{ˆ}{l}}_{n}$ *are such that* $\sup_{X_{2}}\left|{\overset{ˆ}{l}}_{n}\left(X_{2}\right)-l\left(X_{2}\right)\right|=O_{\mathbb{P}}\left(n^{-\frac{1}{2}}\right)$, *and iii) function* $f\;:\;{\mathbb{R}}^{\ell_{2}}\to {\mathbb{R}}^{d},d\leq \ell_{2}$ *is such that* $\sup_{X_{2}}\left\|f\left(X_{2}\right)\right\|<\infty$.

**Lemma 16** *Define* ${\overset{ˆ}{G}}_{k}^{n}\left(X_{2}\right)={\overset{ˆ}{C}}_{n,N}\frac{{\overset{ˆ}{l}}_{n}\left(X_{2}\right)}{{\overset{ˆ}{\pi }}_{n}\left(X_{2}\right)}f\left(X_{2}\right){\overset{ˆ}{\Delta }}_{k}\left(X_{2}\right)-𝔼\left[\frac{l\left(X_{2}\right)}{\pi \left(X_{2}\right)}f\left(x_{2}\right){\overset{ˆ}{\Delta }}_{k}\left(X_{2}\right)\right]$ *for* ${\overset{ˆ}{\Delta }}_{k}\left(X_{2}\right)={\hat{m}}_{k}\left(X_{2}\right)-m\left(X_{2}\right)$, *and* ${\overset{ˆ}{C}}_{n,N}\in \mathbb{R}$ *which satisfies* $\overset{ˆ}{C}=1+O_{\mathbb{P}}\left(n^{-\frac{1}{2}}\right)$. *Under Assumptions 6 and 9, there is* $c_{n_{K}^{-}}=o(1)$ *such that* ${𝔾}_{n,K}=n^{-\frac{1}{2}}\sum_{k=1}^{K}\sum_{i\in {ℐ}_{k}}{\overset{ˆ}{G}}_{k}^{n}\left(X_{2}\right)=O_{\mathbb{P}}\left(c_{n_{K}^{-}}\right)$,

**Proof** [Proof of Lemma 16] First we define

$${𝒢}_{k}^{\left(n\right)}=n^{-\frac{1}{2}}\sum_{i\in {ℐ}_{k}}\frac{l\left(X_{2i}\right)}{\pi \left(X_{2i}\right)}f\left(X_{2i}\right){\hat{\text{Δ}}}_{k}\left(X_{2i}\right)-𝔼\left[\frac{l\left(X_{2i}\right)}{\pi \left(X_{2i}\right)}f\left(X_{2i}\right){\hat{\text{Δ}}}_{k}\left(X_{2i}\right)\right],$$

for any sample subset ${𝕊}_{K}\subseteq ℒ$, let ${\mathbb{P}}_{{𝕊}_{K}}$ denote the joint probability distribution of ${𝕊}_{K}$, and let ${𝔼}_{{𝕊}_{K}}[\cdot ]$ denote expectation with respect to ${\mathbb{P}}_{{𝕊}_{K}}$, and ${𝔾}_{n,K}=K^{-\frac{1}{2}}\sum_{k=1}^{K}{𝒢}_{k}^{\left(n\right)}$, Next by Assumption 6 we have $\hat{d_{k}}\equiv \sup_{X_{2}}\overset{ˆ}{\Delta }\left(X_{2}\right)=o_{\mathbb{P}}(1)$. Finally let $B_{1}=\sup_{X_{2}}{\left\|f\left(X_{2}\right)\right\|}_{2}<\infty$, $B_{2}<\infty$ be the upperbound to $\sup_{X_{2}}\left|\pi {\left(X_{2}\right)}^{-1}\right|,\sup_{X_{2}}\left|{\overset{ˆ}{l}}_{n}\left(X_{2}\right)\right|\sup_{X_{2}}\left|\frac{{\overset{ˆ}{l}}_{n}\left(X_{2}\right)}{{\overset{ˆ}{\pi }}_{n}\left(X_{2}\right)}\right|$.

First note that

$${\left\|{𝔾}_{n,K}\right\|}_{2}\;={\left\|n^{-\frac{1}{2}}\sum_{k=1}^{K}\sum_{i\in {ℐ}_{k}}{\hat{C}}_{n,N}\frac{{\hat{l}}_{n}\left(X_{2i}\right)}{{\hat{\pi }}_{n}\left(X_{2i}\right)}f\left(X_{2i}\right){\hat{\text{Δ}}}_{k}\left(X_{2i}\right)-𝔼\left[\frac{l\left(X_{2i}\right)}{\pi \left(X_{2i}\right)}f\left(X_{2i}\right){\hat{\text{Δ}}}_{k}\left(X_{2i}\right)\right]\right\|}_{2}\;\leq {\left\|\left({\hat{C}}_{n,N}-1\right)n^{-\frac{1}{2}}\sum_{k=1}^{K}\sum_{i\in {ℐ}_{k}}f\left(X_{2i}\right){\hat{\text{Δ}}}_{k}\left(X_{2i}\right)\frac{{\hat{l}}_{n}\left(X_{2i}\right)}{{\hat{\pi }}_{n}\left(X_{2i}\right)}\right\|}_{2}\;+{\left\|n^{-\frac{1}{2}}\sum_{k=1}^{K}\sum_{i\in {ℐ}_{k}}f\left(X_{2i}\right){\hat{\text{Δ}}}_{k}\left(X_{2i}\right){\hat{l}}_{n}\left(X_{2i}\right)\left(\frac{1}{{\hat{\pi }}_{n}\left(X_{2i}\right)}-\frac{1}{\pi \left(X_{2i}\right)}\right)\right\|}_{2}\;+{\left\|n^{-\frac{1}{2}}\sum_{k=1}^{K}\sum_{i\in {ℐ}_{k}}f\left(X_{2i}\right){\hat{\text{Δ}}}_{k}\left(X_{2i}\right)\frac{1}{\pi \left(X_{2i}\right)}\left({\hat{l}}_{n}\left(X_{2i}\right)-l\left(X_{2i}\right)\right)\right\|}_{2}\;+{\left\|n^{-\frac{1}{2}}\sum_{k=1}^{K}\sum_{i\in {ℐ}_{k}}\frac{l\left(X_{2i}\right)}{\pi \left(X_{2i}\right)}f\left(X_{2i}\right){\hat{\text{Δ}}}_{k}\left(X_{2i}\right)-𝔼\left[\frac{l\left(X_{2i}\right)}{\pi \left(X_{2i}\right)}f\left(X_{2i}\right){\hat{\text{Δ}}}_{k}\left(X_{2i}\right)\right]\right\|}_{2},$$

which follows from the triangle inequality, next as $f(\cdot ),{\overset{ˆ}{\pi }}_{n}(\cdot {)}^{-1},\pi (\cdot {)}^{-1},{\overset{ˆ}{l}}_{n}(\cdot )$ are bounded $\forall X_{2}\in 𝒳$, and using uniform bounds of $O_{\mathbb{P}}\left(n^{-\frac{1}{2}}\right)$ for the difference terms we have

$${\left\|{𝔾}_{n,K}\right\|}_{2}\leq O_{\mathbb{P}}\left(n^{-\frac{1}{2}}\right)n^{\frac{1}{2}}B_{1}B_{2}\left|\sum_{k=1}^{K}{\hat{d}}_{k}\right|+O_{\mathbb{P}}\left(n^{-\frac{1}{2}}\right)n^{\frac{1}{2}}B_{1}B_{2}\left|\sum_{k=1}^{K}{\hat{d}}_{k}\right|\;+O_{\mathbb{P}}\left(n^{-\frac{1}{2}}\right)n^{\frac{1}{2}}B_{1}B_{2}\left|\sum_{k=1}^{K}{\hat{d}}_{k}\right|+{\left\|\frac{1}{K}\sum_{k=1}^{K}{𝒢}_{k}^{\left(n\right)}\right\|}_{2},\;\leq {\left\|n^{-\frac{1}{2}}\frac{1}{K}\sum_{k=1}^{K}\sum_{i\in {ℐ}_{k}}\frac{l\left(X_{2i}\right)}{\pi \left(X_{2i}\right)}f\left(X_{2i}\right){\hat{\text{Δ}}}_{k}\left(X_{2i}\right)-𝔼\left[\frac{l\left(X_{2i}\right)}{\pi \left(X_{2i}\right)}f\left(X_{2i}\right){\hat{\text{Δ}}}_{k}\left(X_{2i}\right)\right]\right\|}_{2}+o_{\mathbb{P}}(1).$$

where the last step follows from ${\hat{d}}_{k}=o_{\mathbb{P}}(1)$. Next we want to bound the first term above by $c_{n_{K}^{-}}$ in probability, note that ∀ϵ∃M>0 such that

$$\mathbb{P}\left({\left\|\sum_{k=1}^{K}{𝒢}_{k}^{\left(n\right)}\right\|}_{2}>Mc_{n_{K}^{-}}\right)\leq \mathbb{P}\left(K^{-\frac{1}{2}}{\left\|\sum_{k=1}^{K}{𝒢}_{k}^{\left(n\right)}\right\|}_{2}>Mc_{n_{K}^{-}}\right)\;\leq \sum_{k=1}^{K}\mathbb{P}\left({\left\|{𝒢}_{k}^{\left(n\right)}\right\|}_{2}>\frac{Mc_{n_{K}^{-}}}{K^{\frac{1}{2}}}\right)\leq \sum_{k=1}^{K}\sum_{j=1}^{d}\mathbb{P}\left(\left|{𝒢}_{k[j]}^{\left(n\right)}\right|>\frac{Mc_{n_{K}^{-}}}{{\left(Kd\right)}^{\frac{1}{2}}}\right)\;\leq \sum_{k=1}^{K}\sum_{j=1}^{d}{𝔼}_{{ℒ}_{k}^{-}}\left[{\mathbb{P}}_{{ℒ}_{k}}\left(\left|{𝒢}_{k[j]}^{\left(n\right)}\right|>\frac{Mc_{n_{K}^{-}}}{{\left(Kd\right)}^{\frac{1}{2}}}|{ℒ}_{k}^{-}\right)\right],$$

where the first 3 steps follow from applying Boole’s inequality and the triangle inequality, the fourth step follows from iterated expectations for the the event $\left\{\left|{𝒢}_{k[j]}^{\left(n\right)}\right|>\frac{Mc_{n_{K}^{-}}}{(Kd{)}^{\frac{1}{2}}}\right\}$.

Next, we have ${ℒ}_{k}^{-}⫫{ℒ}_{k},\forall k\in \{1,\ldots ,K\}$, thus conditional on ${ℒ}_{k}^{-},n^{\frac{1}{2}}{𝒢}_{k}^{\left(n\right)}$ is a sum of iid centered random vectors ${\left\{\frac{l\left(X_{2i}\right)}{\pi \left(X_{2i}\right)}f\left(X_{2i}\right){\overset{ˆ}{\Delta }}_{k}\left(X_{2i}\right)\right\}}_{i\in {ℐ}_{k}}$ which are bounded a.s. ${\mathbb{P}}_{ℒ}^{-},\forall k,n$. Thus we can apply Hoeffding’s inequality to ${𝒢}_{k[j]}^{\left(n\right)}\forall j$:

(20)
$${\mathbb{P}}_{{ℒ}_{k}}\left(\left|{𝒢}_{k[j]}^{\left(n\right)}\right|>\frac{Mc_{n_{K}^{-}}}{{\left(Kd\right)}^{\frac{1}{2}}}|{ℒ}_{k}^{-}\right)\leq 2\;\text{exp}\left\{-\frac{M^{2}c_{n_{K}^{-}}^{2}}{2KdB^{2}{\hat{d}}_{k}^{2}}\right\}$$

a.s. ${\mathbb{P}}_{{ℒ}_{k}^{-}}\forall n$; and for each k∈{1,…,K},j∈{1…,d}. Note that $\frac{c_{n_{k}^{-}}}{D_{k}}\geq 0$ is stochastically bounded away from zero as ${\hat{d}}_{k}=o_{\mathbb{P}}(1)$, therefore ∀k and given ϵ>0,∃δ(ϵ,k)>0 such that ${\mathbb{P}}_{{ℒ}_{k}^{-}}\left(\frac{c_{n_{K}^{-}}}{D_{k}}\leq \delta (\epsilon ,k)\right)\leq \frac{\epsilon }{4Kd}$, let $\delta^{*}(\epsilon ,k)={min}_{k}\{\delta (\epsilon ,k)\}$, we have that ${\mathbb{P}}_{{ℒ}_{k}^{-}}\left(\frac{c_{n_{K}^{-}}}{D_{k}}\leq \delta^{*}(\epsilon ,k)\right)\leq \frac{\epsilon }{4Kd}$.

Therefore using the bound in (20) and event $\left\{\frac{c_{n_{k}^{-}}}{D_{k}}\leq \delta^{*}(\epsilon ,k)\right\}$:

$$\mathbb{P}\left({\left\|\sum_{k=1}^{K}{𝒢}_{k}^{\left(n\right)}\right\|}_{2}>Mc_{n_{K}^{-}}\right)\;\leq \sum_{k=1}^{K}\sum_{j=1}^{d}{𝔼}_{{ℒ}_{k}^{-}}\left[{\mathbb{P}}_{{ℒ}_{k}}\left(\left|{𝒢}_{k[j]}^{\left(n\right)}\right|>\frac{Mc_{n_{K}^{-}}}{{\left(Kd\right)}^{\frac{1}{2}}}|{ℒ}_{k}^{-}\right)\right]\;\leq \sum_{k=1}^{K}\sum_{j=1}^{d}{𝔼}_{{ℒ}_{k}^{-}}\left[2\;\text{exp}\left\{-\frac{M^{2}c_{n_{K}^{-}}^{2}}{2KdB^{2}{\hat{d}}_{k}^{2}}\right\}\;\left(I\left\{\frac{c_{n_{K}^{-}}}{D_{k}}\leq \delta^{\text{*}}(\epsilon ,k)\right\}+I\left\{\frac{c_{n_{K}^{-}}}{D_{k}}\delta^{\text{*}}(\epsilon ,k)\right\}\right)\right]\;\leq 2Kd\text{exp}\left\{-\frac{M^{2}\delta^{\text{*}}{\left(\epsilon ,k\right)}^{2}}{2KdB^{2}}\right\}{\mathbb{P}}_{{ℒ}_{k}^{-}}\left(\frac{c_{n_{K}^{-}}}{D_{k}}\leq \delta^{\text{*}}(\epsilon ,k)\right)+2Kd{\mathbb{P}}_{{ℒ}_{k}^{-}}\left(\frac{c_{n_{K}^{-}}}{D_{k}}>\delta^{\text{*}}(\epsilon ,k)\right)\;\leq 2Kd\frac{\epsilon }{4Kd}+2Kd\text{exp}\left\{-\frac{M^{2}\delta^{\text{*}}{\left(\epsilon ,k\right)}^{2}}{2KdB^{2}}\right\}{\mathbb{P}}_{{ℒ}_{k}^{-}}\left(\frac{c_{n_{K}^{-}}}{D_{k}}>\delta^{\text{*}}(\epsilon ,k)\right),$$

next note that choosing a large enough M such that $exp\left\{-\frac{M^{2}\delta^{*}(\epsilon ,k{)}^{2}}{2KdB^{2}}\right\}<\frac{\epsilon }{4Kd}$, since ${\mathbb{P}}_{{ℒ}_{k}^{-}}\left(\frac{c_{n_{K}^{-}}}{D_{k}}>\delta^{*}(\epsilon ,k)\leq 1\right)$ we get $\mathbb{P}\left({\left\|\sum_{k=1}^{K}{𝒢}_{k}^{\left(n\right)}\right\|}_{2}>Mc_{n_{K}^{-}}\right)\leq \frac{\epsilon }{2}+\frac{\epsilon }{2}=\epsilon$.

Finally we have

$${𝔾}_{n,K}=O_{\mathbb{P}}\left(c_{n_{K}^{-}}\right)+o_{\mathbb{P}}(1)=O_{\mathbb{P}}\left(c_{n_{K}^{-}}\right).$$

■

**Lemma 17** *Let* $\hat{\gamma }\in {\mathbb{R}}^{d}$ *be a random variable such that* $\sqrt{n}(\hat{\gamma }-\overset{‾}{\gamma })=O_{\mathbb{P}}(1)$, *then for any fixed vector* $a\in {\mathbb{R}}^{d}$ *we have that (a)* $\sqrt{n}\left({\left[a^{⊤}\hat{\gamma }\right]}_{+}-{\left[a^{⊤}\overset{‾}{\gamma }\right]}_{+}\right)=\sqrt{n}(\hat{\gamma }-\overset{‾}{\gamma })I\left(a^{⊤}\overset{‾}{\gamma }>0\right)+o_{\mathbb{P}}(1)$, *(b) Functions* ${\hat{d}}_{t}\;t=1,2$, *defined in* Section 4 *and propensity scores* $\pi_{1}$ *in* (4) *satisfy*

$$\;\underset{H_{1},a_{1}}{\sup}\left|I\left({\hat{d}}_{1}=A_{1}\right)-I\left({\bar{d}}_{1}=A_{1}\right)\right|=O_{\mathbb{P}}\left(n^{-\frac{1}{2}}\right),\;\underset{H_{2},a_{2}}{\sup}\left|I\left({\hat{d}}_{1}=A_{1}\right)I\left(A_{2}={\hat{d}}_{2}\right)-I\left({\bar{d}}_{1}=A_{1}\right)I\left({\bar{d}}_{2}=A_{2}\right)\right|=O_{\mathbb{P}}\left(n^{-\frac{1}{2}}\right),\;\;\underset{H_{1}}{\sup}\left|\frac{1}{\pi_{1}\left(H_{1};{\hat{\xi }}_{1}\right)}-\frac{1}{\pi_{1}\left(H_{1};{\bar{\xi }}_{1}\right)}\right|=O_{\mathbb{P}}\left(n^{-\frac{1}{2}}\right).$$

*(c) For* $\hat{\theta },\hat{\xi }$ *estimated via our semi-supervised approach, and limits* $\bar{\theta },\bar{\xi }$ *defined in Assumptions 3 and 5 respectively*

$${\hat{C}}_{n,N}^{\left(1\right)}=\frac{\left(1+{\hat{\beta }}_{21}\right){\mathbb{P}}_{N}\left\{\omega_{1}\left({\overset{ˇ}{H}}_{1},A_{1};{\bar{\Theta }}_{1}\right)\right\}}{\left(1+{\hat{\beta }}_{21}\right){\mathbb{P}}_{n}\left\{\omega_{1}\left({\overset{ˇ}{H}}_{1},A_{1};{\hat{\Theta }}_{1}\right)\right\}},\;{\hat{C}}_{n,N}^{\left(2\right)}=\frac{{\mathbb{P}}_{N}\left\{Q_{2-}^{o}\left(H_{2},A_{2};{\bar{\theta }}_{2}\right)\right\}}{{\mathbb{P}}_{n}\left\{Q_{2-}^{o}\left(H_{2},A_{2};{\hat{\theta }}_{2}\right)\right\}},$$

*satisfy* ${\overset{ˆ}{C}}_{n,N}^{\left(1\right)}=1+O_{\mathbb{P}}\left(n^{-\frac{1}{2}}\right),{\overset{ˆ}{C}}_{n,N}^{\left(2\right)}=1+O_{\mathbb{P}}\left(n^{-\frac{1}{2}}\right)$.

**Proof** [Proof of Lemma 17]

Define set ${𝒜}_{q}$ for any *q* dimensional vector $\overset{ˆ}{\gamma }$ as

$${𝒜}_{q}=\left\{\hat{\gamma }\in {\mathbb{R}}^{q}|\frac{1}{2}a^{⊤}\bar{\gamma }<a^{⊤}\hat{\gamma }<2a^{⊤}\bar{\gamma },\;\forall a\in {\mathbb{R}}^{q}\right\}.$$

Now consider $\hat{\gamma }\in {𝒜}_{q}$ :

- if $sign\left(a^{⊤}\overset{‾}{\gamma }\right)=1$, then $0<\frac{1}{2}a^{⊤}\overset{‾}{\gamma }<a^{⊤}\hat{\gamma }⟹sign\left(a^{⊤}\hat{\gamma }\right)=1$,
- if $sign\left(a^{⊤}\bar{\gamma }\right)=-1$, then $a^{⊤}\hat{\gamma }<2a^{⊤}\bar{\gamma }<0⟹sign\left(a^{⊤}\hat{\gamma }\right)=-1$.

Assuming $\sqrt{n}(\hat{\gamma }-\overset{‾}{\gamma })=O_{\mathbb{P}}(1)$, ${𝒜}_{q}$ exists and in fact it is such that $\mathbb{P}\left(\hat{\gamma }\in {𝒜}_{q}\right)\overset{p}{\to }1$.

- Using the above:
  $$\sqrt{n}\left({\left[a^{⊤}\hat{\gamma }\right]}_{+}-{\left[a^{⊤}\bar{\gamma }\right]}_{+}\right)=\sqrt{n}(\hat{\gamma }-\bar{\gamma })I\left(a^{⊤}\bar{\gamma }>0\right)I\left(\hat{\gamma }\in {𝒜}_{q}\right)+\sqrt{n}\left({\left[a^{⊤}\hat{\gamma }\right]}_{+}-{\left[a^{⊤}\bar{\gamma }\right]}_{+}\right)I\left(\hat{\gamma }\notin {𝒜}_{q}\right)\;=\sqrt{n}(\hat{\gamma }-\bar{\gamma })I\left(a^{⊤}\bar{\gamma }>0\right)+o_{\mathbb{P}}(1).$$
- As $A_{ti}\in \{0,1\},t=1,2$, we can write
  $$I\left({\hat{d}}_{1}=A_{1}\right)I\left({\hat{d}}_{2}=A_{2}\right)=I\left\{A_{1}=I\left(H_{11}^{⊤}{\hat{\gamma }}_{1}>0\right)\right\}I\left\{A_{2}=I\left(H_{21}^{⊤}{\hat{\gamma }}_{2}>0\right)\right\}\;=I\left\{A_{1}=I\left(H_{11}^{⊤}{\hat{\gamma }}_{1}>0\right)\right\}I\left\{A_{2}=I\left(H_{21}^{⊤}{\hat{\gamma }}_{2}>0\right)\right\}\;=A_{1}A_{2}I\left(H_{11}^{⊤}{\hat{\gamma }}_{1}>0\right)I\left(H_{21}^{⊤}{\hat{\gamma }}_{2}>0\right)\;+\left(1-A_{1}\right)\left(1-A_{2}\right)I\left(H_{11}^{⊤}{\hat{\gamma }}_{1}<0\right)I\left(H_{21}^{⊤}{\hat{\gamma }}_{2}<0\right)\;+A_{1}\left(1-A_{2}\right)I\left(H_{11}^{⊤}{\hat{\gamma }}_{1}>0\right)I\left(H_{21}^{⊤}{\hat{\gamma }}_{2}<0\right)\;+\left(1-A_{1}\right)A_{2}I\left(H_{11}^{⊤}{\hat{\gamma }}_{1}<0\right)I\left(H_{21}^{⊤}{\hat{\gamma }}_{2}>0\right),$$
  therefore
  $$\left|I\left({\hat{d}}_{1}=A_{1}\right)I\left({\hat{d}}_{2}=A_{2}\right)-I\left({\bar{d}}_{1}=A_{1}\right)I\left({\bar{d}}_{2}=A_{2}\right)\right|\;=|A_{1}A_{2}\left\{I\left(H_{11}^{⊤}{\hat{\gamma }}_{1}>0\right)I\left(H_{21}^{⊤}{\hat{\gamma }}_{2}>0\right)-I\left(H_{11}^{⊤}{\bar{\gamma }}_{1}>0\right)I\left(H_{21}^{⊤}{\bar{\gamma }}_{2}>0\right)\right\}\;+\left(1-A_{1}\right)\left(1-A_{2}\right)\left\{I\left(H_{11}^{⊤}{\hat{\gamma }}_{1}<0\right)I\left(H_{21}^{⊤}{\hat{\gamma }}_{2}<0\right)-I\left(H_{11}^{⊤}{\bar{\gamma }}_{1}<0\right)I\left(H_{21}^{⊤}{\bar{\gamma }}_{2}<0\right)\right\}\;+A_{1}\left(1-A_{2}\right)\left\{I\left(H_{11}^{⊤}{\hat{\gamma }}_{1}>0\right)I\left(H_{21}^{⊤}{\hat{\gamma }}_{2}<0\right)-I\left(H_{11}^{⊤}{\bar{\gamma }}_{1}>0\right)I\left(H_{21}^{⊤}{\bar{\gamma }}_{2}<0\right)\right\}\;+\left(1-A_{1}\right)A_{2}\left\{I\left(H_{11}^{⊤}{\hat{\gamma }}_{1}<0\right)I\left(H_{21}^{⊤}{\hat{\gamma }}_{2}>0\right)-I\left(H_{11}^{⊤}{\bar{\gamma }}_{1}<0\right)I\left(H_{21}^{⊤}{\bar{\gamma }}_{2}>0\right)\right\}|\;\leq A_{1}A_{2}\left|I\left(H_{11}^{⊤}{\hat{\gamma }}_{1}>0\right)I\left(H_{21}^{⊤}{\hat{\gamma }}_{2}>0\right)-I\left(H_{11}^{⊤}{\bar{\gamma }}_{1}>0\right)I\left(H_{21}^{⊤}{\bar{\gamma }}_{2}>0\right)\right|\;+\left(1-A_{1}\right)\left(1-A_{2}\right)\left|I\left(H_{11}^{⊤}{\hat{\gamma }}_{1}<0\right)I\left(H_{21}^{⊤}{\hat{\gamma }}_{2}<0\right)-I\left(H_{11}^{⊤}{\bar{\gamma }}_{1}<0\right)I\left(H_{21}^{⊤}{\bar{\gamma }}_{2}<0\right)\right|\;+A_{1}\left(1-A_{2}\right)\left|I\left(H_{11}^{⊤}{\hat{\gamma }}_{1}>0\right)I\left(H_{21}^{⊤}{\hat{\gamma }}_{2}<0\right)-I\left(H_{11}^{⊤}{\bar{\gamma }}_{1}>0\right)I\left(H_{21}^{⊤}{\bar{\gamma }}_{2}<0\right)\right|\;+\left(1-A_{1}\right)A_{2}\left|I\left(H_{11}^{⊤}{\hat{\gamma }}_{1}<0\right)I\left(H_{21}^{⊤}{\hat{\gamma }}_{2}>0\right)-I\left(H_{11}^{⊤}{\bar{\gamma }}_{1}<0\right)I\left(H_{21}^{⊤}{\bar{\gamma }}_{2}>0\right)\right|$$
  where the first step follows from above, the second step from the triangle inequality, now as ${\hat{\gamma }}_{1},{\hat{\gamma }}_{2}$ have dimensions $q_{12},q_{22}$ respectively, we use sets ${𝒜}_{q_{12}},{𝒜}_{q_{22}}$ and have
  $$\left|I\left({\hat{d}}_{1}=A_{1}\right)I\left({\hat{d}}_{2}=A_{2}\right)-I\left({\bar{d}}_{1}=A_{1}\right)I\left({\bar{d}}_{2}=A_{2}\right)\right|\;\leq A_{1}A_{2}I\left({\hat{\gamma }}_{1}\notin {𝒜}_{q_{12}}\right)I\left({\hat{\gamma }}_{2}\notin {𝒜}_{q_{22}}\right)+\left(1-A_{1}\right)\left(1-A_{2}\right)I\left({\hat{\gamma }}_{1}\notin {𝒜}_{q_{12}}\right)I\left({\hat{\gamma }}_{2}\notin {𝒜}_{q_{22}}\right)\;+A_{1}\left(1-A_{2}\right)I\left({\hat{\gamma }}_{1}\notin {𝒜}_{q_{12}}\right)I\left({\hat{\gamma }}_{2}\notin {𝒜}_{q_{22}}\right)+\left(1-A_{1}\right)A_{2}I\left({\hat{\gamma }}_{1}\notin {𝒜}_{q_{12}}\right)I\left({\hat{\gamma }}_{2}\notin {𝒜}_{q_{22}}\right)\;=I\left({\hat{\gamma }}_{1}\notin {𝒜}_{q_{12}}\right)I\left({\hat{\gamma }}_{2}\notin {𝒜}_{q_{22}}\right)$$
  which follows from the fact that for any term within absolute value:
  $$\left|I\left(H_{11}^{⊤}{\hat{\gamma }}_{1}<0\right)I\left(H_{21}^{⊤}{\hat{\gamma }}_{2}>0\right)-I\left(H_{11}^{⊤}{\bar{\gamma }}_{1}<0\right)I\left(H_{21}^{⊤}{\bar{\gamma }}_{2}>0\right)\right|=I\left({\hat{\gamma }}_{1}\notin {𝒜}_{q_{12}}\right)I\left({\hat{\gamma }}_{2}\notin {𝒜}_{q_{22}}\right)$$
  since for $I\left(H_{11}^{⊤}{\overset{‾}{\gamma }}_{1}<0\right)I\left(H_{21}^{⊤}{\overset{‾}{\gamma }}_{2}>0\right)\neq I\left(H_{11}^{⊤}{\hat{\gamma }}_{1}<0\right)I\left(H_{21}^{⊤}{\hat{\gamma }}_{2}>0\right)$ both ${\hat{\gamma }}_{1},{\hat{\gamma }}_{2}$ have to be outside sets ${𝒜}_{q_{12}},{𝒜}_{q_{22}}$ respectively. Thus $|I\left({\hat{d}}_{1}=A_{1}\right)I\left({\hat{d}}_{2}=A_{2}\right)-I\left({\overset{‾}{d}}_{1}=A_{1}\right)I\left({\overset{‾}{d}}_{2}=A_{2}\right)\left|=O_{\mathbb{P}}\left(n^{-\frac{1}{2}}\right)\right.$, we can analogous show that $\left|I\left({\hat{d}}_{1}=A_{1}\right)-I\left({\overset{‾}{d}}_{1}=A_{1}\right)\right|=O_{\mathbb{P}}\left(n^{-\frac{1}{2}}\right)\;\forall i$. Next to see $\sup_{H_{1}}\left|\frac{1}{\pi_{1}\left(H_{1};{\overset{ˆ}{\xi }}_{1}\right)}-\frac{1}{\pi_{1}\left(H_{1};{\overset{¯}{\xi }}_{1}\right)}\right|=O_{\mathbb{P}}\left(n^{-\frac{1}{2}}\right)$, note that as ${ℋ}_{1},\Omega_{1}$ are bounded sets we have
  $$\underset{H_{1}}{\sup}\left|\frac{1}{\pi_{1}\left(H_{1};{\hat{\xi }}_{1}\right)}-\frac{1}{\pi_{1}\left(H_{1};{\bar{\xi }}_{1}\right)}\right|=\underset{H_{1}\in {ℋ}_{1}}{\sup}\left|e^{-H_{1}^{⊤}{\hat{\xi }}_{1}}-e^{-H_{1}^{⊤}{\bar{\xi }}_{1}}\right|\;\leq \underset{H_{1}\in {ℋ}_{1},\xi_{1}\in {\text{Ω}}_{1}}{\sup}\left|\frac{d}{dx}{e^{-x}|}_{x=H_{1}^{⊤}\xi_{1}}\right|\underset{H_{1}\in {ℋ}_{1}}{\sup}\left|H_{1}^{⊤}{\hat{\xi }}_{1}-H_{1}^{⊤}\bar{\xi }\right|\;\leq \underset{H_{1}\in {ℋ}_{1},\xi_{1}\in {\text{Ω}}_{1}}{\sup}\left|\frac{d}{dx}{e^{-x}|}_{x=H_{1}^{⊤}\xi_{1}}\right|\underset{H_{1}\in {ℋ}_{1}}{\sup}\left\|H_{1}\right\|{\left\|{\hat{\xi }}_{1}-{\bar{\xi }}_{1}\right\|}_{2}=O_{\mathbb{P}}\left(n^{-\frac{1}{2}}\right),$$
  where we use the definition of $\pi_{1}$ in (4), Lipschitz and ${\left\|{\overset{ˆ}{\xi }}_{1}-{\bar{\xi }}_{1}\right\|}_{2}=O_{\mathbb{P}}\left(n^{-\frac{1}{2}}\right)$ from Assumptions (5) and Theorem 5.21 in Vart (1998) as we are using Z-estimation for $\xi_{1}$.
- By Theorem 2 we have ${\overset{ˆ}{\beta }}_{21}-{\overset{‾}{\beta }}_{21}=O_{\mathbb{P}}\left(n^{-\frac{1}{2}}\right)$. Next, we can write
  $$\omega_{1}\left(H_{1},A_{1};{\hat{\Theta }}_{1}\right)=I\left\{A_{1}=d_{1}\left(H_{1};{\hat{\xi }}_{1}\right)\right\}\;\left\{\frac{A_{1}}{\pi_{1}\;\left(H_{1};{\hat{\xi }}_{1}\right)}+\frac{1-A_{1}}{1-\pi_{1}\;\left(H_{1};{\hat{\xi }}_{1}\right)}\right\}.$$

By Lemma 17 (b) it follows that

$${\mathbb{P}}_{n}\;\left[I\left\{A_{1}=d_{1}\;\left(H_{1};{\hat{\xi }}_{1}\right)\right\}-I\;\left\{A_{1}=d_{1}\;\left(H_{1};{\bar{\xi }}_{1}\right)\right\}\right]=O_{\mathbb{P}}\left(n^{-\frac{1}{2}}\right),\;\;{\mathbb{P}}_{n}\left[\frac{A_{1}}{\pi_{1}\left(H_{1};{\hat{\xi }}_{1}\right)}-\frac{A_{1}}{\pi_{1}\;\left(H_{1};{\bar{\xi }}_{1}\right)}\right]=O_{\mathbb{P}}\left(n^{-\frac{1}{2}}\right),\;\;{\mathbb{P}}_{n}\;\left[\frac{1-A_{1}}{1-\pi_{1}\left(H_{1};{\hat{\xi }}_{1}\right)}-\frac{1-A_{1}}{1-\pi_{1}\;\left(H_{1};{\bar{\xi }}_{1}\right)}\right]=O_{\mathbb{P}}\left(n^{-\frac{1}{2}}\right).$$

Using the above and Lemma 14 we get

$$\left(1+{\hat{\beta }}_{21}\right){\mathbb{P}}_{n}\left\{\omega_{1}\left({\overset{ˇ}{H}}_{1},A_{1};{\hat{\Theta }}_{1}\right)\right\}=\left(1+{\bar{\beta }}_{21}\right){\mathbb{P}}_{n}\left\{\omega_{1}\left({\overset{ˇ}{H}}_{1},A_{1};{\bar{\Theta }}_{1}\right)\right\}+O_{\mathbb{P}}\left(n^{-\frac{1}{2}}\right)$$

Also by CLT we have

$$\left(1+{\bar{\beta }}_{21}\right){\mathbb{P}}_{n}\left\{\omega_{1}\left({\overset{ˇ}{H}}_{1},A_{1};{\bar{\Theta }}_{1}\right)\right\}=\left(1+{\bar{\beta }}_{21}\right)𝔼\left\{\omega_{1}\left({\overset{ˇ}{H}}_{1},A_{1};{\bar{\Theta }}_{1}\right)\right\}+O_{\mathbb{P}}\left(n^{-\frac{1}{2}}\right),\;\left(1+{\bar{\beta }}_{21}\right){\mathbb{P}}_{N}\;\left\{\omega_{1}\left({\overset{ˇ}{H}}_{1},A_{1};{\bar{\Theta }}_{1}\right)\right\}=\left(1+{\bar{\beta }}_{21}\right)𝔼\left\{\omega_{1}\left({\overset{ˇ}{H}}_{1},A_{1};{\bar{\Theta }}_{1}\right)\right\}+O_{\mathbb{P}}\left(N^{-\frac{1}{2}}\right),$$

finally by Slutsky’s theorem ${\overset{ˆ}{C}}_{n,N}^{\left(1\right)}-1=O_{\mathbb{P}}\left(n^{-\frac{1}{2}}\right)$. With similar arguments, and using Lemma 17 (a) to see ${\mathbb{P}}_{n}\left({\left[H_{21}^{⊤}{\hat{\gamma }}_{2}\right]}_{+}-{\left[H_{21}^{⊤}{\overset{‾}{\gamma }}_{2}\right]}_{+}\right)=O_{\mathbb{P}}\left(n^{-\frac{1}{2}}\right)$, we can show ${\overset{ˆ}{C}}_{n,N}^{\left(2\right)}-1=O_{\mathbb{P}}\left(n^{-\frac{1}{2}}\right)$.

■

**Lemma 18** *Let* $Q_{t}\left({\overset{ˇ}{H}}_{t};\theta_{t}\right),\pi_{t}\left({\overset{ˇ}{H}}_{t};\xi_{t}\right)\;t=1, 2$ *be estimator functions of* (1) & (4) *respectively and define the bias as Bias* $\left(\overset{‾}{V},{𝒱}_{{\mathrm{SUP}}_{DR}}(L;\Theta )\right)\equiv \overset{‾}{V}-𝔼\left[{𝒱}_{{\mathrm{SUP}}_{DR}}(L;\Theta )\right]$, *then*

$$\mathrm{Bias}\left(\bar{V},{𝒱}_{{\text{SUP}}_{\text{DR}}}(L;\Theta )\right)\;=𝔼\left[\left\{1-\frac{\pi_{1}\left({\overset{ˇ}{H}}_{1}\right)}{\pi_{1}\left({\overset{ˇ}{H}}_{1};\xi_{1}\right)}\right\}\;\left\{Q_{1}^{o}\left({\overset{ˇ}{H}}_{1}\right)-Q_{1}^{o}\left({\overset{ˇ}{H}}_{1};\theta_{1}\right)\right\}\right]\;+𝔼\;\left[\left\{1-\frac{1-\pi_{1}\left({\overset{ˇ}{H}}_{1}\right)}{1-\pi_{1}\left({\overset{ˇ}{H}}_{1};\xi_{1}\right)}\right\}\;\left\{Q_{1}^{o}\left({\overset{ˇ}{H}}_{1}\right)-Q_{1}^{o}\left({\overset{ˇ}{H}}_{1};\theta_{1}\right)\right\}\right]\;+𝔼\;\left[\left\{\frac{A_{1}}{\pi_{1}\left({\overset{ˇ}{H}}_{1};\xi_{1}\right)}+\frac{1-A_{1}}{1-\pi_{1}\left({\overset{ˇ}{H}}_{1};\xi_{1}\right)}\right\}\;\left\{1-\frac{\pi_{2}\left({\overset{ˇ}{H}}_{2}\right)}{\pi_{2}\left({\overset{ˇ}{H}}_{2};\xi_{2}\right)}\right\}\;\left\{Q_{2}^{o}\left({\overset{ˇ}{H}}_{2}\right)-Q_{2}^{o}\left({\overset{ˇ}{H}}_{2};\theta_{2}\right)\right\}\right]\;+𝔼\;\left[\left\{\frac{A_{1}}{\pi_{1}\left({\overset{ˇ}{H}}_{1};\xi_{1}\right)}+\frac{1-A_{1}}{1-\pi_{1}\left({\overset{ˇ}{H}}_{1};\xi_{1}\right)}\right\}\;\left\{1-\frac{1-\pi_{2}\left({\overset{ˇ}{H}}_{2}\right)}{1-\pi_{2}\left({\overset{ˇ}{H}}_{2};\xi_{2}\right)}\right\}\;\left\{Q_{2}^{o}\left({\overset{ˇ}{H}}_{2}\right)-Q_{2}^{o}\left({\overset{ˇ}{H}}_{2};\theta_{2}\right)\right\}\right].$$

*where* $\overset{‾}{V}=𝔼\left[𝔼\left[Y_{2}+𝔼\left[Y_{3}|H_{2},Y_{2},A_{2}={\overset{‾}{d}}_{2}\left({\overset{ˇ}{H}}_{2}\right)\right]|H_{1},A_{1}={\overset{‾}{d}}_{1}\left({\overset{ˇ}{H}}_{1}\right)\right]\right]$ *is the mean population value under the optimal treatment rule*.

**Proof** [Proof of Lemma 18]

$$\text{Bias}\left(\bar{V},{𝒱}_{{\text{SUP}}_{\text{DR}}}(L;\Theta )\right)=𝔼\left[𝔼\left[Y_{2}+𝔼\left[Y_{3}|H_{2},Y_{2},A_{2}={\bar{d}}_{2}\right]|H_{1},A_{1}={\bar{d}}_{1}\right]\right]-𝔼\left[{𝒱}_{{\text{SUP}}_{\text{DR}}}(L;\Theta )\right]\;=𝔼\left[Q_{1}^{o}\left(H_{1}\right)-Q_{1}^{o}\left(H_{1};\theta_{1}\right)\right]\;-𝔼\left[\omega_{1}\left({\overset{ˇ}{H}}_{1},A_{1};\Theta_{1}\right)\left\{Y_{2}-Q_{1}^{o}\left({\overset{ˇ}{H}}_{1};\theta_{1}\right)\right\}\right]\;-𝔼\left[\omega_{1}\left({\overset{ˇ}{H}}_{1},A_{1};\Theta_{1}\right)Q_{2}^{o}\left({\overset{ˇ}{H}}_{2};\theta_{2}\right)\right]\;-𝔼\left[\omega_{2}\left({\overset{ˇ}{H}}_{2},A_{2};\Theta_{2}\right)\left\{Y_{3}-Q_{2}^{o}\left({\overset{ˇ}{H}}_{2};\theta_{2}\right)\right\}\right].$$

Adding and subtracting $𝔼\left[\omega_{1}\left({\overset{ˇ}{H}}_{1},A_{1};\Theta_{1}\right)Q_{2}^{o}\left({\overset{ˇ}{H}}_{2}\right)\right]=𝔼\left[\omega_{1}\left({\overset{ˇ}{H}}_{1},A_{1};\Theta_{1}\right)𝔼\left[Y_{3}|H_{2},{\overset{‾}{d}}_{2}\left({\overset{ˇ}{H}}_{2};\theta_{2}\right),Y_{2}\right]\right]$,

$$\text{Bias}\left(\bar{V},{𝒱}_{{\text{SUP}}_{\text{DR}}}(L;\Theta )\right)\;=𝔼\left[Q_{1}^{o}\left(H_{1}\right)-Q_{1}^{o}\left(H_{1};\theta_{1}\right)\right]\;-𝔼\left[\omega_{1}\left({\overset{ˇ}{H}}_{1},A_{1};\Theta_{1}\right)\left\{Y_{2}+𝔼\left[Y_{3}|H_{2}{\bar{d}}_{2}\left({\overset{ˇ}{H}}_{2};\theta_{2}\right),Y_{2}\right]-Q_{1}^{o}\left({\overset{ˇ}{H}}_{1};\theta_{1}\right)\right\}\right]\;-𝔼\left[\omega_{1}\left({\overset{ˇ}{H}}_{1},A_{1};\Theta_{1}\right)\left\{Q_{2}^{o}\left({\overset{ˇ}{H}}_{2};\theta_{2}\right)-Q_{2}^{o}\left({\overset{ˇ}{H}}_{2}\right)\right\}\right]\;-𝔼\left[\omega_{2}\left({\overset{ˇ}{H}}_{2},A_{2};\Theta_{2}\right)\left\{Y_{3}-Q_{2}^{o}\left({\overset{ˇ}{H}}_{2};\theta_{2}\right)\right\}\right],$$

using iterated expectations in the second and fourth terms:

$$\text{Bias}\left(\bar{V},{𝒱}_{{\text{SUP}}_{\text{DR}}}(L;\Theta )\right)\;=𝔼\left[Q_{1}^{o}\left({\overset{ˇ}{H}}_{1}\right)-Q_{1}^{o}\left({\overset{ˇ}{H}}_{1};\theta_{1}\right)\right]\;-𝔼\left[𝔼\left[\omega_{1}\left({\overset{ˇ}{H}}_{1},A_{1};\Theta_{1}\right)\left\{Y_{2}+𝔼\left[Y_{3}|{\overset{ˇ}{H}}_{2},{\bar{d}}_{2}\left({\overset{ˇ}{H}}_{2}\right),Y_{2}\right]-Q_{1}^{o}\left({\overset{ˇ}{H}}_{1};\theta_{1}\right)\right\}|{\overset{ˇ}{H}}_{1},A_{1}\right]\right]\;-𝔼\left[\omega_{1}\left({\overset{ˇ}{H}}_{1},A_{1};\Theta_{1}\right)\left\{Q_{2}^{o}\left({\overset{ˇ}{H}}_{2};\theta_{2}\right)-Q_{2}^{o}\left({\overset{ˇ}{H}}_{2}\right)\right\}\right]\;-𝔼\left[𝔼\left[\omega_{2}\left({\overset{ˇ}{H}}_{2},A_{2};\Theta_{2}\right)\left\{Y_{3}-Q_{2}^{o}\left({\overset{ˇ}{H}}_{2};\theta_{2}\right)\right\}|{\overset{ˇ}{H}}_{2},A_{2},Y_{2}\right]\right]\;=𝔼\left[Q_{1}^{o}\left({\overset{ˇ}{H}}_{1}\right)-Q_{1}^{o}\left({\overset{ˇ}{H}}_{1};\theta_{1}\right)\right]\;-𝔼\left[\omega_{1}\left({\overset{ˇ}{H}}_{1},A_{1};\Theta_{1}\right)\left\{𝔼\left[Y_{2}+𝔼\left[Y_{3}|{\overset{ˇ}{H}}_{2},{\bar{d}}_{2}\left({\overset{ˇ}{H}}_{2}\right),Y_{2}\right]|{\overset{ˇ}{H}}_{1},A_{1}\right]-Q_{1}^{o}\left({\overset{ˇ}{H}}_{1};\theta_{1}\right)\right\}\right]\;-𝔼\left[\omega_{1}\left({\overset{ˇ}{H}}_{1},A_{1};\Theta_{1}\right)\left\{Q_{2}^{o}\left({\overset{ˇ}{H}}_{2};\theta_{2}\right)-Q_{2}^{o}\left({\overset{ˇ}{H}}_{2}\right)\right\}\right]\;-𝔼\left[\omega_{2}\left({\overset{ˇ}{H}}_{2},A_{2};\Theta_{2}\right)\left\{𝔼\left[Y_{3}|{\overset{ˇ}{H}}_{2},A_{2},Y_{2}\right]-Q_{2}^{o}\left({\overset{ˇ}{H}}_{2};\theta_{2}\right)\right\}\right].$$

using definitions of $\omega_{t}\left({\overset{ˇ}{H}}_{t},A_{t};\Theta_{t}\right)\;t=1, 2$ we can write:

$$\text{Bias}\left(\bar{V},{𝒱}_{{\text{SUP}}_{\text{DR}}}(L;\Theta )\right)\;=𝔼\left[Q_{1}^{o}\left({\overset{ˇ}{H}}_{1}\right)-Q_{1}^{o}\left({\overset{ˇ}{H}}_{1};\theta_{1}\right)\right]\;-𝔼\left[\left\{\frac{{\bar{d}}_{1}A_{1}}{\pi_{1}\left({\overset{ˇ}{H}}_{1};\xi_{1}\right)}+\frac{\left(1-{\bar{d}}_{1}\right)\left(1-A_{1}\right)}{1-\pi_{1}\left({\overset{ˇ}{H}}_{1};\xi_{1}\right)}\right\}\;\left\{Q_{1}^{o}\left({\overset{ˇ}{H}}_{1}\right)-Q_{1}^{o}\left({\overset{ˇ}{H}}_{1};\theta_{1}\right)\right\}\right]\;-𝔼\left[\frac{{\bar{d}}_{1}A_{1}}{\pi_{1}\left({\overset{ˇ}{H}}_{1};\xi_{1}\right)}\left\{Q_{2}^{o}\left({\overset{ˇ}{H}}_{2}\right)-Q_{2}^{o}\left({\overset{ˇ}{H}}_{2};\theta_{2}\right)\right\}\right]-𝔼\left[\frac{\left(1-{\bar{d}}_{1}\right)\left(1-A_{1}\right)}{1-\pi_{1}\left({\overset{ˇ}{H}}_{1};\xi_{1}\right)}\left\{Q_{2}^{o}\left({\overset{ˇ}{H}}_{2}\right)-Q_{2}^{o}\left({\overset{ˇ}{H}}_{2};\theta_{2}\right)\right\}\right]\;-𝔼\left[\left\{\frac{{\bar{d}}_{1}A_{1}}{\pi_{1}\left({\overset{ˇ}{H}}_{1};\xi_{1}\right)}+\frac{\left(1-{\bar{d}}_{1}\right)\left(1-A_{1}\right)}{1-\pi_{1}\left({\overset{ˇ}{H}}_{1};\xi_{1}\right)}\right\}\;\left\{\frac{{\bar{d}}_{2}A_{2}}{\pi_{2}\left({\overset{ˇ}{H}}_{2};\xi_{2}\right)}+\frac{\left(1-{\bar{d}}_{2}\right)\left(1-A_{2}\right)}{1-\pi_{2}\left({\overset{ˇ}{H}}_{2};\xi_{2}\right)}\right\}\;\times \left\{Q_{2}^{o}\left({\overset{ˇ}{H}}_{2}\right)-Q_{2}^{o}\left({\overset{ˇ}{H}}_{2};\theta_{2}\right)\right\}\right]$$

assuming $A_{1}⊥A_{2}|H_{2},Y_{2}$, we use iterated expectations:

$$\text{Bias}\left(\bar{V},{𝒱}_{{\mathrm{SUP}}_{\mathrm{DR}}}(L;\Theta )\right)\;=𝔼\left[Q_{1}^{o}\left({\overset{ˇ}{H}}_{1}\right)-Q_{1}^{o}\left({\overset{ˇ}{H}}_{1};\theta_{1}\right)\right]\;-𝔼\left[𝔼\left[\left\{\frac{{\bar{d}}_{1}A_{1}}{\pi_{1}\left({\overset{ˇ}{H}}_{1};\xi_{1}\right)}+\frac{\left(1-{\bar{d}}_{1}\right)\left(1-A_{1}\right)}{1-\pi_{1}\left({\overset{ˇ}{H}}_{1};\xi_{1}\right)}\right\}\;\left\{Q_{1}^{o}\left({\overset{ˇ}{H}}_{1}\right)-Q_{1}^{o}\left({\overset{ˇ}{H}}_{1};\theta_{1}\right)\right\}|{\overset{ˇ}{H}}_{1}\right]\right]\;-𝔼\left[\left\{\frac{{\bar{d}}_{1}A_{1}}{\pi_{1}\left({\overset{ˇ}{H}}_{1};\xi_{1}\right)}+\frac{\left(1-{\bar{d}}_{1}\right)\left(1-A_{1}\right)}{1-\pi_{1}\left({\overset{ˇ}{H}}_{1};\xi_{1}\right)}\right\}\;\left\{Q_{2}^{o}\left({\overset{ˇ}{H}}_{2}\right)-Q_{2}^{o}\left({\overset{ˇ}{H}}_{2};\theta_{2}\right)\right\}\right]\;-𝔼[𝔼\left[\left\{\frac{{\bar{d}}_{1}A_{1}}{\pi_{1}\left({\overset{ˇ}{H}}_{1};\xi_{1}\right)}+\frac{\left(1-{\bar{d}}_{1}\right)\left(1-A_{1}\right)}{1-\pi_{1}\left({\overset{ˇ}{H}}_{1};\xi_{1}\right)}\right\}\;\left\{\frac{{\bar{d}}_{2}A_{2}}{\pi_{2}\left({\overset{ˇ}{H}}_{2};\xi_{2}\right)}+\frac{\left(1-{\bar{d}}_{2}\right)\left(1-A_{2}\right)}{1-\pi_{2}\left({\overset{ˇ}{H}}_{2};\xi_{2}\right)}\right\}\;\times \left\{Q_{2}^{o}\left({\overset{ˇ}{H}}_{2}\right)-Q_{2}^{o}\left({\overset{ˇ}{H}}_{2};\theta_{2}\right)\right\}|{\overset{ˇ}{H}}_{2}]\right]\;=𝔼\left[Q_{1}^{o}\left({\overset{ˇ}{H}}_{1}\right)-Q_{1}^{o}\left({\overset{ˇ}{H}}_{1};\theta_{1}\right)\right]\;-𝔼\left[\left\{\frac{{\bar{d}}_{1}\pi_{1}\left({\overset{ˇ}{H}}_{1}\right)}{\pi_{1}\left({\overset{ˇ}{H}}_{1};\xi_{1}\right)}+\frac{\left\{1-{\bar{d}}_{1}\right\}\left\{1-\pi_{1}\left({\overset{ˇ}{H}}_{1}\right)\right\}}{1-\pi_{1}\left({\overset{ˇ}{H}}_{1};\xi_{1}\right)}\right\}\;\left\{Q_{1}^{o}\left({\overset{ˇ}{H}}_{1}\right)-Q_{1}^{o}\left({\overset{ˇ}{H}}_{1};\theta_{1}\right)\right\}\right]\;-𝔼\left[\left\{\frac{{\bar{d}}_{1}A_{1}}{\pi_{1}\left({\overset{ˇ}{H}}_{1};\xi_{1}\right)}+\frac{\left(1-{\bar{d}}_{1}\right)\left(1-A_{1}\right)}{1-\pi_{1}\left({\overset{ˇ}{H}}_{1};\xi_{1}\right)}\right\}\;\left\{Q_{2}^{o}\left({\overset{ˇ}{H}}_{2}\right)-Q_{2}^{o}\left({\overset{ˇ}{H}}_{2};\theta_{2}\right)\right\}\right]\;-𝔼\left[\left\{\frac{{\bar{d}}_{1}A_{1}}{\pi_{1}\left({\overset{ˇ}{H}}_{1};\xi_{1}\right)}+\frac{\left(1-{\bar{d}}_{1}\right)\left(1-A_{1}\right)}{1-\pi_{1}\left({\overset{ˇ}{H}}_{1};\xi_{1}\right)}\right\}\;\left\{\frac{{\bar{d}}_{2}\pi_{2}\left({\overset{ˇ}{H}}_{2}\right)}{\pi_{2}\left({\overset{ˇ}{H}}_{2};\xi_{2}\right)}+\frac{\left\{1-{\bar{d}}_{2}\right\}\left\{1-\pi_{2}\left({\overset{ˇ}{H}}_{2}\right)\right\}}{1-\pi_{2}\left({\overset{ˇ}{H}}_{2};\xi_{2}\right)}\right\}\;\times \left\{Q_{2}^{o}\left({\overset{ˇ}{H}}_{2}\right)-Q_{2}^{o}\left({\overset{ˇ}{H}}_{2};\theta_{2}\right)\right\}\right]$$

finally, factorizing common terms:

$$\text{Bias}\left(\bar{V},{𝒱}_{{\text{Sup}}_{\text{DR}}}(L;\Theta )\right)\;=𝔼\left[{\bar{d}}_{1}\left\{1-\frac{\pi_{1}\left({\overset{ˇ}{H}}_{1}\right)}{\pi_{1}\left({\overset{ˇ}{H}}_{1};\xi_{1}\right)}\right\}\;\left\{Q_{1}^{o}\left({\overset{ˇ}{H}}_{1}\right)-Q_{1}^{o}\left({\overset{ˇ}{H}}_{1};\theta_{1}\right)\right\}\right]\;+𝔼\left[\left\{1-{\bar{d}}_{1}\right\}\left\{1-\frac{1-\pi_{1}\left({\overset{ˇ}{H}}_{1}\right)}{1-\pi_{1}\left({\overset{ˇ}{H}}_{1};\xi_{1}\right)}\right\}\;\left\{Q_{1}^{o}\left({\overset{ˇ}{H}}_{1}\right)-Q_{1}^{o}\left({\overset{ˇ}{H}}_{1};\theta_{1}\right)\right\}\right]\;+𝔼\left[{\bar{d}}_{2}\left\{\frac{A_{1}}{\pi_{1}\left({\overset{ˇ}{H}}_{1};\xi_{1}\right)}+\frac{1-A_{1}}{1-\pi_{1}\left({\overset{ˇ}{H}}_{1};\xi_{1}\right)}\right\}\;\left\{1-\frac{\pi_{2}\left({\overset{ˇ}{H}}_{2}\right)}{\pi_{2}\left({\overset{ˇ}{H}}_{2};\xi_{2}\right)}\right\}\;\left\{Q_{2}^{o}\left({\overset{ˇ}{H}}_{2}\right)-Q_{2}^{o}\left({\overset{ˇ}{H}}_{2};\theta_{2}\right)\right\}\right]\;+𝔼\left[\left\{1-{\bar{d}}_{2}\right\}\left\{\frac{A_{1}}{\pi_{1}\left({\overset{ˇ}{H}}_{1};\xi_{1}\right)}+\frac{1-A_{1}}{1-\pi_{1}\left({\overset{ˇ}{H}}_{1};\xi_{1}\right)}\right\}\;\left\{1-\frac{1-\pi_{2}\left({\overset{ˇ}{H}}_{2}\right)}{1-\pi_{2}\left({\overset{ˇ}{H}}_{2};\xi_{2}\right)}\right\}\;\left\{Q_{2}^{o}\left({\overset{ˇ}{H}}_{2}\right)-Q_{2}^{o}\left({\overset{ˇ}{H}}_{2};\theta_{2}\right)\right\}\right]\;\leq \sqrt{\underset{{\overset{ˇ}{H}}_{1}}{\sup}\left|{\left\{1-\pi_{1}\left({\overset{ˇ}{H}}_{1};\xi_{1}\right)\right\}}^{-1}\right|}\sqrt{{\left\|\pi_{1}\left({\overset{ˇ}{H}}_{1};\xi_{1}\right)-\pi_{1}\left({\overset{ˇ}{H}}_{1}\right)\right\|}_{L_{2}(\mathbb{P})}}\sqrt{{\left\|Q_{1}^{o}\left({\overset{ˇ}{H}}_{1};{\hat{\theta }}_{1}\right)-Q_{1}^{o}\left({\overset{ˇ}{H}}_{1}\right)\right\|}_{L_{2}(\mathbb{P})}}\;+\sqrt{\underset{{\overset{ˇ}{H}}_{2}}{\sup}\left|\left\{\frac{A_{1}}{\pi_{1}\left({\overset{ˇ}{H}}_{1};\xi_{1}\right)}+\frac{1-A_{1}}{1-\pi_{1}\left({\overset{ˇ}{H}}_{1};\xi_{1}\right)}\right\}\;{\left\{1-\pi_{1}\left({\overset{ˇ}{H}}_{1};\xi_{1}\right)\right\}}^{-1}{\left\{1-\pi_{2}\left({\overset{ˇ}{H}}_{2};\xi_{2}\right)\right\}}^{-1}\right|}\;\times \sqrt{{\left\|\pi_{2}\left({\overset{ˇ}{H}}_{2};\xi_{2}\right)-\pi_{2}\left({\overset{ˇ}{H}}_{2}\right)\right\|}_{L_{2}(\mathbb{P})}}\sqrt{{\left\|Q_{2}^{o}\left({\overset{ˇ}{H}}_{2};{\hat{\theta }}_{2}\right)-Q_{2}^{o}\left({\overset{ˇ}{H}}_{2}\right)\right\|}_{L_{2}(\mathbb{P})}},$$

which follows by Cauchy–Schwarz Inequality. ■

## Appendix F. Additional Theoretical Results

### F.1 Augmented Value Function Estimation

We first re-write Assumption 5 to account for only using sample ℒ in estimation of the *Q* functions and propensity scores.

**Assumption 10** *Define the following class of functions:*

(21)
$${𝒬}_{1}\equiv \left\{Q_{1}\left(H_{1},A_{1};\theta_{1}\right)|\theta_{1}\in {\text{Θ}}_{1}\subset {\mathbb{R}}^{q_{1}}\right\},\;{𝒬}_{2}\equiv \left\{Q_{2}\left(H_{2},A_{2},Y_{2};\theta_{2}\right)|\theta_{2}\in {\text{Θ}}_{2}\subset {\mathbb{R}}^{q_{2}}\right\},\;{𝒲}_{1}\equiv \left\{\pi_{1}\left(H_{1};\xi_{1}\right)|\xi_{1}\in {\text{Ω}}_{1}\subset {\mathbb{R}}^{p_{1}}\right\},\;{𝒲}_{2}\equiv \left\{\pi_{2}\left({\overset{ˇ}{H}}_{2};\xi_{2}\right)|\xi_{2}\in {\text{Ω}}_{2}\subset {\mathbb{R}}^{p_{2}}\right\},$$

*with* $p_{1},p_{2},q_{1},q_{2}$ *fixed under model definitions* (1) & (4). *Let the population equations* $𝔼\left[S_{t}^{\xi }\left(\xi_{t}\right)\right]=0,\;t=1, 2$ *have solutions* ${\bar{\xi }}_{1},{\bar{\xi }}_{2}$, *where*

$$S_{1}^{\xi }\left(\xi_{1}\right)=\frac{\partial }{\partial \xi_{1}}\text{log}\left[\pi_{1}{\left(H_{1};\xi_{1}\right)}^{A_{1}}{\left(1-\pi_{1}\left(H_{1};\xi_{1}\right)\right)}^{\left(1-A_{1}\right)}\right],\;S_{2}^{\xi }\left(\xi_{2}\right)=\frac{\partial }{\partial \xi_{2}}\text{log}\left[\pi_{2}{\left({\overset{ˇ}{H}}_{2};\xi_{2}\right)}^{A_{2}}{\left(1-\pi_{2}\left({\overset{ˇ}{H}}_{2};\xi_{2}\right)\right)}^{\left(1-A_{2}\right)}\right],$$

*and the population equations for the Q functions* $𝔼\left[S_{t}^{\theta }\left(\theta_{t}\right)\right]=0,t=1,2$ *have solutions* ${\bar{\theta }}_{1},{\bar{\theta }}_{2}$, *where*

$$S_{2}^{\theta }\left(\theta_{2}\right)=\frac{\partial }{\partial \theta_{2}^{\text{T}}}{\left\|Y_{3}-Q_{2}\left({\overset{ˇ}{H}}_{2},A_{2};\theta_{2}\right)\right\|}_{2}^{2},\;S_{1}^{\theta }\left(\theta_{1}\right)=\frac{\partial }{\partial \theta_{1}^{\text{T}}}{\left\|Y_{2}+{\bar{Q}}_{2}\left({\overset{ˇ}{H}}_{2};{\bar{\theta }}_{2}\right)-Q_{1}\left(H_{1},A_{1};\theta_{1}\right)\right\|}_{2}^{2},$$

*(i)* $\xi_{1},\xi_{2}$ *are bounded sets. (ii)* $\Theta_{1},\Theta_{2}$ *are open bounded sets and for some* r>0 *and* $g_{t}(\cdot )$

(22)
$$\left|Q_{t}\left(\cdot ;\theta_{t}\right)-Q_{t}\left(\cdot ;\theta_{t}^{'}\right)\right|\leq g_{t}(\cdot )\left\|\theta_{t}-\theta_{t}^{'}\right\|\forall \theta_{t},\theta_{t}^{'}\in {\text{Θ}}_{t},𝔼\left[{\left|g_{t}(\cdot )\right|}^{r}\right]<\infty ,t=1,2.$$

*(iii) The population minimizers satisfy* ${\bar{\theta }}_{t}\in \Theta_{t},{\bar{\xi }}_{t}\in \Omega_{t},t=1,2$. *(iv) For* ${\bar{\xi }}_{t},t=1,2$, ${\overset{‾}{\pi }}_{1}\left(H_{1};{\bar{\xi }}_{1}\right)>0,{\overset{‾}{\pi }}_{2}\left({\overset{ˇ}{H}}_{2};{\bar{\xi }}_{2}\right)>0\forall H\in ℋ$.

Existence of solutions ${\bar{\theta }}_{t}\in \Theta_{t},t=1,2$ is clear as $\Theta_{1},\Theta_{2}$ are open and bounded.

**Theorem 19 (Asymptotic Normality for** ${\hat{V}}_{{SSL}_{DR}}$) *Under Assumptions 1, 4, and 10*, ${\hat{V}}_{{\text{SUP}}_{\text{DR}}}$ *as defined in* (5) *is such that*

$$\sqrt{n}\left\{{\hat{V}}_{{\text{SUP}}_{\text{DR}}}-{𝔼}_{𝕊}\left[{𝒱}_{{\text{SUP}}_{\text{DR}}}(L;\bar{\Theta })\right]\right\}=\frac{1}{\sqrt{n}}\sum_{i=1}^{n}\psi_{{\text{SUP}}_{\text{DR}}}^{v}\left(L_{i};\bar{\Theta }\right)+o_{\mathbb{P}}(1)\overset{d}{\to }N\left(0,\sigma_{{\text{SUP}}_{\text{DR}}}^{2}\right).$$

*where*

$$\psi_{{\text{SUP}}_{\text{DR}}}^{v}(L;\bar{\Theta })={𝒱}_{{\text{SUP}}_{\text{DR}}}(L;\bar{\Theta })-{𝔼}_{𝕊}\left[{𝒱}_{{\text{SUP}}_{\text{DR}}}(L;\bar{\Theta })\right]+{\psi_{\text{SUP}}^{\theta }{\left(L\right)}^{⊤}\frac{\partial }{\partial \theta }\int {𝒱}_{{\text{SUP}}_{\text{DR}}}(L;\bar{\Theta })d{\mathbb{P}}_{L}|}_{\Theta =\bar{\Theta }}\;+{\psi_{\text{SUP}}^{\xi }{\left(L\right)}^{⊤}\frac{\partial }{\partial \xi }\int {𝒱}_{{\text{SUP}}_{\text{DR}}}(L;\bar{\Theta })d{\mathbb{P}}_{L}|}_{\Theta =\bar{\Theta }},\;\sigma_{{\text{SUP}}_{\text{DR}}}^{2}=𝔼\left[\psi_{{\text{SUP}}_{\text{DR}}}^{v}{\left(L;\bar{\Theta }\right)}^{2}\right].$$

**Proof** [proof of theorem 19]

Letting $g(\Theta )=\int {𝒱}_{{\text{SUP}}_{\text{DR}}}(L;\Theta )d{\mathbb{P}}_{L}$, we start by centering (5) and scaling by $\sqrt{n}$:

$$\sqrt{n}\left\{{\mathbb{P}}_{n}\left({𝒱}_{{\text{SUP}}_{\text{DR}}}\left(L;{\hat{\Theta }}_{\text{SUP}}\right)\right)-𝔼\left[{𝒱}_{{\text{SUP}}_{\text{DR}}}(L;\bar{\Theta })\right]\right\}\;={𝔾}_{n}\left\{{𝒱}_{{\text{SUP}}_{\text{DR}}}(L;\bar{\Theta })\right\}+{𝔾}_{n}\left\{{𝒱}_{{\text{SUP}}_{\text{DR}}}\left(L;{\hat{\Theta }}_{\text{SUP}}\right)-{𝒱}_{{\text{SUP}}_{\text{DR}}}(L;\bar{\Theta })\right\}+\sqrt{n}\left\{g\left({\hat{\Theta }}_{\text{SUP}}\right)-g(\bar{\Theta })\right\}$$

#### I) Empirical Process Term

We first show that under Assumption 10, ${𝔾}_{n}\left\{{𝒱}_{{\mathrm{SUP}}_{DR}}\left(L;{\hat{\Theta }}_{\mathrm{SUP}}\right)-{𝒱}_{{\mathrm{SUP}}_{DR}}(L;\bar{\Theta })\right\}=o_{\mathbb{P}}(1)$, let

$$f_{\Theta }(\vec{U})=Q_{1}^{o}\left({\overset{ˇ}{H}}_{1};\theta_{1}\right)+\omega_{1}\left({\overset{ˇ}{H}}_{1},A_{1},\Theta \right)\left\{Y_{2}-Q_{1}^{o}\left({\overset{ˇ}{H}}_{1};\theta_{1}\right)+Q_{2}^{o}\left({\overset{ˇ}{H}}_{2};\theta_{2}\right)\right\}\;\;+\omega_{2}\left({\overset{ˇ}{H}}_{1},A_{1};\Theta \right)\left\{Y_{3}-Q_{2}^{o}\left({\overset{ˇ}{H}}_{2};\theta_{2}\right)\right\},$$

we define the class of functions ${𝒞}_{3}=\left\{f_{\Theta }(\vec{U})|\vec{U},\Theta \in 𝒮(\delta )\right\}$, and

$$\ell =\left\{l\;:\;{\left\{0,1\right\}}^{2}\mapsto \{0,1\}\right\}.$$

i) By Assumptions 10 and Theorem 19.5 in Vaart (1998), $\ell ,{𝒲}_{t},{𝒬}_{t},\;t=1, 2$ are a ℙ-Donsker class, thus it follows that ${𝒞}_{3}$ is a Donsker class.

ii) We estimate $\xi_{1},\xi_{2}$ from (21) with their maximum likelihood estimator ${\hat{\xi }}_{\text{1SUP}},{\hat{\xi }}_{\text{2SUP}}$, solving ${\mathbb{P}}_{n}\left[S_{t}\left(\xi_{t}\right)\right]=0,\;t=1, 2$ and estimate functions $\pi_{1}\left(H_{1};{\hat{\xi }}_{\text{1SUP}}\right),\;\pi_{2}\left({\overset{ˇ}{H}}_{2};{\hat{\xi }}_{\text{2SUP}}\right)$ with ${\hat{\xi }}_{\text{1SUP}},{\hat{\xi }}_{\text{2SUP}}$. By Assumption 10 and weak law of large numbers ${\hat{\xi }}_{t\text{SUP}}\overset{p}{\to }{\bar{\xi }}_{t},t=1, 2$.

Analogous, under regularity conditions, (2) have unique solutions ${\hat{\theta }}_{t\text{SUP}}$ for which ${\hat{\theta }}_{t\text{SUP}}\overset{p}{\to }{\bar{\theta }}_{t},t=1,2$ by Assumption 10 and weak law of large numbers. Both regardless of whether models (1) & (4) are correct. Thus $\mathbb{P}\left({\hat{\Theta }}_{\text{SUP}}\in 𝒮(\delta )\right)\to 1,\forall \delta$.

iii) We next show $\int \;{\left\{{𝒱}_{{\text{SUP}}_{\text{DR}}}\left(L;{\hat{\Theta }}_{\text{SUP}}\right)-{𝒱}_{{\text{SUP}}_{\text{DR}}}(L;\bar{\Theta })\right\}}^{2}d{\mathbb{P}}_{L}⟶0$. Using (7), for a large enough constant c we can write

$$\int \;{\left\{{𝒱}_{{\text{SUP}}_{\text{DR}}}\left(L;{\hat{\Theta }}_{\text{SUP}}\right)-{𝒱}_{{\text{SUP}}_{\text{DR}}}(L;\bar{\Theta })\right\}}^{2}d{\mathbb{P}}_{L}\;\leq c\;\underset{H_{1}}{\sup}{\left(H_{10}^{⊤}{\bar{\beta }}_{1}+{\left[H_{11}^{⊤}{\bar{\gamma }}_{1}\right]}_{+}-H_{10}^{⊤}{\hat{\beta }}_{\mathrm{1SUP}}-{\left[H_{11}^{⊤}{\hat{\gamma }}_{\mathrm{1SUP}}\right]}_{+}\right)}^{2}\;+c\;\underset{{\overset{˘}{H}}_{2}}{\sup}{\left({\overset{ˇ}{H}}_{20}^{⊤}{\bar{\beta }}_{2}+{\left[H_{21}^{⊤}{\bar{\gamma }}_{2}\right]}_{+}-{\overset{ˇ}{H}}_{20}^{⊤}{\hat{\beta }}_{\mathrm{2SUP}}-{\left[H_{21}^{⊤}{\hat{\gamma }}_{\mathrm{2SUP}}\right]}_{+}\right)}^{2}\;+c\;\underset{H_{1},A_{1}}{\sup}{\left\{\omega_{1}\left(H_{1},A_{1};{\hat{\Theta }}_{\mathrm{1SUP}}\right)-\omega_{1}\left(H_{1},A_{1};{\bar{\Theta }}_{1}\right)\right\}}^{2}+{\left({\hat{\beta }}_{21\text{SUP}}-{\bar{\beta }}_{21}\right)}^{2}\;\to 0$$

where we use $(a-b{)}^{2},(a+b{)}^{2}\leq 2a^{2}+2b^{2}\forall a,b\in \mathbb{R}$, boundedness of $\bar{\Theta }$ and covariates by Assumptions 1, 2, and 10. Next, from assumption (7) it can be shown that ${\hat{\theta }}_{\mathrm{2SUP}}-{\bar{\theta }}_{2}=O_{\mathbb{P}}\left(n^{-\frac{1}{2}}\right),{\hat{\theta }}_{\mathrm{1SUP}}-{\bar{\theta }}_{1}=O_{\mathbb{P}}\left(n^{-\frac{1}{2}}\right)$, also from Lemma 17 (a) it follows that for t=1, 2

$$\underset{{\overset{ˇ}{H}}_{t}}{\sup}{\left(H_{t0}^{⊤}{\bar{\beta }}_{t}+{\left[H_{t1}^{⊤}{\bar{\gamma }}_{t}\right]}_{+}-H_{t0}^{⊤}{\hat{\beta }}_{t\text{SUP}}-{\left[H_{t1}^{⊤}{\hat{\gamma }}_{t\text{SUP}}\right]}_{+}\right)}^{2}\;\leq 2\underset{{\overset{ˇ}{H}}_{t0}}{\sup}{\left\|{\overset{ˇ}{H}}_{t0}\right\|}_{2}^{2}{\left\|{\hat{\beta }}_{t}-{\bar{\beta }}_{t}\right\|}_{2}^{2}+2\underset{H_{t1}}{\sup}{\left\|H_{t1}\right\|}_{2}^{2}{\left\|{\hat{\gamma }}_{t}-{\bar{\gamma }}_{t}\right\|}_{2}\;=O_{\mathbb{P}}\left(n^{-1}\right).$$

Next, we can write

$$\omega_{1}\left(H_{1},A_{1};{\hat{\Theta }}_{\mathrm{1SUP}}\right)=I\left\{A_{1}=d_{1}\left(H_{1};{\hat{\xi }}_{\mathrm{1SUP}}\right)\right\}\;\left\{\frac{A_{1}}{\pi_{1}\left(H_{1};{\hat{\xi }}_{\mathrm{1SUP}}\right)}+\frac{1-A_{1}}{1-\pi_{1}\left(H_{1};{\hat{\xi }}_{\mathrm{1SUP}}\right)}\right\}.$$

By Lemma 17 (b) it follows that

$$\underset{H_{1}}{\sup}\left|\frac{1}{\pi_{1}\left(H_{1};{\hat{\xi }}_{1\text{sup}}\right)}-\frac{1}{\pi_{1}\left(H_{1};{\bar{\xi }}_{1}\right)}\right|=O_{\mathbb{P}}\left(n^{-\frac{1}{2}}\right).$$

Using the above and Lemma 14 we get

$$\underset{{\overset{ˇ}{H}}_{1},A_{1}}{\sup}{\left\{\omega_{1}\left({\overset{ˇ}{H}}_{1},A_{1};{\hat{\Theta }}_{\mathrm{1SUP}}\right)-\omega_{1}\left({\overset{ˇ}{H}}_{1},A_{1};{\bar{\Theta }}_{1}\right)\right\}}^{2}=o_{\mathbb{P}}(1),$$

which gives us $\int {\left\{{𝒱}_{{\text{SUP}}_{\text{DR}}}\left(L;{\hat{\Theta }}_{\text{SUP}}\right)-{𝒱}_{{\text{SUP}}_{\text{DR}}}(L;\bar{\Theta })\right\}}^{2}d{\mathbb{P}}_{L}⟶0$.

Hence, we have i) $\mathbb{P}\left({\hat{\Theta }}_{\text{SUP}}\in 𝒮(\delta )\right)\to 1,\forall \delta$, ii) ${𝒞}_{1}$ is a Donsker class, and iii) $\int \;{\left\{{𝒱}_{{\text{SUP}}_{\text{DR}}}\left(L;{\hat{\Theta }}_{\text{SUP}}\right)-{𝒱}_{{\text{SUP}}_{\text{DR}}}(L;\bar{\Theta })\right\}}^{2}d{\mathbb{P}}_{L}⟶0$, then by Theorem 2.1 in Van Der Vaart and Wellner (2007)

$$\sqrt{n}\left[{\mathbb{P}}_{n}\left\{{𝒱}_{{\text{SUP}}_{\text{DR}}}\left(L;{\hat{\Theta }}_{\text{SUP}}\right)-g\left({\hat{\Theta }}_{\text{SUP}}\right)\right\}-{\mathbb{P}}_{n}\left\{{𝒱}_{{\text{SUP}}_{\text{DR}}}(L;\bar{\Theta })-g(\bar{\Theta })\right\}\right]=o_{\mathbb{P}}(1).$$

#### Centered Sample Average

Next we consider ${𝔾}_{n}\left\{{𝒱}_{{\text{SUP}}_{\text{DR}}}(L;\bar{\Theta })\right\}$. Note that ${𝒱}_{{\text{SUP}}_{\text{DR}}}(L;\bar{\Theta })$ is a deterministic function of random variable **L** as parameters are fixed. We have that $𝔼\left[\left({𝒱}_{{\text{SUP}}_{\text{DR}}}(L;\bar{\Theta }{)}^{2}\right]<\infty \right.$ holds by Assumption 1 & 10. Thus the central limit theorem yields

$${𝔾}_{n}\left\{{𝒱}_{{\text{SUP}}_{\text{DR}}}(L;\bar{\Theta })\right\}\overset{d}{\to }𝒩\left(0,\mathrm{Var}\left[{𝒱}_{{\text{SUP}}_{\text{DR}}}(L;\bar{\Theta })\right]\right).$$

#### Bias Term

We finally analyze the bias: $\sqrt{n}\left\{g\left({\hat{\Theta }}_{\text{SUP}}\right)-g(\bar{\Theta })\right\}$. Using a Taylor series expansion

$$g\left({\hat{\Theta }}_{\text{SUP}}\right)=g(\bar{\Theta })+{\left({\hat{\theta }}_{\text{SUP}}-\bar{\theta }\right)}^{⊤}\frac{\partial }{\partial \theta_{\text{SUP}}}g(\bar{\Theta })+{\left({\hat{\xi }}_{\text{SUP}}-\bar{\xi }\right)}^{⊤}\frac{\partial }{\partial \xi_{\text{SUP}}}g(\bar{\Theta })+O_{\mathbb{P}}\left(n^{-1}\right),$$

therefore

$$\sqrt{n}\left\{g\left({\hat{\Theta }}_{\text{SUP}}\right)-g(\bar{\Theta })\right\}=\sqrt{n}{\left({\hat{\theta }}_{\text{SUP}}-\bar{\theta }\right)}^{⊤}\frac{\partial }{\partial \theta_{\text{SUP}}}g(\bar{\Theta })+\sqrt{n}{\left({\hat{\xi }}_{\text{SUP}}-\bar{\xi }\right)}^{⊤}\frac{\partial }{\partial \xi_{\text{SUP}}}g(\bar{\Theta })+o_{\mathbb{P}}(1).$$

Using the *Q* function and propensity score function influence functions we can write

$$\sqrt{n}\left\{g\left({\hat{\Theta }}_{\text{SUP}}\right)-g(\bar{\Theta })\right\}=\frac{\partial }{\partial \theta_{\text{SUP}}}g(\bar{\Theta })\frac{1}{\sqrt{n}}\sum_{i=1}^{n}\psi_{\text{SUP}}^{\theta }\left(L_{i}\right)+\frac{\partial }{\partial \xi }g(\bar{\Theta })\frac{1}{\sqrt{n}}\sum_{i=1}^{n}\psi_{\text{SUP}}^{\xi }\left(L_{i}\right)+o_{\mathbb{P}}(1)$$

■
